# Mild Direct Remote Hydroxylation of Enals in Air

**DOI:** 10.1021/acs.joc.6c00095

**Published:** 2026-03-11

**Authors:** Fernanda Liu, Alessandro Vicidomini, Stacey E. Brenner-Moyer

**Affiliations:** Department of Chemistry, 67206 Rutgers University-Newark, 73 Warren Street, Newark, New Jersey 07102, United States

## Abstract

A mild direct remote hydroxylation of α,β-unsaturated aldehydes is reported. Air is the oxidant, and tertiary alcohol products are generated in up to 80% yield. This method selectively hydroxylates enals with acidic γ-carbons and therefore may be suitable for late-stage installation of remote hydroxyl groups on medicinal aldehydes. Mechanistic studies that enabled the development of this transformation are described, as is the first example of catalytic enantioselective γ-hydroxylation of an enal.

## Graphical abstract

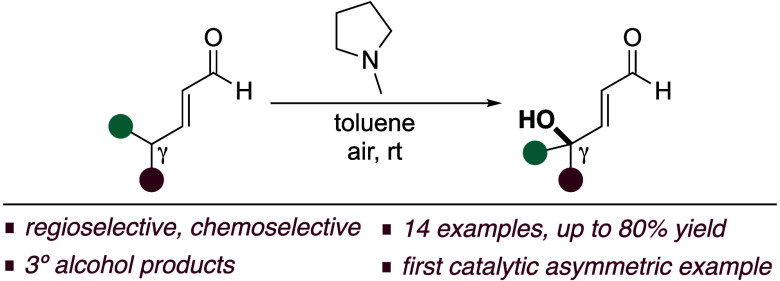

γ-Hydroxyenals (i.e., **7**, Scheme 1) are versatile synthetic building blocks for natural products containing 3° alcohols,^1^ which are also attractive motifs in medicinal chemistry.^2^ Additionally, γ-hydroxyenals afford rapid access to heterocycles, such as γ-butenolides, γ-lactones, and substituted tetrahydrofurans, and acylation of **7** provides substrates for NHC-catalyzed installation of diverse functionality at the remote γ-position.^3^ Accessing these valuable substructures directly from α,β-unsaturated aldehydes via remote oxygenation would be ideal and could facilitate late-stage functionalization of enal-containing complex molecules. Despite the utility of such methods, few exist. While organocatalytic methods that are presumed to proceed via dienamine intermediates (e.g., **3**) have been developed for γ-oxygenation of enals (Scheme 1A), none directly provide the free hydroxylated product.^4^ Similarly, an umpolung method employing catalytic Brønsted acid introduces ether functionality at the γ-position of enal substrates that, additionally, require α-substitution for desired reactivity.^5^ Recently, a method entailing Cu catalysis was reported (Scheme 1B),^6^ in which Cu is proposed to serve as a Lewis acid catalyst, activating the enal toward deprotonation by the strong base tetramethylguanidine (TMG). Reaction of the resulting Cu dienolate with ^3^O_2_ from air, and reduction of the resulting perhydroxylated intermediate **6-Cu** by PPh_3_, provides γ-hydroxylated enal **7**. Unsaturated esters, ketones, amides, nitriles, and sulfones also underwent hydroxylation under these conditions. Most recently, a photocatalytic visible-light-induced HAT method was demonstrated to work nicely for 40 enone substrates.^7^ The single enal substrate examined, which also had α substitution, generated the product in 27% yield. We present herein our discovery of a mild direct remote hydroxylation of enals in air (Scheme 1C), requiring only a stoichiometric 3° amine that plays a dual role in this transformation.

**Scheme 1:**
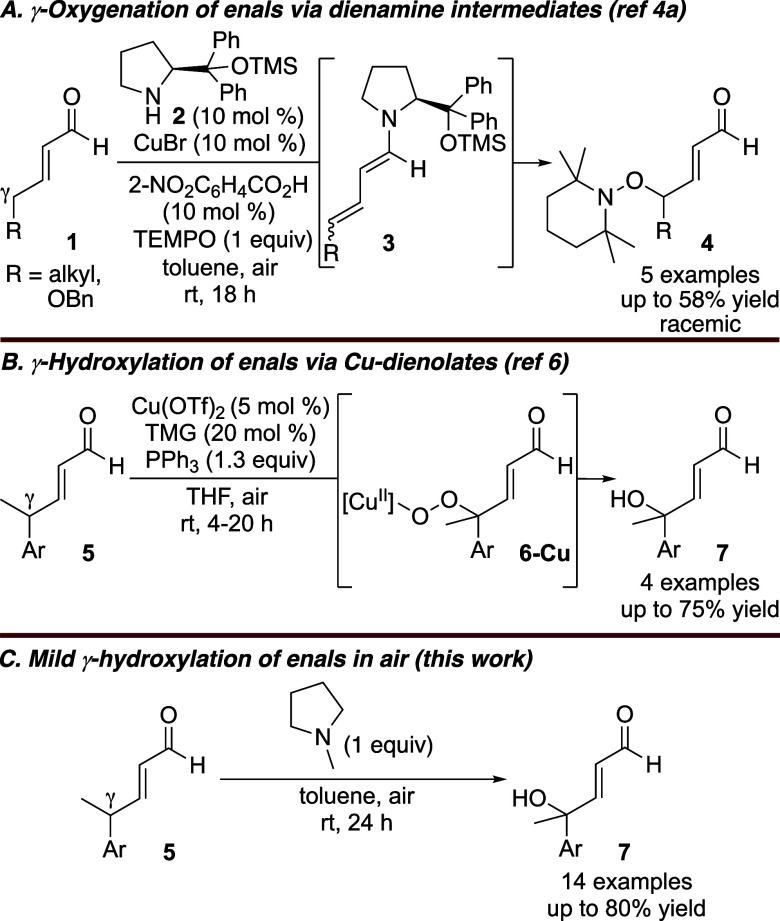
Methods for Direct γ-Oxygenation of Enals

These investigations were initiated after the unexpected observation of γ-oxygenated enal products **9** and **10** in a high combined yield from **8** under the conditions illustrated in Scheme 2. We presumed that these products arose from the reaction of O_2_ with a dienamine intermediate (**11**), generated through enamine formation from **8** by virtue of the preexisting β,γ-unsaturation. Recognizing that dienamine **11** could alternatively be accessed from the corresponding α,β-unsaturated aldehyde and that direct γ-oxygenation of enals is of great synthetic value, further development of this transformation was pursued.

**Scheme 2:**
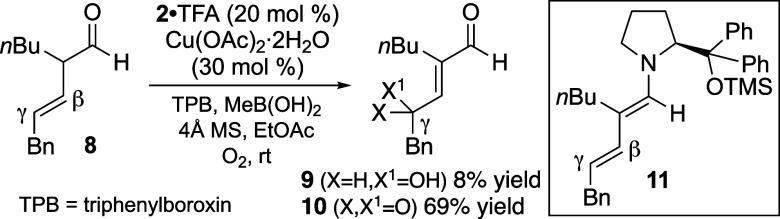
Initial Observation

Using substrate **5a,** which contained a γ-Me group to prevent overoxidation and lacked a steric blocking α-substituent, product **7a** was generated in comparable yield to **9** under related reaction conditions (entry 1, Table 1). Switching the acid additive to benzoic acid doubled the product yield (entry 2). To our surprise, Cu was not required for reactivity (entry 3). Control experiments revealed that the observed reactivity in the absence of Cu arose neither from an adventitious radical species^8^ nor from trace metal impurities.^9,10^ Since products were racemic under these conditions, use of an achiral 2° amine catalyst, **12**, was examined (entry 4).

**Table 1:**
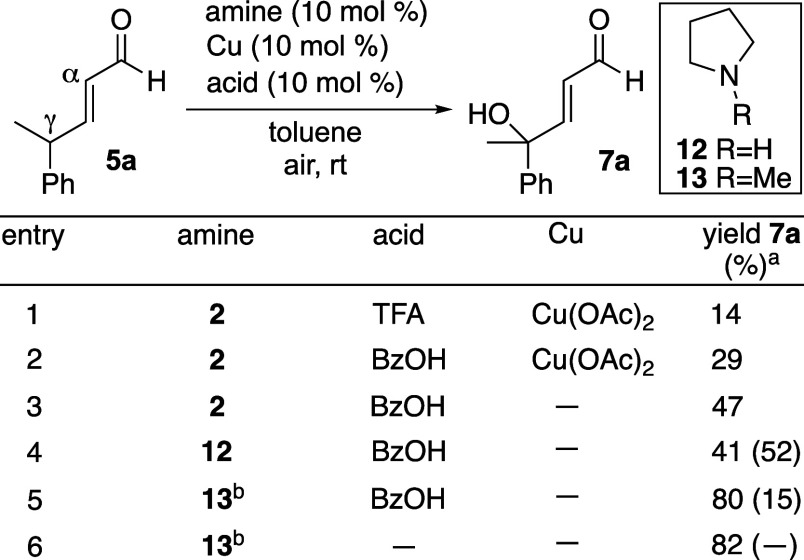
Reaction Optimization

entry	amine	acid	Cu	yield 7a (%)^a^
1	**2**	TFA	Cu(OAc)_2_	14
2	**2**	BzOH	Cu(OAc)_2_	29
3	**2**	BzOH	–	47
4	**12**	BzOH	–	41 (52)
5	**13** ^b^	BzOH	–	80 (15)
6	**13** ^b^	–	–	82 (−)

a ^1^H NMR yield with cyclohexene as the internal standard; ^1^H NMR yield of remaining **5a** in parentheses.

b 1 equiv of **13** used.

In entry 4, as with most substituted benzoic acids evaluated (Table S1), full conversion of the starting material was not achieved, and combined yields of the product and recovered starting material accounted for nearly all of the mass balance (i.e., 75–97%). While we postulated that the reaction proceeded via a dienamine intermediate by a mechanism analogous to that operative in eq 2 in Scheme 1, our conditions lacked an obvious reductant (e.g., PPh_3_). Thus, in this stage, it was envisioned that elucidation of these mechanistic details would enable improved reaction conversions.

To this end, since the combined yields of the product and recovered starting material accounted for nearly all of the mass balance in reactions, these could be excluded as reductants. Next, the presumed hydroperoxide intermediate **6a-H** (Scheme 3A), was independently synthesized.^11^ Neither catalytic BzOH nor H_2_O, which is generated through dienamine formation, reduced **6a-H** to alcohol **7a**. Catalytic 2° amines **2** and **12** provided **7a** from **6a-H** in 28% and 25% yield, respectively (see Table S4 for full details). In addition to **7a**, exposure of **6a-H** to 2° amines generated byproducts identified as push–pull enamine **16** (Scheme 3B) and acetophenone (**17a**). These byproducts are proposed to form from zwitterionic iminium **14** by intramolecular conjugate addition and subsequent oxidative C–C bond cleavage.^6^ Byproducts **16** and **17a** were also observed in reactions in entries 1–4 in Table 1, and neither byproduct converted **6a-H** into **7a**. Structurally related 3° amine *N*-methylpyrrolidine (**13**) reduced **6a-H** to alcohol **7a** in 34% yield. Finally, the addition of catalytic quantities of preformed dienamines **18a** (Scheme 3C) and **18b** (Ar = 4-FC_6_H_4_) provided **7a** from **6a-H** in 45% and 44% yields, respectively (Scheme 3A).

**Scheme 3:**
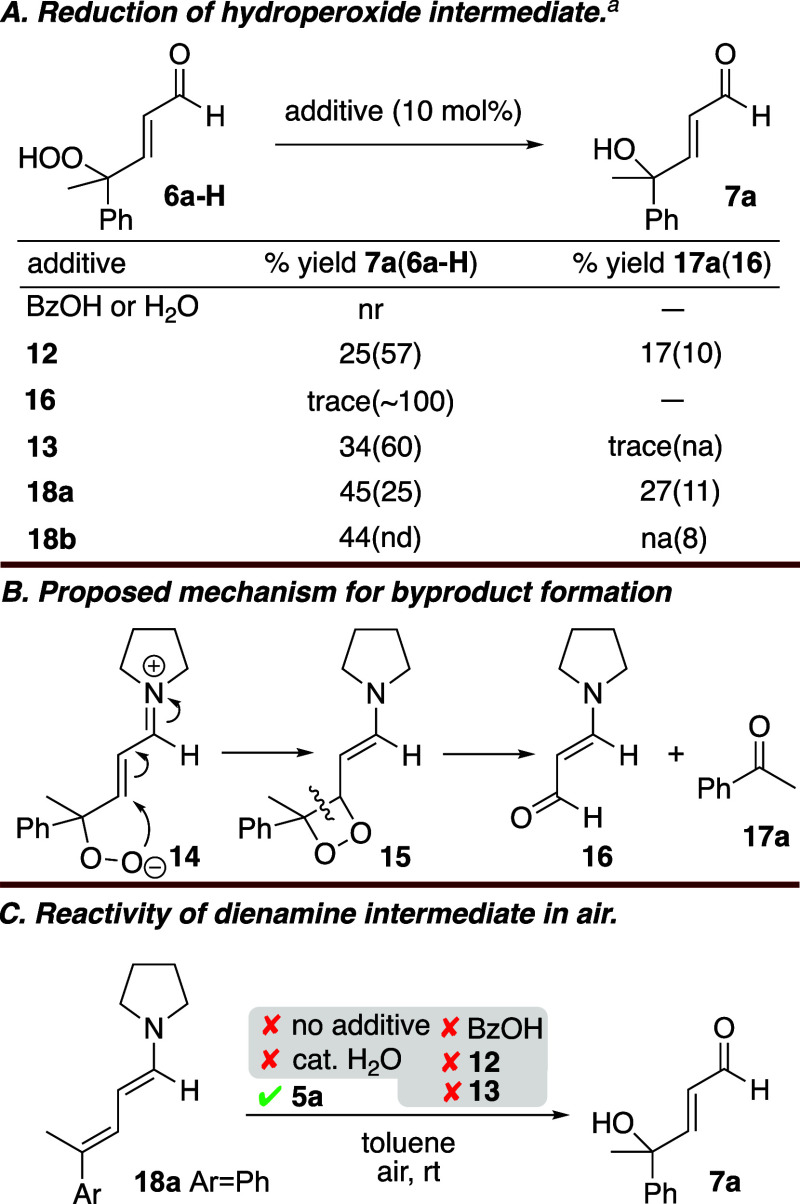
Control Experiments

a
^1^H NMR yield with cyclohexene as the internal standard; nr = no reaction; na = not applicable; nd = not determined.

Concurrent with these investigations, the reaction of dienamine **18a** under air was examined (Scheme 3C). Dienamine **18a** was generated in situ from **5a** and stoichiometric pyrrolidine, both with and without BzOH. Dienamine **18a** did react in air but formed only push–pull enamine **16** and acetophenone **17a**. Presumably, the direct product of the reaction of **18a** with O_2_ is zwitterionic iminium **14**, which proceeded to **16** and **17a** as described above. In the event that this pathway was favored only in the absence of a hydroperoxide reducing agent, various additives that could conceivably act in this capacity were added. These included various stoichiometries of H_2_O, **12**, and **13** and combinations thereof. In all cases, only **16** and **17a** were observed. The only time that formation of **7a** was observed was upon the addition of the starting material **5a** (0.5 equiv) to dienamine **18a**.

The fact that dienamine **18a** was not observed to convert to **7a**, rather it produced **16** and **17a**, which were also observed in reactions in entries 1–4 in Table 1, suggested that the formation of **7a** did not proceed via a dienamine intermediate but that transformation of this intermediate was a competing pathway.^9^ Instead, **5a** likely reacts in air to provide **7a** via a dienolate (or dienol) intermediate (Scheme S1). Although these pathways are separate, there may be some interaction between the two. The dienamine intermediate may be promoting dienolate formation from **5a** and/or reducing the proposed hydroperoxide intermediate **6a-H**. This supposition is corroborated by the observation of **7a** only upon addition of the starting material **5a** to preformed dienamine **16a** (Scheme 3C) and by the formation of **7a** from hydroperoxide **6a-H** upon the addition of catalytic dienamines **16a** and **16b** (Scheme 3A).

Thus, it was realized that use of stoichiometric 3° amine could promote the productive pathway by activating **5a** toward dienolate formation while also serving as a stoichiometric reductant and circumventing the competing dienamine-mediated pathway (Scheme S2). Indeed, exposure of **5a** to stoichiometric **13** in toluene, open to air, for 24 h generated **7a** in 82% ^1^H NMR yield (Table 1, entry 6). HRMS analysis of the crude reaction mixture detected the *N*-oxide of **13** ([M + H] Calcd for [C_5_H_12_NO^+^] 102.0913; found 102.0910), in support of this mechanistic proposal.

Exhaustive reoptimization was not required (Table 1, entries 5–6), and the substrate scope of this transformation was examined under these conditions (Scheme 4). Enal substrates containing phenyl R^1^ groups with electron-neutral, electron-rich, or weakly electron-withdrawing substituents at the *para* or *meta* positions provided alcohol products (**7a**–**d**, **7h**–**i**) in moderate to good yields, and the reaction was scalable.^9^ Enals with strongly electron-withdrawing substituents on the phenyl R^1^ group were considerably more reactive, leading to a reduction in both reaction times and product yields (**7e**-**7g**). *Ortho*-substitution was not tolerated, as evidenced by, e.g., the unreactivity of a substrate with a 1-naphthyl R^1^ group.^9^ A product with a 2-naphthyl group (**7j**), however, was generated smoothly. Heteroaromatic R^1^ groups could also be tolerated (**7k**). Nonaromatic enals with acidic γ-positions were either significantly less reactive (i.e., R^1^ = Bn, R^2^ = CF_3_)^9^ or generated alcohol products (**7l**) with concomitant alkene isomerization (i.e., of the δ,ε-unsaturation in **5l**). In the latter case, the overoxidation product **19** accompanied the secondary alcohol **7l**. Nonetheless, the formation of **7l** affirms the suitability of this mild method for the late-stage remote hydroxylation of medicinal aldehydes. Also pertinent to this point is the chemoselectivity of this method; an enone substrate did not undergo hydroxylation, although partial olefin isomerization (i.e., into β,γ-unsaturation) was observed under these conditions.^9^

**Scheme 4:**
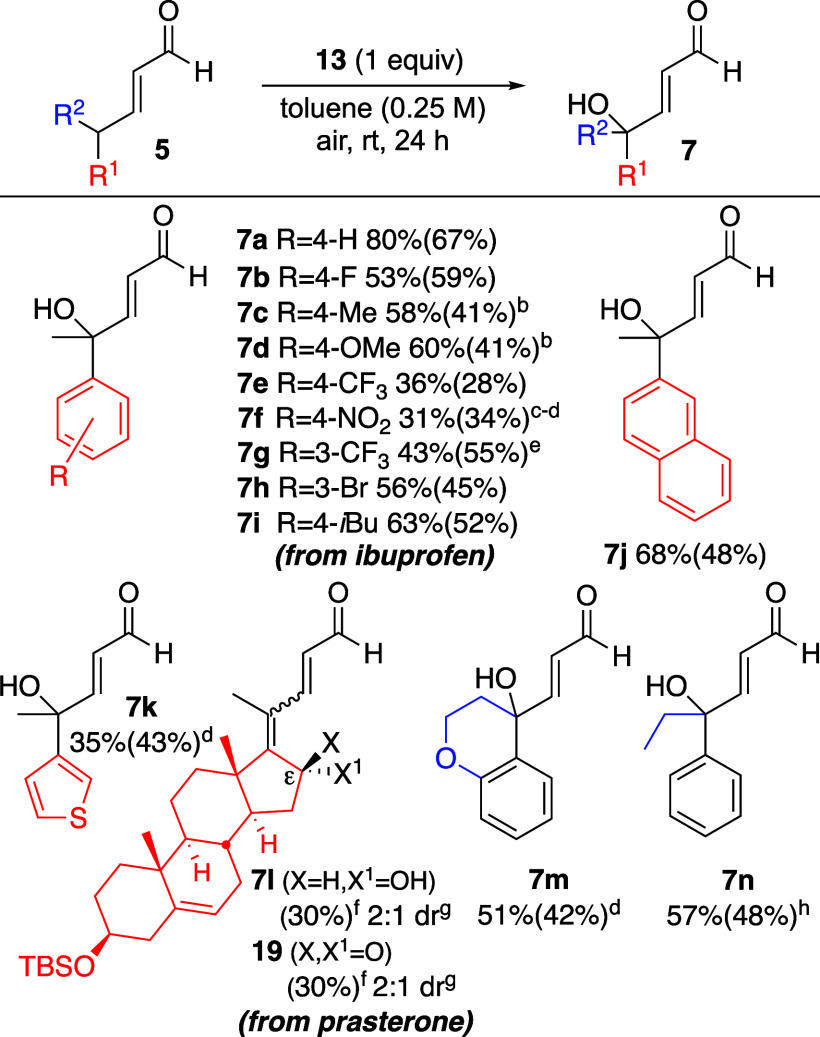
Substrate Scope[Fn s4fn1]–^h^

a
^1^H NMR yield with cyclohexene as the internal standard; isolated yield in parentheses.
b 2 d.
c 2 h.
d 0 °C.
e 3.5 h.
f DABCO instead of **18**, at 45 °C.
g dr arises from *E*/*Z* γ,δ-alkene isomers.
h 4 d.

Next, R^2^ groups other than Me were examined. Comparable reactivity was observed when the γ-carbon was part of a ring system (**7m**) and when R^2^ = Et (**7n**), although a larger R^2^ group (i.e., Cy) resulted in no reaction (data not shown). For limitations of this method, see Figure S1 in the Supporting Information

Finally, although Cu is not required for reactivity, it is required for enantioselectivity in this transformation. As mentioned, racemic products were obtained when the chiral amine catalyst **2** was employed in the absence of Cu. In the presence of Cu, however, and using 2-nitrobenzoic acid (2-NBA) as the acid additive, tertiary alcohols **7a** and **7c** were obtained in low yield but in very high ee under unoptimized conditions (Scheme 5). Notably, this is the first example of catalytic enantioselective γ-oxygenation of enals.

**Scheme 5:**
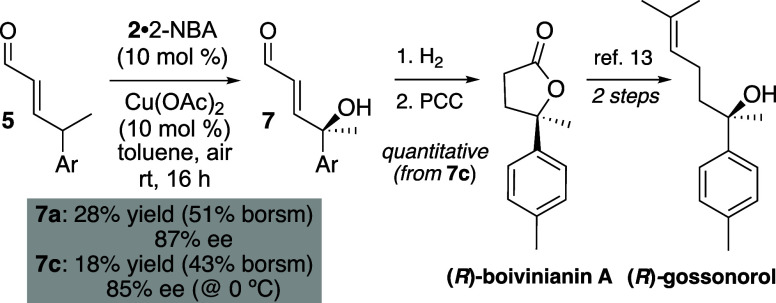
Catalytic Asymmetric Example and Natural Product Synthesis

Furthermore, the enantioenriched product **7c** was transformed in two steps and in quantitative yield into the natural product (*R*)-boivinianin A.^12^ Comparison of its optical rotation with that reported in the literature established the configuration of **7c** and, by analogy, **7a**.^9^ This also completed a formal synthesis of the 3° alcohol-containing natural product (*R*)-gossonorol,^13^ which presumably could be accessed directly from **7c** in two steps.

In conclusion, a chemoselective method for metal-free direct γ-hydroxylation of α,β-unsaturated aldehydes in air has been developed. Reaction products are 3° alcohols generated in up to 80% yield. Mechanistic studies were critical to enabling efficiency in this process. The potential utility of this method for the remote hydroxylation of medicinal aldehydes was demonstrated. Also disclosed herein is the first example of catalytic enantioselective γ-oxygenation of enals, which provided synthetically useful enantioenriched 3° alcohols and is the subject of ongoing investigations in our laboratory.

## Supplementary Material



## Data Availability

The data underlying this study are available in the published article and its Supporting Information.

## Notes

Further details of reaction optimization and scope, control experiments, and proposed mechanism; preparation of substrates and the natural product; characterization data; and copies of ^1^H, ^13^C­{^1^H}, and ^19^F spectra and HPLC chromatograms (PDF)

## Notes

Dedicated to Professor Steven V. Ley CBE FMedSci FRS on the occasion of his 80th birthday.
