# Supplementary material for: Mild Direct Remote Hydroxylation of Enals in Air
Source: J Org Chem. 2026 Mar 11;91(12):4516–20. doi: 10.1021/acs.joc.6c00095 (PMC13036763; doi:10.1021/acs.joc.6c00095)
Supplement: Supplementary file 1 [file jo6c00095_si_001.pdf]

Supporting Information for Mild Direct Remote Hydroxylation of Enals in Air

Fernanda Liu, Alessandro Vicidomini, Stacey E. Brenner-Moyer*

Department of Chemistry, Rutgers University-Newark, 73 Warren Street, Newark, New Jersey 07102

Table of Contents

I. General Methods S3

II. Reaction Optimizations S3

III. Control Experiments and Proposed Mechanism S5

IV. Other Substrates...S7

V. Preparation of substrates and characterization data..S7

VI. γ-Hydroxylation of enals S22

VII. Characterization of remotely hydroxylated-α,β-unsaturated aldehydes S23

VIII. Preparation of products 9 and 10 ....S29

IX. Asymmetric γ-Hydroxylation of aldehydes S35

X. Spectroscopic data (NMR Spectra) S37

XI. Spectroscopic data (HPLC) S109

XII. References S113

I. General Methods: All NMR data were acquired on a Bruker Avance III HD 500/600 MHz NMR spectrometer and processed via TopSpin 4.1.4 software or MestReNova. All column chromatography was carried out with F60, 40–63 nm, 60 Å silica gel and EMD silica 60 F_{254} glass TLC plates. Solvents were dried and kept air free in a solvent purification unit and were evaporated using a standard rotovapor and high vacuum. Chemicals were used as store bought unless specified otherwise. All reactions were performed in oven-dried glassware and under Ar atmosphere unless otherwise stated. Reactions involving lower temperatures were cooled 0 °C under an ice bath/cold room or to -78 °C using a dry ice/acetone bath. Reactions carried out at rt were performed at 20–25 °C. Reactions involving higher temperatures were heated in an oil bath using a Corning Laboratory stirrer/heat plate. HPLC samples and spectra were analyzed using a Flexar HPLC system with a Totalchrom processing software. All mass spectrometry samples and HRMS spectra were acquired via high resolution electrospray (ESI) technique with Q-TOF mass analyzer. The specific rotation ($[\alpha]^{x}_{D}$) was determined for all characterized chiral compounds using a JASCO P-2000 digital polarimeter. Melting point of all solids was determined using an Electrothermal Mel-Temp Melting Point Apparatus. All the chiral catalysts were purchased from Sigma Aldrich.

II. Reaction Optimizations

Table S1. Substituted Benzoic Acids and Benzoic Acid Loading.[a]

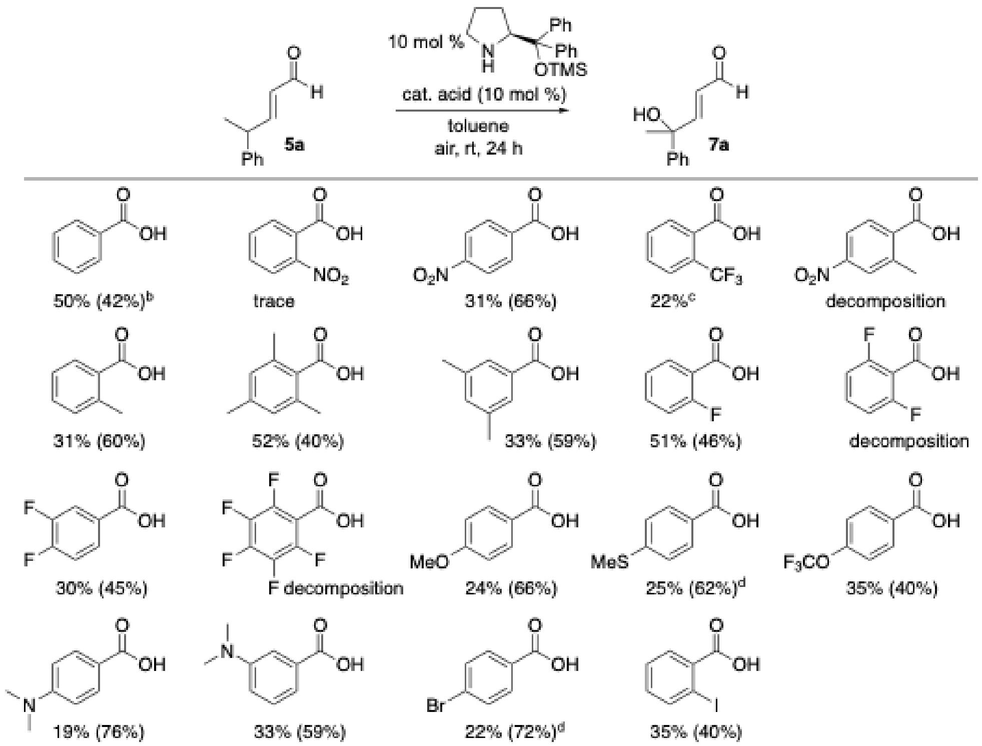

[a] ^{1}H NMR yield of **7a** using cyclohexene as internal standard; ^{1}H NMR yield of remaining **5a** in parentheses. [b] Similar results with 20 mol % acid. [c] Isolated yield. [d] 2 days.

Table S2. Solvent Screen.

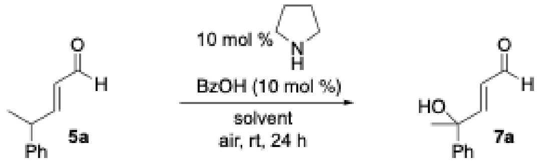

solvent	yield **7a** (%)[a]	yield **5a** (%)[a]
toluene	41	52
o-xylene	36	59
m-xylene	32	56
p-xylene	41	51
EtOH	—	>98
TFE	—	>98
THF	—	>98
Et_2O[b–c]	—	—
EtOAc	15	84
MeCN	trace	>90
DMSO-D_6	—	>98
$CHCl_3$	20	71
DCM	22	62
hexane[b]	—	—

[a] ^{1}H NMR yield using cyclohexene as internal standard. [b] Decomposition. [c] Solvent evaporated.

III. Control Experiments and Proposed Mechanism

Table S3. Control Experiments.

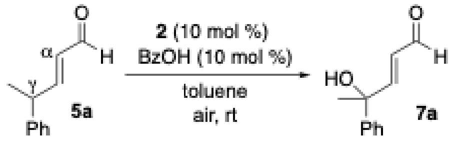

entry	deviation from conditions	yield **7a** (%)[a]
1	none	47
2	in the dark with BHT	55
3	using distilled toluene	50
4	BzOH was recrystallized and washed with HCl	50

[a] ^{1}H NMR yield with cyclohexene as internal standard.

To exclude the possibility that the observed reactivity in the absence of Cu (entry 1, Table S3) arose from an adventitious radical species, the reaction was run in the dark and in the presence of the radical inhibitor BHT (butylated hydroxytoluene). To exclude the possibility that a trace metal(s) was catalyzing this transformation in the absence of Cu, the reaction solvent was distilled (entry 3), and BzOH was recrystallized and washed with HCl prior to use (entry 4). Additionally, as seen above in Table S1, a variety of substituted benzoic acids enabled this transformation in the absence of Cu, further corroborating that the observed reactivity was not attributable to a trace impurity associated with BzOH.

Table S4. Reduction of Hydroperoxide Intermediate.

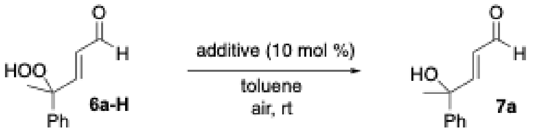

entry	additive	yield **7a** (%)[a-b]	yield **17a** (%)[a,c]
1	**12**, BzOH	33 (—)	19 (nd)
2	BzOH	nr	—
3	**12**	25 (57)	17 (10)
4	**2**	28 (47)	25 (nd)
5	H_2O	nr	—
6	**16**	trace	—
7	**17a**	— (88)	14 (—)
8	**13**	34 (60)	trace
9[d]	**18a**	44 (39)	16 (nd)
10	**18a**	45 (25)	27 (11)
11	**18b**	44[e](nd)	na (8)

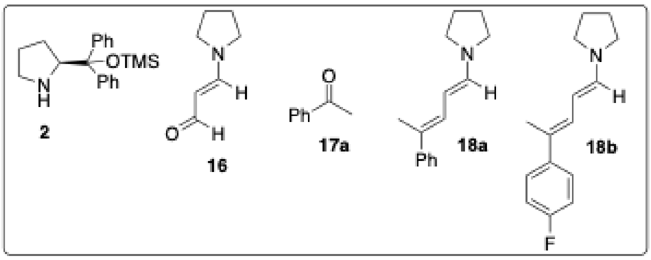

[a] ^{1}H NMR yield using cyclohexene as internal standard. [b] ^{1}H NMR yield of remaining **6a-H** in parentheses. [c] Yield of push-pull enamine **16** in parentheses. [d] BzOH (10 mol %) used in the preformation of **18a**. [e] Isolated yield of **7a**=42%. nd=not determined; nr=no reaction; na=not applicable.

Scheme S1. Revised Mechanism for γ-Hydroxylation of an Unsaturated Aldehyde w/2º Amine.

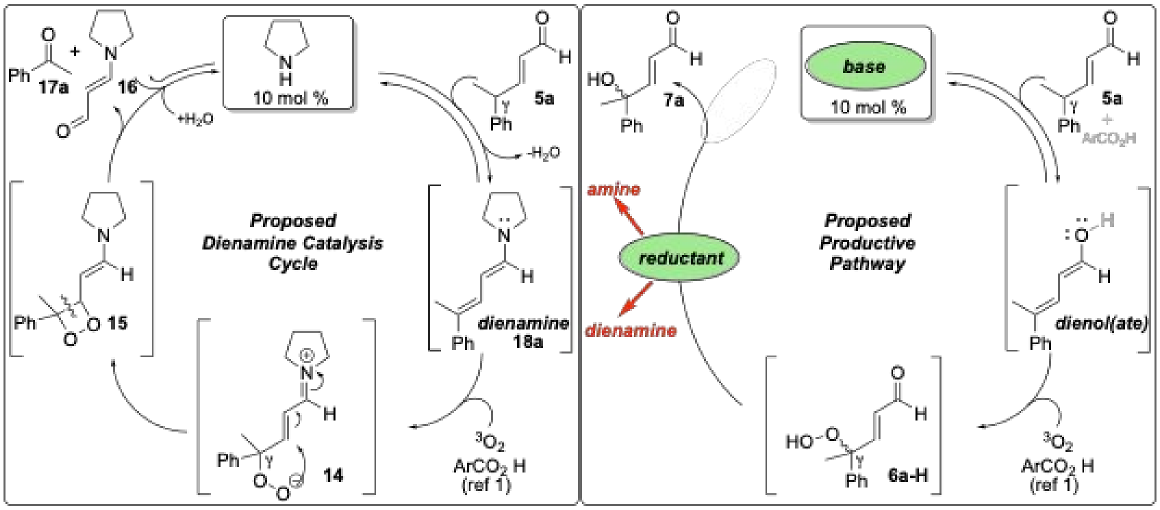

Scheme S2. Proposed Mechanism for γ-Hydroxylation of an Unsaturated Aldehyde w/3º Amine.

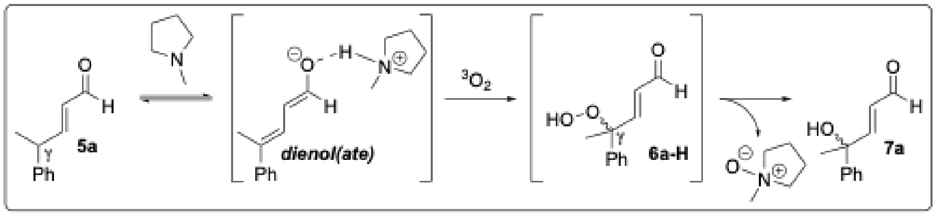

The intermediate **dienol(ate)** could form via γ-deprotonation of **5a**, or via conjugate addition of the 3º amine to **5a**, followed by γ-deprotonation. α-Protonation of the **dienol(ate)**, or of the conjugate addition product (i.e., of 3º amine to **5a**) prior to γ-deprotonation, would result in double bond isomerization of **5a** to the corresponding β,γ-unsaturated aldehyde.

IV. Other Substrates

Figure S1. Other Substrates.[a]

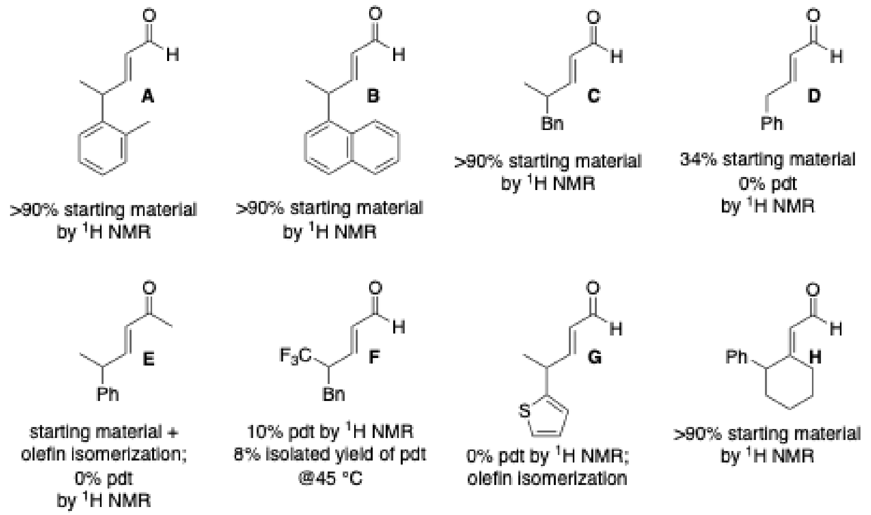

[a] Cyclohexene used as internal standard.

V. Preparation of substrates and characterization data

Synthesis of enals

Compounds **C**, **D**, **E** and **F** (Figure S1) were prepared according to literature procedures[2,2,3,4] and ^{1}H NMR and $^{13}C\{^{1}H\}$ NMR spectra match those reported in the respective literature precedents.

Procedure A:

Preparation of 5a-h, k-n

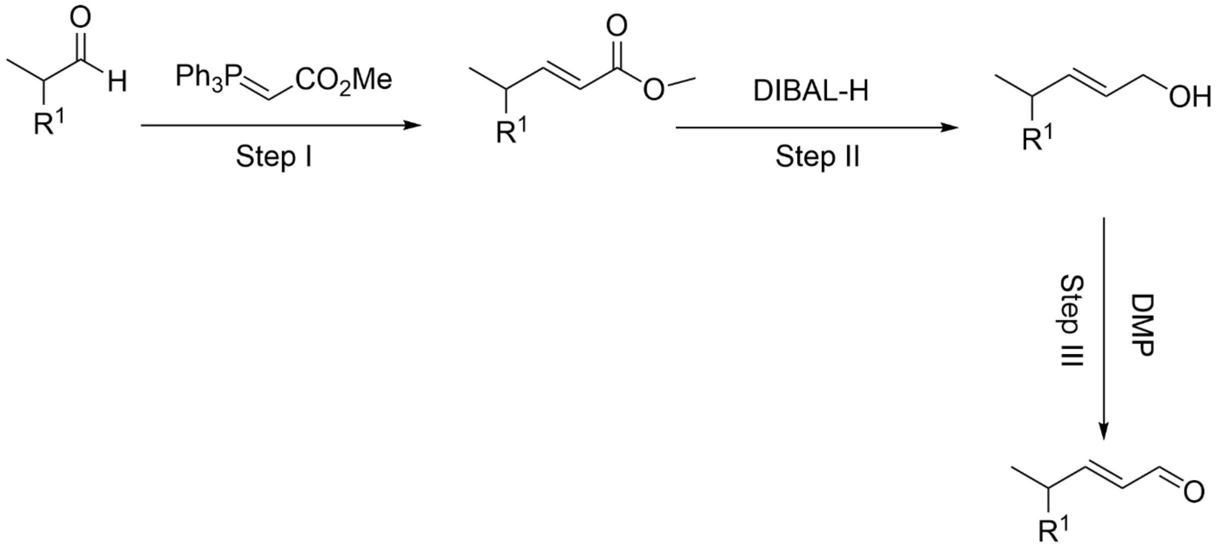

For commercially available α-methyl aldehydes:

Step I:[2] To a stirred solution of the aldehyde (1.0 equiv.) in anhydrous DCM (2.0 M), cooled to 0 ˚C, methyl(triphenylphosphoranylidene)acetate (1.1 equiv.) was added portion-wise. The reaction mixture was stirred at rt until full consumption of the starting material was observed by TLC (24-72 h). The solvent was removed under low pressure and the residue was purified by flash chromatography (EtOAc/P.E., 1%) to afford the ester.

Step II: Reduction: To a stirred solution of the ester (1.0 equiv.) in anhydrous DCM (0.2 M), cooled to -78 ˚C, DIBAL-H (2.5 equiv., 1.0 M in hexane) was added dropwise under Ar. After stirring at -78 ˚C for 1 h, the reaction mixture was quenched with MeOH and 1M NaOH. Then, the remaining mixture was warmed up to rt and stirred until the layers were clear. The aqueous phase was then extracted with DCM and the combined organic layers were washed with brine and dried over Na_2SO_4. The solvent was removed under low pressure after filtration and the crude product mixture was purified by flash chromatography (EtOAc/P.E., 10%→20%) to afford the alcohol product.

Step III: Dess-Martin oxidation: To a stirred solution of the alcohol (1.0 equiv.) in anhydrous DCM (0.1M), DMP (1.2 equiv.) was added in one portion at rt. After stirring for 30 min, the reaction was quenched by sat. aq. $Na_2S_2O_3$ and $NaHCO_3$ solutions. The remaining mixture was stirred until no more bubbling was observed and the layers became clear. The resulting solution was then extracted with DCM and the combined organic layers were dried over Na_2SO_4. The solvent was removed under low pressure after filtration and the crude product mixture was purified by flash chromatography (EtOAc/P.E., 1%) to afford the α,β-unsaturated aldehyde.

Preparation of non-commercially available α-methyl aldehydes:

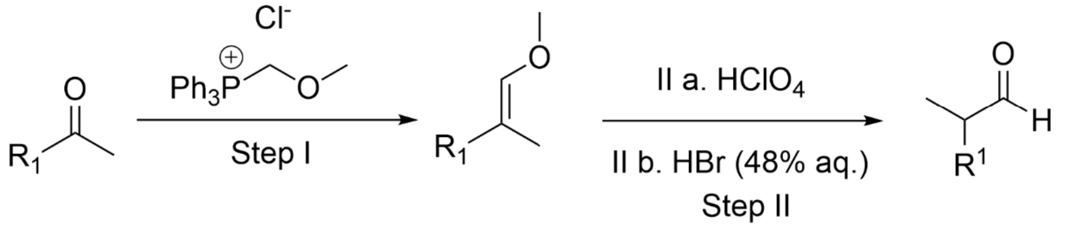

Step I: Wittig reaction:[5] To a stirred solution of methoxymethyl triphenylphosphonium chloride (1.6 equiv.) in Et_2O (0.67 M), at 0 ˚C, *t*-BuOK (1.6 equiv.) was added portion-wise to afford a dark red solution. The mixture was stirred for 30 min at 0 ˚C and a solution of the starting material (1 equiv.) in Et_2O (4.0 M) was added dropwise. The reaction mixture was stirred at 0 ˚C and monitored by TLC until completion (20 min - 1.5 h). After completion, the reaction was quenched with H_2O and the aqueous layer was extracted with EtOAc. The combined organic layers were dried over Na_2SO_4. The solvent was removed under low pressure after filtration and the crude product mixture was purified by flash chromatography (EtOAc/P.E., 1%) to afford the enol-ether.

Step II a: Deprotection with $HClO_4$:[5] *For substrates 5b-d, 5f, 5j.* To a stirred solution of the enol-ether (1.0 equiv.) in Et_2O (1.0 M), $HClO_4$ (1.0 equiv., 60%) was added dropwise and the reaction mixture was stirred until completion and monitored by TLC (3-24 h). After completion, the reaction was quenched with slow addition of H_2O and the aqueous layer was extracted with EtOAc. The combined organic layers were dried over Na_2SO_4. The solvent was removed under low pressure after filtration and the crude product mixture was purified by flash chromatography (EtOAc/P.E., 1%) to afford the α-methyl aldehyde.

Step II b: Deprotection with HBr (48%):[6] *For substrates 5e, 5g, 5h, 5k.* The enol-ether was dissolved in a solution of acetone and water (4:1, 0.8 M) and the solution was cooled to 0 ˚C with continuous stirring. Then, HBr (2.3 equiv,. 48% aq.) was added dropwise and the reaction mixture was allowed to stir for 30 min at 0 ˚C, then warmed up to rt. The reaction was stirred for (24-72 h) then quenched carefully with a sat. aq. $NaHCO_3$ solution. The aqueous phase was

extracted with EtOAc. The combined organic layers were washed with brine and dried over Na_2SO_4. The solvent was removed under low pressure after filtration and the residue was purified by flash chromatography (EtOAc/P.E., 2%).

(*E*)-4-phenylpent-2-enal (5a)

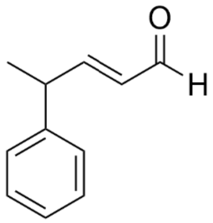

Synthesized using *Procedure A* from commercially available α-methyl aldehyde. Purified by column chromatography (EtOAc/P.E., 1%) and isolated as a colorless oil (1 g, 62% yield over 3 steps). ^{1}H NMR (500 MHz, $CDCl_3$): δ 9.53 (1H, d, *J* = 7.8 Hz), 7.18−7.35 (5H, m), 6.96 (1H, dd, *J* = 15.6, 6.4 Hz), 6.11 (1H, ddd, *J* = 15.6, 7.8, 1.4 Hz), 3.73 (1H, quint, *J* = 6.6 Hz), δ 1.47 (d, *J* = 7.0 Hz, 3H). ^{13}C{^{1}H} NMR (126 MHz, $CDCl_3$): δ 194.1, 161.8, 142.7, 131.3, 128.9, 127.3, 127.1, 42.5, 19.9. HRMS (ESI) *m/z* $[M+H]^+$ calcd. for $[C_{11}H_{12}O]$ 161.0961, found 161.0966.

(*E*)-4-(4-fluorophenyl)pent-2-enal (5b)

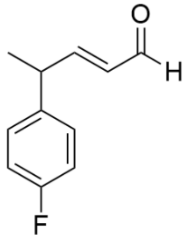

Synthesized using *A*. Purified by column chromatography (EtOAc/P.E., 1%) and isolated as a colorless oil (400 mg, 31% yield after 5 steps). ^{1}H NMR (500 MHz, $CDCl_3$): δ 9.58 (d, *J* = 7.8 Hz, 1H), 7.20 – 7.17 (m, 2H), 7.05 (t, *J* = 8.8 Hz, 2H), 6.97 (dd, *J* = 15.7, 6.2 Hz, 1H), 6.12 (dd, *J* = 15.8, 7.8 Hz, 1H), 3.79 (p, *J* = 7.0 Hz, 1H), 1.50 (d, *J* = 7.0 Hz, 3H). ^{19}F NMR (471 MHz, $CDCl_3$) δ -115.7. ^{13}C{^{1}H} NMR (126 MHz, $CDCl_3$): δ 193.9, 161.2, 131.3, 128.8, 128.8, 115.8, 115.6, 41.7, 20.1. HRMS (ESI) *m/z* $[M+H]^+$ calcd. for $[C_{11}H_{12}OF]$ 179.0867 found 179.0875.

(*E*)-4-(p-tolyl)pent-2-enal (5c)

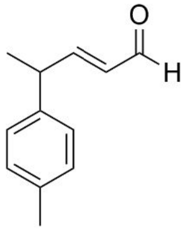

Synthesized using *Procedure A*. Purified by column chromatography (EtOAc/P.E., 1%) and isolated as a colorless oil (700 mg, 35% yield after 5 steps). ^{1}H NMR (500 MHz, $CDCl_3$): δ 9.51 (d, *J* = 7.8 Hz, 1H), 7.14 (d, *J* = 8.5 Hz, 2H), 7.08 (d, *J* = 8.2 Hz, 2H), 6.93 (dd, *J* = 15.6, 6.5 Hz, 1H), 6.09 (ddd, *J* = 15.6, 7.8, 1.5 Hz, 1H), 3.69 (pd, *J* = 7.0, 1.5 Hz, 1H), 2.32 (s, 3H), 1.45 (d, *J* = 7.0 Hz, 4H). ^{13}C{^{1}H} NMR (126 MHz, $CDCl_3$): δ 194.2, 162.2, 139.8, 136.9, 131.2, 129.7, 127.3, 42.3, 21.2, 20.1. HRMS (ESI) *m/z* $[M+H]^+$ calcd. for $[C_{12}H_{15}O]$ 175.1117, found 175.1126.

(*E*)-4-(4-methoxyphenyl)pent-2-enal (5d)

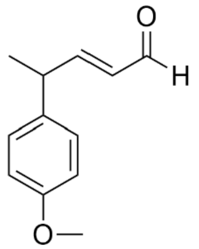

Synthesized using *Procedure A.* Purified by column chromatography (EtOAc/P.E., 1%) and isolated as a colorless oil (800 mg, 40% yield after 5 steps). ^{1}H NMR (500 MHz, $CDCl_3$): δ 9.51 (d, *J* = 7.8 Hz, 1H), 7.10 (d, *J* = 8.7 Hz, 2H), 6.92 (ddd, *J* = 15.6, 6.4 Hz, 1H), 6.86 (d, *J* = 8.6 Hz, 2H), 6.08 (ddd, *J* = 15.7, 7.8, 1.5 Hz, 1H), 3.78 (s, 3H), 3.74 – 3.56 (m, 1H), 1.43 (d, *J* = 7.0 Hz, 3H). ^{13}C{^{1}H} NMR (126 MHz, $CDCl_3$): δ 193.9, 161.9, 158.4, 134.4, 130.7, 128.1, 110.4, 55.1, 41.5, 19.7. HRMS (ESI) *m/z* $[M+H]^+$ calcd. for $[C_{12}H_{15}O_2]$ 191.1066, found 191.1063.

(*E*)-4-(4-(trifluoromethyl)phenyl)pent-2-enal (5e)

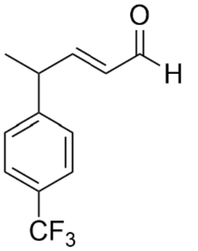

Synthesized using *Procedure A.* Purified by column chromatography (EtOAc/P.E., 1%) and isolated as a colorless oil (40 mg, 10% yield after 5 steps). ^{1}H NMR (500 MHz, $CDCl_3$): δ 9.58 (d, *J* = 7.7 Hz, 1H), 7.63 (d, *J* = 8.0 Hz, 2H), 7.35 (d, *J* = 8.0 Hz, 2H), 6.96 (dd, *J* = 15.7, 6.3 Hz, 1H), 6.14 (ddd, *J* = 15.8, 7.8, 1.5 Hz, 1H), 3.85 (p, *J* = 6.9 Hz, 1H), 1.53 (d, *J* = 7.0 Hz, 3H). ^{19}F NMR (471 MHz, $CDCl_3$) δ -62.5. ^{13}C{^{1}H} NMR (126 MHz, $CDCl_3$): δ 193.6, 160.0, 146.7, 131.8, 127.7, 125.9, 125.9, 125.9, 125.8, 42.3, 23.8, 19.9. HRMS (ESI) *m/z* $[M+H]^+$ calcd. for $[C_{12}H_{12}OF_3]$ 229.0835, found 229.0832.

(*E*)-4-(4-nitrophenyl)pent-2-enal (5f)

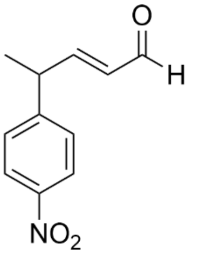

Synthesized using *Procedure A.* Purified by column chromatography (EtOAc/P.E., 1%→20%) and isolated as a light red oil (25 mg, 5% yield after 5 steps). ^{1}H NMR (500 MHz, $CDCl_3$): δ 9.55 (d, *J* = 7.7 Hz, 1H), 8.19 (d, *J* = 8.7 Hz, 2H), 7.37 (d, *J* = 8.7 Hz, 2H), 6.91 (dd, *J* = 15.7, 6.3 Hz, 1H), 6.11 (ddd, *J* = 15.7, 7.7, 1.5 Hz, 1H), 3.86 (pd, *J* = 7.0, 1.6 Hz, 1H), 1.51 (d, *J* = 7.1 Hz, 3H). ^{13}C{^{1}H} NMR (126 MHz, $CDCl_3$): δ 193.7, 159.2, 150.3, 147.2, 132.3, 128.5, 124.4, 42.4, 20.1. HRMS (ESI) *m/z* $[M+H]^+$ calcd. for $[C_{11}H_{12}NO_3]$ 206.0812, found 206.0815.

(*E*)-4-(3-(trifluoromethyl)phenyl)pent-2-enal (5g)

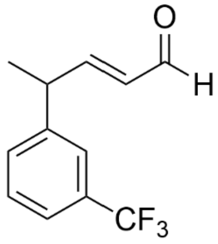

Synthesized using *Procedure A.* Purified by column chromatography (EtOAc/P.E., 1%) and isolated as a colorless oil (50 mg, 16% yield after 5 steps). ^{1}H NMR (500 MHz, $CDCl_3$): δ 9.54 (d, *J* = 7.7 Hz, 1H), 7.52 (d, *J* = 7.9 Hz, 1H), 7.48 – 7.42 (m, 2H), 7.38 (d, *J* = 7.7 Hz, 1H), 6.92 (dd, *J* = 15.7, 6.4 Hz, 1H), 6.10 (ddd, *J* = 15.7, 7.7, 1.5 Hz, 1H), 3.80 (tt, *J* = 7.1, 5.9 Hz, 1H), 1.50 (d, *J* = 7.1 Hz, 3H). ^{19}F NMR (471 MHz, $CDCl_3$): δ -62.60. ^{13}C{^{1}H} NMR (126 MHz, $CDCl_3$): δ 193.7, 160.1, 143.6, 131.8, 130.8, 129.4, 124.1, 124.1, 124.1, 124.0, 123.9, 42.3, 19.9. HRMS (ESI) *m/z* $[M+H]^+$ calcd. for $[C_{12}H_{12}F_3O]$, 229.0835 found 229.0832.

(*E*)-4-(3-bromophenyl)pent-2-enal (5h)

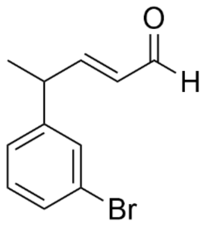

Synthesized using *Procedure A.* Purified by column chromatography (EtOAc/P.E., 1%) and isolated as a colorless oil (50 mg, 5% yield after 5 steps). ^{1}H NMR (500 MHz, $CDCl_3$): δ 9.53 (d, *J* = 7.7 Hz, 1H), 7.40 – 7.36 (m, 1H), 7.33 (d, *J* = 2.2 Hz, 1H), 7.20 (t, *J* = 7.8 Hz, 1H), 7.11 (d, *J* = 8.0 Hz, 1H), 6.90 (dd, *J* = 15.8, 6.4 Hz, 1H), 6.09 (ddd, *J* = 15.7, 7.8, 1.6 Hz, 1H), 3.70 (p, *J* = 6.9 Hz, 1H), 1.45 (d, *J* = 7.0 Hz, 3H). ^{13}C{^{1}H} NMR (126 MHz, $CDCl_3$): δ 194.1, 160.68, 145.1, 131.8, 130.6, 130.4, 126.2, 123.1, 42.3, 20.0. HRMS (ESI) *m/z* $[M+H]^+$ calcd. for $[C_{11}H_{12}OBr]$ 239.0066, 241.0046, found 239.0063, 241.0043.

(*E*)-4-(naphthalen-2-yl)pent-2-enal (5j)

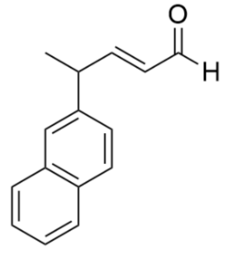

Synthesized using *Procedure A.* Purified by column chromatography (EtOAc/P.E., 1%) and isolated as a light yellow oil (100 mg, 15% yield after 5 steps). ^{1}H NMR (500 MHz, $CDCl_3$): δ 9.55 (d, *J* = 7.8 Hz, 1H), 7.83 – 7.77 (m, 3H), 7.63 (s, 1H), 7.50 – 7.42 (m, 2H), 7.31 (dd, *J* = 8.5, 1.8 Hz, 1H), 7.03 (dd, *J* = 15.6, 6.4 Hz, 1H), 6.16 (ddd, *J* = 15.7, 7.8, 1.5 Hz, 1H), 3.94 – 3.87 (m, 1H), 1.56 (d, *J* = 7.0 Hz, 4H). ^{13}C{^{1}H} NMR (126 MHz, $CDCl_3$): δ 193.9, 161.45, 139.9, 133.4, 132.4, 131.3, 128.5, 127.5, 126.2, 125.8, 125.6, 42.5, 19.8. HRMS (ESI) *m/z* $[M+H]^+$ calcd. for $[C_{15}H_{15}O]$ 211.1174, found 211.1172.

(*E*)-4-(thiophen-3-yl)pent-2-enal (5k)

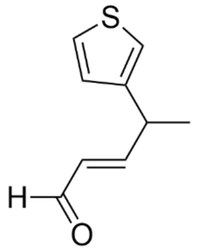

Synthesized using *Procedure A*. Purified by column chromatography (EtOAc/P.E., 1%) and isolated as a light yellow oil (37 mg, 5% yield after 5 steps). ^{1}H NMR (500 MHz, $CDCl_3$): δ 9.53 (d, *J* = 7.8 Hz, 1H), 7.30 (dd, *J* = 5.0, 2.9 Hz, 1H), 7.02 (dt, *J* = 2.9, 1.1 Hz, 1H), 6.94 – 6.87 (m, 2H), 6.09 (ddd, *J* = 15.6, 7.8, 1.3 Hz, 1H), 3.88 – 3.80 (m, 1H), 1.47 (d, *J* = 7.0 Hz, 3H). $^{13}C\{^{1}H\}$ NMR (126 MHz, $CDCl_3$): δ 194.1, 161.2, 143.0, 131.2, 126.8, 126.3, 120.6, 38.0, 19.7. HRMS (ESI) *m/z* $[M+H]^+$ calcd. for $[C_9H_{11}O^{32}S]$ 167.0525, found 167.0523.

(*E*)-4-phenylhex-2-enal (5n)

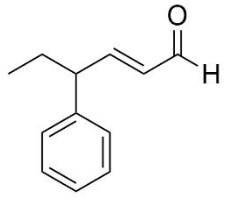

Synthesized using *Procedure A* from corresponding commercially available α-ethyl aldehyde. Purified by column chromatography (EtOAc/P.E., 1%) and isolated as a colorless oil (400 mg, 50% yield over 3 steps). ^{1}H NMR (500 MHz, $CDCl_3$): δ 9.55 (d, *J* = 7.9 Hz, 1H), 7.38-7.20 (m, 5H), 6.96 (dd, *J* = 15.7, 7.4 Hz, 1H), 6.13 (dd, *J* = 15.7, 7.9 Hz, 1H), 3.46 (m, 1H), 1.96 – 1.82 (m, 2H), 0.93 (t, *J* = 7.3 Hz, 3H). $^{13}C\{^{1}H\}$ NMR (126 MHz, $CDCl_3$): δ 194.1, 161.0, 141.4, 131.9, 128.8, 127.8, 127.1, 50.6, 27.6, 12.1. HRMS (ESI) *m/z* $[M+H]^+$ calcd. for $[C_{12}H_{15}O]$ 175.1117, found 175.1125.

(*E*)-4-(o-tolyl)pent-2-enal (A, Figure S1)

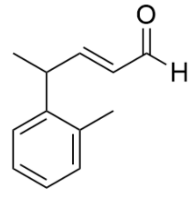

Synthesized using *Procedure A*. Purified by column chromatography (EtOAc/P.E., 1%) and isolated as a colorless oil (550 mg, 40% yield after 5 steps). ^{1}H NMR (500 MHz, $CDCl_3$): δ 9.53 (d, *J* = 7.8 Hz, 1H), 7.22 – 7.10 (m, 4H), 6.94 (dd, *J* = 15.7, 5.9 Hz, 1H), 6.08 (ddd, *J* = 15.7, 7.9, 1.6 Hz, 1H), 3.99 – 3.92 (m, 1H), 2.32 (s, 3H), 1.44 (d, *J* = 7.0 Hz, 3H). $^{13}C\{^{1}H\}$ NMR (126 MHz, $CDCl_3$): δ 194.4, 161.9, 140.9, 135.8, 131.5, 130.9, 127.1, 126.8, 126.6, 38.3, 19.7, 19.4. HRMS (ESI) *m/z* $[M+H]^+$ calcd. for $[C_{12}H_{15}O]$ 175.1117, found 175.1124.

(*E*)-4-(naphthalen-1-yl)pent-2-enal (B, Figure S1)

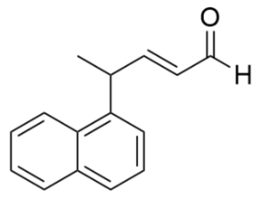

Synthesized using *Procedure A.* Purified by column chromatography (EtOAc/P.E., 1%) and isolated as a light yellow oil (300 mg, 24% yield after 5 steps). ^{1}H NMR (500 MHz, $CDCl_3$): δ 9.59 (d, *J* = 7.8 Hz, 1H), 8.05 (d, *J* = 8.3 Hz, 1H), 7.92 (dd, *J* = 7.8, 1.7 Hz, 1H), 7.82 (d, *J* = 8.2 Hz, 1H), 7.61 – 7.52 (m, 2H), 7.49 (t, *J* = 7.7 Hz, 1H), 7.38 (d, *J* = 7.1 Hz, 1H), 7.15 (dd, *J* = 15.8, 5.7 Hz, 1H), 6.22 (ddd, *J* = 15.8, 7.8, 1.7 Hz, 1H), 4.65 – 4.56 (m, 1H), 1.67 (d, *J* = 7.0 Hz, 3H). ^{13}C{^{1}H} NMR (126 MHz, $CDCl_3$): δ 194.1, 161.8, 138.7, 134.1, 131.8, 131.1, 129.2, 127.8, 126.4, 125.8, 125.6, 124.1, 122.9, 37.5, 19.5. HRMS (ESI) *m/z* $[M+H]^+$ calcd. for $[C_{15}H_{15}O]$ 211.1117, found 211.1122.

(*E*)-4-methyl-5-phenylpent-2-enal (C, Figure S1)

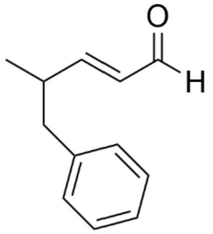

Purified by column chromatography (EtOAc/P.E., 1%) and isolated as a colorless oil (700 mg, 56% yield over 4 steps). ^{1}H NMR (500 MHz, $CDCl_3$): δ 9.51 (d, *J* = 7.8 Hz, 1H), 7.32 (t, *J* = 7.5 Hz, 2H), 7.24 (t, *J* = 7.4 Hz, 1H), 7.17 (d, *J* = 7.4 Hz, 2H), 6.81 (dd, *J* = 15.7, 6.3 Hz, 1H), 6.07 (dd, *J* = 15.7, 7.8 Hz, 1H), 3.02 – 2.46 (m, 3H), 1.15 (d, *J* = 6.0 Hz, 3H). ^{13}C{^{1}H}NMR (126 MHz, $CDCl_3$): δ 194.1, 162.8, 139.06, 131.5, 129.0, 128.4, 126.4, 42.3, 38.6, 18.6. HRMS (ESI) *m/z* $[M+H]^+$ calcd. for $[C_{12}H_{15}O]$ 175.1117, found 175.1125.

(*E*)-4-(thiophen-2-yl)pent-2-enal (G, Figure S1)

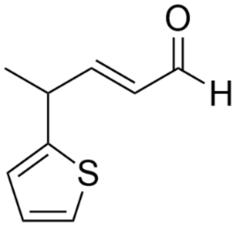

Synthesized using *Procedure A.* Purified by column chromatography (EtOAc/P.E., 1%) and isolated as a light yellow oil (37 mg, 5% yield after 5 steps).^{1}H NMR (500 MHz, $CDCl_3$): δ 9.59 (d, *J* = 7.8 Hz, 1H), 7.24 (dd, *J* = 5.1, 1.2 Hz, 1H), 7.03 – 6.87 (m, 3H), 6.16 (ddd, *J* = 15.6, 7.8, 1.4 Hz, 1H), 4.05 (p, *J* = 7.1 Hz, 1H), 1.58 (d, *J* = 7.1 Hz, 3H). ^{13}C{^{1}H} NMR (126 MHz, $CDCl_3$): δ 193.9, 160.3, 145.9, 131.3, 127.0, 124.2, 124.1, 37.8, 20.9. HRMS (ESI) *m/z* $[M+H]^+$ calcd. for $[C_9H_{11}O^{32}S]$, 167.0525 found 167.0523.

Preparation of 5i:

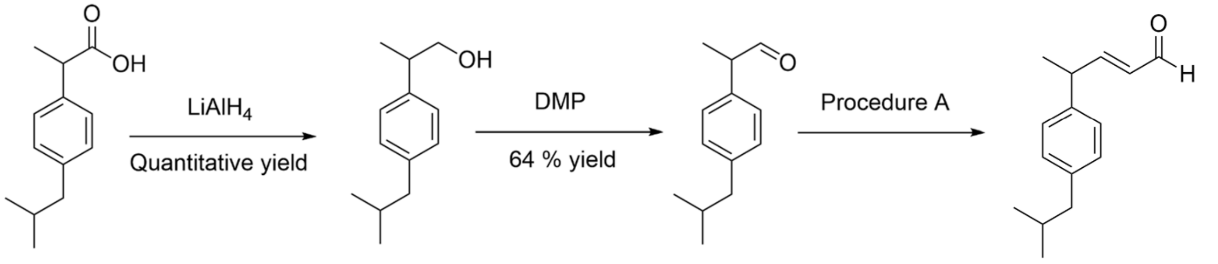

A solution of Ibuprofen in THF (0.7M) was added dropwise to a suspension of $LiAlH_4$ (0.975 equiv.) in THF (2.5M) under Ar at 0 ˚C. The reaction mixture was allowed to reach rt and stirred for 1 h before it was quenched with $Na_2SO_4 \cdot 10H_2O$. The white suspension was filtered through Celite and washed with DCM. The filtrate was concentrated under reduced pressure. The crude alcohol was then taken to the next step.

Dess-Martin oxidation: To a stirred solution of the alcohol (1.0 equiv.) in anhydrous DCM (0.1M), DMP (1.2 equiv.) was added in one portion at rt. After stirring for 30 min, the reaction was quenched by sat. aq. $Na_2S_2O_3$ and $NaHCO_3$ solutions. The remaining mixture was stirred until no more bubbling was observed and the layers became clear. The resulting solution was then extracted with DCM and the combined organic layers were dried over Na_2SO_4. The solvent was removed under low pressure after filtration and the crude product mixture was purified by flash chromatography (EtOAc/P.E., 1%) to afford the aldehyde. After that, the pure product was taken through the steps of Procedure A.

(*E*)-4-(4-isobutylphenyl)pent-2-enal (5i)

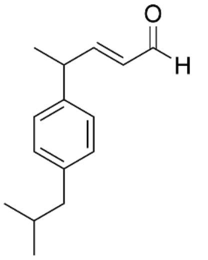

Purified by column chromatography (EtOAc/P.E., 1%→2%) and isolated as a colorless oil (400 mg, 40% yield after 5 steps). ^{1}H NMR (500 MHz, $CDCl_3$): δ 9.56 (d, J = 7.8 Hz, 1H), 7.13 (s, 4H), 6.99 (dd, J = 15.7, 6.4 Hz, 1H), 6.14 (ddd, J = 15.6, 7.9, 1.6 Hz, 1H), 3.74 (p, J = 6.0 Hz, 1H), 2.48 (d, J = 7.1 Hz, 2H), 1.87 (dt, J = 13.6, 6.8 Hz, 1H), 1.49 (d, J = 7.0 Hz, 3H), 0.92 (d, J = 6.6 Hz, 6H). $^{13}C\{^1H\}$NMR (126 MHz, $CDCl_3$): δ 194.1, 162.1, 140.6, 139.8, 131.1, 129.6, 127.0, 45.0, 42.2, 30.2, 22.4, 19.9. HRMS (ESI) *m/z* $[M+H]^+$ calcd. for $[C_{15}H_{21}O]$ 217.1587, found 217.1576.

Preparation of 5l:

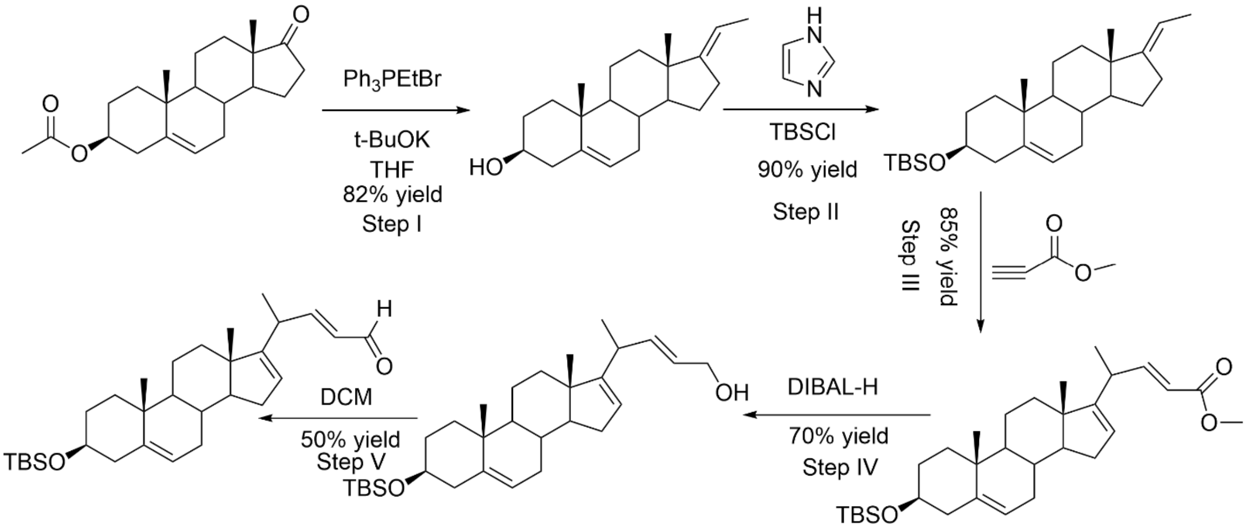

Step I: To a solution of Ph_3PEtBr (10 equiv.), in anhydrous THF (0.22M) was added dropwise a solution of *t*-BuOK (10 equiv.) in anhydrous THF (0.33M) under Ar at 0 ˚C. After stirring for 1.5 h at rt, a solution of starting material (1 equiv.) in THF (0.33M) was added dropwise. After stirring at 70 ˚C for 10 min, the reaction was quenched with sat. aq. NH_4Cl solution (16.5 mL/mmol of starting material) and extracted with EtOAc. The combined organic solution was dried over Na_2SO_4, filtered and concentrated. The residue was purified by flash chromatography (EtOAc/P.E., 1%→20%) to afford the product.

Step II: To an oven-dried round bottom flask equipped with stir bar and septum, cooled under Ar, was added starting material (1 equiv.), THF (1.38M), imidazole (1.47 equiv.) and TBSCl (1.1 equiv.). The reaction was stirred for 2 h and quenched with H_2O and MeOH. The aqueous layer was extracted with Et_2O and the organic extracts were washed with brine, dried over Na_2SO_4, filtered and concentrated. The residue was purified by flash chromatography (*n*-hexane, 100%) to afford the product.

Step III: To a solution of the starting material (1 equiv.) and methyl propiolate (2.5 equiv.) in anhydrous DCM (0.2M) at 0 ˚C was added, dropwise, a solution of Et_2AlCl (4 equiv., 1.0 M in hexane). After warming up to rt and stirring for 3 h, the mixture was quenched with sat. aq. $NaHCO_3$ solution and extracted with DCM. The combined organic solution was dried over Na_2SO_4, filtered and concentrated. The residue was purified by flash chromatography (EtOAc/P.E., 0%→2%) to afford the product.

Step IV: To a solution of the ester (1 equiv.) in anhydrous DCM (0.2 M) was added DIBAL-H (2.5 equiv., 1.0 M in hexane) at 0 ˚C under Ar. The mixture was stirred for 30 min and quenched with MeOH and NaOH (1 M) until the layers became clear. The aqueous layer was extracted with DCM, the organic fraction was washed with brine, dried over Na_2SO_4, filtered

and concentrated. The residue was purified by flash chromatography (EtOAc/P.E., 5%→20%) to afford the product.

Step V: To a solution of the alcohol (1 equiv.) in DCM (0.1 M) was added DMP (1.2 equiv.) in one portion. The mixture was stirred at rt for 10 min and quenched with $Na_2S_2O_3$ and an aq. $NaHCO_3$ solution, stirred until the layers became transparent, and then extracted with DCM . Organic layers were dried over Na_2SO_4, filtered and concentrated. The residue was purified by flash chromatography (EtOAc/P.E., 0%→2%) to afford the product.

(*E*)-4-((3*S*,10*R*,13*S*)-3-((tert-butyldimethylsilyl)oxy)-10,13-dimethyl-2,3,4,7,8,9,10,11,12,13,14,15-dodecahydro-1H-cyclopenta[a]phenanthren-17-yl)pent-2-enal (5l)

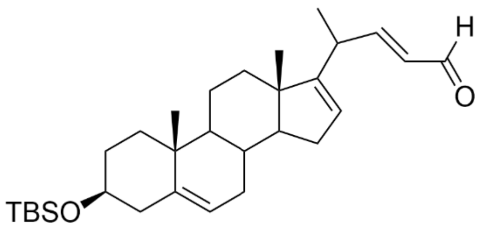

White solid (100 mg, 22% yield after 5 steps). mp 132-134 °C; ^1H NMR (500 MHz, $CDCl_3$): δ 9.52 (d, *J* = 7.9 Hz, 1H), 6.79 (dd, *J* = 15.6, 7.5 Hz, 1H), 6.10 (ddd, *J* = 15.6, 7.9, 1.2 Hz, 1H), 5.42 (t, *J* = 3.1 Hz, 1H), 5.34 – 5.27 (t, 1H), 3.51 – 3.42 (m, 1H), 3.14 (p, *J* = 7.0 Hz, 1H), 2.25 (t, *J* = 12.8 Hz, 1H), 2.16 (ddd, *J* = 13.4, 5.0, 2.4 Hz, 1H), 2.09 (ddd, *J* = 15.1, 6.5, 3.2 Hz, 1H), 2.02 – 1.96 (m, 1H), 1.89 – 1.81 (dt, 1H), 1.78 (m, 2H), 1.73 – 1.62 (m, 4H), 1.58 – 1.46 (m, 2H), 1.38 – 1.28 (m, 3H), 1.22 (d, *J* = 6.9 Hz, 3H), 1.01 (s, 3H), 0.97 (m, 1H), 0.87 (s, 9H), 0.78 (s, 3H), 0.04 (s, 6H). $^{13}C\{^1H\}$NMR (126 MHz, $CDCl_3$): δ 194.4, 163.0, 156.6, 141.8, 130.7, 124.6, 120.9, 72.5, 57.3, 50.7, 47.2, 42.8, 37.3, 36.8, 35.8, 34.8, 32.0, 31.5, 31.20, 30.5, 29.7, 25.9, 22.7, 20.7, 19.5, 19.3, 18.3, 16.4, 14.1, -4.6. HRMS (ESI) *m/z* $[M+H]^+$ calcd. for $[C_{30}H_{49}{}^{28}SiO_2]$ 469.3493, found 469.3505.

Preparation of 5m:

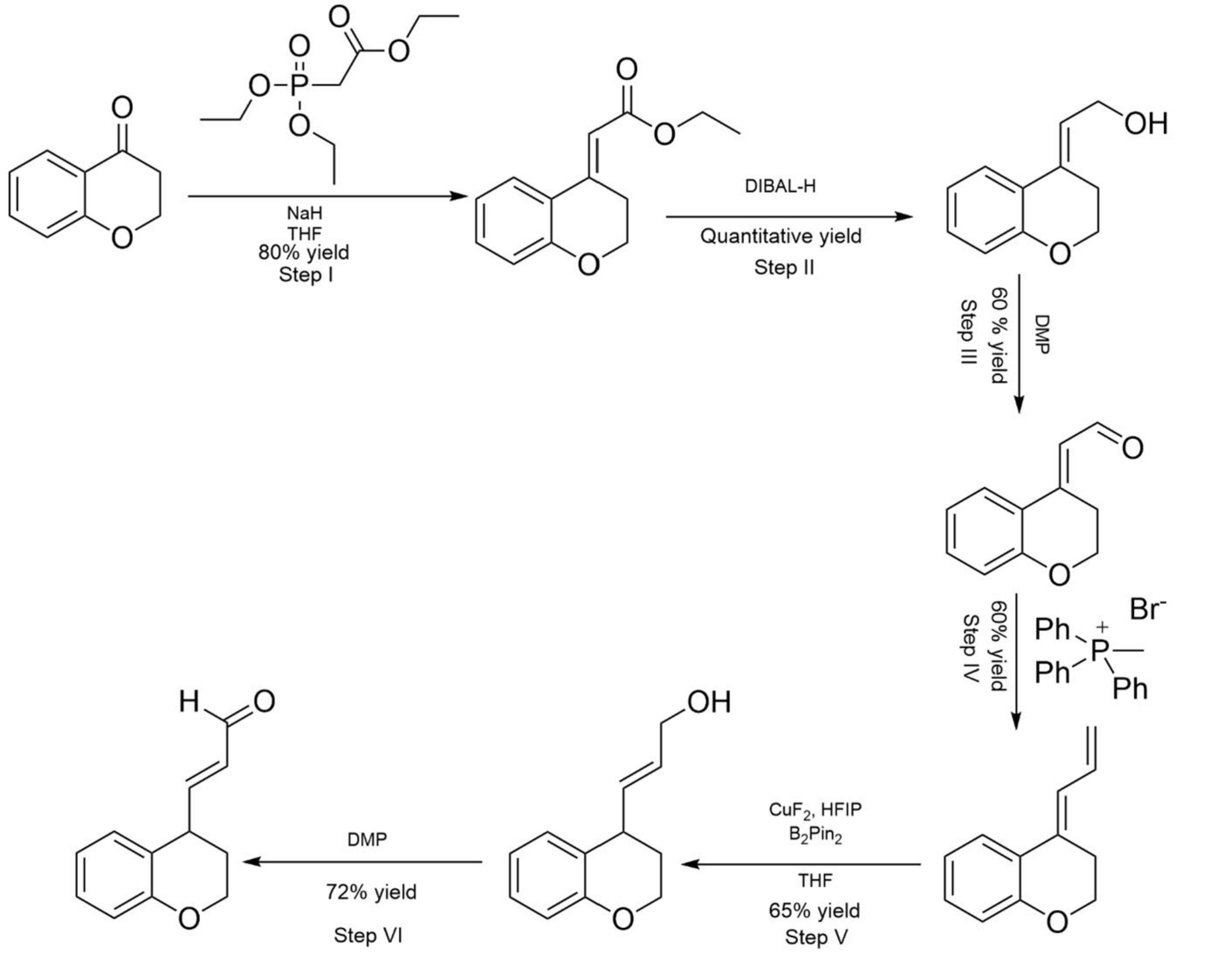

Step I: Wittig reaction: To an oven-dried round bottom flask was added NaH (1.5 equiv., 60% in mineral oil). The flask was purged and backfilled three times with N_2. Then, dry THF (0.4 M) was added and the resulting grey suspension was cooled to 0 ˚C and stirred. To the cooled suspension was added neat triethyl phosphonoacetate (1.5 equiv.) dropwise causing rapid gas evolution, and the reaction mixture was stirred for 30 min, during which time the solution became clear. The solution was warmed up to rt, then heated to 40 ˚C. A solution of the ketone (1 equiv.) in THF (1.0 M) was then added slowly and the resulting red solution was allowed to stir overnight at 40 ˚C. The mixture was cooled to 0 ˚C, diluted with Et_2O and quenched with H_2O. The layers were extracted with EtOAc (x3), washed with brine, dried over Na_2SO_4, filtered and concentrated. The residue was purified by flash chromatography (EtOAc/P.E., 2%) to afford the product as a yellow oil.

Step II: Reduction: To an oven-dried round bottom flask was added the starting material and DCM (0.25 M) and the solution was cooled to -78 ˚C. DIBAL-H (3 equiv., 1.0 M in hexane)

was added dropwise. The solution was stirred at -78 ˚C for 40 min and upon completion as judged by TLC, the reaction was quenched by MeOH and H_2O. The layers were then diluted with NaOH (1 M) until they became clear, extracted with EtOAc, washed with brine, dried over Na_2SO_4, filtered and concentrated. The crude product was taken to the next step.

Step III: Oxidation: To a solution of the alcohol in DCM (0.1 M) was added Dess-Martin periodinane (1.2 equiv.) in one portion. The mixture was stirred at rt for 10 min and quenched with sat. aq. $Na_2S_2O_3$, and diluted with a sat. aq. $NaHCO_3$ solution. The solution was stirred until the layers became transparent and was then extracted with DCM, dried over Na_2SO_4, filtered and concentrated. The residue was purified by flash chromatography (EtOAc/P.E., 1%→5%) to afford the product.

Step IV: Wittig reaction: To an oven-dried round bottom flask was added $MePPh_3Br$ (1.2 equiv.) under Ar. Then, dry THF (0.3 M) was added and the mixture was cooled to 0 ˚C. To this, *n*-BuLi (1.2 equiv., 1.6 M in hexane) was added dropwise over 20 min. The reaction mixture was allowed to stir at 0 ˚C for 1 h before the starting material in THF (2.0 M) was added dropwise to the ylide. The reaction was allowed to warm up to rt and stirred for 5 min before it was quenched with NH_4Cl and extracted with EtOAc. The organic layer was dried over Na_2SO_4, filtered and concentrated. The crude mixture was purified by flash chromatography (EtOAc/P.E., 1%) to afford the product as a yellow oil.

Step V: Hydroboration: The reaction was carried out exactly according to the literature precedent.[7]

Step VI: Oxidation: To a solution of the alcohol in DCM (0.1 M) was added Dess-Martin periodinane (1.2 equiv.) in one portion. The mixture was stirred at rt for 10 min and quenched with sat. aq. $Na_2S_2O_3$, and diluted with a sat. aq. $NaHCO_3$ solution. The solution was stirred until the layers became transparent and was then extracted with DCM, dried over Na_2SO_4, filtered and concentrated. The residue was purified by flash chromatography (EtOAc/P.E., 1%→3%) to afford the product as a white solid.

(*E*)-3-(chroman-4-yl)acrylaldehyde (5m)

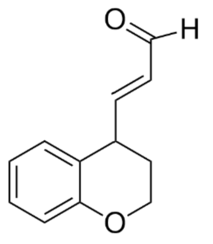

Purified by column chromatography (EtOAc/P.E., 1%→3%) and isolated as a white solid (30 mg, 14% yield after 6 steps). mp: 83-85 ˚C; ^{1}H NMR (500 MHz, $CDCl_3$): δ 9.57 (d, *J* = 7.8 Hz, 1H), 7.20 – 7.12 (m, 1H), 6.98 (dd, *J* = 7.7, 1.7 Hz, 1H), 6.89 – 6.81 (m, 3H), 6.14 (dd, *J* = 15.6, 7.7 Hz, 1H), 4.23 – 4.13 (m, 2H), 3.81 (q, *J* = 6.7 Hz, 1H), 2.27 – 2.18 (m, 1H), 2.00 (dtd, *J* = 13.5, 6.4, 3.4 Hz, 1H). $^{13}C\{^1H\}$ NMR (126 MHz, $CDCl_3$): δ 193.7, 159.5, 154.7, 134.0, 130.1, 128.9, 121.2, 120.9, 117.5, 63.5, 38.18, 27.8. HRMS (ESI) *m/z* $[M-H]^-$ calcd. for $[C_{12}H_{11}O_2]$ 187.0765, found 187.0765.

Preparation of H

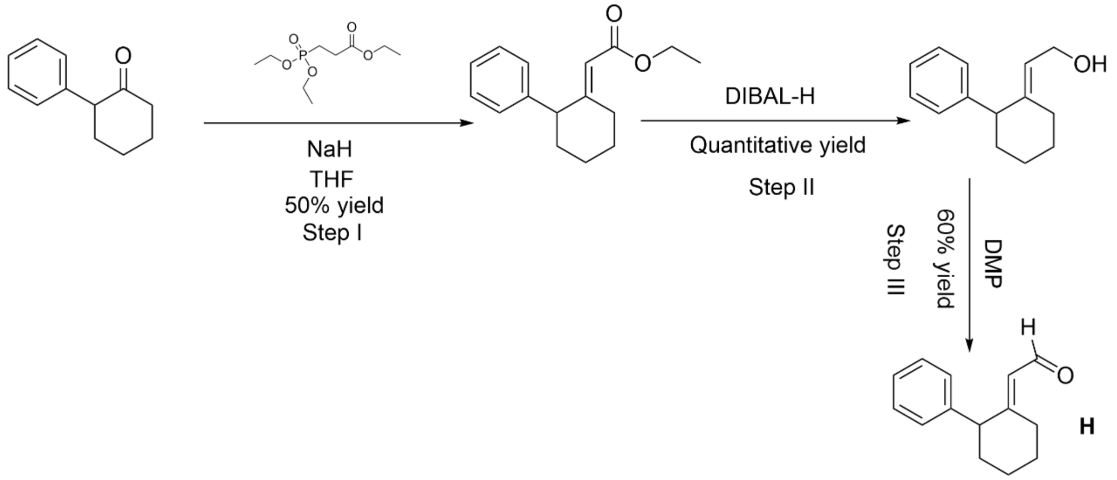

Step I: Wittig: To a suspension of NaH (60% in mineral oil, 44 mg/mmol) in dry THF (0.1 M) was added triethyl phosphonoacetate (1.1 equiv.) and the mixture was stirred for 1 h before the starting material (1 equiv.) was added. The reaction mixture was refluxed until full consumption of the starting material, cooled down to rt and then quenched by the addition of sat. aq. NH_4Cl solution at 0 ˚C. The mixture was extracted with EtOAc and the organic layer was washed with H_2O and brine, then dried over Na_2SO_4, filtered and concentrated. The crude mixture was purified by flash chromatography (EtOAc/P.E., 1%) to afford the ester.

Step II: Reduction: To a stirred solution of the ester (1.0 equiv.) in anhydrous DCM (0.2 M), cooled to -78 ˚C, DIBAL-H (2.5 equiv., 1.0 M in hexane) was added dropwise under Ar. After stirring at -78 ˚C for 1 h, the reaction mixture was quenched with MeOH and 1M NaOH. Then, the remaining mixture was warmed up to rt and stirred until the layers were clear. The aqueous phase was then extracted with DCM and the combined organic layers were washed with brine and dried over Na_2SO_4. The solvent was removed under low pressure after filtration and the crude product mixture was purified by flash chromatography (EtOAc/P.E., 10%→20%) to afford the alcohol product.

Step III: Dess-Martin oxidation: To a stirred solution of the alcohol (1.0 equiv.) in anhydrous DCM (0.1 M), DMP (1.2 equiv.) was added in one portion at rt. After stirring for 30 min, the reaction was quenched by sat. aq. $Na_2S_2O_3$ and $NaHCO_3$ solutions. The remaining mixture was stirred until no more bubbling was observed and the layers became clear. The resulting solution was then extracted with DCM and the combined organic layers were dried over Na_2SO_4. The solvent was removed under low pressure after filtration and the crude product mixture was purified by flash chromatography (EtOAc/P.E., 1%) to afford the α,β-unsaturated aldehyde.

(*E*)-2-(2-phenylcyclohexylidene)acetaldehyde (H, Figure S1)

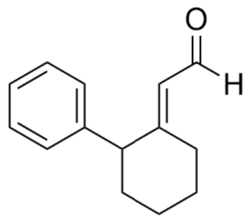

Colorless oil. (50 mg, 30% yield after 3 steps). ^{1}H NMR (500 MHz, $CDCl_3$) δ 10.07 (d, *J* = 8.1 Hz, 1H), 7.35 (t, *J* = 7.5 Hz, 2H), 7.28 (d, *J* = 3.8 Hz, 1H), 7.17 (d, *J* = 7.5 Hz, 2H), 5.38 (d, *J* = 8.0 Hz, 1H), 3.49 (dd, *J* = 11.3, 4.3 Hz, 1H), 3.44 – 3.37 (m, 1H), 2.35 (ddd, *J* = 15.8, 12.2, 4.4 Hz, 1H), 2.15 (t, *J* = 7.8 Hz, 1H), 2.10 – 1.96 (m, 3H), 1.78 – 1.60 (m, 2H). ^{13}C{^{1}H}NMR (126 MHz, $CDCl_3$): δ 190.7, 170.1, 141.0, 129.3, 128.4, 126.9, 125.8, 52.1, 34.2, 29.9, 29.5, 25.9. HRMS (ESI) *m/z* [M+H]$^+$ calcd. for [$C_{14}H_{16}O$] 201.1274, found 201.1269.

VI. γ-Hydroxylation of enals

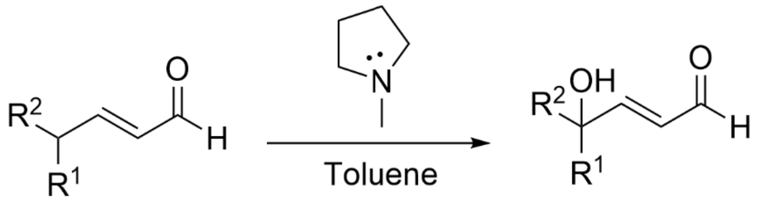

General procedure: To an oven-dried vial cooled under Ar, a mixture of starting material (1 equiv.), toluene (0.25 M) and *N*-methylpyrrolidine (1 equiv.) was added. The reaction mixture was allowed to stir open to air, at rt, until completion, as monitored by TLC and ^{1}H NMR. The crude reaction mixture was loaded directly on to a silica gel column (EtOAc/P.E., 1%→20%) to afford the desired γ-hydroxyl α,β-unsaturated aldehyde.

1 gram scale: To an oven-dried 50 mL round bottom flask, equipped with stir bar and cooled under Ar, was added a mixture of **5a** (1 g, 6.24 mmol), toluene (25 mL) and *N*-methylpyrrolidine (664 µL, 6.24 mmol). The reaction mixture was allowed to stir, open to air, at rt until completion, as monitored by TLC and ^{1}H NMR. The crude reaction mixture was concentrated and loaded directly on to a silica gel column (EtOAc/P.E., 1%→20%) to afford **7a** aldehyde in 51 % yield (560 mg).

VII. Characterization of remotely hydroxylated-α,β-unsaturated aldehydes

(*E*)-4-hydroxy-4-phenylpent-2-enal (7a)

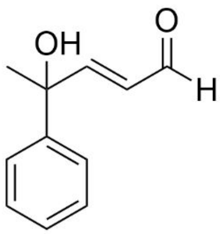

^{1}H NMR yield with cyclohexene as internal standard: trial 1=82% (Table 1), trial 2=80% (Scheme 4).

(21 mg, 67% yield) Light yellow oil. ^{1}H NMR (500 MHz, $CDCl_3$): δ 9.57 (d, J = 7.0 Hz, 1H), 7.45 (d, J = 7.6 Hz, 2H), 7.37 (t, J = 7.6 Hz, 2H), 7.30 (t, J = 7.3 Hz, 1H), 6.96 (d, J = 15.6 Hz, 1H), 6.40 – 6.33 (m, 1H), 2.19 (s, 1H), 1.77 (s, 3H). $^{13}C\{^{1}H\}$NMR (126 MHz, $CDCl_3$): δ 193.8, 161.8, 144.1, 128.8, 128.8, 127.9, 125.2, 74.4, 28.7. HRMS (ESI) *m/z* $[M+H]^+$ calcd. for $[C_{11}H_{13}O_2]$ 177.0917, found 177.0910.

(*E*)-4-(4-fluorophenyl)-4-hydroxypent-2-enal (7b)

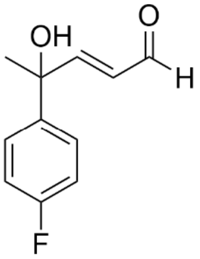

(18 mg, 59% yield). Light yellow oil. ^{1}H NMR (500 MHz, $CDCl_3$): δ 9.61 (d, J = 7.7 Hz, 1H), 7.45 (dd, J = 8.8, 5.3 Hz, 2H), 7.07 (t, J = 8.7 Hz, 2H), 6.95 (d, J = 15.6 Hz, 1H), 6.38 (dd, J = 15.6, 7.7 Hz, 1H), 2.06 (s, 1H), 1.79 (s, 3H). ^{19}F NMR (471 MHz, $CDCl_3$): δ -115.6. $^{13}C\{^{1}H\}$NMR (126 MHz, $CDCl_3$): δ 193.5, 161.1, 128.9, 127.1, 127.0, 115.7, 115.5, 74.1, 28.8. HRMS (ESI) *m/z* $[M+H]^+$ calcd. for $[C_{11}H_{12}FO_2]$ 195.0816, found 195.0825.

(*E*)-4-hydroxy-4-(p-tolyl)pent-2-enal (7c)

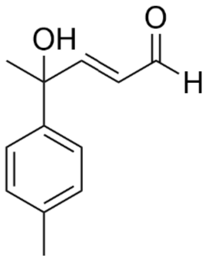

(11 mg, 41% yield). Light yellow oil. ^{1}H NMR (500 MHz, $CDCl_3$): δ 9.61 (d, J = 7.8 Hz, 1H), 7.37 (d, J = 7.9 Hz, 2H), 7.22 (d, J = 7.9 Hz, 2H), 6.98 (d, J = 15.6 Hz, 1H), 6.39 (dd, J = 15.6, 7.8 Hz, 1H), 2.38 (s, 3H), 1.79 (s, 3H). $^{13}C\{^{1}H\}$NMR (126 MHz, $CDCl_3$): δ 193.7, 161.8, 141.2, 137.8, 129.4, 128.7, 125.1, 74.3, 28.6, 20.9. HRMS (ESI) *m/z* $[M+H]^+$ calcd. for $[C_{12}H_{15}O_2]$ 191.1067, found 191.1076.

(*E*)-4-hydroxy-4-(4-methoxyphenyl)pent-2-enal (7d)

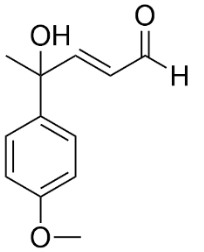

(13 mg, 41% yield). Light yellow oil. ^1H NMR (500 MHz, $CDCl_3$): δ 9.58 (d, J = 7.8 Hz, 1H), 7.36 (d, J = 8.8 Hz, 2H), 6.93 (d, J = 15.5 Hz, 1H), 6.89 (d, J = 8.9 Hz, 2H), 6.35 (dd, J = 15.6, 7.8 Hz, 1H), 3.80 (s, 3H), 1.99 (s, 1H), 1.75 (s, 3H). $^{13}C\{^1H\}$NMR (126 MHz, $CDCl_3$): δ 193.9, 161.6, 158.2, 135.9, 128.4, 126.3, 115.7, 73.9, 55.1, 28.3. HRMS (ESI) *m/z* $[M+H]^+$ calcd. for $[C_{12}H_{15}O_3]$ 207.1016, found 207.1027.

(*E*)-4-hydroxy-4-(4-(trifluoromethyl)phenyl)pent-2-enal (7e)

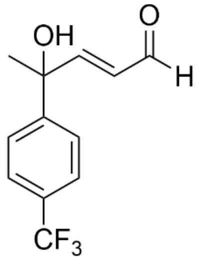

(9 mg, 28% yield). Light yellow oil. ^1H NMR (500 MHz, $CDCl_3$): δ 9.63 (d, J = 7.7 Hz, 1H), 7.70 – 7.60 (m, 4H), 6.97 (d, J = 15.6 Hz, 1H), 6.41 (dd, J = 15.6, 7.7 Hz, 1H), 2.20 (s, 1H), 1.83 (s, 3H). $^{13}C\{^1H\}$NMR (126 MHz, $CDCl_3$): δ 193.3, 160.1, 148.0, 130.6, 130.3, 130.0, 129.8, 129.4, 125.8, 125.7, 125.7, 125.7, 125.6, 125.0, 122.9, 74.2, 28.9. ^{19}F NMR (471 MHz MHz, $CDCl_3$): δ -62.62. HRMS (ESI) *m/z* $[M+H]^+$ calcd. for $[C_{12}H_{12}O_2F_3]$ 245.0784, found 245.0777.

(*E*)-4-hydroxy-4-(4-nitrophenyl)pent-2-enal (7f)

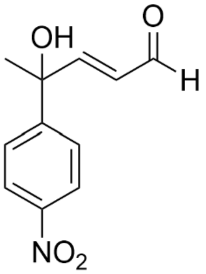

(9 mg, 34% yield at 0 ˚C). Light red oil. ^1H NMR (500 MHz, $CDCl_3$): δ 9.60 (d, J = 7.6 Hz, 1H), 8.22 (d, J = 8.9 Hz, 2H), 7.66 (d, J = 4.4 Hz, 1H), 6.92 (d, J = 15.7 Hz, 1H), 6.38 (dd, J = 15.7, 7.6 Hz, 1H), 2.17 (s, 1H), 1.81 (s, 3H). $^{13}C\{^1H\}$NMR (126 MHz, $CDCl_3$): δ 193.1, 159.2, 151.1, 147.4, 129.6, 126.2, 123.9, 74.2, 29.1. HRMS (ESI) *m/z* $[M+H]^+$ calcd. for $[C_{11}H_{12}NO_4]$ 222.0761, found 222.0762.

(*E*)-4-hydroxy-4-(3-(trifluoromethyl)phenyl)pent-2-enal (7g)

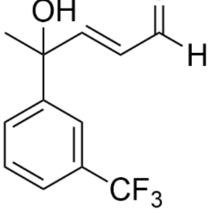

(18 mg, 55% yield). Light yellow oil. ^1H NMR (500 MHz, $CDCl_3$): δ 9.59 (d, J = 7.6 Hz, 1H), 7.74 (s, 1H), 7.63 (d, J = 7.8 Hz, 1H), 7.56 (d, J = 7.6 Hz, 1H), 7.49 (t, J = 7.8 Hz, 1H), 6.93 (d, J = 15.8 Hz, 1H), 6.36 (dd, J = 15.7, 7.6 Hz, 1H), 2.23 (s, 1H), 1.79 (s, 3H). ^{19}F NMR (471 MHz , $CDCl_3$): δ -62.6. $^{13}C\{^1H\}$NMR (126 MHz, $CDCl_3$): δ 193.4, 160.2, 145.2, 129.3, 129.2, 128.6, 124.7, 124.7, 124.7, 121.9, 121.9, 121.9, 74.1, 28.9. HRMS (ESI) *m/z* $[M-H]^-$ calcd. for $[C_{12}H_{10}O_2F_3]$ 243.0638, found 243.0644.

(*E*)-4-(3-bromophenyl)-4-hydroxypent-2-enal (7h)

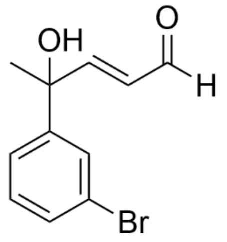

(15 mg, 45% yield). Light yellow oil. ^{1}H NMR (500 MHz, $CDCl_3$): δ 9.58 (d, *J* = 7.9 Hz, 1H), 7.61 (s, 1H), 7.43 (d, *J* = 8.2 Hz, 1H), 7.36 (d, *J* = 8.2 Hz, 1H), 7.24 (t, *J* = 3.9 Hz, 1H), 6.91 (d, *J* = 15.8 Hz, 1H), 6.35 (dd, *J* = 15.8, 7.7 Hz, 1H), 2.15 (s, 1H), 1.75 (s, 3H). ^{13}C{^{1}H}NMR (126 MHz, $CDCl_3$): δ 193.3, 160.3, 146.2, 130.8, 130.1, 128.9, 128.2, 123.6, 122.7, 73.8, 28.6. HRMS (ESI) *m/z* $[M+H]^+$ calcd. for $[C_{11}H_{12}O_2Br]$ 255.0015, found 255.0009.

(*E*)-4-hydroxy-4-(4-isobutylphenyl)pent-2-enal (7i)

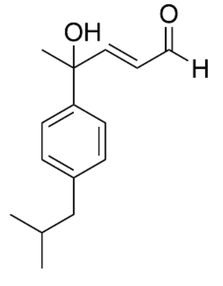

(22 mg, 52% yield). Light yellow oil. ^{1}H NMR (500 MHz, $CDCl_3$): δ 9.61 (d, *J* = 7.8 Hz, 1H), 7.38 (d, *J* = 7.9 Hz, 2H), 7.18 (d, *J* = 7.9 Hz, 2H), 6.99 (d, *J* = 15.6 Hz, 1H), 6.40 (dd, *J* = 15.6, 7.8 Hz, 1H), 2.49 (d, *J* = 7.2 Hz, 2H), 2.19 (bs, 1H), 1.88 (dp, *J* = 13.6, 6.8 Hz, 1H), 1.79 (s, 3H), 0.92 (d, *J* = 6.6 Hz, 6H). ^{13}C{^{1}H}NMR (126 MHz, $CDCl_3$): δ 193.8, 162.0, 141.6, 141.4, 129.5, 128.7, 124.9, 74.3, 44.9, 30.2, 28.5, 22.4. HRMS (ESI) *m/z* $[M-H]^-$ calcd. for $[C_{15}H_{19}O_2]$ 231.1391, found 231.1391.

(*E*)-4-hydroxy-4-(naphthalen-2-yl)pent-2-enal (7j)

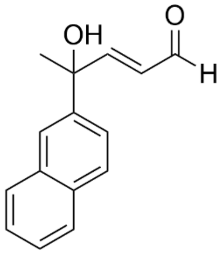

(16 mg, 48% yield). Light yellow oil. ^{1}H NMR (500 MHz, $CDCl_3$): δ 9.59 (d, *J* = 8.0 Hz, 1H), 7.91 (s, 1H), 7.87 – 7.79 (m, 3H), 7.56 – 7.39 (m, 3H), 7.03 (d, *J* = 15.6 Hz, 1H), 6.42 (dd, *J* = 15.6, 7.8 Hz, 1H), 2.25 (s, 1H), 1.86 (s, 3H). ^{13}C{^{1}H}NMR (126 MHz, $CDCl_3$): δ 193.7, 161.4, 141.3, 133.1, 132.7, 129.0, 128.6, 128.2, 127.6, 126.5, 126.5, 123.7, 123.4, 74.5, 28.6. HRMS (ESI) *m/z* $[M+H]^+$ calcd. for $[C_{15}H_{15}O_2]$ 227.1067, found 227.1074.

(*E*)-4-hydroxy-4-(thiophen-3-yl)pent-2-enal (7k)

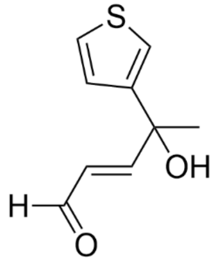

(10 mg, 35% yield at 0˚C). Light yellow oil. ^{1}H NMR (500 MHz, $CDCl_3$): δ 9.58 (d, *J* = 7.8 Hz, 1H), 7.33 (dd, *J* = 5.1, 3.0 Hz, 1H), 7.24 (s, 1H), 7.06 (dd, *J* = 5.1, 1.4 Hz, 1H), 6.95 (d, *J* = 15.6 Hz, 1H), 6.35 (dd, *J* = 15.5, 7.8 Hz, 1H), 2.21 (s, 1H), 1.76 (s, 3H). ^{13}C{^{1}H}NMR (126 MHz, $CDCl_3$): δ 193.7, 161.0, 145.7, 128.8, 126.9, 125.5, 121.0, 72.8, 28.6. HRMS (ESI) *m/z* $[M-H]^-$ calcd. for $[C_9H_9SO_2]$ 181.0329, found 181.0325.

(2*E*,4*Z*)-4-((3*S*,10*R*,13*S*,16*S*)-3-((tert-butyldimethylsilyl)oxy)-16-hydroxy-10,13-dimethyl-1,2,3,4,7,8,9,10,11,12,13,14,15,16-tetradecahydro-17H-cyclopenta[a]phenanthren-17-ylidene)pent-2-enal (7l major)

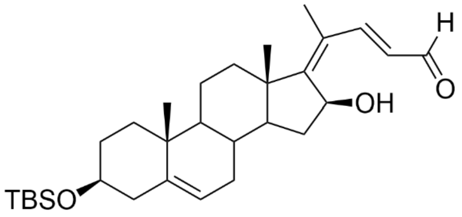

(9 mg, 20% yield at 45 °C with DABCO). Light yellow oil. ^{1}H NMR (500 MHz, $CDCl_3$): δ 9.66 (d, *J* = 7.7 Hz, 1H), 7.80 (d, *J* = 15.6 Hz, 1H), 6.23 (dd, *J* = 15.5, 7.7 Hz, 1H), 5.36 (t, *J* = 5.3 Hz, 1H), 4.99 (t, *J* = 4.9 Hz, 1H), 3.51 (dq, *J* = 10.7, 5.5 Hz, 1H), 2.45 – 2.39 (m, 1H), 2.30 (t, *J* = 12.0 Hz, 1H), 2.21 (d, *J* = 12.8 Hz, 1H), 2.19 (s, 1), 2.05 (m, 1H), 1.98 (s, 3H), 1.87 – 1.76 (m, 4H), 1.76 – 1.60 (m, 7H), 1.54 (m, 2H), 1.05 (s, 3H), 0.98 (s, 3H), 0.91 (s, 9H), 0.09 (s, 6H). ^{13}C{^{1}H}NMR (126 MHz, $CDCl_3$): δ 194.5, 162.5, 153.9, 141.7, 129.4, 128.2, 120.7, 72.5, 71.8, 52.6, 49.9, 45.8, 42.7, 37.2, 36.6, 35.5, 31.9, 31.6, 30.6, 29.7, 25.9, 21.2, 19.4, 18.3, 16.5, 14.1, -4.6. HRMS (ESI) *m/z* $[M-H]^-$ calcd. for $[C_{30}H_{47}O_3{}^{28}Si]$ 483.3300, found 483.3299.

(2*E*,4*E*)-4-((3*S*,10*R*,13*S*,16*S*)-3-((tert-butyldimethylsilyl)oxy)-16-hydroxy-10,13-dimethyl-1,2,3,4,7,8,9,10,11,12,13,14,15,16-tetradecahydro-17H-cyclopenta[a]phenanthren-17-ylidene)pent-2-enal (7l minor)

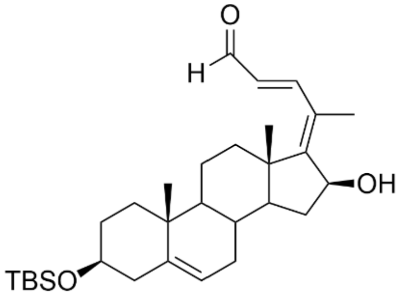

(4 mg, 10% yield at 45 °C with DABCO). Light yellow oil. ^{1}H NMR (500 MHz, $CDCl_3$): δ 9.59 (d, *J* = 7.8 Hz, 1H), 7.81 (d, *J* = 15.6 Hz, 1H), 6.18 (dd, *J* = 15.6, 7.4 Hz, 1H), 5.32 (s, 1H), 4.80 – 4.76 (m, 1H), 3.47 (m, 1H), 2.40 (d, *J* = 13.2 Hz, 1H), 2.26 (t, *J* = 12.7 Hz, 1H), 2.20 – 2.15 (m, 1H), 2.00 (s, 3H), 1.77 (dd, *J* = 24.0, 10.1 Hz, 1H), 1.7 – 1.28 (m, 11H), 1.05 (m, 1H), 1.01 (s, 3H), 0.99 (s, 3H), 0.95 (m, 1), 0.87 (s, 9 H), 0.04 (s, 6H). ^{13}C{^{1}H}NMR (126 MHz, CDCl3): δ194.2, 162.9, 151.2, 141.9, 129.6, 128.9, 120.9, 73.3, 72.7, 53.3, 49.9, 46.0, 42.9, 39.4, 37.4, 36.8, 35.3, 32.2, 31.8, 30.9, 26.1, 21.7, 19.6, 19.2, 18.5, 16.5, -4.4. HRMS (ESI) *m/z* $[M-H]^-$ calcd. for $[C_{30}H_{47}O_3{}^{28}Si]$ 483.3300, found 483.3299.

(2*E*,4*Z*)-4-((3*S*,10*R*,13*S*)-3-((tert-butyldimethylsilyl)oxy)-10,13-dimethyl-16-oxo-1,2,3,4,7,8,9,10,11,12,13,14,15,16-tetradecahydro-17H-cyclopenta[a]phenanthren-17-ylidene)pent-2-enal (19). Inseparable mixture of 2:1 diastereomers.

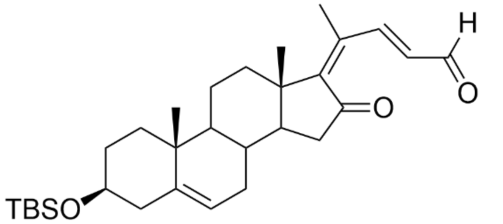

(13 mg, 30% yield at 45 °C with DABCO). Light yellow oil. ^{1}H NMR (500 MHz, $CDCl_3$): δ 9.72 (d, *J* = 9.2 Hz, 1H), 8.81 (d, *J* = 16.0 Hz, 1H), 7.79* (d, *J* = 15.7 Hz, 1H), 6.50* (dd, *J* = 15.7, 7.5 Hz, 1H), 6.40 (dd, *J* = 15.9, 7.8 Hz, 1H), 5.35 (s, 1H), 3.51 (dq, *J* = 10.6, 5.4 Hz, 1H), 2.55 – 2.34 (m, 1H), 2.32* (s, 3H), 2.28 – 2.22 (m, 2H), 2.15 (dd, *J* = 14.4, 5.0 Hz, 1H), 2.10 (s, 3H), 2.03 – 1.52 (m, 10H), 1.34 – 1.22 (m, 2H), 1.14* (s, 3H), 1.13 (m,1H), 1.08 (s, 3H), 0.92 (s, 9H), 0.09 (s, 6H). ^{13}C{^{1}H}NMR (126 MHz, $CDCl_3$): δ 208.0*, 207.4, 195.0, 193.3*, 151.3, 150.5, 150.1*, 141.8, 138.6, 138.1, 133.5, 132.3*, 120.3, 72.4, 49.8, 49.7, 49.6, 49.6, 45.8, 45.7, 42.7, 39.7, 39.6*, 38.8, 37.0, 36.9, 36.7, 36.6, 31.9, 31.5, 30.9, 30.9, 25.9, 21.3, 21.0, 19.4, 18.3, 18.2*, 16.4, 15.2, 15.1, -4.6. HRMS (ESI) *m/z* [M-H]$^-$ calcd. for [$C_{30}H_{45}O_3{}^{28}Si$] 481.3144, found 481.3141.

*indicates minor diastereomer

(E)-3-(4-hydroxychroman-4-yl)acrylaldehyde (7m)

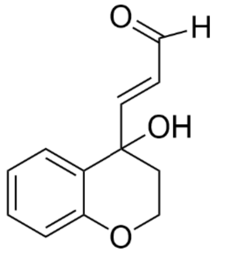

(15 mg, 42% yield at 0 ˚C). Light yellow oil. ^{1}H NMR (500 MHz, $CDCl_3$): δ 9.71 (d, *J* = 7.8 Hz, 1H), 7.28 (t, *J* = 4.7 Hz, 2H), 7.19 (d, *J* = 7.7 Hz, 1H), 6.94 (dt, *J* = 13.7, 6.7 Hz, 2H), 6.65 (dd, *J* = 15.5, 7.8 Hz, 1H), 4.36 (dd, *J* = 7.6, 3.2 Hz, 2H), 2.28 – 2.18 (m, 2H), 2.09 (dt, *J* = 14.3, 3.3 Hz, 1H). ^{13}C{^{1}H}NMR (126 MHz, $CDCl_3$): δ 193.2, 160.2, 154.3, 131.2, 130.6, 128.1, 123.8, 121.2, 117.8, 69.3, 62.2, 35.4. HRMS (ESI) *m/z* [M-H]$^-$ calcd. for [$C_{12}H_{11}O_3$] 203.0714, found 203.0714.

(*E*)-4-hydroxy-4-phenylhex-2-enal (7n)

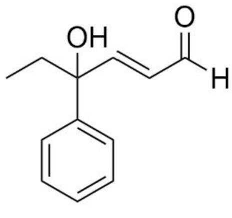

(15 mg, 48% yield). Colorless oil. ^{1}H NMR (500 MHz, $CDCl_3$): δ 9.56 (d, *J* = 8.8 Hz, 1H), 7.43 (d, *J* = 7.5 Hz, 2H), 7.37 (t, *J* = 7.8 Hz, 2H), 7.28 (t, *J* = 7.9 Hz, 1H), 6.98 (d, *J* = 15.7 Hz, 1H), 6.38 (ddd, *J* = 15.6, 7.8, 0.9 Hz, 1H), 2.06 (bs, 1H), 2.05 (m, 2H), 0.88 (t, *J* = 7.9 Hz, 3H). ^{13}C{^{1}H}NMR (126 MHz, $CDCl_3$): δ 193.6, 161.4, 143.0, 129.4, 128.6, 127.5, 125.2, 76.9, 33.8, 7.5. HRMS (ESI) *m/z* [M+H]$^+$ calcd. for [$C_{12}H_{14}O_2Na$] 213.0886, found 213.0882.

(*E*)-4-benzyl-5,5,5-trifluoro-4-hydroxypent-2-enal (from Figure S1)

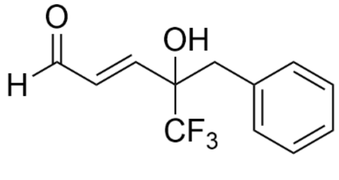

(2 mg, 8% yield at 45 ˚C). Light yellow oil. ^{1}H NMR (500 MHz, $CDCl_3$): δ 9.58 (d, *J* = 7.5 Hz, 1H), 7.30 (t, *J* = 7.0 Hz, 3H), 7.21 – 7.10 (m, 2H), 6.78 (d, *J* = 15.8 Hz, 1H), 6.23 (dd, *J* = 15.7, 7.5 Hz, 1H), 3.23 (d, *J* = 14.1 Hz, 1H), 3.10 (d, *J* = 14.0 Hz, 1H), 2.36 (s, 1H). ^{19}F NMR (471 MHz, $CDCl_3$): δ -79.3. ^{13}C{^{1}H}NMR (126 MHz, $CDCl_3$): δ 192.1, 149.8, 134.7, 132.1, 130.8, 130.5, 128.9, 128.2, 113.9, 76.7, 76.5, 40.5. HRMS (ESI) *m/z* [M-H]$^-$ calcd. for [$C_{12}H_{10}O_2F_3$] 243.0638, found 243.0637.

Reaction conditions with E (from Figure S1)

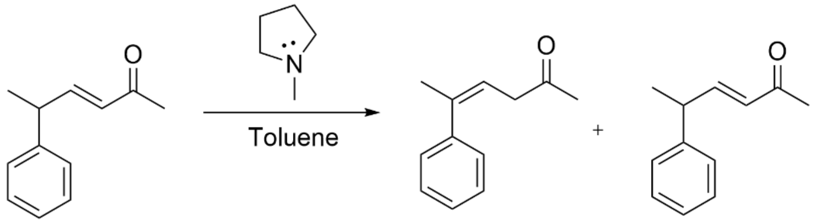

Procedure: To an oven-dried vial cooled under Ar, a mixture of starting material (25 mg, 1 equiv.), toluene (580 µL, 0.25 M) and *N*-methylpyrrolidine (15.2 µL,1 equiv.) was added. The reaction mixture was allowed to stir, open to air, at rt and monitored. After 16 h, a mixture of starting material and isomerized product was observed. Then, *N*-methylpyrrolidine (1 equiv.) was added every 16 h until full conversion to the isomerized form was observed. The product was then purified by a silica plug (EtOAc/P.E., 20%).

(*Z*)-5-phenylhex-4-en-2-one

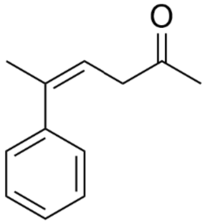

(25 mg, quantitative yield). Colorless oil. ^{1}H NMR (500 MHz, $CDCl_3$) δ 7.43 (d, *J* = 7.5 Hz, 2H), 7.39 – 7.31 (m, 2H), 7.28 (d, *J* = 7.3 Hz, 1H), 6.00 (t, *J* = 7.2 Hz, 1H), 3.37 (d, *J* = 7.2 Hz, 2H), 2.24 (s, 3H), 2.09 (s, 3H). ^{13}C{^{1}H}NMR (126 MHz, $CDCl_3$): δ 206.4, 143.1, 138.4, 128.8, 128.3, 127.1, 125.8, 119.2, 43.9, 16.3. HRMS (ESI) *m/z* [M-H]$^+$ calcd. for [$C_{12}H_{15}O$] 175.1117 found 175.1120.

VIII. Preparation of products 9 and 10

Reaction Conditions:

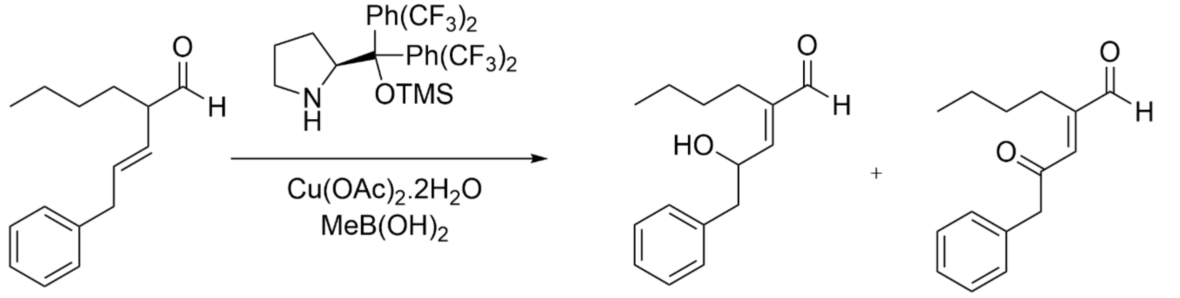

Procedure: On the benchtop, to a round bottom flask containing $MeB(OH)_2$ (2 equiv.), 2,4,6-triphenylboroxin (0.67 equiv.), organocatalyst (0.2 equiv.), TFA (0.2 equiv.), and activated 4 Å mol. sieves (1g/mmol of substrate), was added EtOAc (0.05 M), aldehyde (1 equiv.),[8] and $Cu(OAc)_2.H_2O$ (0.3 equiv.), respectively. Stirring was initiated and the reaction was sparged by a gentle stream of O_2 for 60 s. The reaction mixture was then stirred under O_2 atmosphere for 10-16 h at rt and purified by preparative TLC (EtOAc/P.E., 20%).

(*E*)-2-(2-hydroxy-3-phenylpropylidene)hexanal (9)

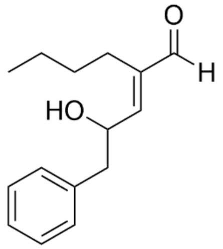

(4 mg, 10% yield). Colorless oil. ^{1}H NMR (500 MHz, $CDCl_3$): δ 9.44 (s, 1H), 7.41-7.25 (m, 5H), 6.41 (d, J = 8.5 Hz, 1H), 4.86 (d, J = 7.5 Hz, 1H), 2.98 – 2.91 (m, 2H), 2.21 (m, 2H), 1.82 (s, 1H), 1.30 (m, 4H), 0.90 (t, J = 7.0 Hz, 3H). ^{13}C{^{1}H}NMR (126 MHz, $CDCl_3$): δ 194.9, 152.7, 143.7, 136.4, 129.6, 128.8, 127.1, 69.4, 43.4, 31.0, 24.3, 22.9, 13.8. HRMS (ESI) *m/z* $[M-H]^-$ calcd. for $[C_{15}H_{19}O_2]$ 231.1391, found 231.1392.

(*E*)-2-(2-oxo-3-phenylpropylidene)hexanal (10)

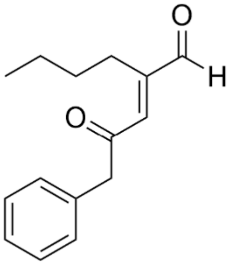

(11 mg, 69% yield). Colorless oil. ^{1}H NMR (500 MHz, $CDCl_3$): δ 9.44 (s, 1H), 7.38 (t, J = 7.4 Hz, 2H), 7.33 (d, J = 7.0 Hz, 1H), 7.25 (d, J = 7.5 Hz, 2H), 6.74 (s, 1H), 3.91 (s, 2H), 2.60 (t, 2H), 1.36 – 1.26 (m, 4H), 0.94 – 0.83 (m, 3H). ^{13}C{^{1}H}NMR (126 MHz, $CDCl_3$): δ 197.9, 194.87, 152.9, 139.2, 133.1, 129.5, 129.0, 127.5, 51.5, 30.9, 24.8, 22.9, 13.8. HRMS (ESI) *m/z* $[M+H]^+$ calcd. for $[C_{15}H_{19}O_2]$ 231.1379, found 231.1377.

Synthesis of γ-hydroperoxy-enal (6a-H)

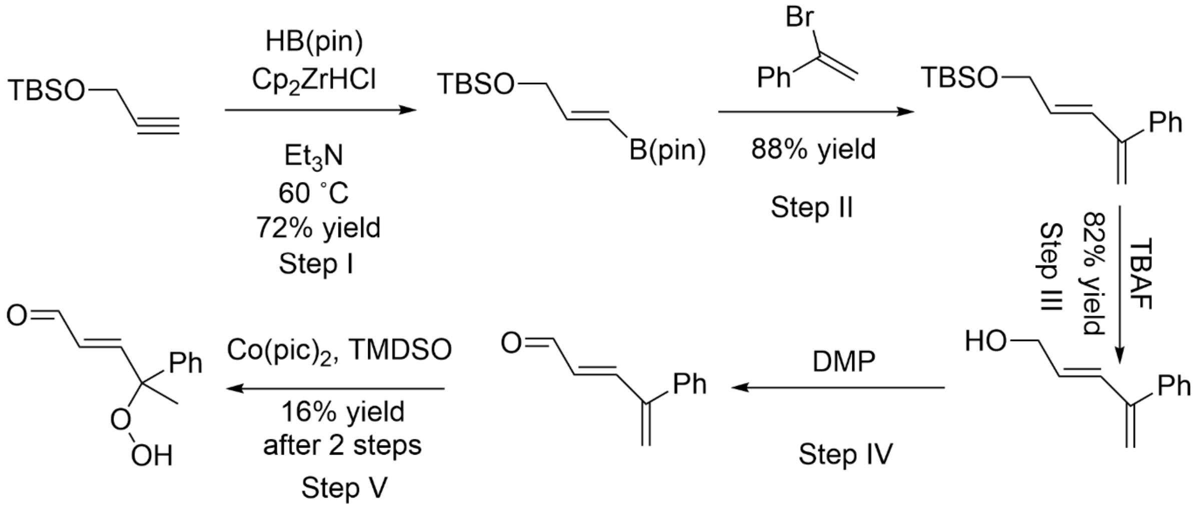

Procedure:

Step I: Terminal alkyne (1 equiv.), HB(pin) (1.05 equiv.), Et_3N (0.1 equiv.), and Cp_2ZrHCl (0.025 equiv.) were combined in a pressure tube under Ar atmosphere and allowed to stir at 60 ˚C for 15-20 h. The reaction contents were cooled and diluted with H_2O (2 mL/mmol alkyne). The aqueous layer was extracted with EtOAc. Combined organic fractions were washed with brine, dried over Na_2SO_4, filtered, and concentrated under reduced pressure. The crude mixture was purified by flash chromatography (EtOAc/P.E., 1%→5%) to afford the product.

Step II: To a pressure tube equipped with a stir bar, was added G_3Pd(XPhos) (0.02 equiv.), which was then dissolved in THF (2 mL/mmol starting material) and allowed to stir at rt for 10 min. To the resulting grey solution was added starting material (1 equiv.) and α-bromo styrene (1 equiv.). To this solution was added a freshly degassed aq. solution of K_3PO_4 (4 mL/mmol starting material, 0.5 M aqueous solution) and the reaction was allowed to stir at 40 ˚C for 2 h. The reaction was then cooled to rt. The aqueous layer was separated from the organic layer, and washed with Et_2O. The combined organic fractions were dried over Na_2SO_4, filtered, and concentrated under reduced pressure. The crude mixture was purified by flash chromatography (Et_2O /P.E., 0%→2%) to afford the product.

Step III: To a stirred solution of protected diene in THF (0.073 M) was added TBAF (2 equiv., 1.0 M in THF). Stirring was initiated and continued for 20 min before the mixture was diluted with Et_2O. The mixture was washed with sat. aq. NH_4Cl solution, brine, then dried, filtered, and concentrated. The crude product mixture was purified by flash chromatography (EtOAc/P.E., 20 %) to afford the product.

Step IV: To a solution of the corresponding alcohol (1 equiv.) in DCM (2.5 mL/mmol starting material) at 0 ˚C, was added DMP (1.1 equiv.) in one portion. The reaction mixture was

allowed to stir for 15 min at 0 ˚C, then quenched with sat. aq. $Na_2S_2O_3$ and $NaHCO_3$ solutions. The mixture was extracted with DCM. The organic layers were washed with H_2O and brine, dried over Na_2SO_4, filtered and concentrated to about (3 mL/mmol) of DCM left in the flask, to avoid decomposition of product when neat. The crude product solution was loaded on to a column (EtOAc/P.E., 2 %) and pure fractions were concentrated to about (5.5 mL/mmol) of solvent before proceeding to the next step.

Step V: To a solution of the dienal (1 equiv.), was added isopropanol (0.003 M) and $Co(pic)_2$ (0.05 equiv.). The reaction mixture was sonicated until the catalyst was dissolved (1 min). Then, TMDSO (2.2 equiv.), *t*-BuOOH (0.2 equiv., 1 M in decane), and $CHCl_3$ (3 equiv.) were added. The reaction mixture was sparged with a balloon of O_2 for 2 minutes and stirred for 48 h. The reaction mixture was concentrated, filtered through a pad of silica (EtOAc/P.E., 40%), concentrated and purified by flash chromatography (EtOAc/P.E., 5 %→20%) to afford the product.

(*E*)-4-hydroperoxy-4-phenylpent-2-enal (6a-H)

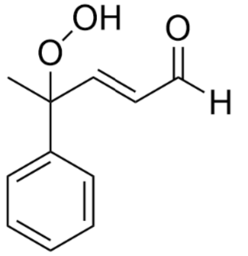

(80 mg, 8% yield over 5 steps). Colorless viscous oil. ^{1}H NMR (500 MHz, $CDCl_3$): δ 9.66 (d, J = 7.7 Hz, 1H), 7.59 (s, 1H), 7.48 – 7.33 (m, 5H), 7.11 (d, J = 16.1 Hz, 1H), 6.33 (dd, J = 16.1, 7.7 Hz, 1H), 1.84 (s, 3H). $^{13}C\{^1H\}$NMR (126 MHz, $CDCl_3$): δ 193.5, 156.9, 140.2, 131.8, 128.9, 128.6, 126.2, 85.8, 23.3. HRMS (ESI) *m/z* $[M-H]^-$ calcd. for $[C_{11}H_{11}O_3]$ 191.0714, found 191.0719.

In situ formation of dienamines 18a and 18b

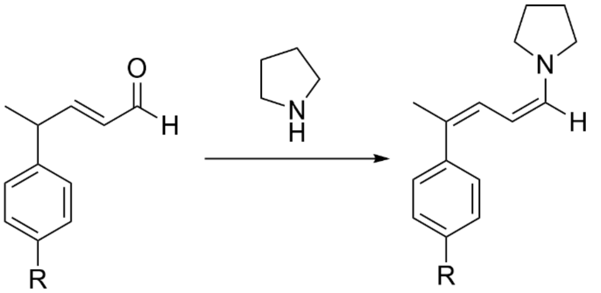

Procedure: To an oven-dried 2 dram vial equipped with stir bar and cooled under Ar, was added aldehyde (25 mg, 0.1560 mmol) and toluene (0.25 M). To this mixture, pyrrolidine (12.8 μL, 0.1560 mmol) was added and the mixture was allowed to stir for 4-5 h until complete conversion to dienamine as determined by ^{1}H NMR.

1-4-phenylpenta-1,3-dien-1-yl)pyrrolidine (18a)

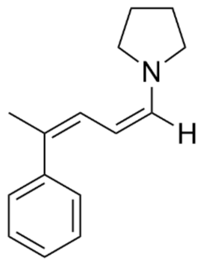

(33 mg, quantitative yield). Dark yellow oil. ^{1}H NMR (500 MHz, $CDCl_3$) δ 7.38 (d, *J* = 8.3 Hz, 2H), 7.37 – 7.30 (m, 1H), 7.28 – 7.08 (m, 2H), 6.65 (d, *J* = 13.0 Hz, 1H), δ 6.50* (d, *J* = 13.2 Hz, 1H), 6.46 (d, *J* = 11.0 Hz, 1H), 6.01* (d, *J* = 11.0 Hz, 1H), 5.11 (t, *J* = 13.0 Hz, 1H), 3.20 (t, 4H), 3.07 – 3.01* (m, 4H), 2.06* (s, 3H), 2.05 (s, 3H), 1.99 – 1.82 (m, 4H). 1.84 – 1.77* (m, 4H). ^{13}C{^{1}H}NMR (126 MHz, $CDCl_3$) δ 144.5, 143.4, 140.4, 139.4, 133.3, 128.8, 128.5, 128.4, 128.3, 128.2, 127.6, 127.5, 125.8, 125.1, 124.8, 123.2, 96.8, 49.2, 49.1, 25.5, 25.3, 15.6. HRMS (ESI) m/z $[M+H]^+$ calcd. for $[C_{15}H_{20}N]$ 214.1590, found 214.1601.

*indicates minor diastereomer

1-(4-(4-fluorophenyl)penta-1,3-dien-1-yl)pyrrolidine (18b)

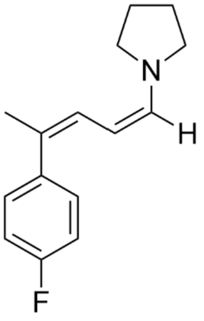

(36 mg, quantitative yield). Dark yellow oil. ^{1}H NMR (500 MHz, $CDCl_3$) δ 7.38 – 7.25 (m, 2H), 6.98 (t, *J* = 8.8 Hz, 2H), 6.68 (d, *J* = 12.9 Hz, 1H), δ 6.55* (d, *J* = 13.1 Hz, 1H), 6.41 (d, *J* = 11.0 Hz, 1H), 6.04* (d, *J* = 11.2 Hz, 1H), 5.13 (t, *J* = 12.1 Hz, 1H), 5.07* (t, *J* = 12.1 Hz, 1H), 3.26 – 3.17 (m, 4H), 3.08* (t, *J* = 6.7 Hz, 4H), 2.08 (s, 3H), 2.07* (s, 3H), 1.94 (d, *J* = 3.2 Hz, 4H), 1.85* (t, *J* = 3.4 Hz, 4H). ^{19}F NMR (470 MHz, $CDCl_3$) δ -117.24*, -117.25*, -117.25*, -117.27*, -117.28*, -117.29*, -117.30*, -118.91, -118.92, -118.93, -118.94, -118.95, -118.96, -118.97. ^{13}C{^{1}H}NMR (126 MHz, $CDCl_3$) δ 161.9, 159.9, 140.5, 140.5, 140.1, 139.3, 139.1, 129.7,

129.6, 127.4, 127.3, 125.9, 125.8, 123.9, 122.0, 114.8, 114.7, 96.5, 96.2, 48.9, 48.9, 25.3, 25.2, 25.1, 15.6. HRMS (ESI) m/z $[M+H]^+$ calcd. for $[C_{15}H_{19}FN]$ 232.1496, found 232.1495.

*indicates minor diastereomer

Isolation of Acetophenone (17a) and push-pull enamine (16)

Procedure: To a 1 dram vial was added **5a** (24 mg, 0.1249 mmol) and pyrrolidine (1.0 µL, 0.01249) in toluene (500 µL). The reaction was stirred overnight at rt and purified by flash chromatography (EtOAc/P.E., 5%→100% → MeOH, 100%) to afford acetophenone in 17% yield (2.5 mg) and the push-pull enamine in 10% yield (2 mg).

Acetophenone (17a)

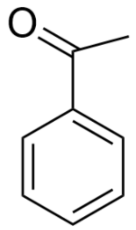

Colorless oil. ^1H NMR (500 MHz, $CDCl_3$) δ 7.97 – 7.91 (m, 2H), 7.53 (d, J = 7.5 Hz, 1H), 7.44 (dd, J = 8.4, 7.1 Hz, 2H), 2.58 (s, 3H). $^{13}C\{^1H\}$NMR (126 MHz, $CDCl_3$): δ 198.3, 137.2, 133.2, 128.7, 128.4, 26.7. HRMS (ESI) *m/z* $[M+H]^+$ calcd. for $[C_8H_8O]$ 121.0648, found 121.0649.

(*E*)-3-(pyrrolidin-1-yl)acrylaldehyde (16)

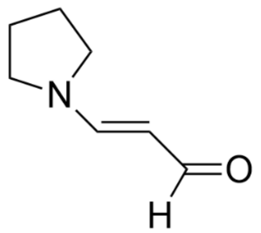

Dark yellow oil. ^1H NMR (500 MHz, $CDCl_3$): δ 9.08 (d, J = 8.7 Hz, 1H), 7.28 (s, 1H), 5.16 – 5.08 (m, 1H), 3.54 (t, J = 6.7 Hz, 2H), 3.22 (t, J = 7.1 Hz, 2H), 2.02-1.96 (m, 4H). $^{13}C\{^1H\}$NMR (126 MHz, $CDCl_3$): δ 189.0, 155.9, 102.5, 52.3, 47.1, 25.3, 25.2. HRMS (ESI) *m/z* $[M+H]^+$ calcd. for $[C_7H_{12}NO]$ 126.0913, found 126.0916.

HRMS analysis of crude reaction mixture.

Procedure: To an oven-dried vial, cooled under Ar, a mixture of starting material (1 equiv.), toluene (0.25 M) and *N*-methylpyrrolidine (1 equiv.) was added. The reaction mixture was allowed to stir, open to air, at rt until completion, as monitored by TLC and ^1H NMR. The crude reaction mixture was analyzed by Mass Spectroscopy.

1-hydroxy-1-methylpyrrolidin-1-ium

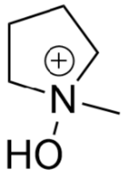

HRMS (ESI) *m/z* [M+H] calcd. for [$C_5H_{12}NO^+$] 102.0913, found 102.0910.

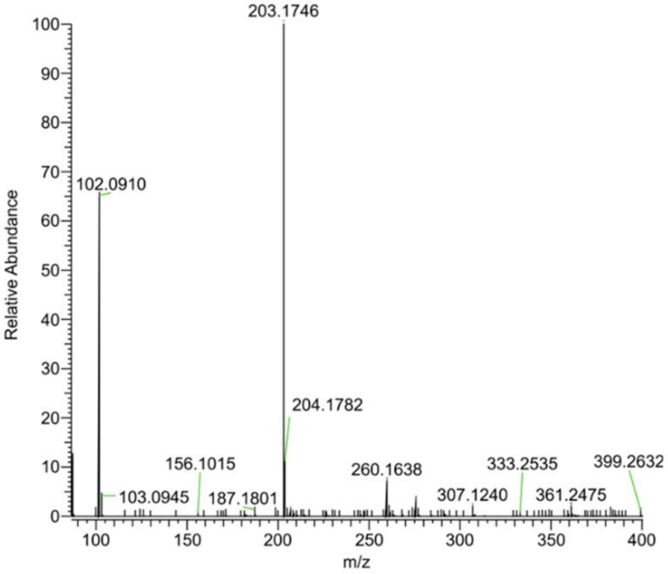

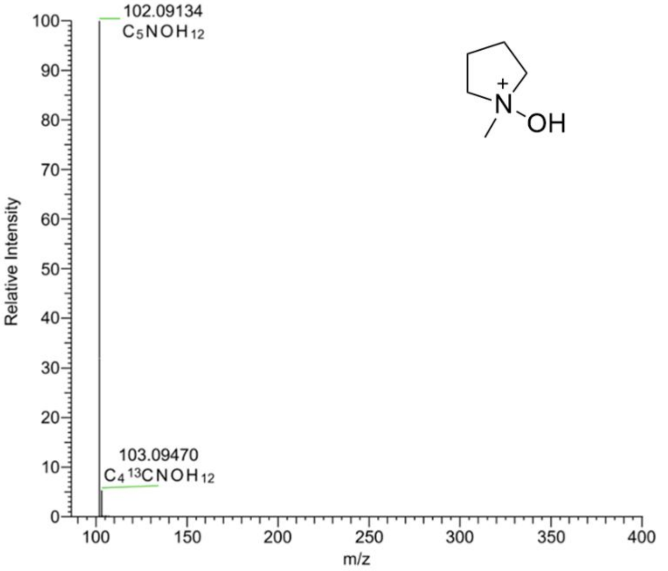

IX. Asymmetric γ-Hydroxylation of aldehydes

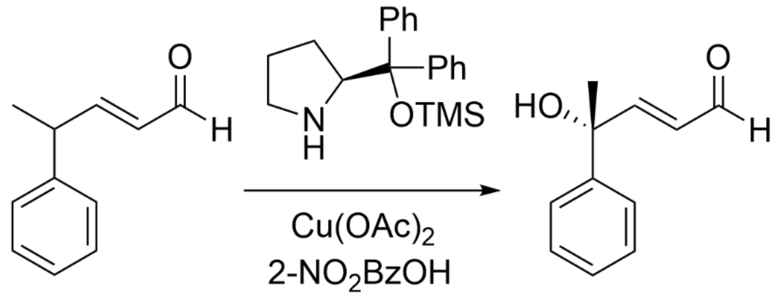

Procedure: To a vial was added 2-nitrobenzoic acid (2.6 mg, 0.1 equiv.), $Cu(OAc)_2$ (2.8 mg, 0.1 equiv.), catalyst (5.1 mg, 0.1 equiv.), aldehyde (25 mg, 1 equiv.) and toluene (625 µL, 0.25 M). The reaction was stirred open to air at rt for 16 h and purified by flash chromatography (EtOAc/P.E., 3%→20%) to afford **7a**.

(*R,E*)-4-hydroxy-4-phenylpent-2-enal (7a)

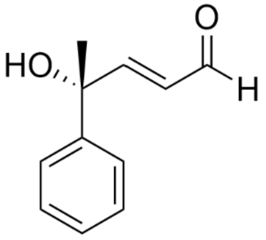

(8 mg, 28% yield, 93:6 er) Light yellow oil. ^{1}H NMR (500 MHz, $CDCl_3$): δ 9.57 (d, *J* = 7.0 Hz, 1H), 7.45 (d, *J* = 7.6 Hz, 2H), 7.37 (t, *J* = 7.6 Hz, 2H), 7.30 (t, *J* = 7.3 Hz, 1H), 6.96 (d, *J* = 15.6 Hz, 1H), 6.40 – 6.33 (m, 1H), 2.19 (s, 1H), 1.77 (s, 3H). ^{13}C{^{1}H}NMR (126 MHz, $CDCl_3$): δ 193.8, 161.8, 144.1, 128.8, 128.8, 127.9, 125.2, 74.4, 28.7. HPLC with an OD- H column (n-hexane/i-PrOH= 80:20 at 1.1 mL/min, 220 nm); major enantiomer t_R = 10.77 min, minor enantiomer t_R = 12.74 min; HRMS (ESI) *m/z* $[M+H]^+$ calcd. for $[C_{11}H_{13}O_2]$ 177.0917, found 177.0910. $[\alpha]_D^{22}$= +7.6 (c 0.19, CH_2Cl_2).

(*R,E*)-4-hydroxy-4-(p-tolyl)pent-2-enal (7c)

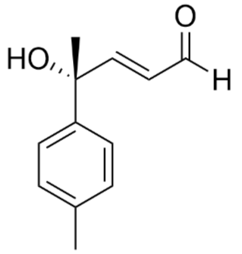

As above, except at 0 ˚C. (10 mg, 18% yield, 92:7 er). Light yellow oil. ^{1}H NMR (500 MHz, $CDCl_3$): δ 9.61 (d, *J* = 7.8 Hz, 1H), 7.37 (d, *J* = 7.9 Hz, 2H), 7.22 (d, *J* = 7.9 Hz, 2H), 6.98 (d, *J* = 15.6 Hz, 1H), 6.39 (dd, *J* = 15.6, 7.8 Hz, 1H), 2.38 (s, 3H), 1.79 (s, 3H). ^{13}C{^{1}H}NMR (126 MHz, $CDCl_3$): δ 193.7, 161.8, 141.2, 137.8, 129.4, 128.7, 125.1, 74.3, 28.6, 20.9. HPLC with an OD- H column (n-hexane/i-PrOH= 80:20 at 1.1 mL/min, 220 nm); major enantiomer t_R = 10.65 min, minor enantiomer t_R = 15.71 min HRMS (ESI) *m/z* $[M+H]^+$ calcd. for $[C_{12}H_{15}O_2]$ 191.1067, found 191.1076. $[\alpha]_D^{22}$= +1.1 (c 0.34, CH_2Cl_2).

Synthesis of Boivinianin A

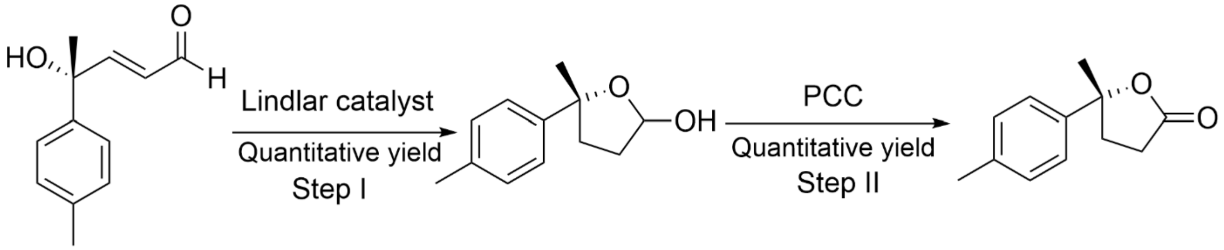

Procedure: *Step I:* To a 2-neck round bottom flask was added the aldehyde (14 mg, 1 equiv.) and Lindlar catalyst (3 mg) to 3mL of a 1:1 solution of EtOAc:EtOH. The flask was evacuated of air by applying vacuum and then the solution was purged with a H_2 balloon for 3 minutes. The reaction was stirred at rt for 2 h 45 min and then filtered through a pad of Celite with EtOAc and concentrated to afford the product as a colorless oil.

Step II: The hemiacetal (1 equiv.) was diluted with DCM (0.23 M) and PCC (2 equiv.) was added in one portion. The reaction was stirred for 30 minutes and then filtered through a plug of silica pad with (EtOAc/hexane, 20%) to afford the product as a colorless oil.

Boivinianin A

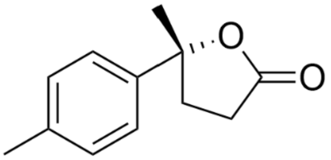

(Quantitative, 14 mg). Colorless oil. ^{1}H NMR (500 MHz, $CDCl_3$): δ 7.24 (d, *J* = 8.2 Hz, 2H), 7.16 (d, *J* = 7.9 Hz, 2H), 2.69 – 2.55 (m, 1H), 2.53 – 2.42 (m, 2H), 2.38 – 2.35 (m, 1H), 2.33 (s, 3H), 1.69 (s, 3H). $^{13}C\{^{1}H\}$NMR (126 MHz, $CDCl_3$): δ 176.6, 141.3, 137.4, 129.2, 124.1, 87.0, 36.2, 29.5, 29.0, 20.9. HRMS (ESI) *m/z* $[M+H]^+$ calcd. for $[C_{12}H_{15}O_2]$ 191.1067, found 191.1069. $[\alpha]_D^{22}$ = +5.0 (c 0.23, $CHCl_3$, 85% ee); lit. $[\alpha]_D^{22}$ = +13.8 (c 0.74, $CHCl_3$).[9]

X. Spectroscopic data (NMR Spectra)

(*E*)-4-phenylpent-2-enal (5a)

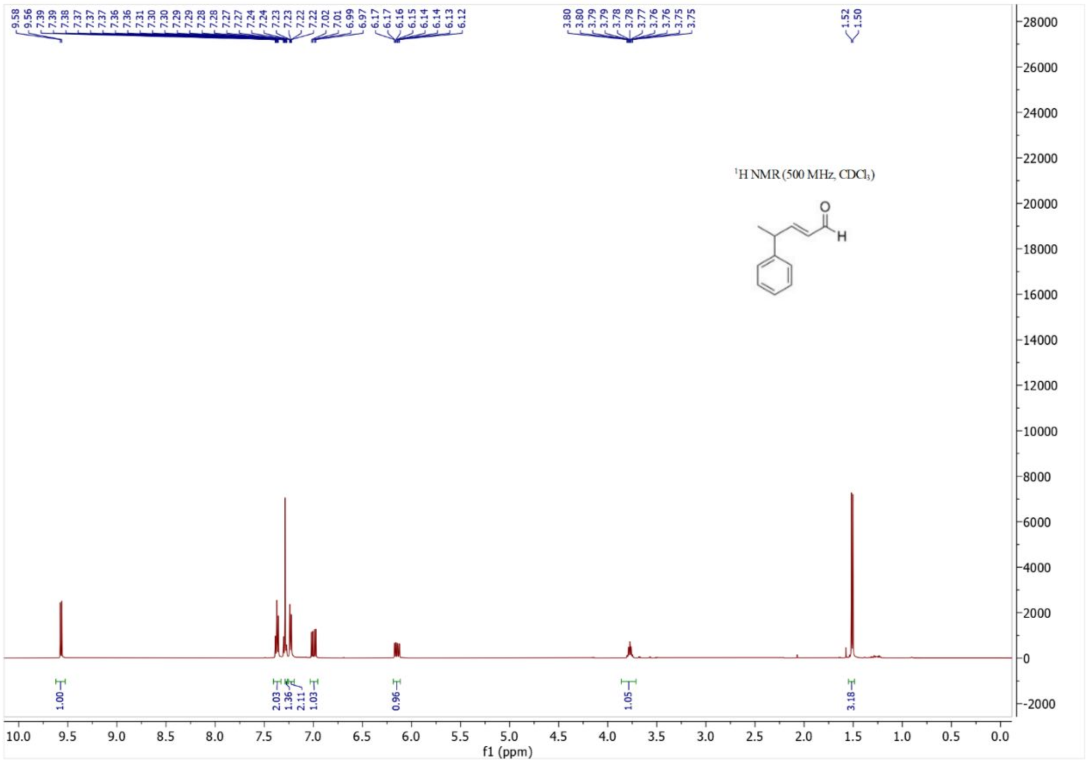

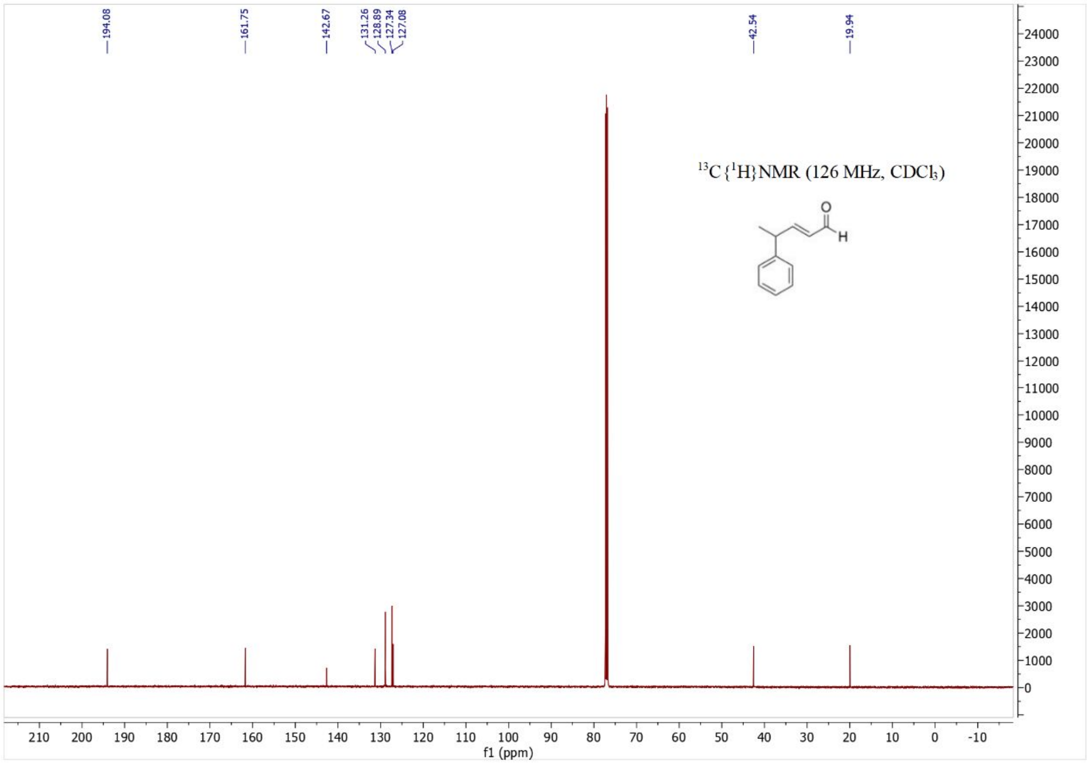

(*E*)-4-(4-fluorophenyl)pent-2-enal (5b)

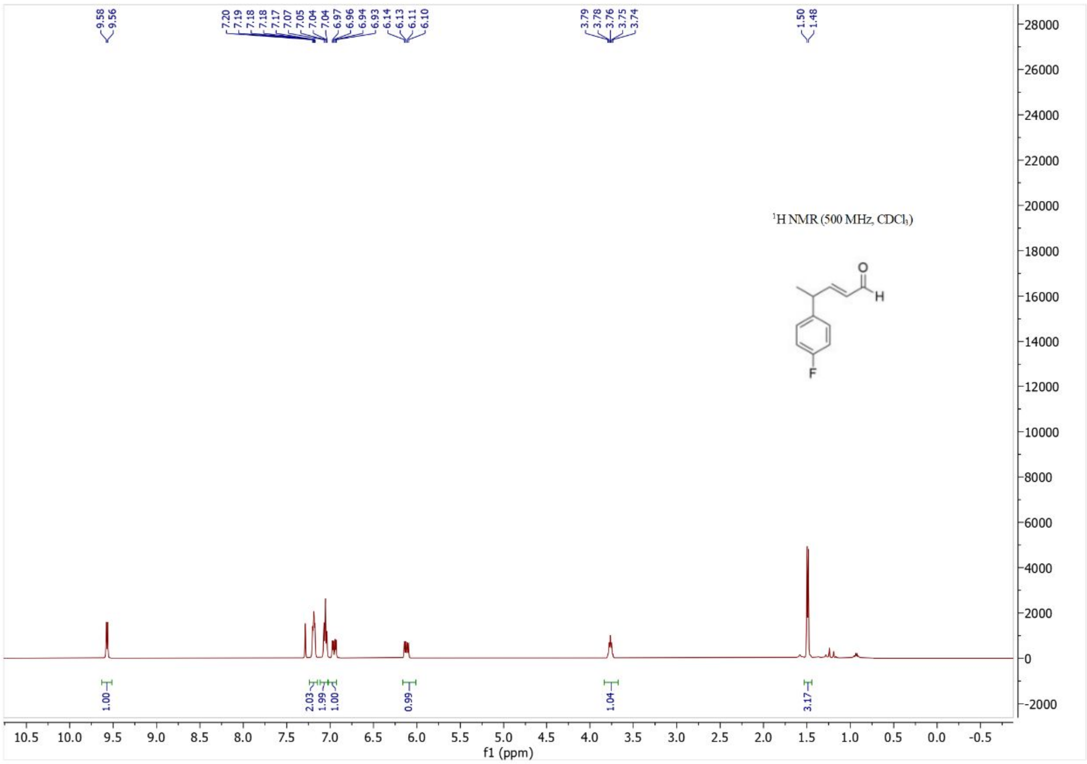

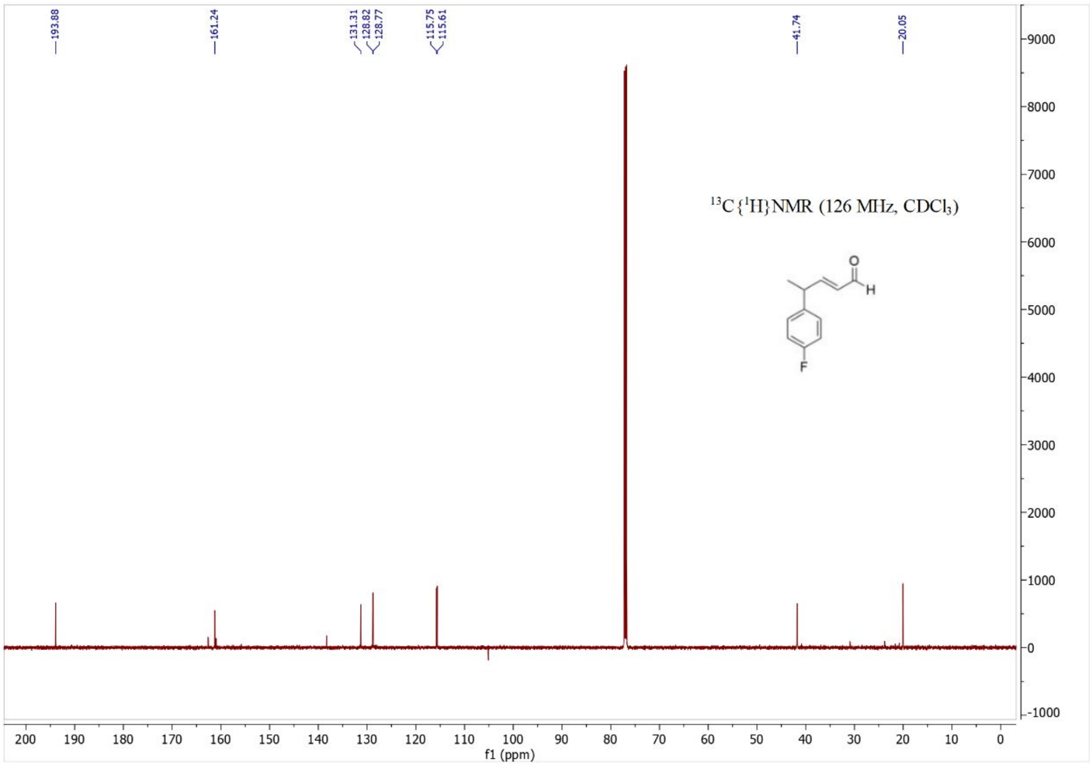
193.88
161.24
131.31
128.82
128.77
115.75
115.61
41.74
20.05
$^{13}C\{^1H\}$NMR (126 MHz, $CDCl_3$)
O
H
F
9000
8000
7000
6000
5000
4000
3000
2000
1000
0
-1000
200 190 180 170 160 150 140 130 120 110 100 90 80 70 60 50 40 30 20 10 0
f1 (ppm)

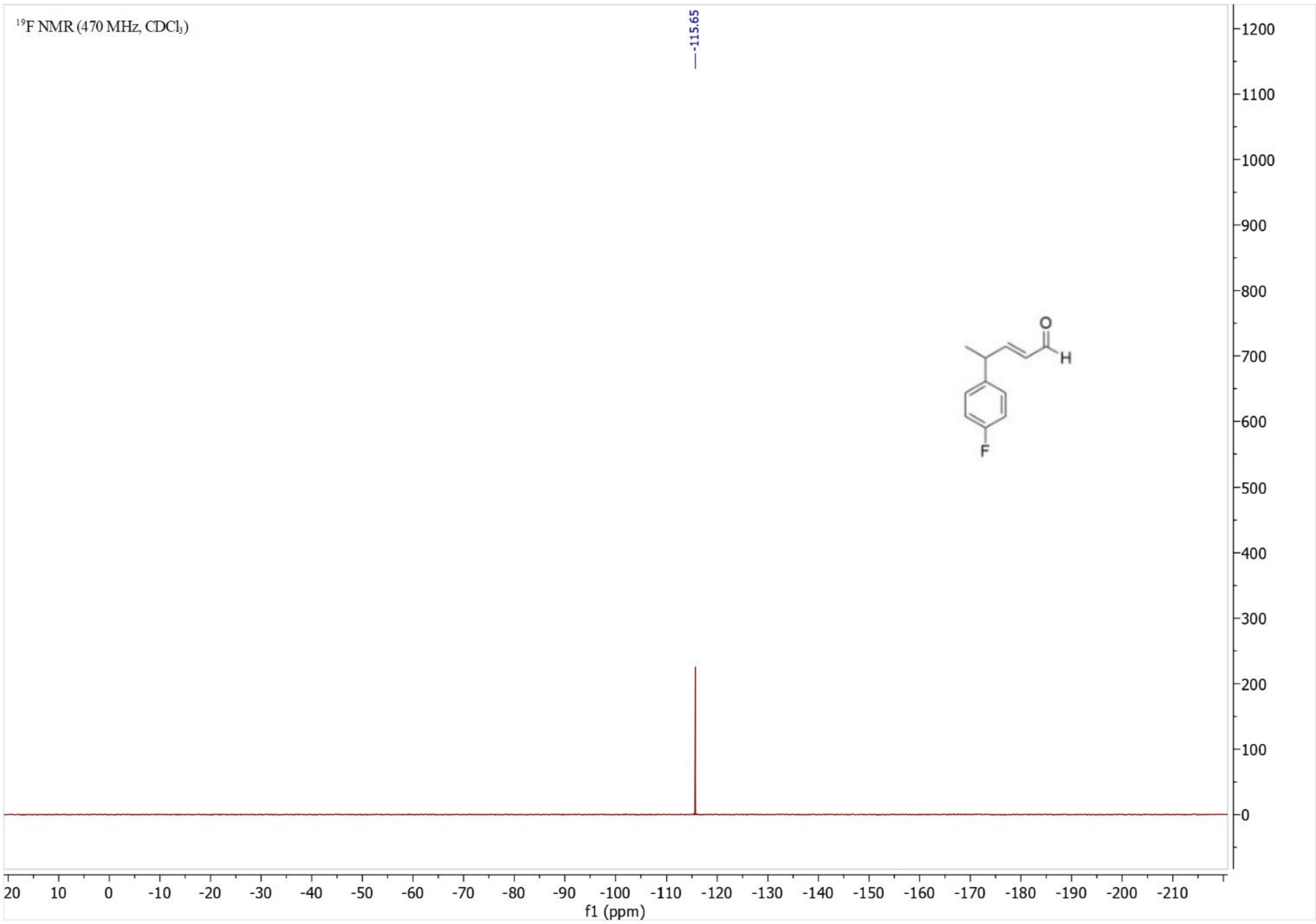

^{19}F NMR (470 MHz, $CDCl_3$)
-115.65
O
H
F
1200
1100
1000
900
800
700
600
500
400
300
200
100
0
20 10 0 -10 -20 -30 -40 -50 -60 -70 -80 -90 -100 -110 -120 -130 -140 -150 -160 -170 -180 -190 -200 -210
f1 (ppm)

(*E*)-4-(p-tolyl)pent-2-enal (5c)

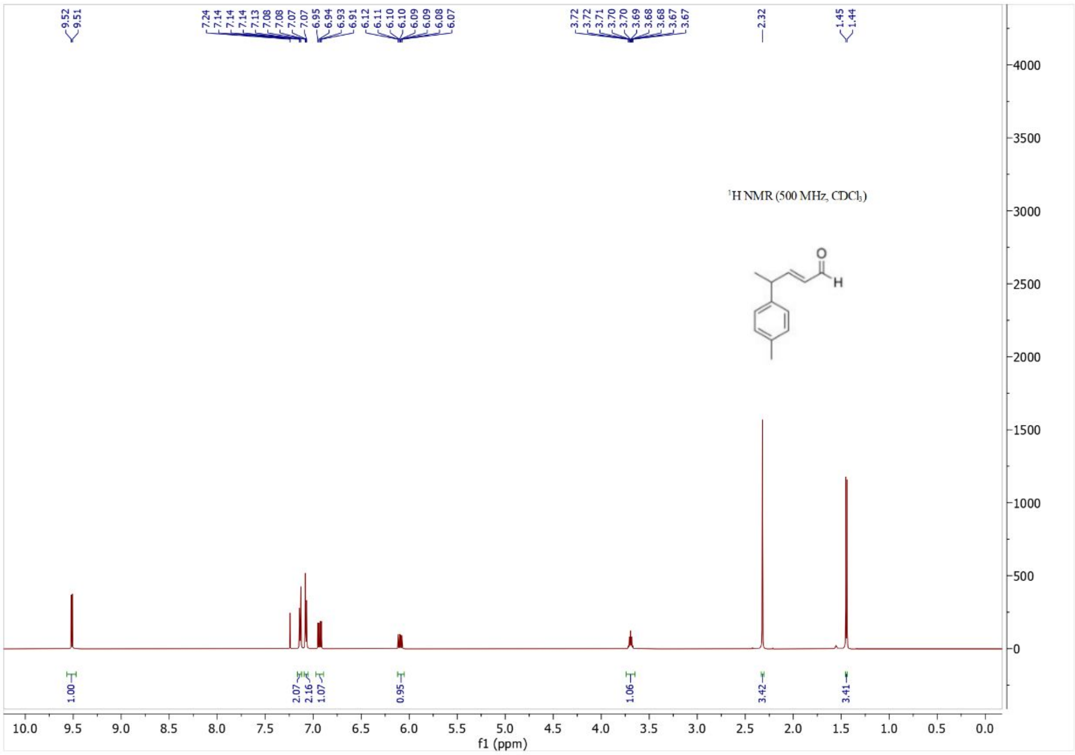

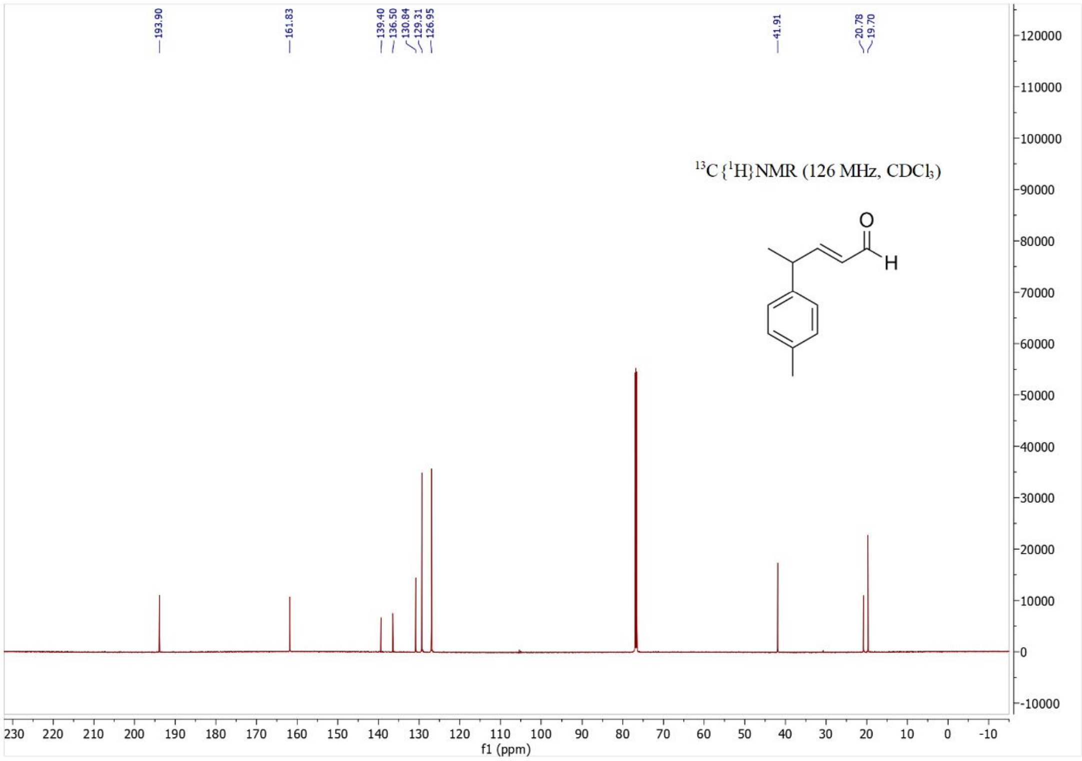

(*E*)-4-(4-methoxyphenyl)pent-2-enal (5d)

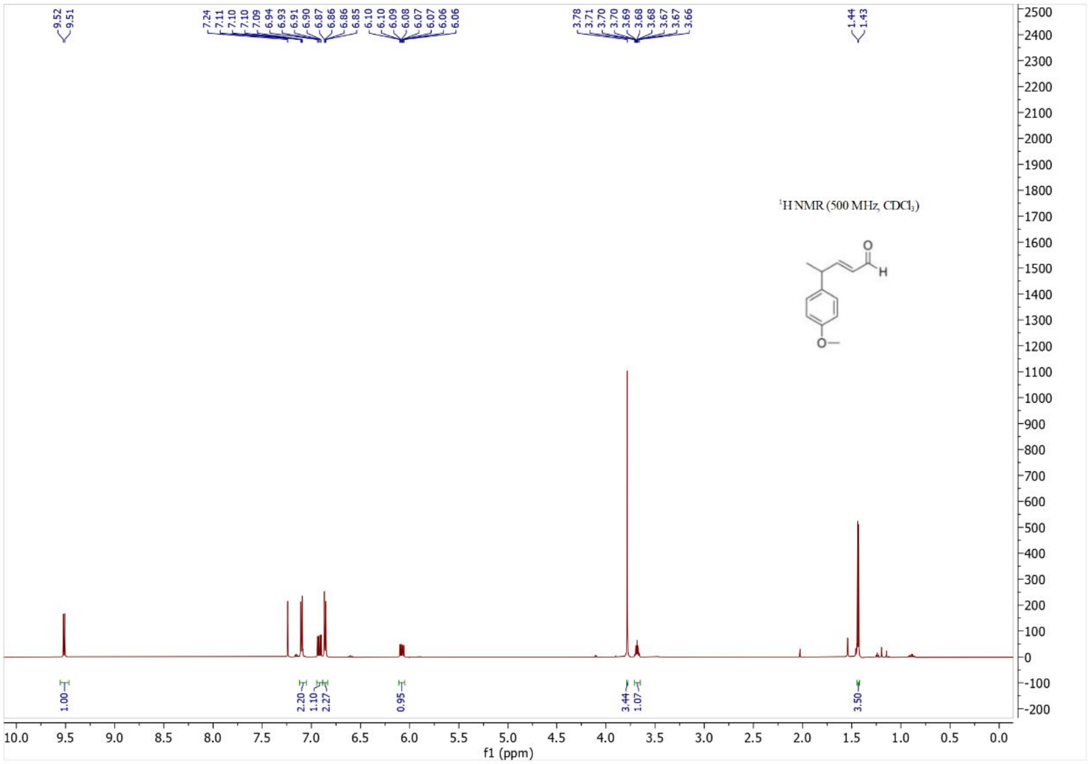

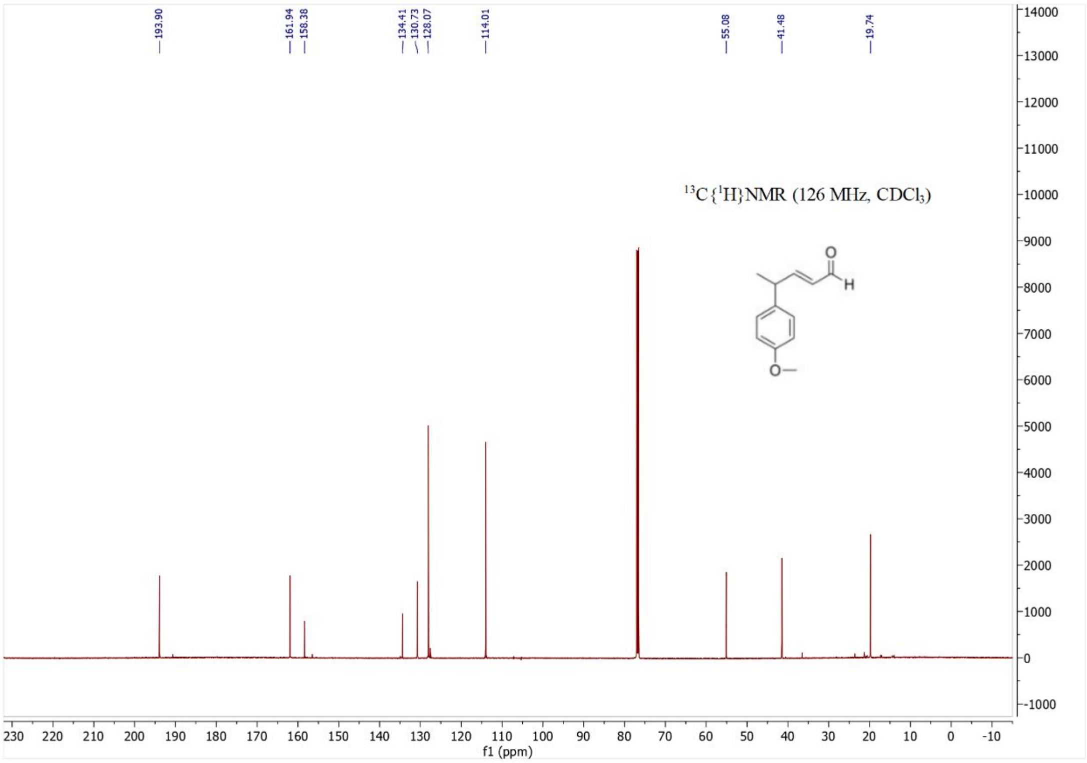

(*E*)-4-(4-(trifluoromethyl)phenyl)pent-2-enal (5e)

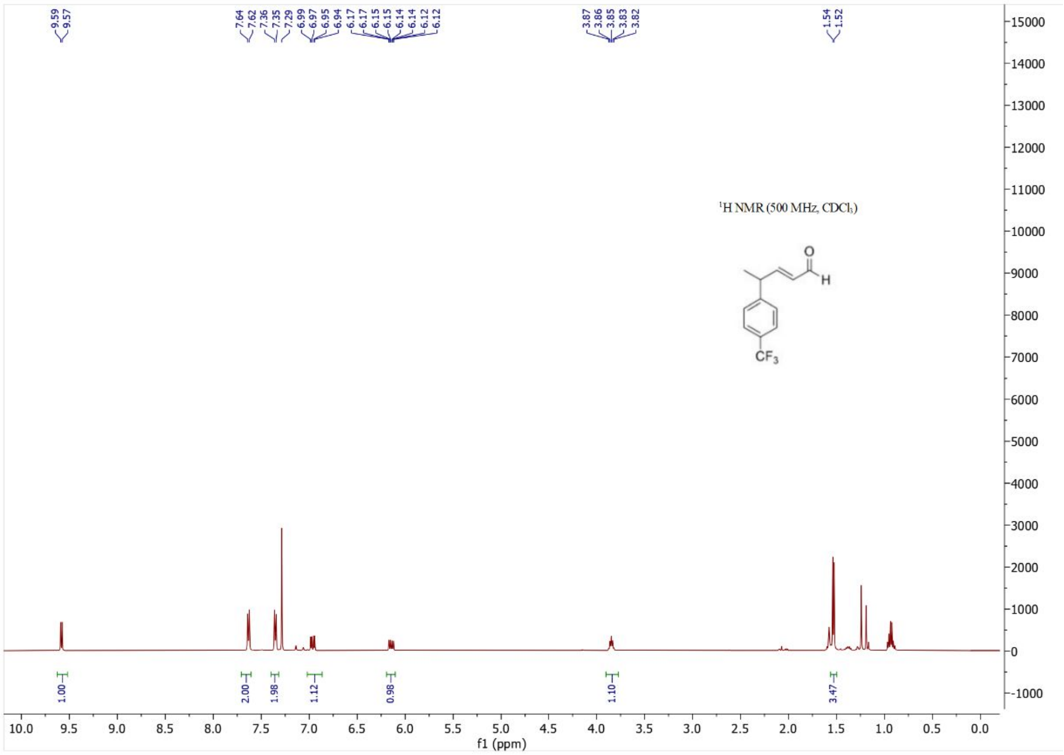

9.59
9.57
7.64
7.62
7.36
7.35
7.29
6.99
6.97
6.95
6.94
6.17
6.17
6.15
6.15
6.14
6.14
6.12
6.12
3.87
3.86
3.85
3.83
3.82
1.54
1.52
^{1}H NMR (500 MHz, CDCl$_3$)
O
H
CF$_3$
1.00
2.00
1.98
1.12
0.98
1.10
3.47
f1 (ppm)

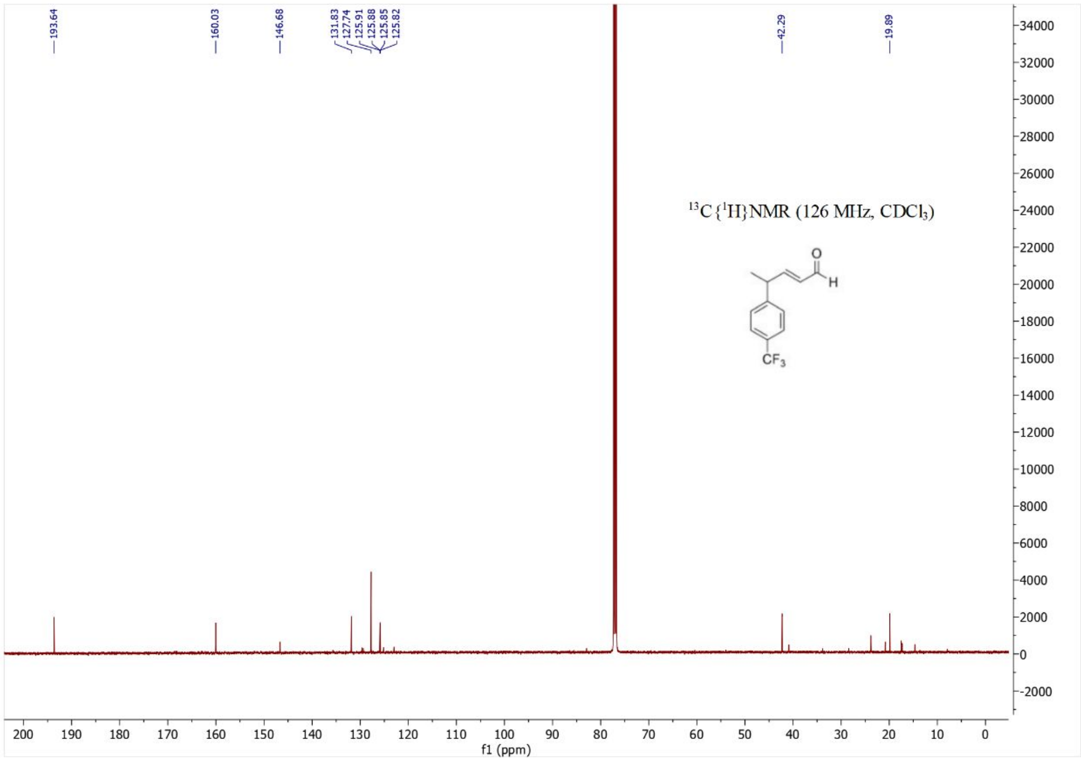

193.64
160.03
146.68
131.83
127.74
125.91
125.88
125.85
125.82
42.29
19.89
^{13}C{^{1}H}NMR (126 MHz, CDCl$_3$)
O
H
CF$_3$
f1 (ppm)

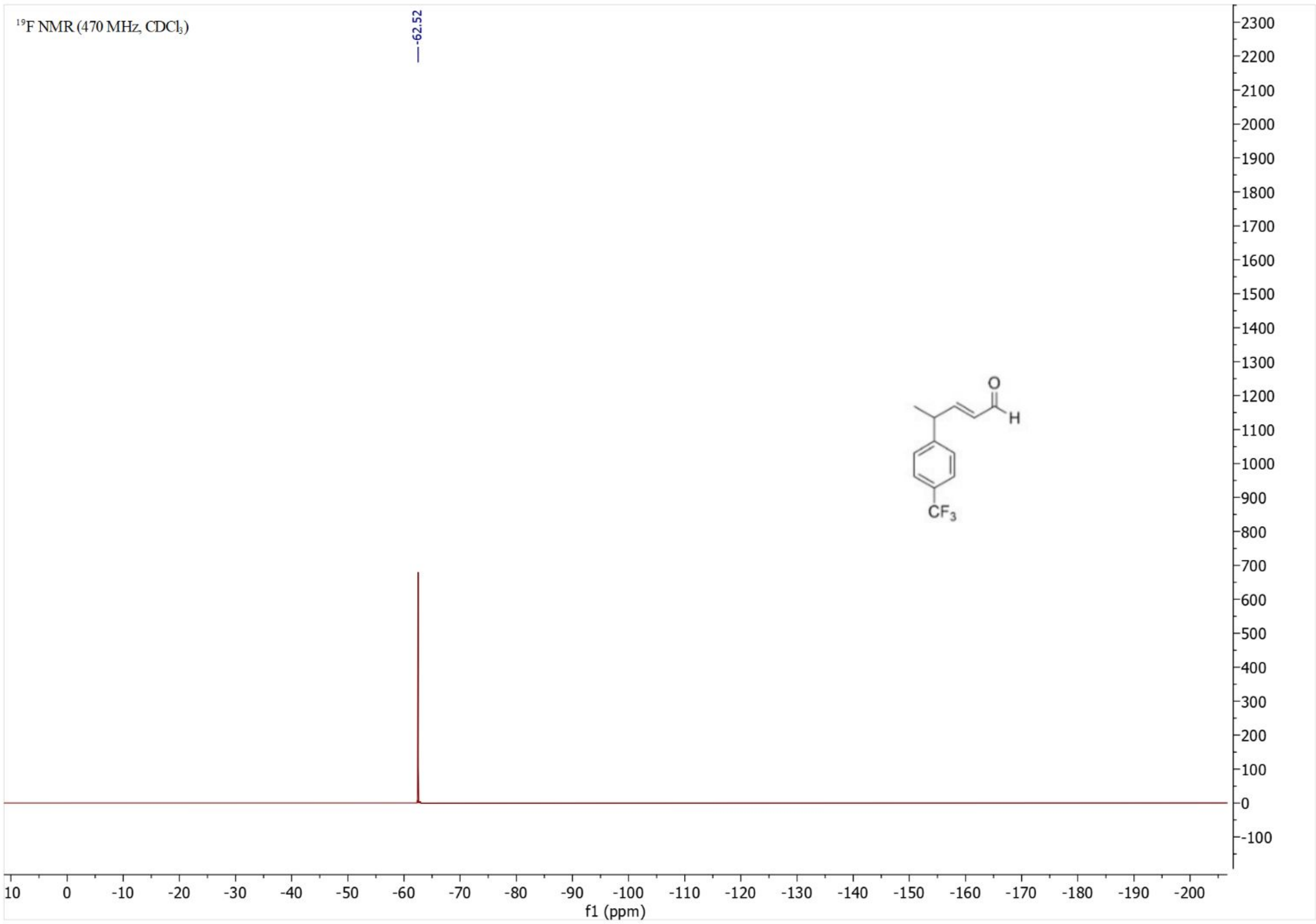

19F NMR (470 MHz, CDCl3)
-62.52
CF3
H
O
f1 (ppm)

(*E*)-4-(4-nitrophenyl)pent-2-enal (5f)

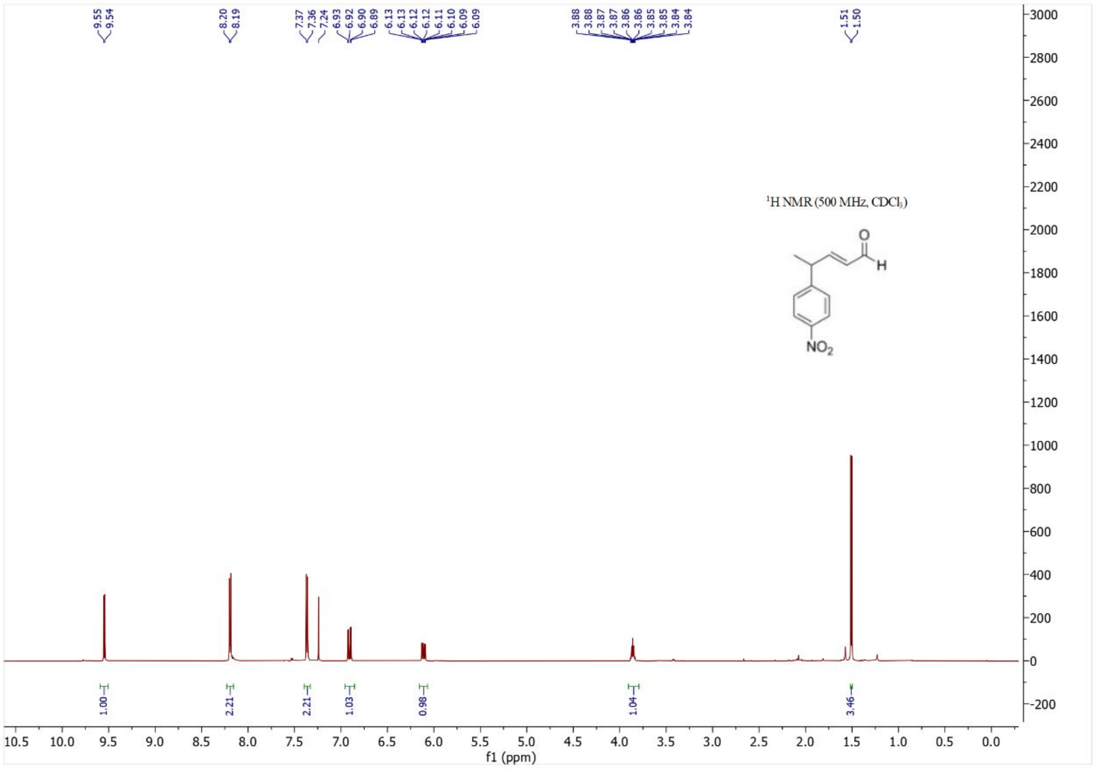

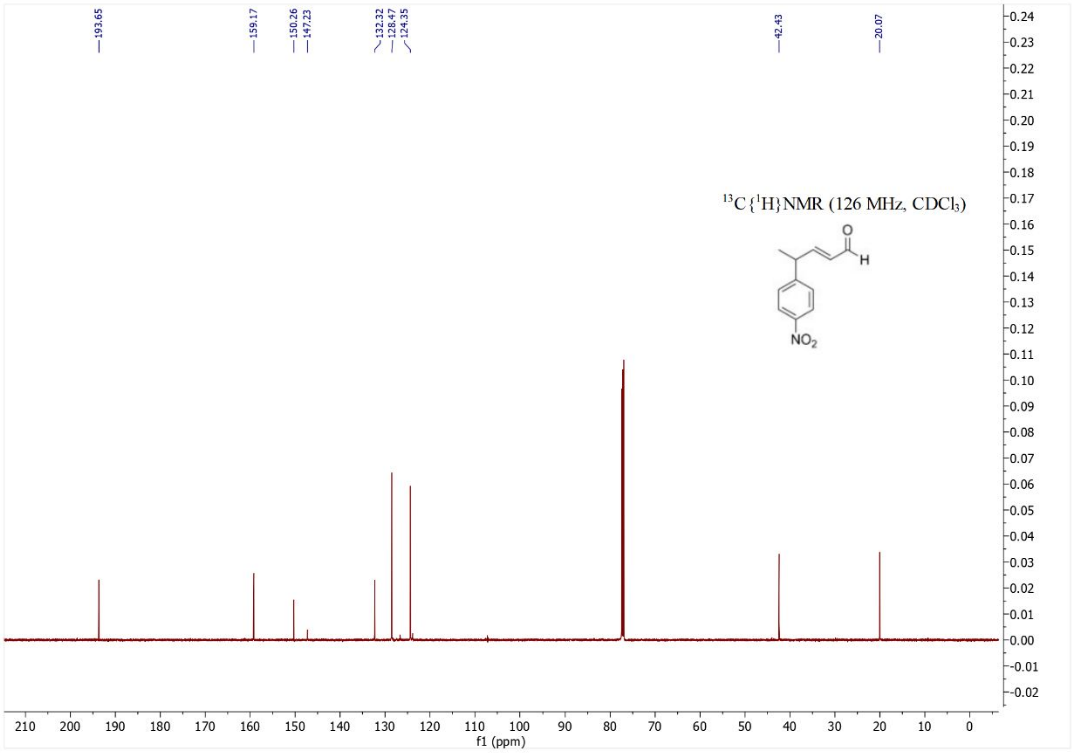

(*E*)-4-(3-(trifluoromethyl)phenyl)pent-2-enal (5g)

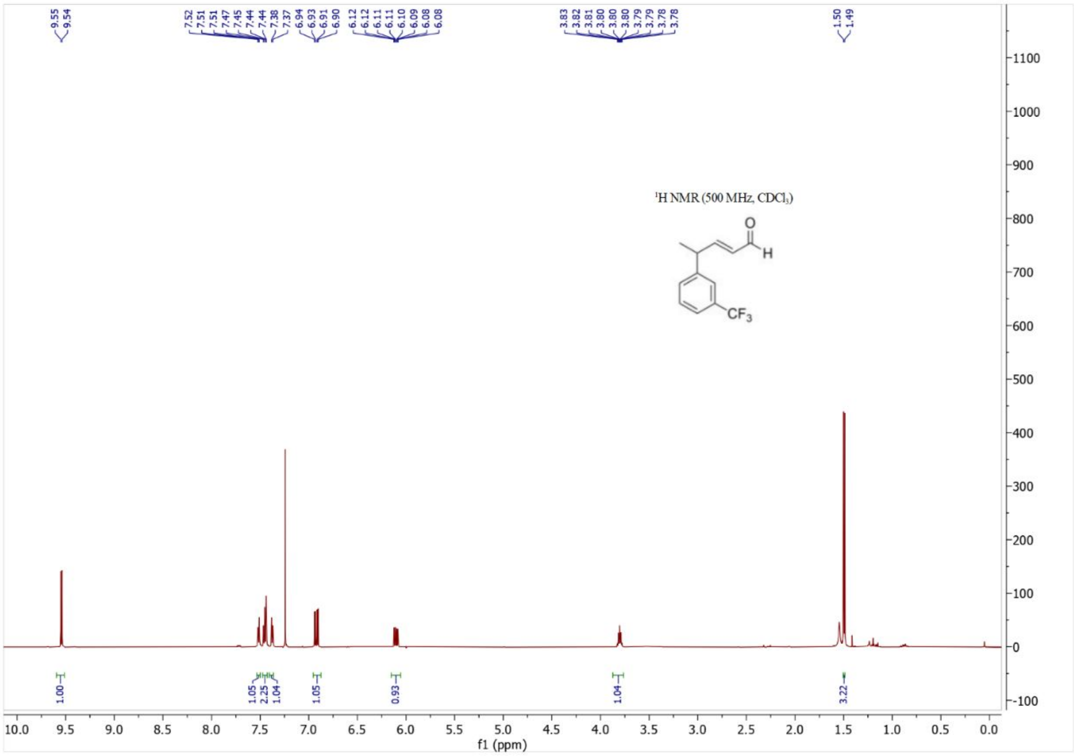

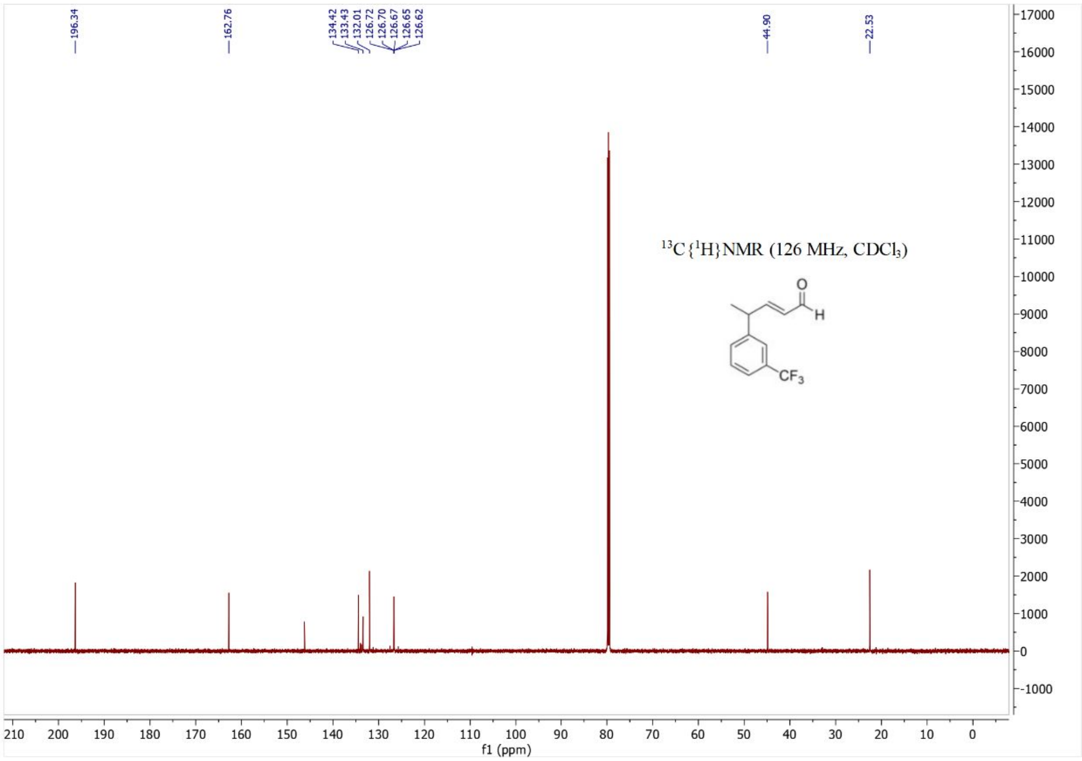
196.34
162.76
134.42
133.43
132.01
126.72
126.70
126.67
126.65
126.62
44.90
22.53
$^{13}C\{^1H\}$NMR (126 MHz, $CDCl_3$)
O
H
CF_3
f1 (ppm)

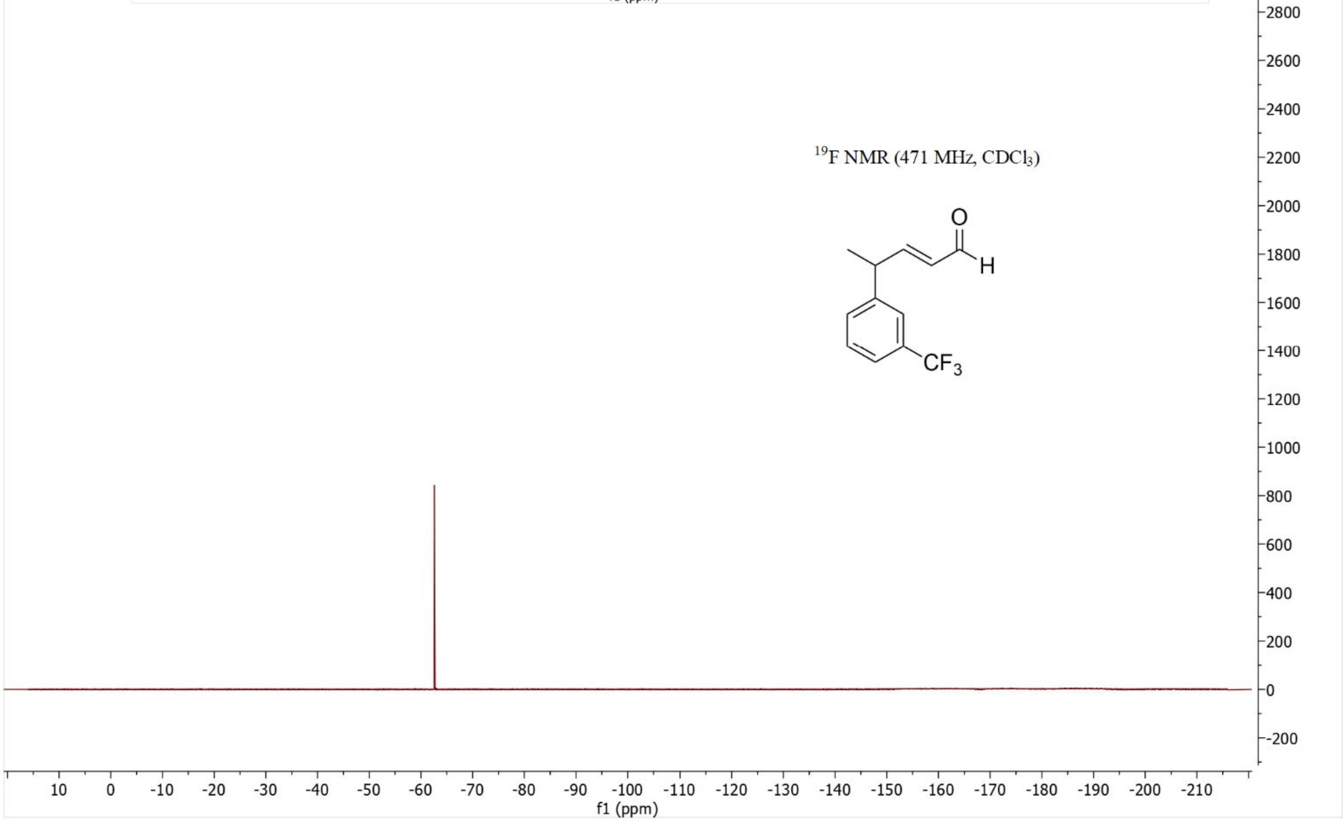
^{19}F NMR (471 MHz, $CDCl_3$)
O
H
CF_3
f1 (ppm)

(*E*)-4-(3-bromophenyl)pent-2-enal (5h)

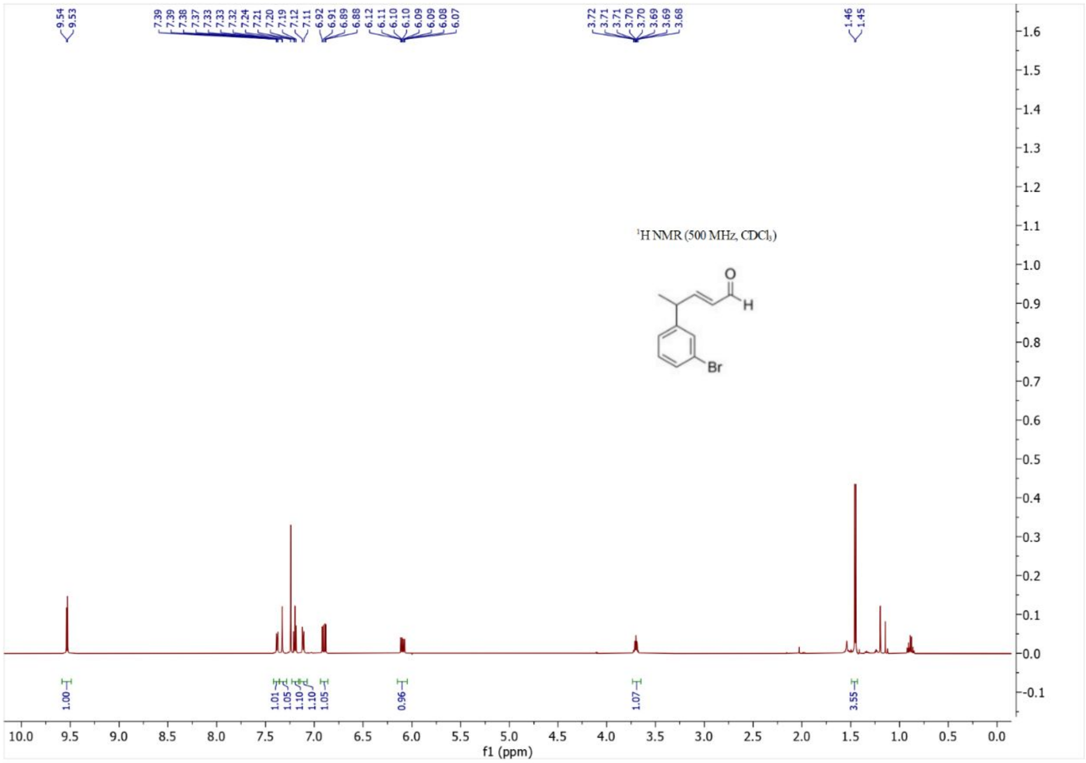

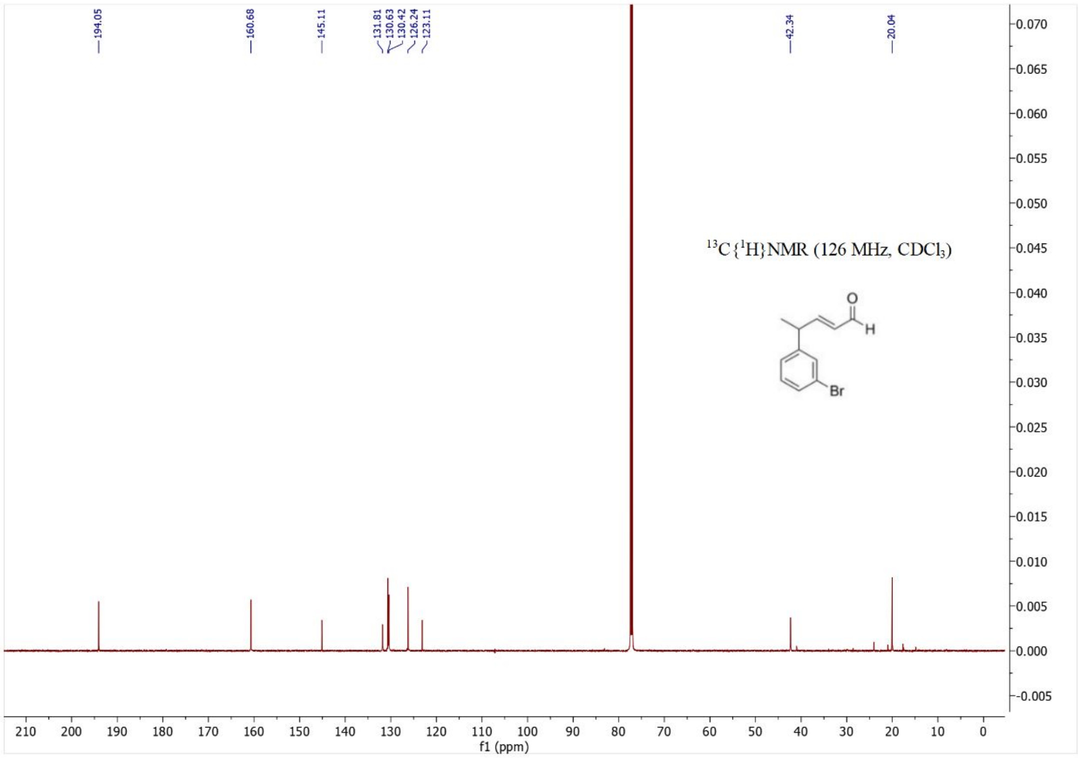

(*E*)-4-(4-isobutylphenyl)pent-2-enal (5i)

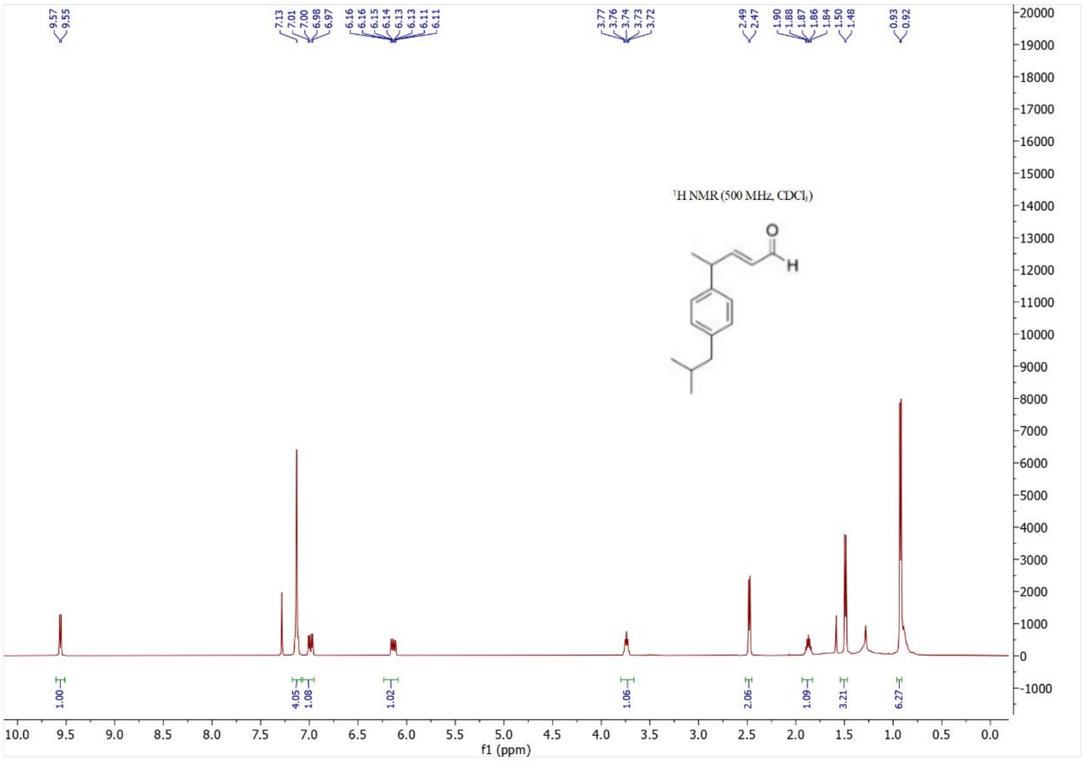

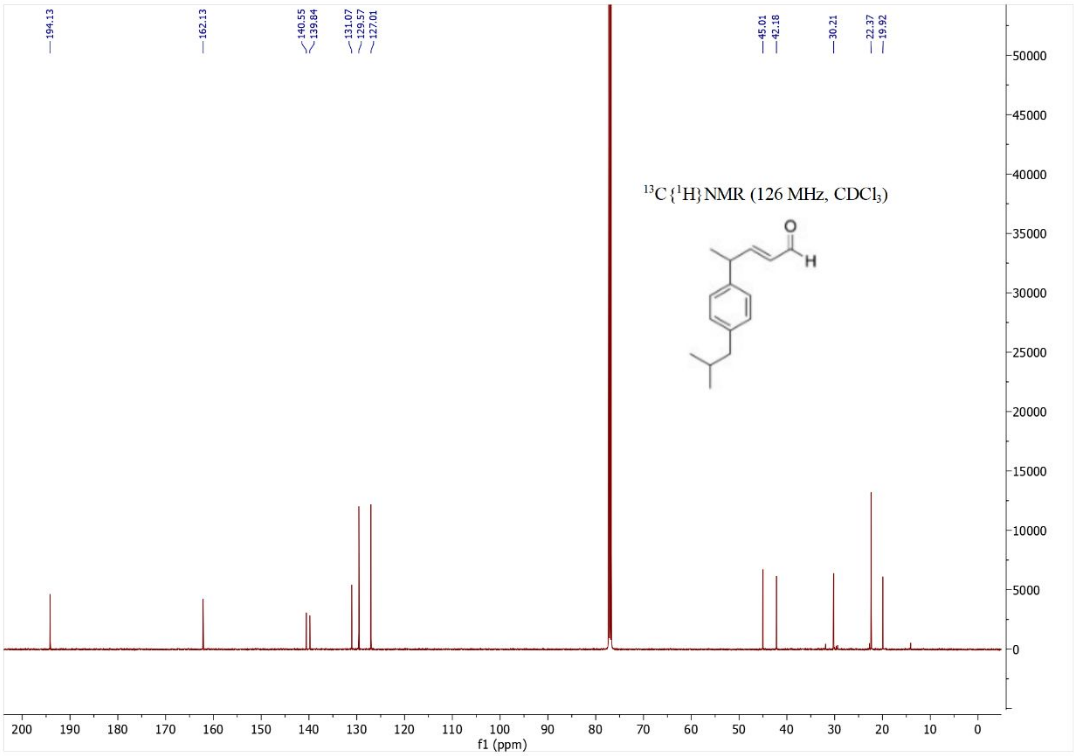

(*E*)-4-(naphthalen-2-yl)pent-2-enal (5j)

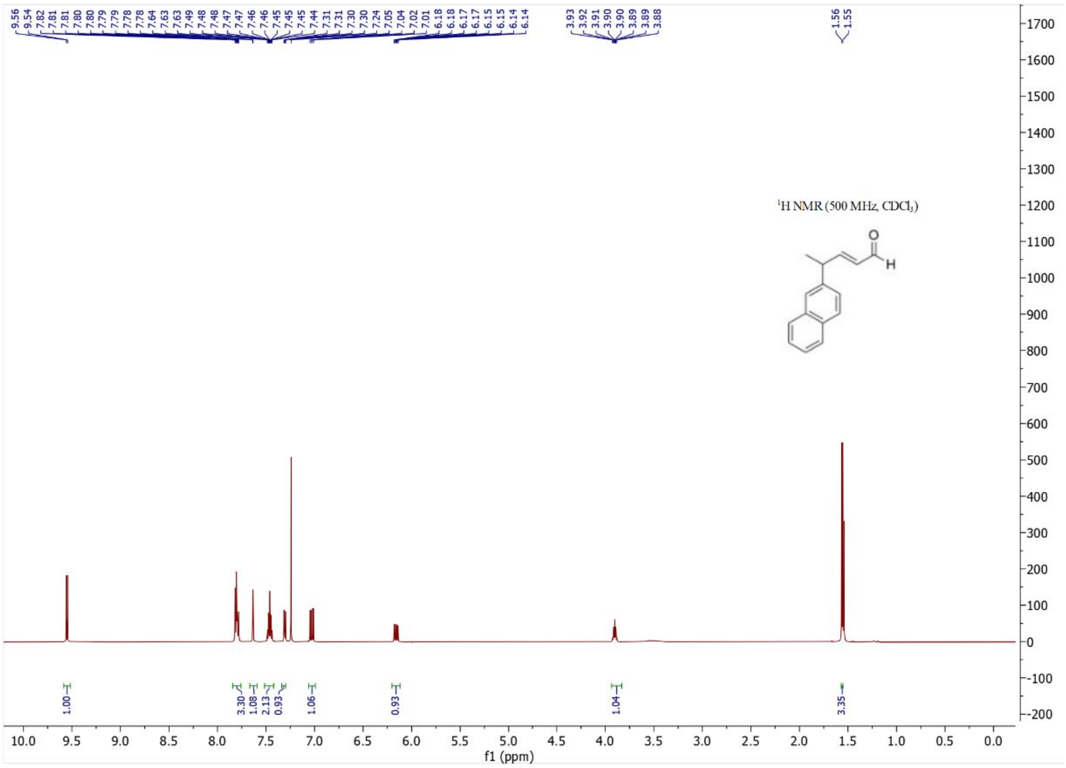

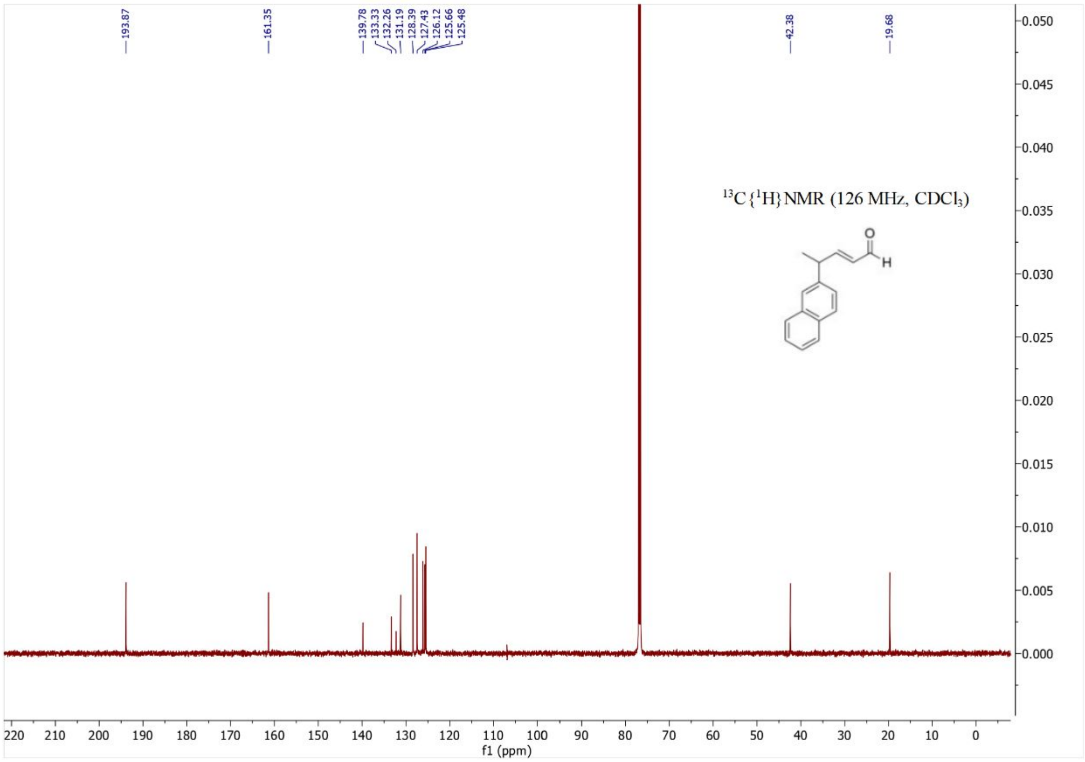

(*E*)-4-(thiophen-3-yl)pent-2-enal (5k)

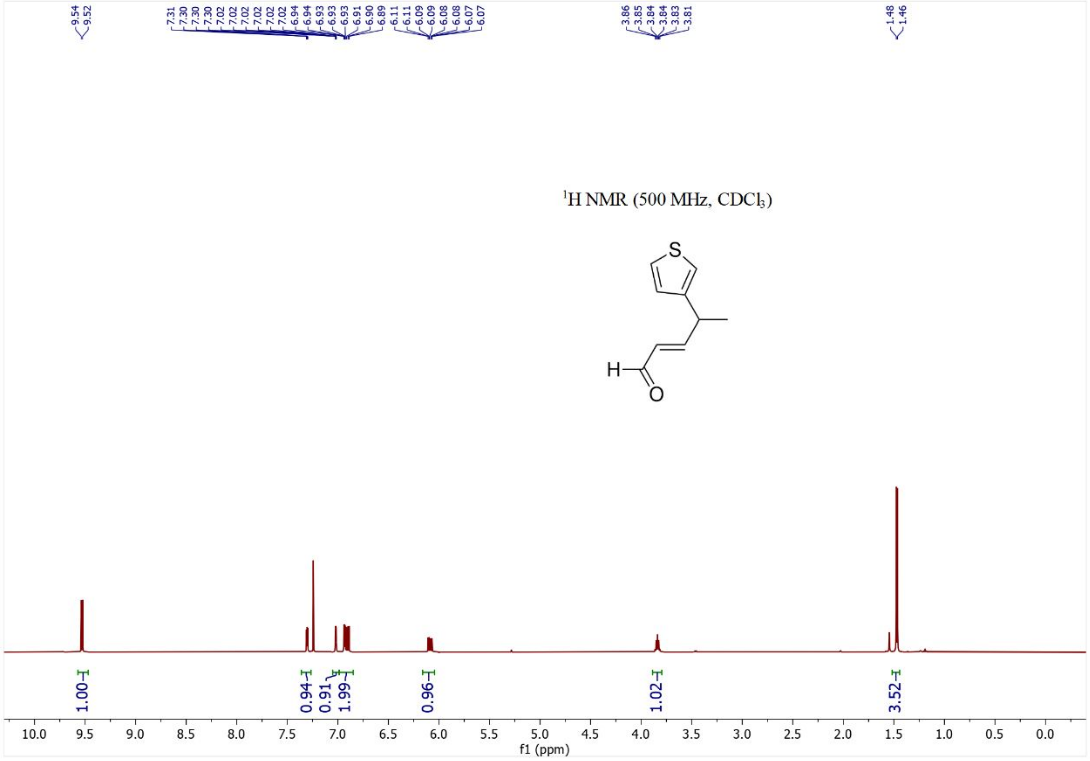

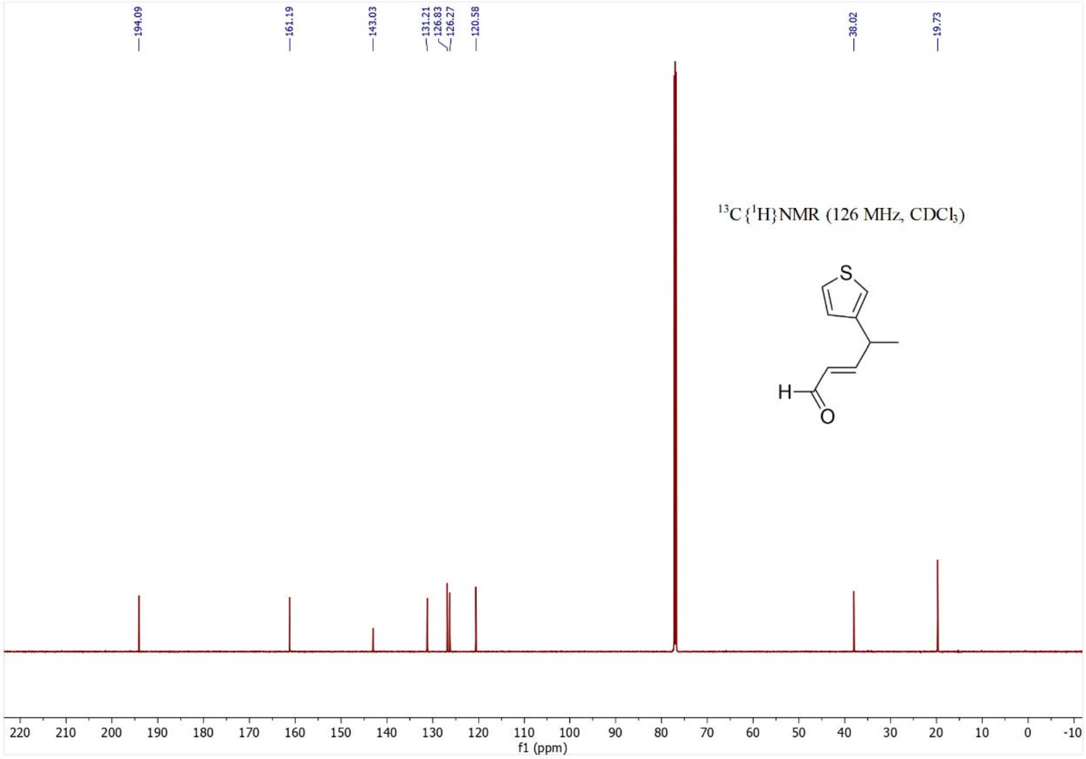

(*E*)-4-((3*S*,10*R*,13*S*)-3-((tert-butyldimethylsilyl)oxy)-10,13-dimethyl-2,3,4,7,8,9,10,11,12,13,14,15-dodecahydro-1H-cyclopenta[a]phenanthren-17-yl)pent-2-enal (5l)

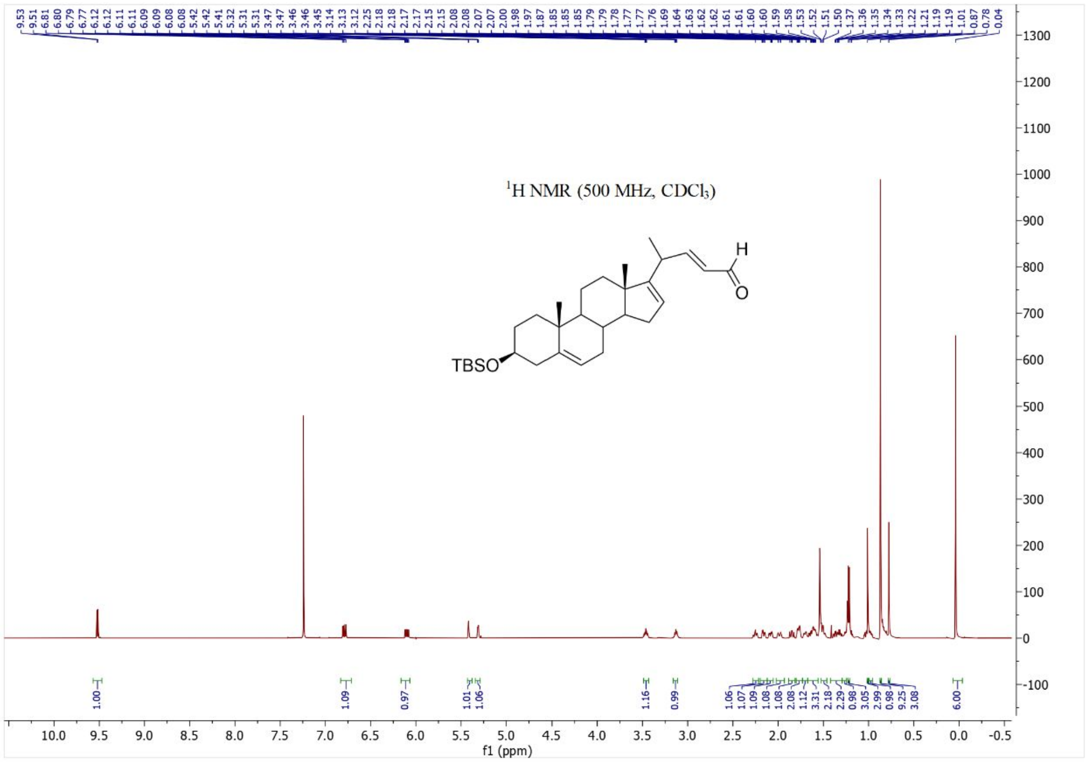

^{1}H NMR (500 MHz, CDCl$_3$)
TBSO
f1 (ppm)

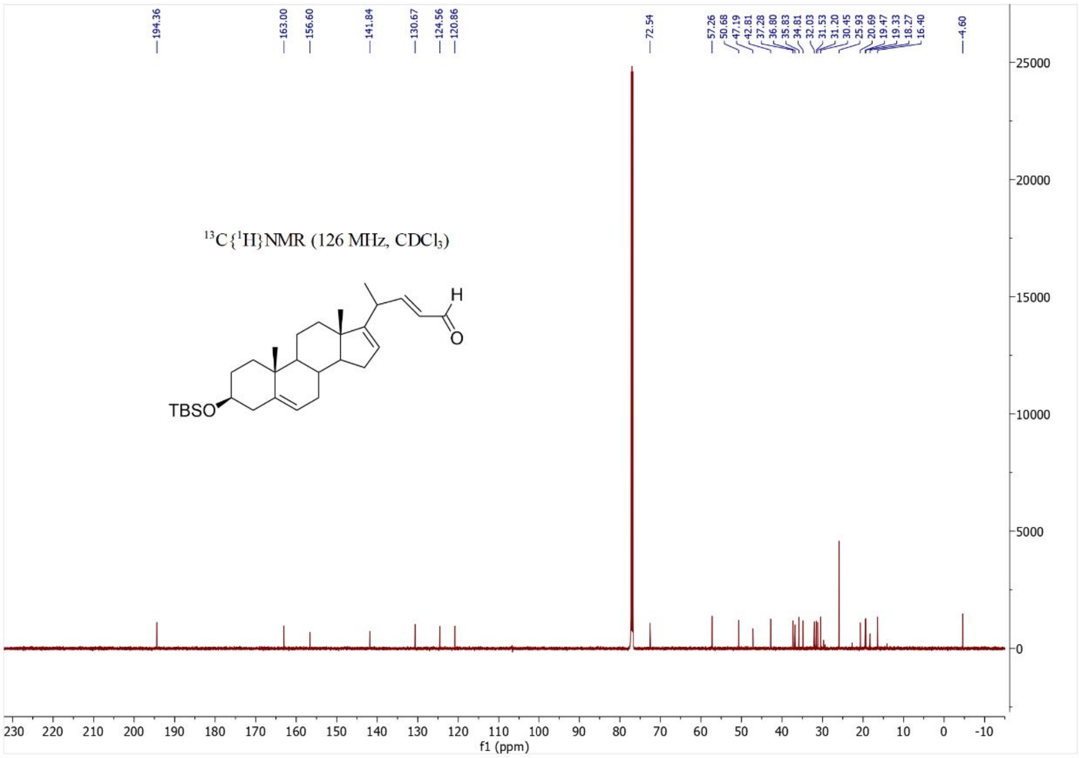

^{13}C{^{1}H}NMR (126 MHz, CDCl$_3$)
TBSO
f1 (ppm)

(*E*)-3-(chroman-4-yl)acrylaldehyde (5m)

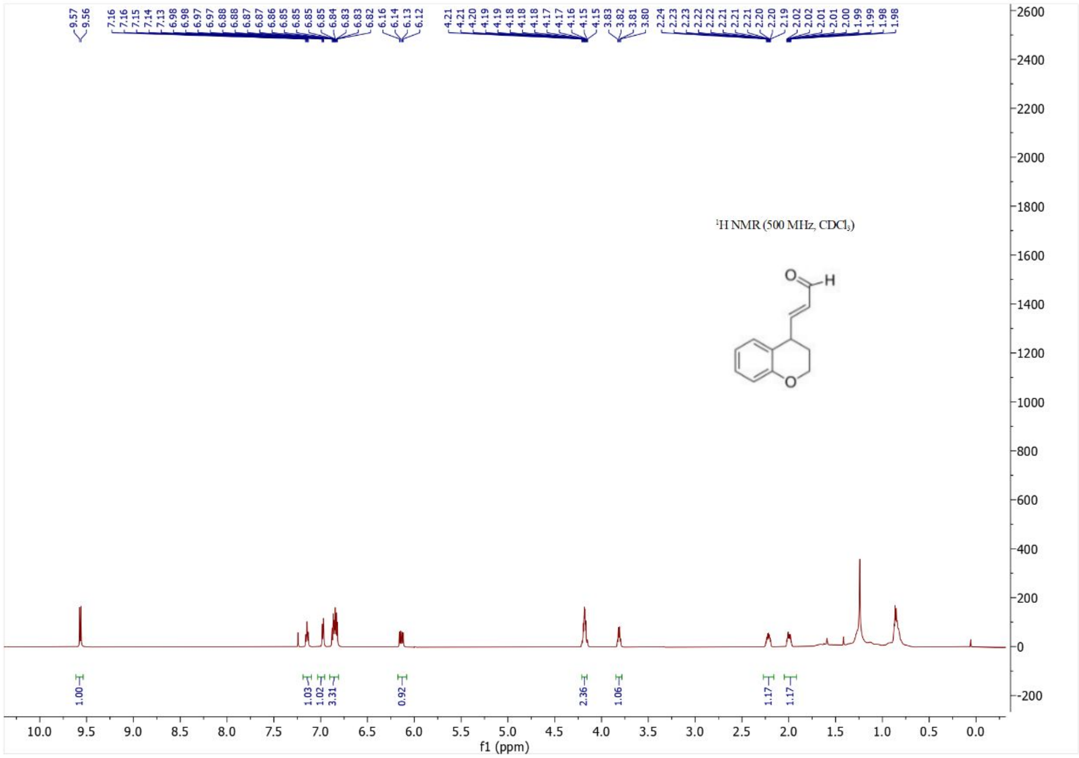

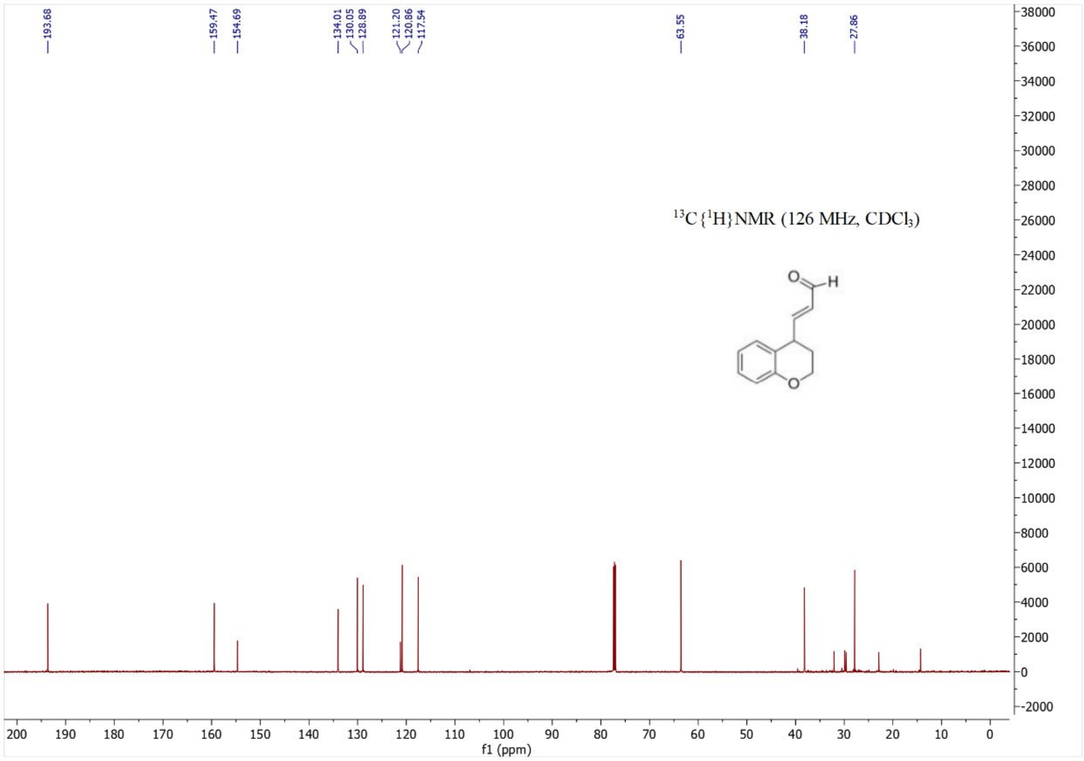

(*E*)-4-phenylhex-2-enal (5n)

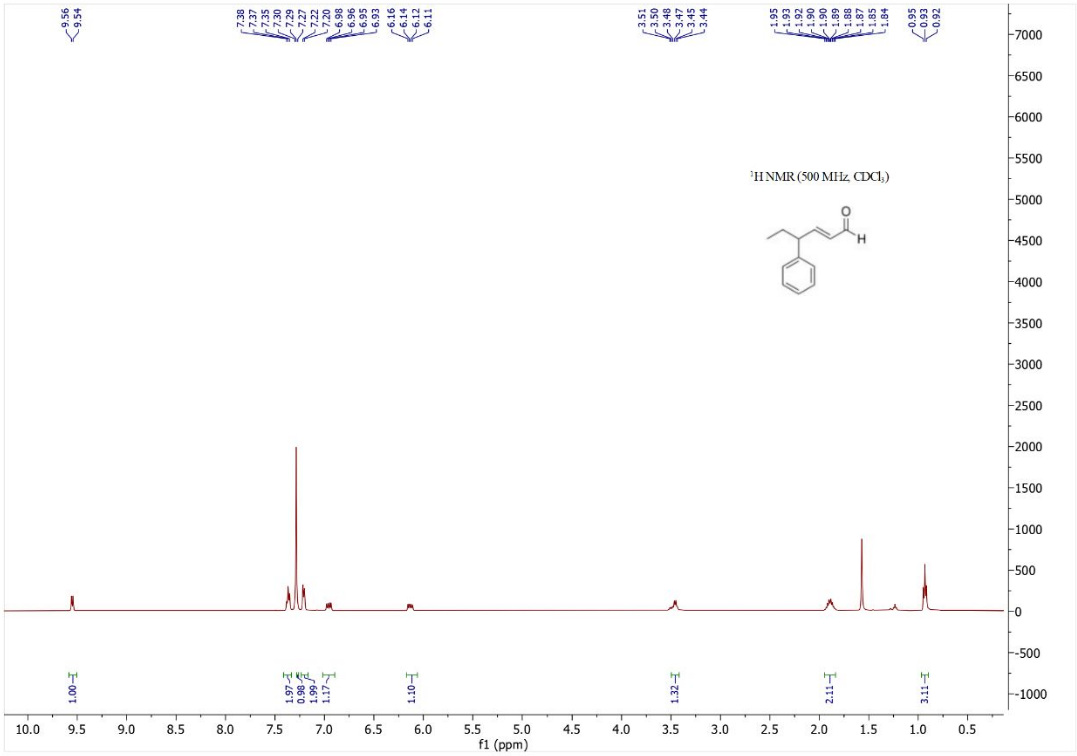

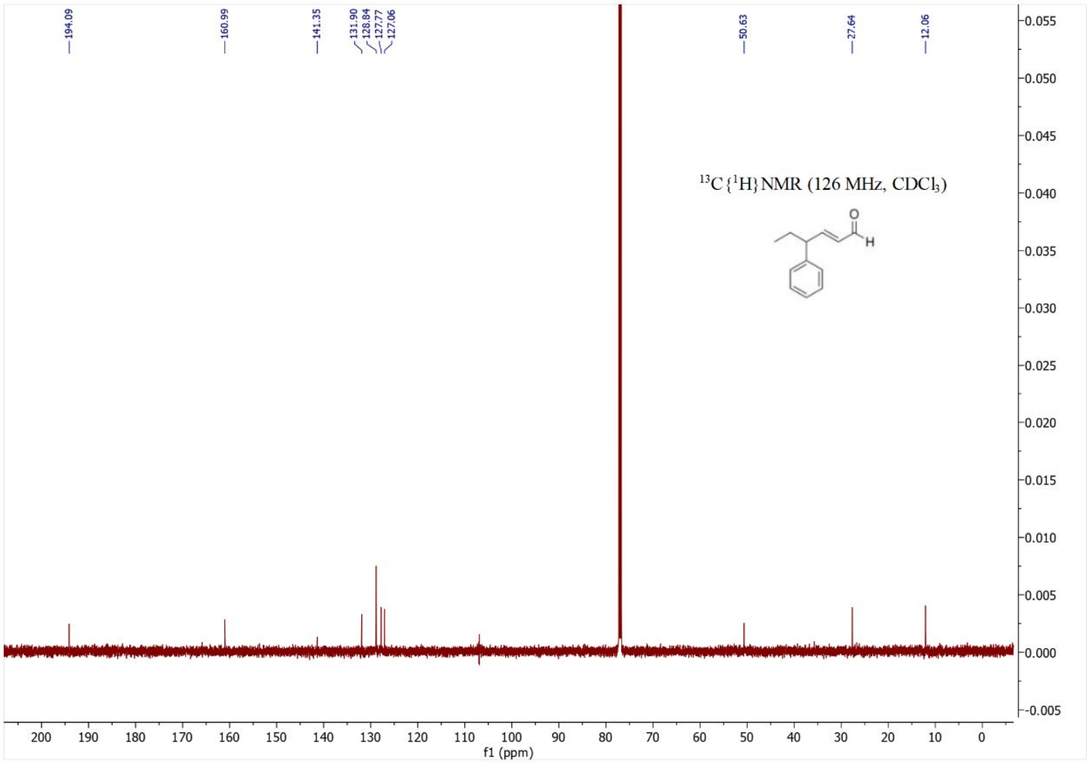

(*E*)-4-(o-tolyl)pent-2-enal (A)

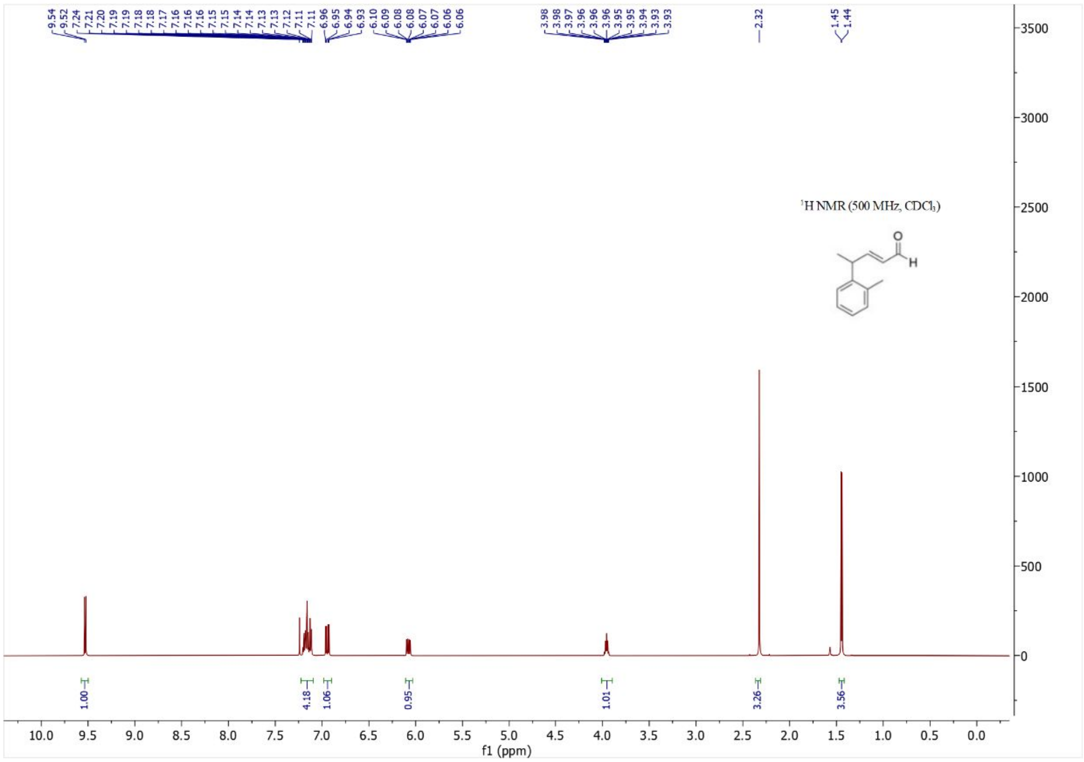

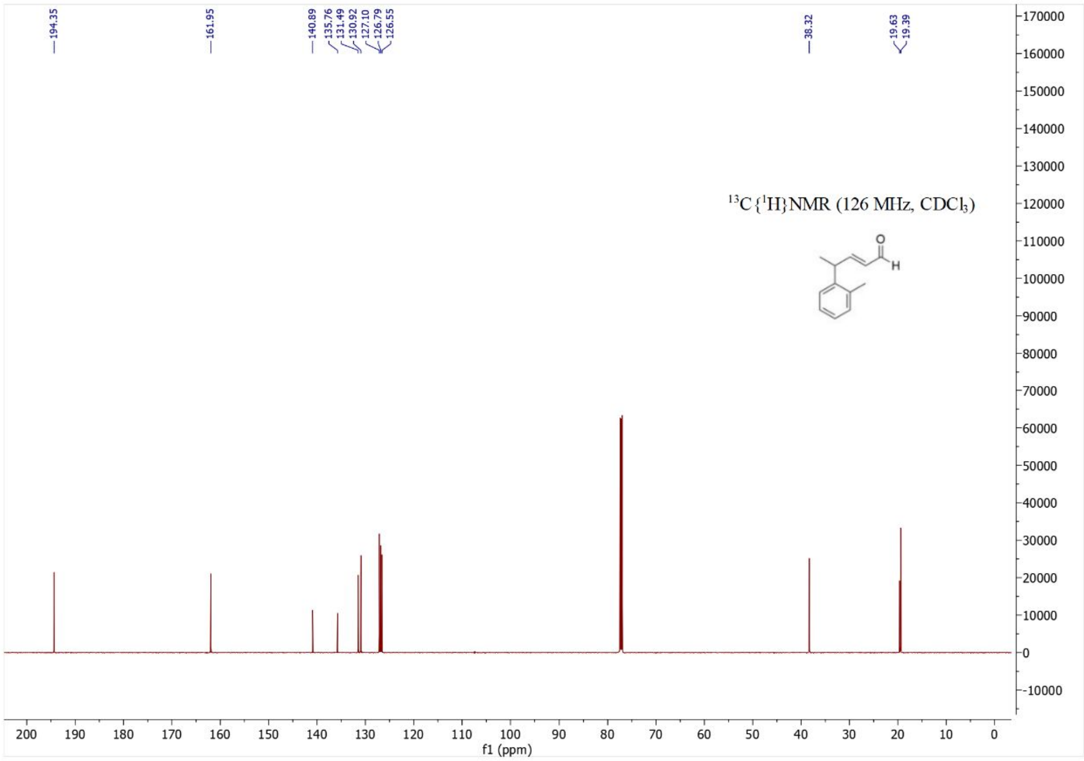

(*E*)-4-(naphthalen-1-yl)pent-2-enal (B)

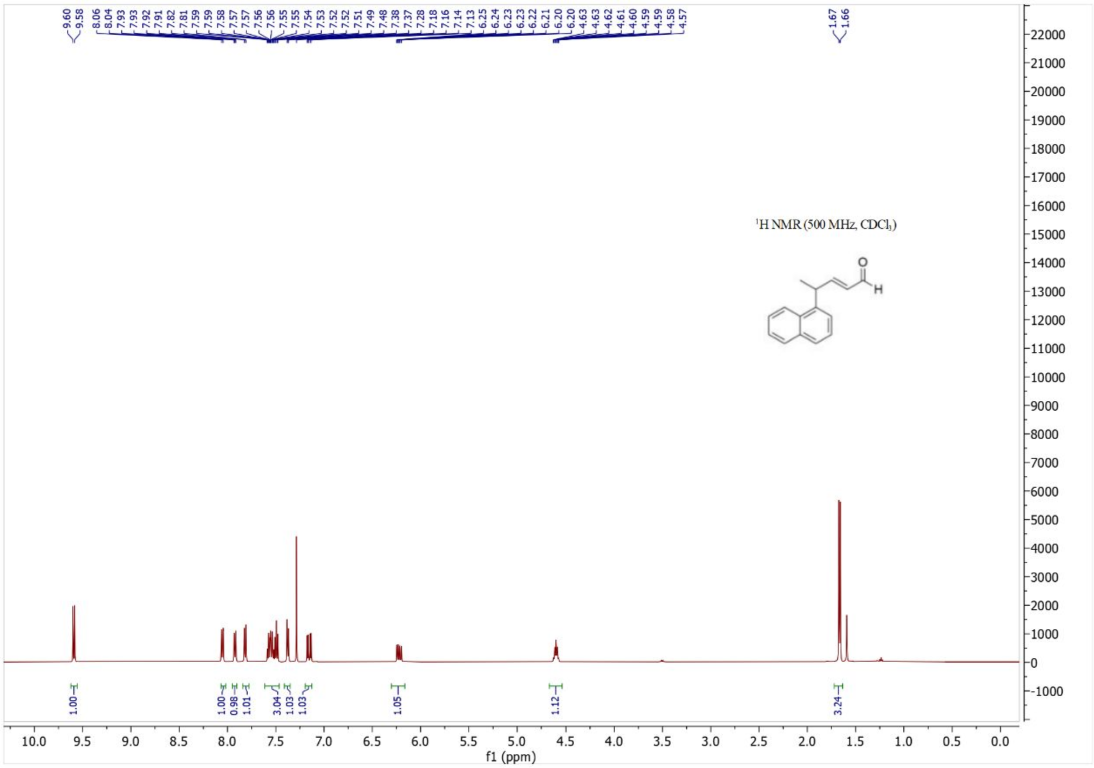

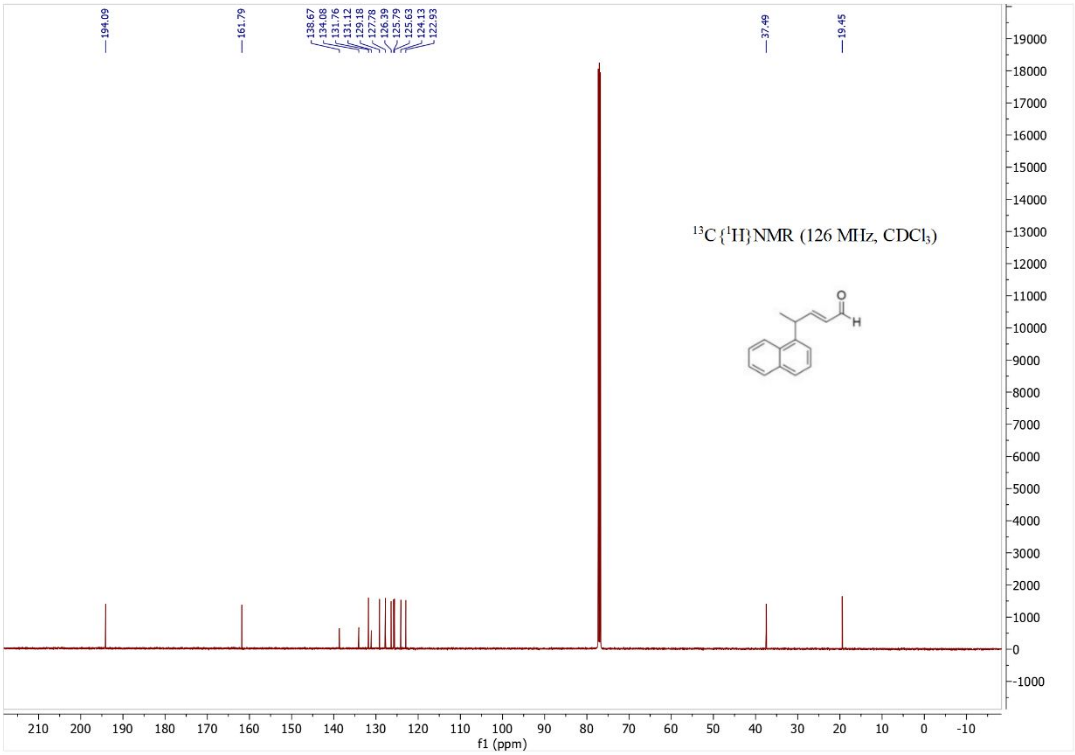

(*E*)-4-methyl-5-phenylpent-2-enal (C)

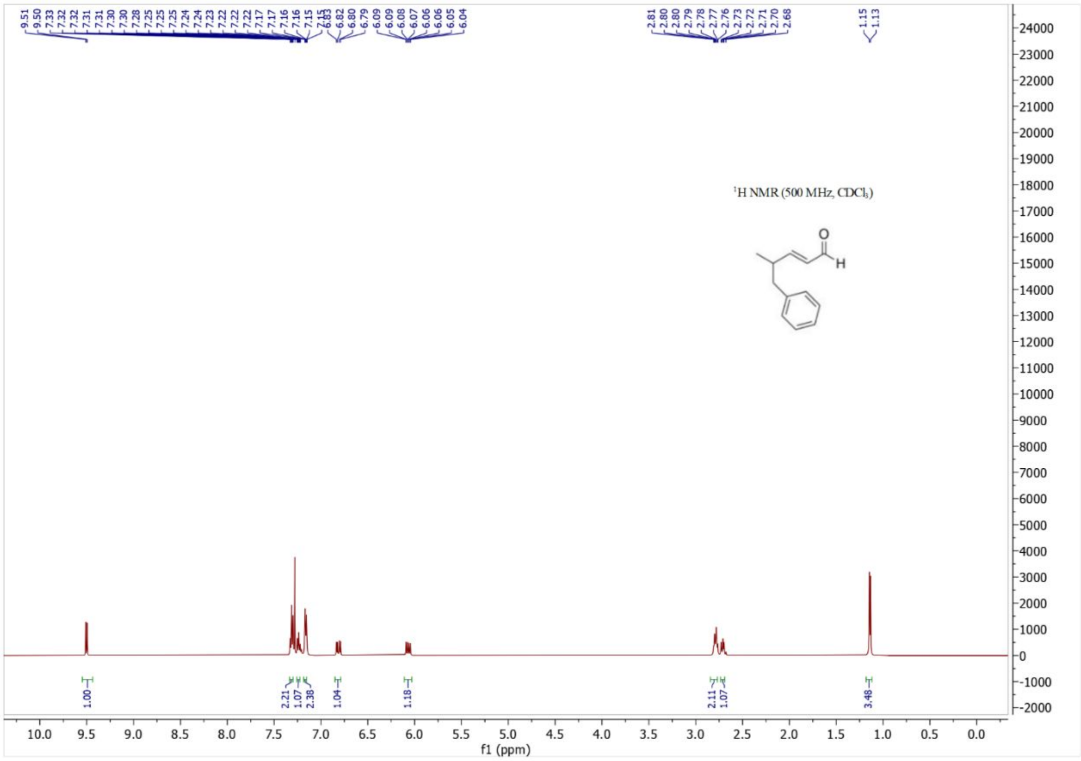

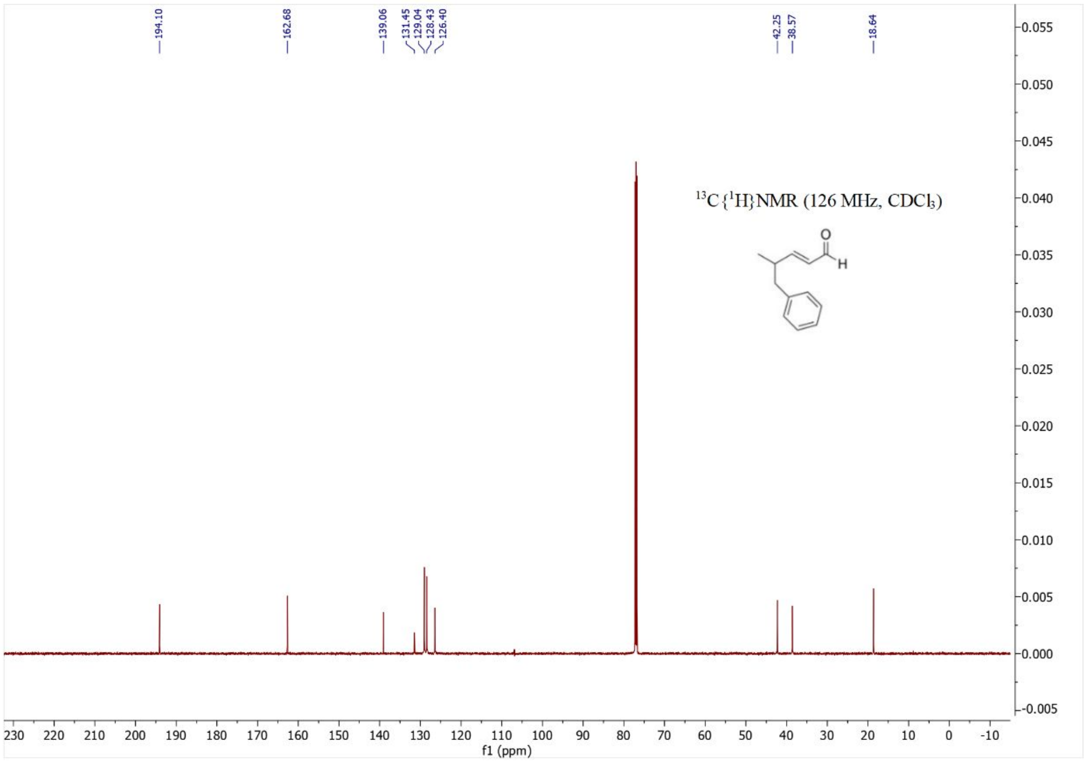

(*E*)-5-phenylhex-3-en-2-one (E)

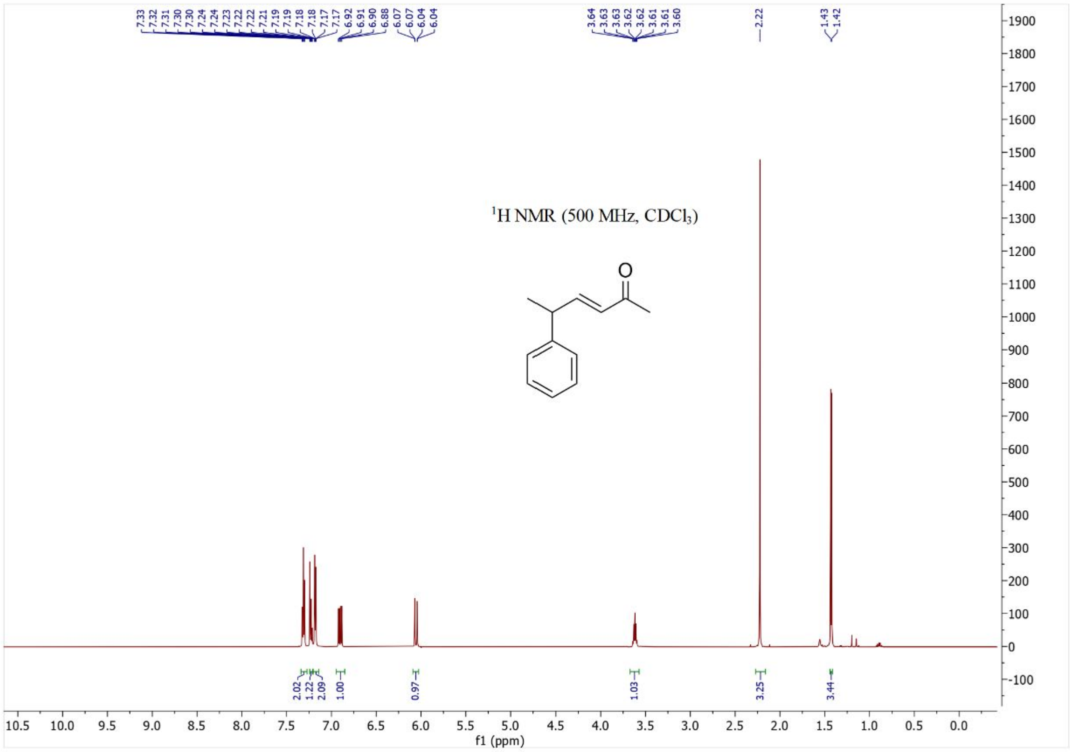

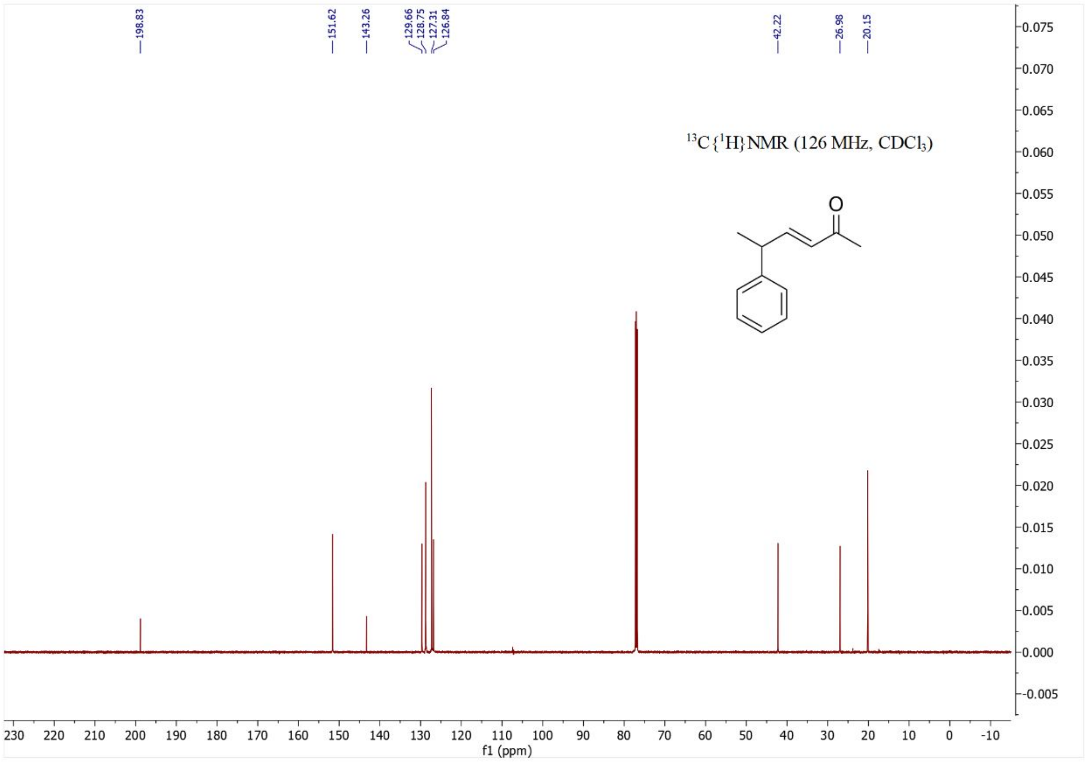

(*E*)-4-(thiophen-2-yl)pent-2-enal (G)

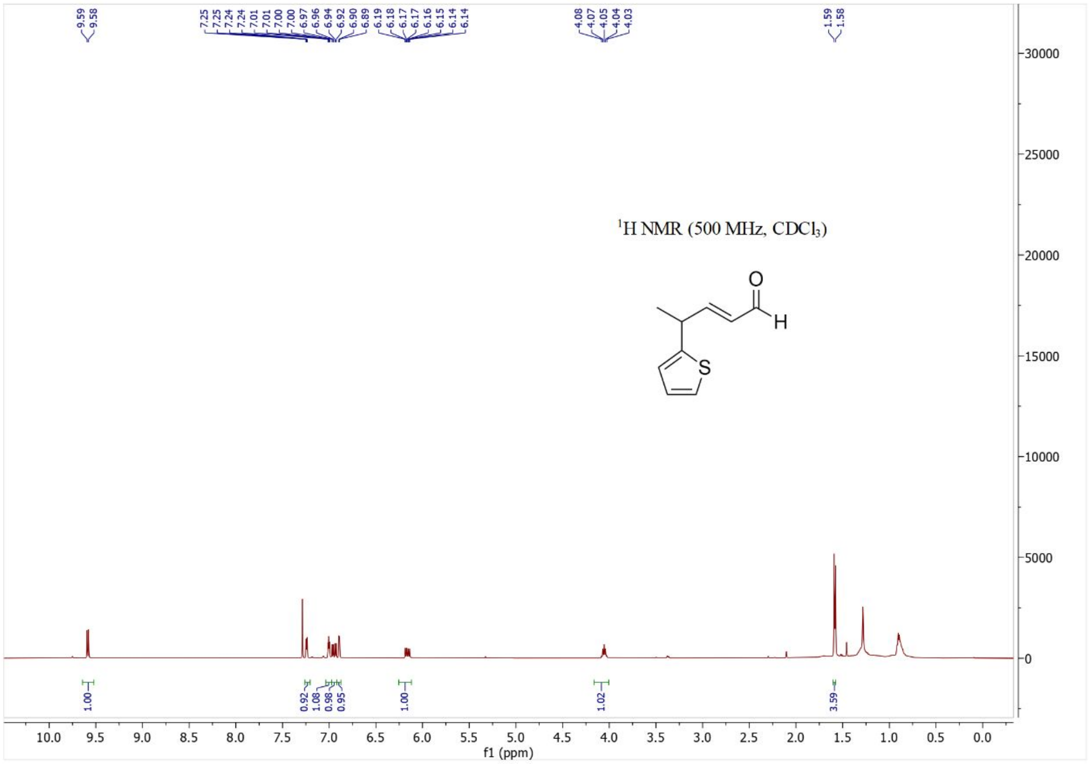

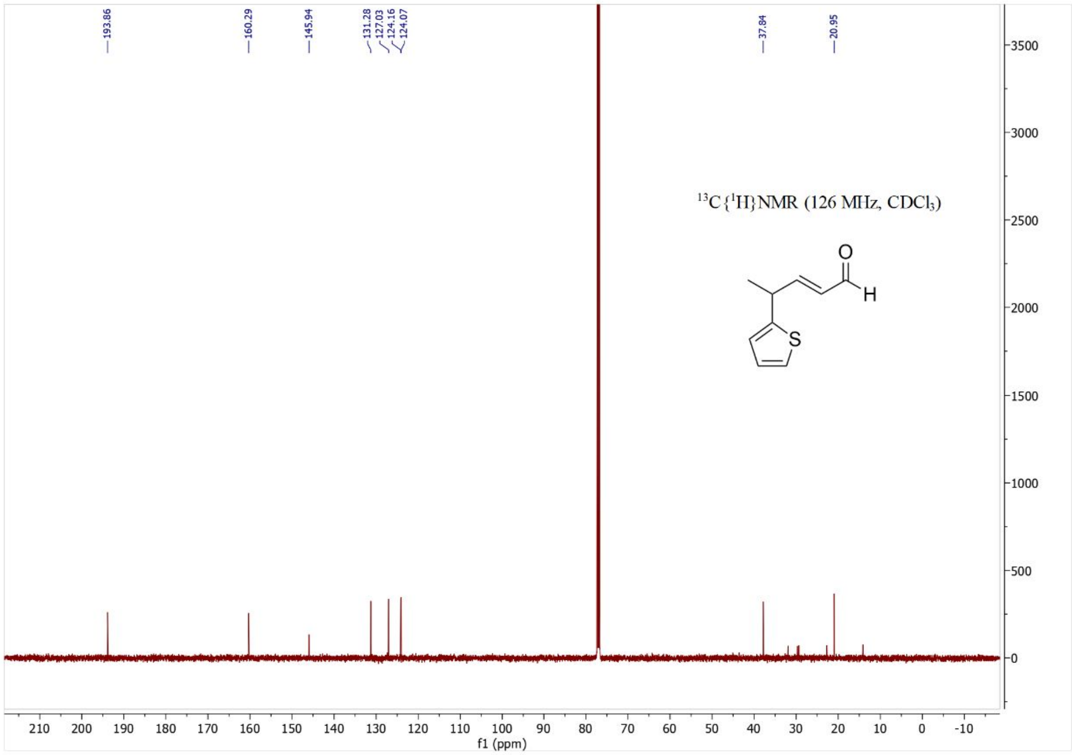

(*E*)-2-(2-phenylcyclohexylidene)acetaldehyde (H)

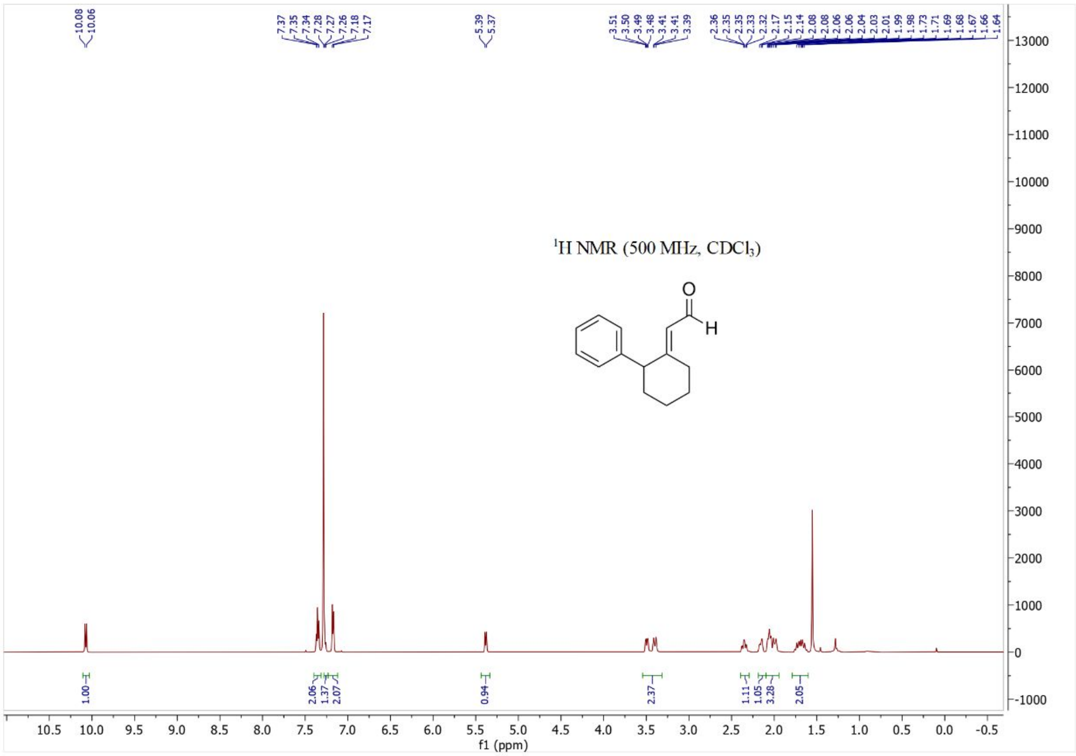

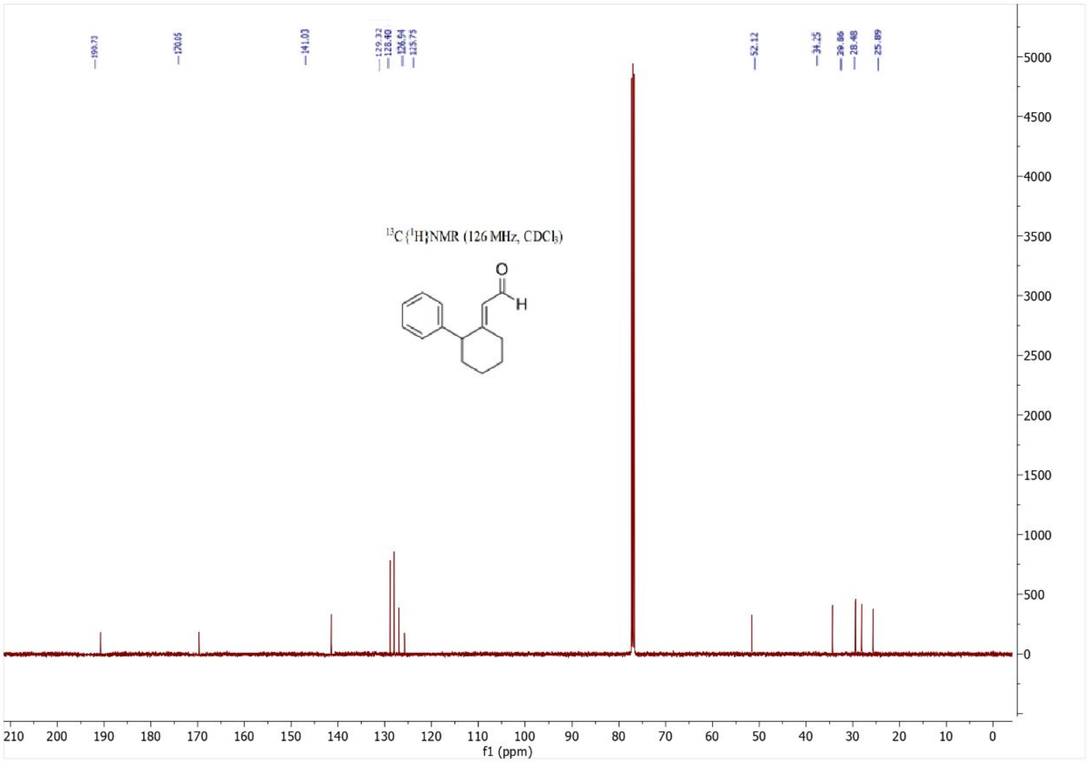

(*E*)-4-hydroxy-4-phenylpent-2-enal (7a)

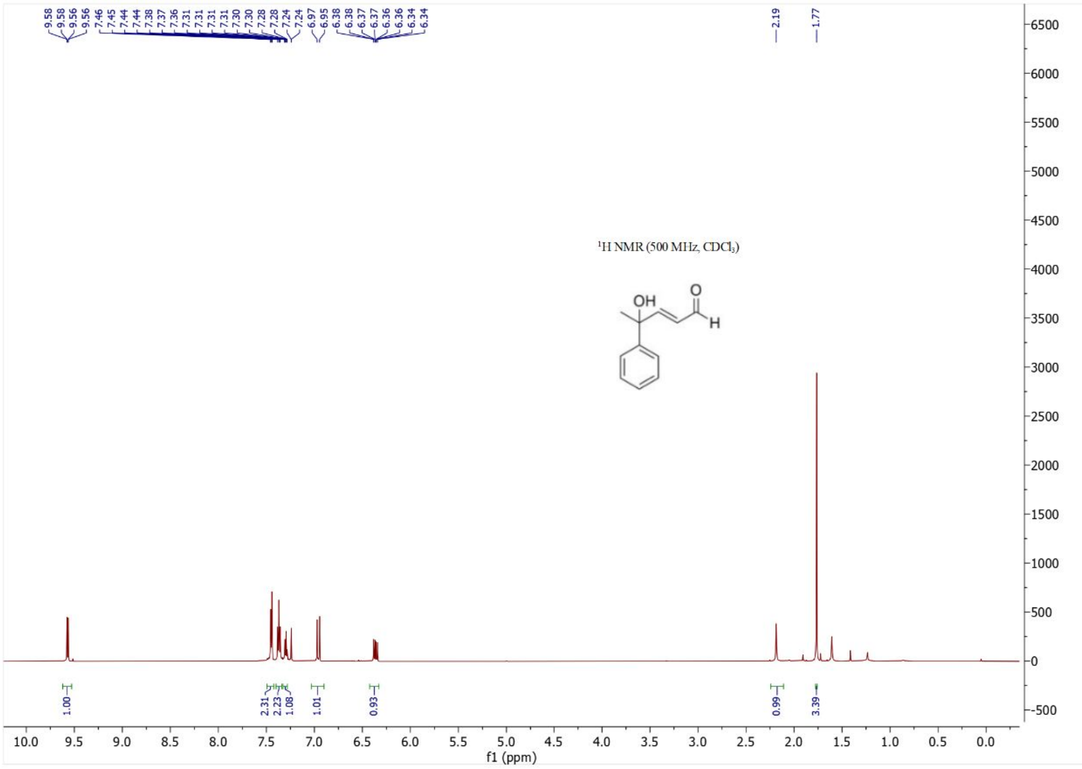

9.58 9.58 9.56 9.56 7.46 7.45 7.44 7.44 7.38 7.37 7.36 7.31 7.31 7.31 7.31 7.30 7.30 7.28 7.28 7.24 7.24 6.97 6.95 6.38 6.38 6.37 6.37 6.36 6.36 6.34 6.34
2.19
1.77
^{1}H NMR (500 MHz, CDCl$_3$)
OH
O
H
1.00
2.31
2.23
1.08
1.01
0.93
0.99
3.39
f1 (ppm)

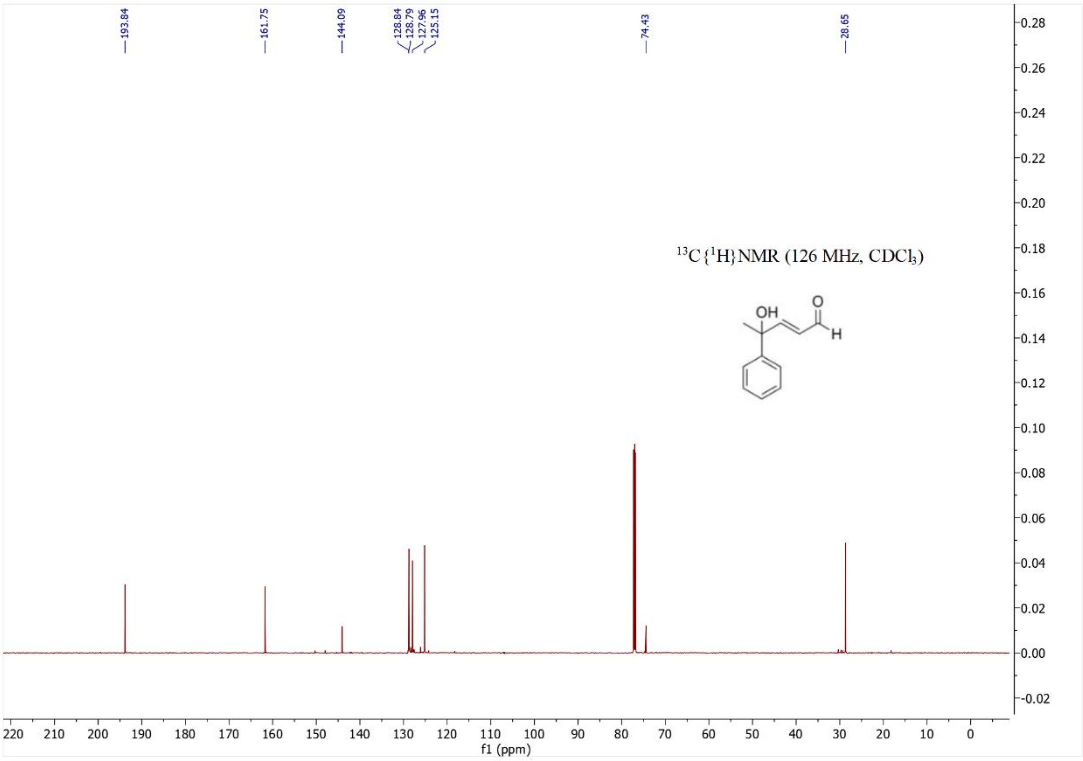

193.84
161.75
144.09
128.84 128.79 127.96 125.15
74.43
28.65
^{13}C{^{1}H}NMR (126 MHz, CDCl$_3$)
OH
O
H
f1 (ppm)

(*E*)-4-(4-fluorophenyl)-4-hydroxypent-2-enal (7b)

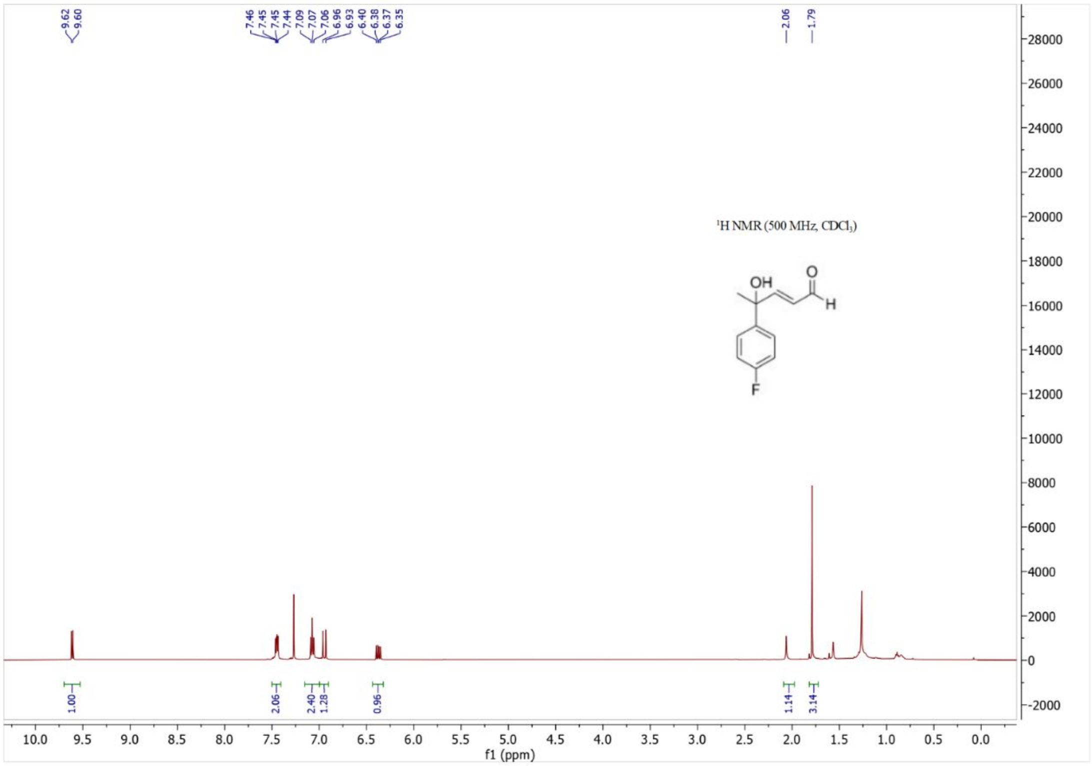

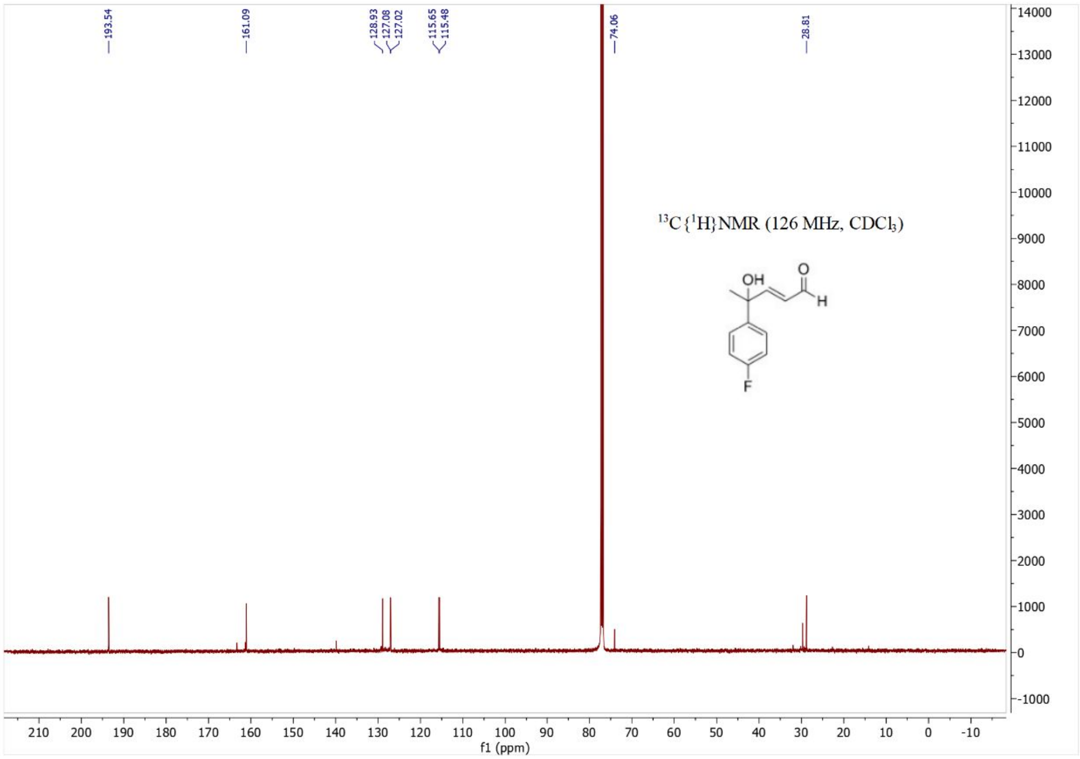

193.54
161.09
128.93
127.08
127.02
115.65
115.48
74.06
28.81
$^{13}C\{^{1}H\}$NMR (126 MHz, $CDCl_3$)
OH
O
H
F
f1 (ppm)

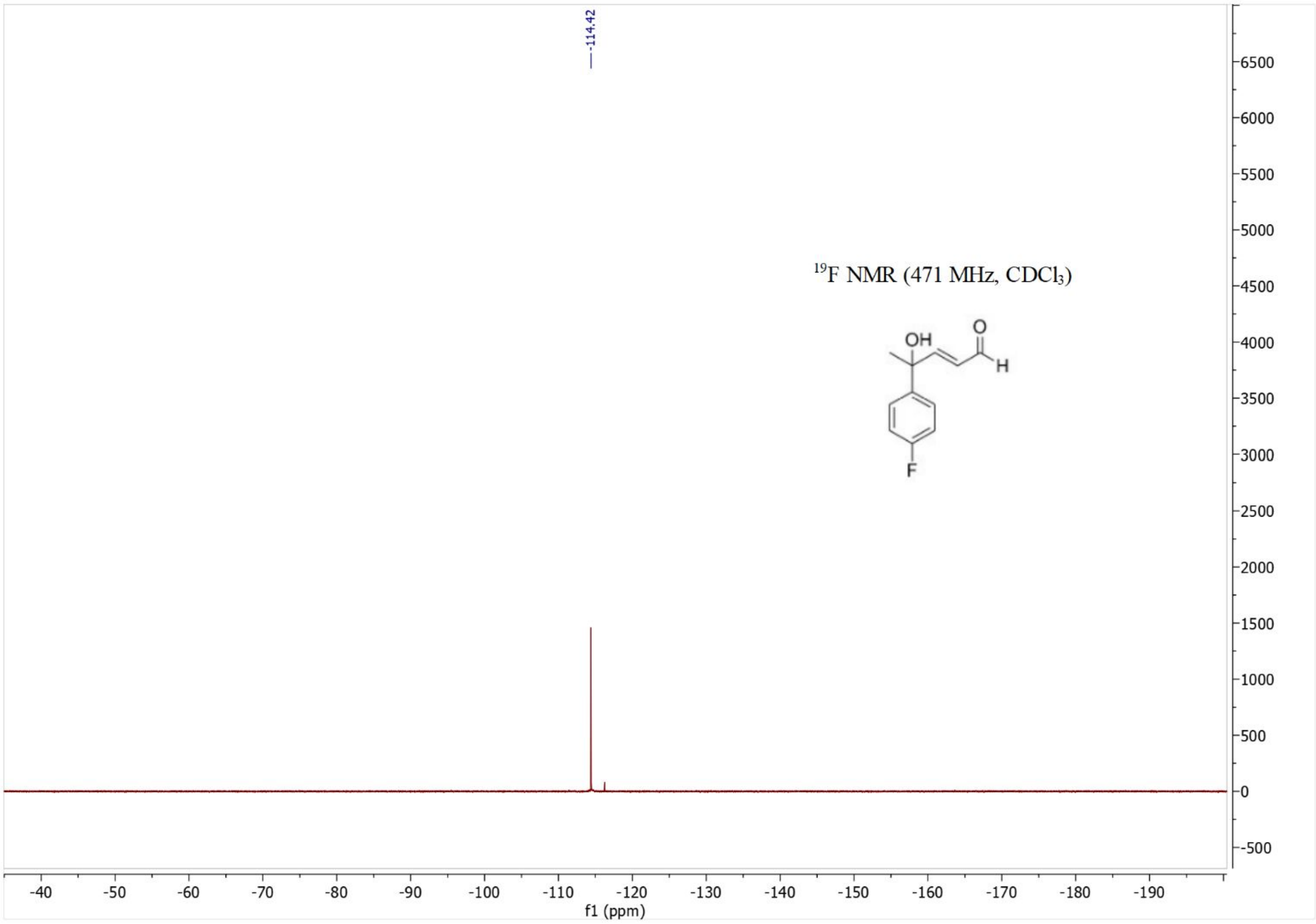
-114.42
^{19}F NMR (471 MHz, CDCl$_3$)
OH
O
H
F
f1 (ppm)

(*E*)-4-hydroxy-4-(p-tolyl)pent-2-enal (7c)

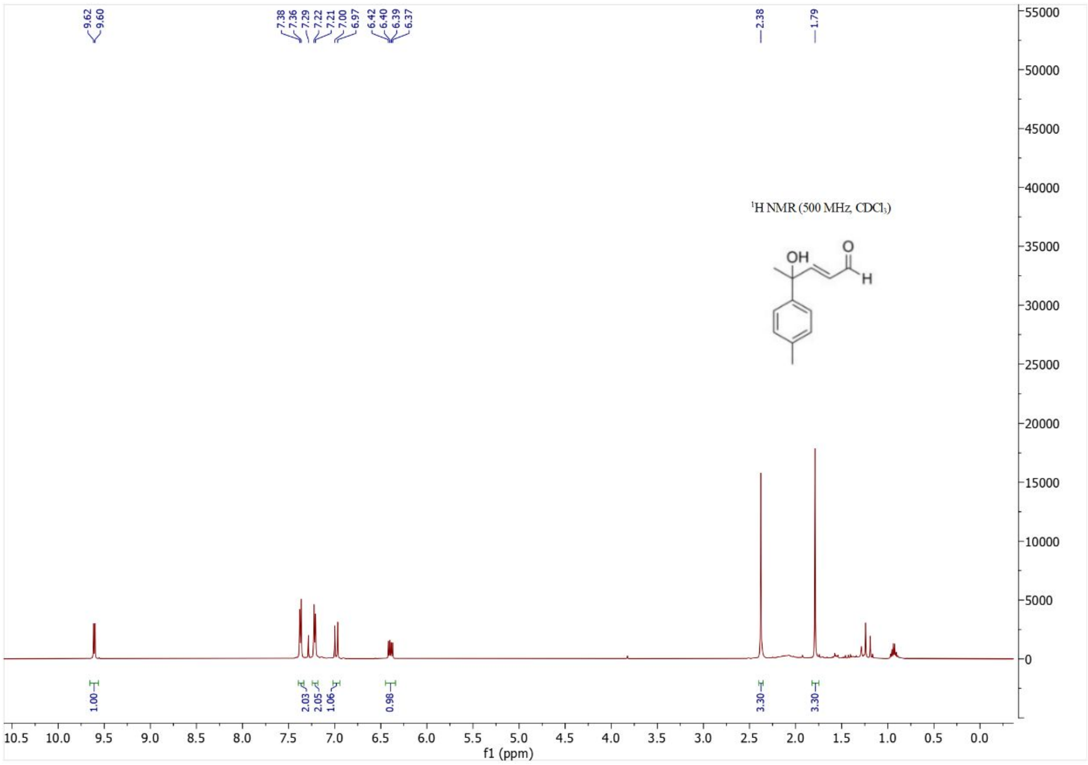

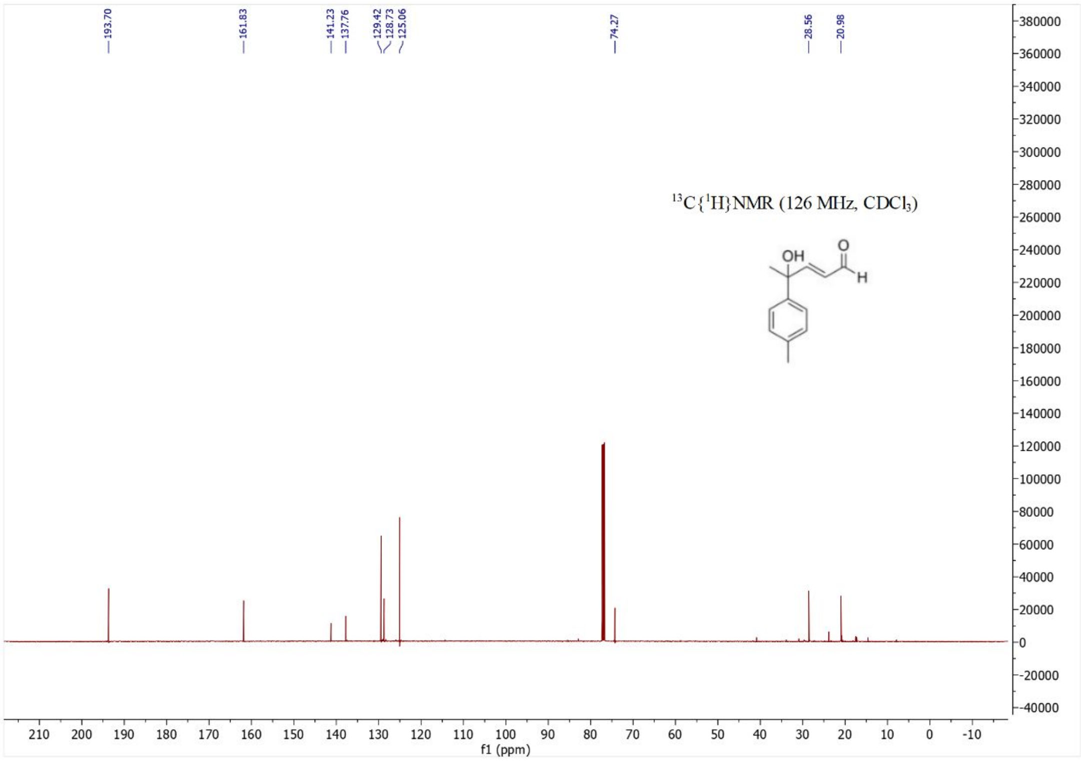

(*E*)-4-hydroxy-4-(4-methoxyphenyl)pent-2-enal (7d)

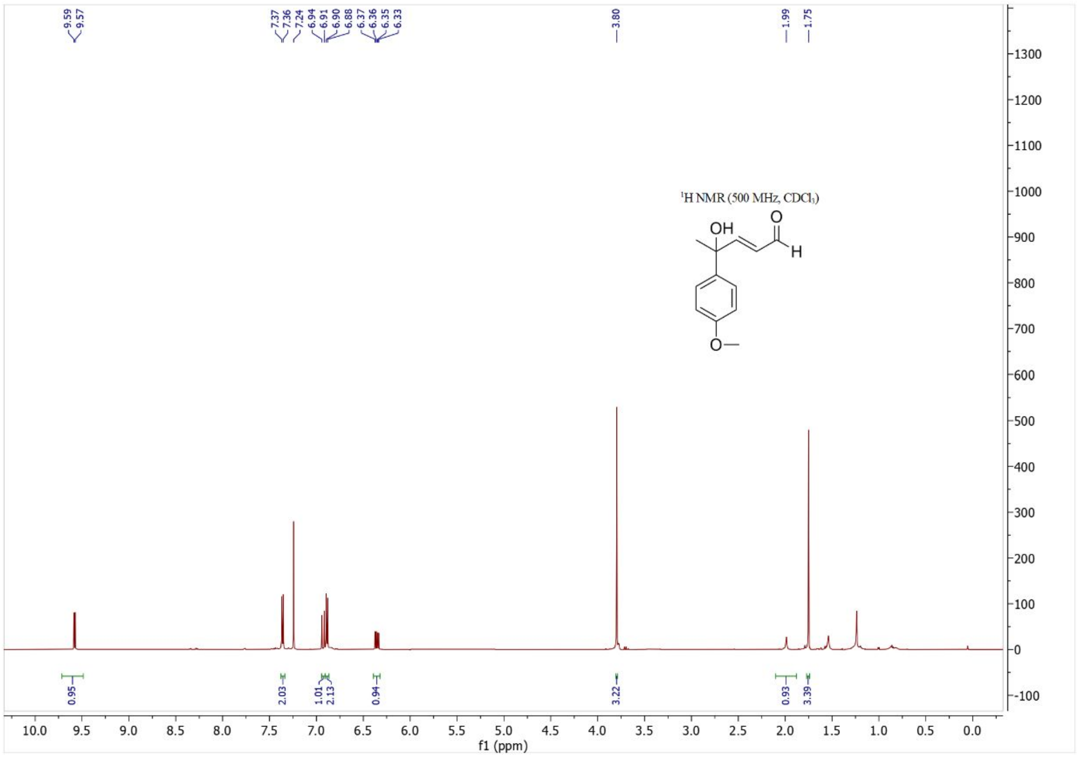

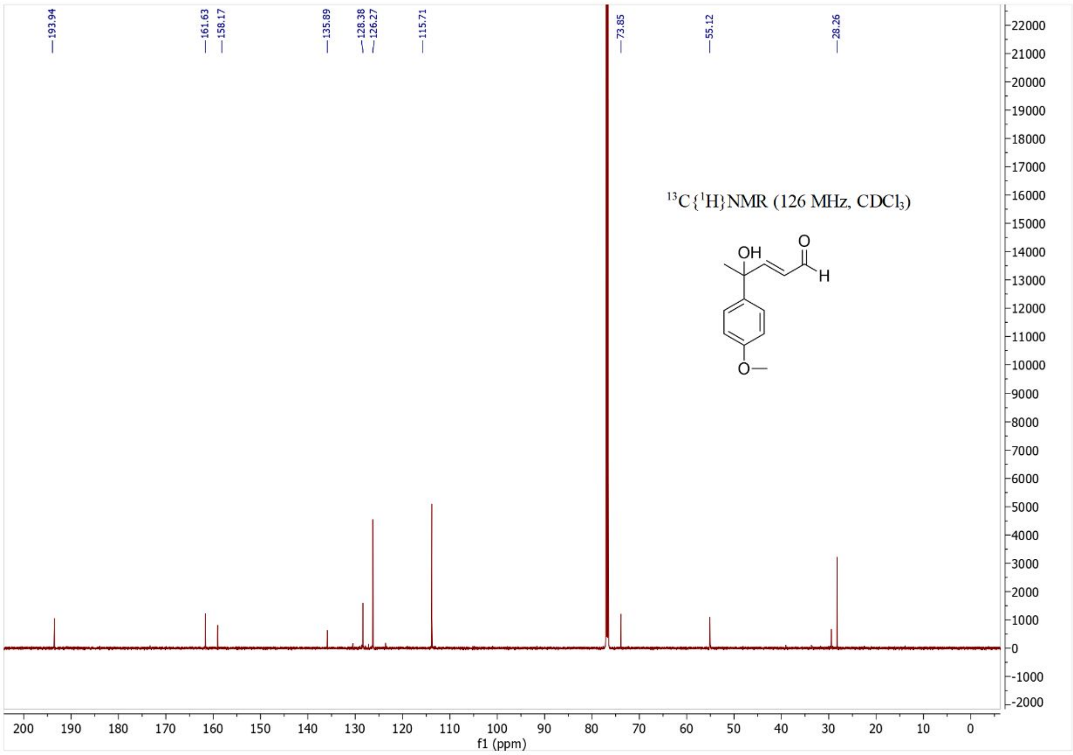

(*E*)-4-hydroxy-4-(4-(trifluoromethyl)phenyl)pent-2-enal (7e)

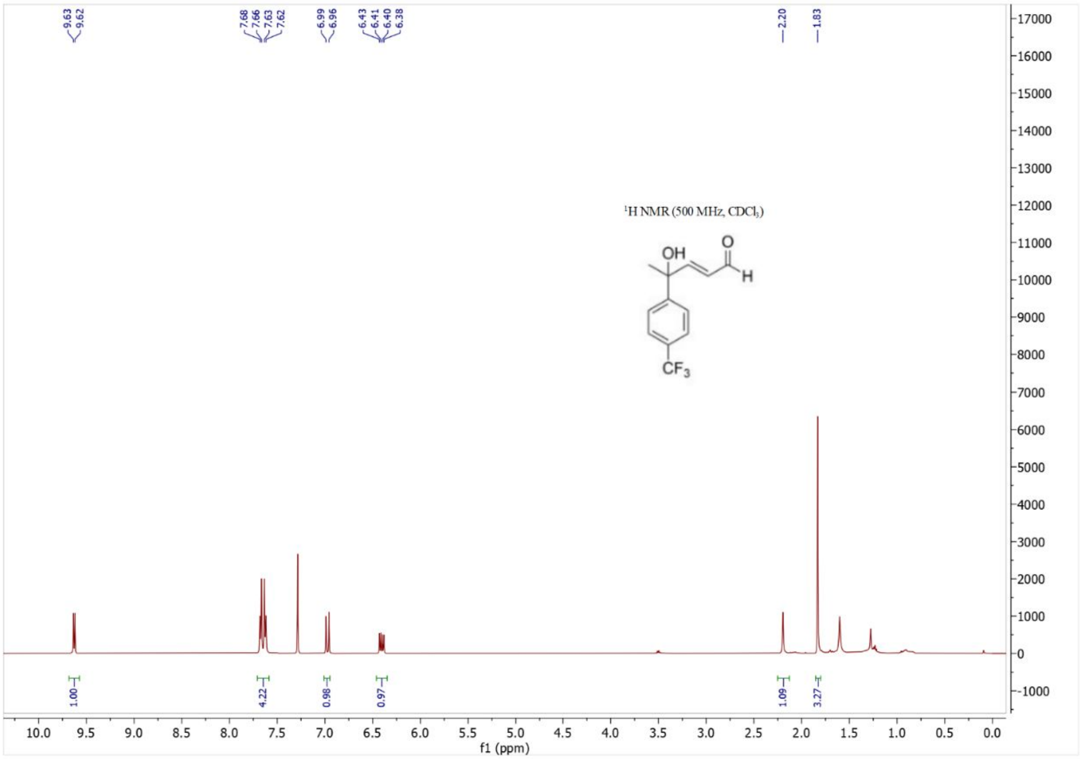

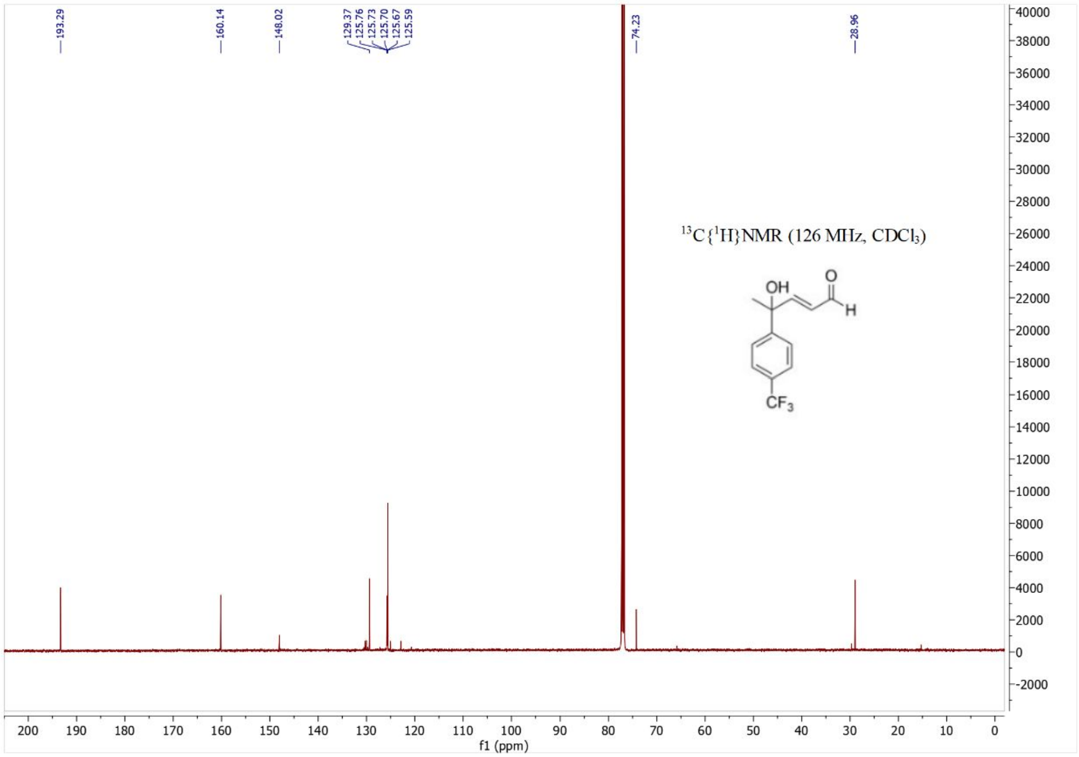

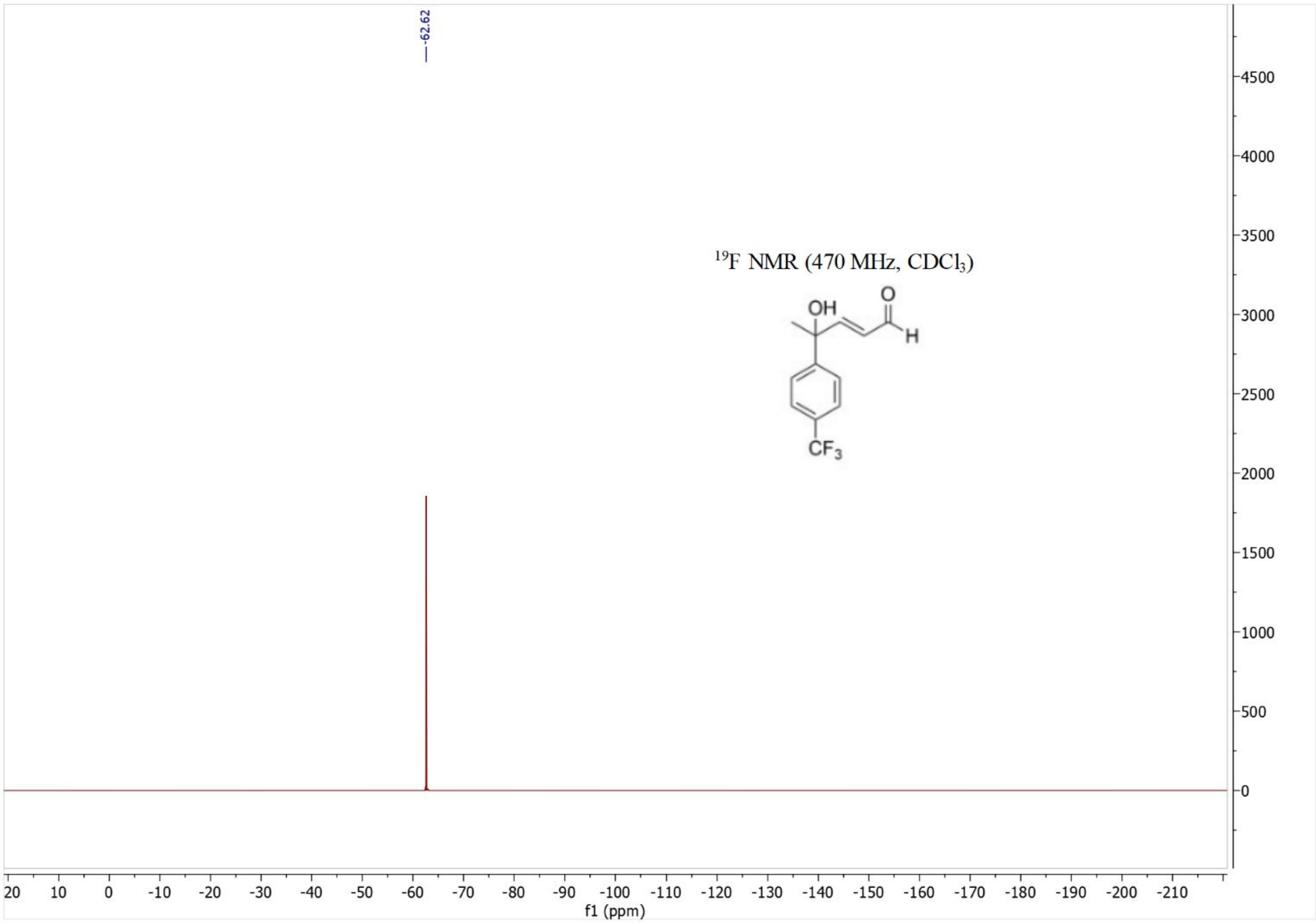

-62.62
19F NMR (470 MHz, CDCl3)
OH
O
H
CF3
4500
4000
3500
3000
2500
2000
1500
1000
500
0
20 10 0 -10 -20 -30 -40 -50 -60 -70 -80 -90 -100 -110 -120 -130 -140 -150 -160 -170 -180 -190 -200 -210
f1 (ppm)

(*E*)-4-hydroxy-4-(4-nitrophenyl)pent-2-enal (7f)

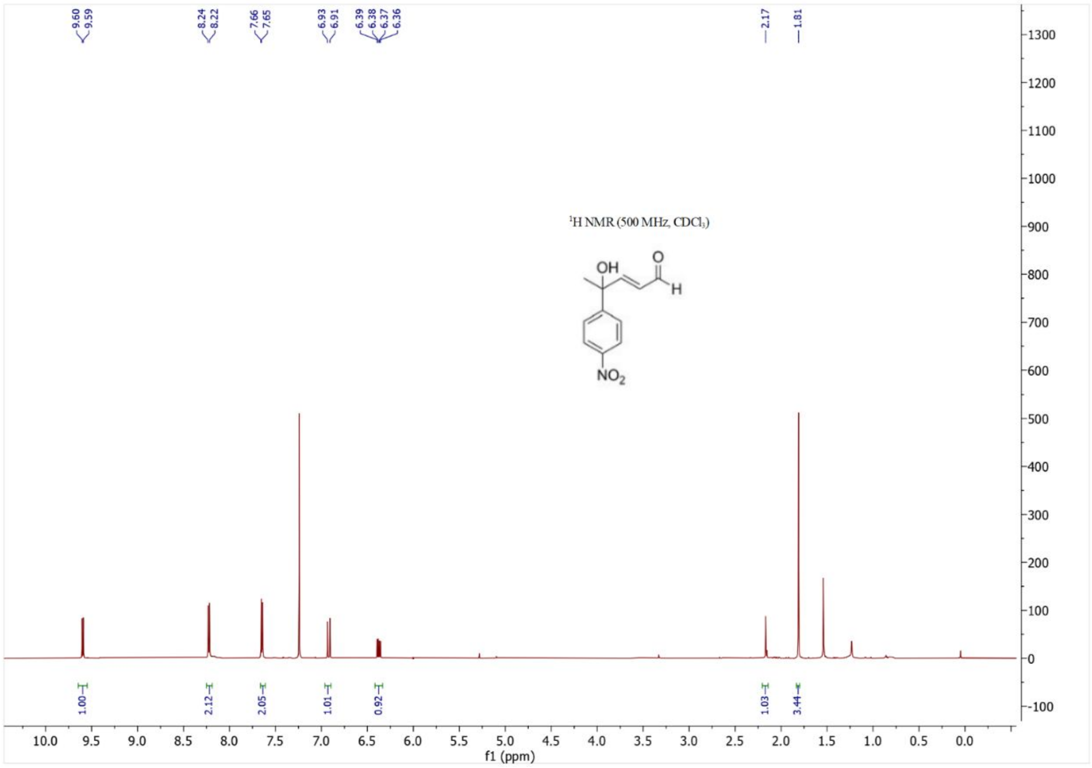

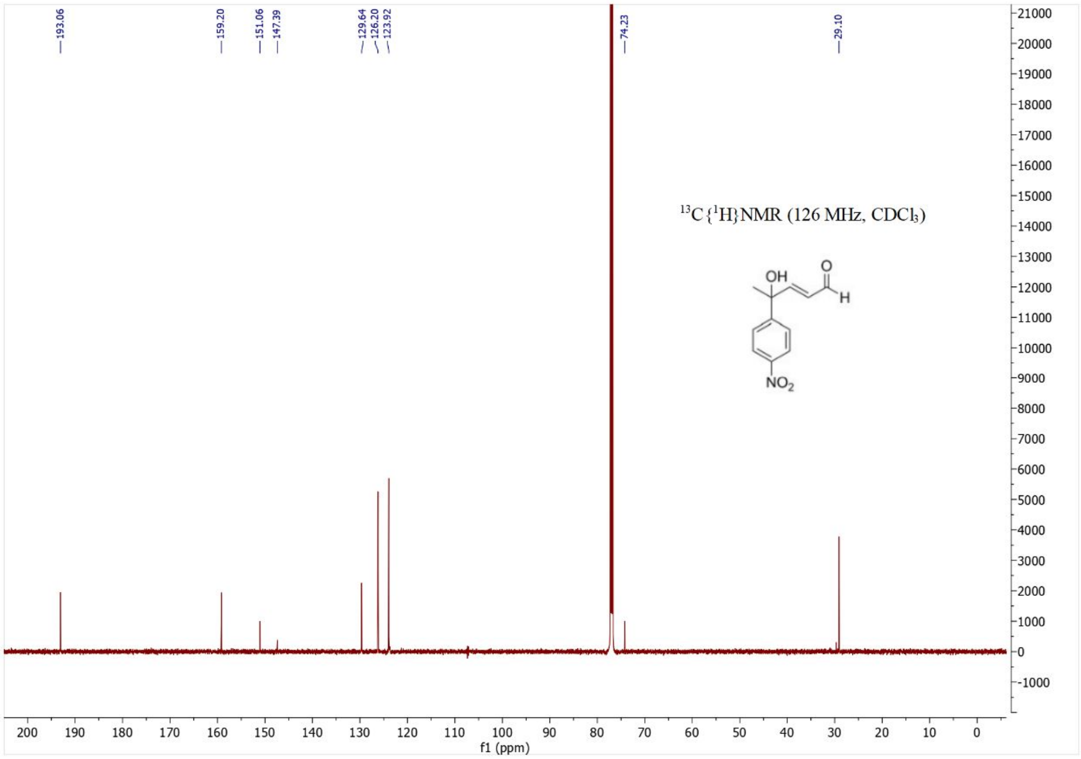

(*E*)-4-hydroxy-4-(3-(trifluoromethyl)phenyl)pent-2-enal (7g)

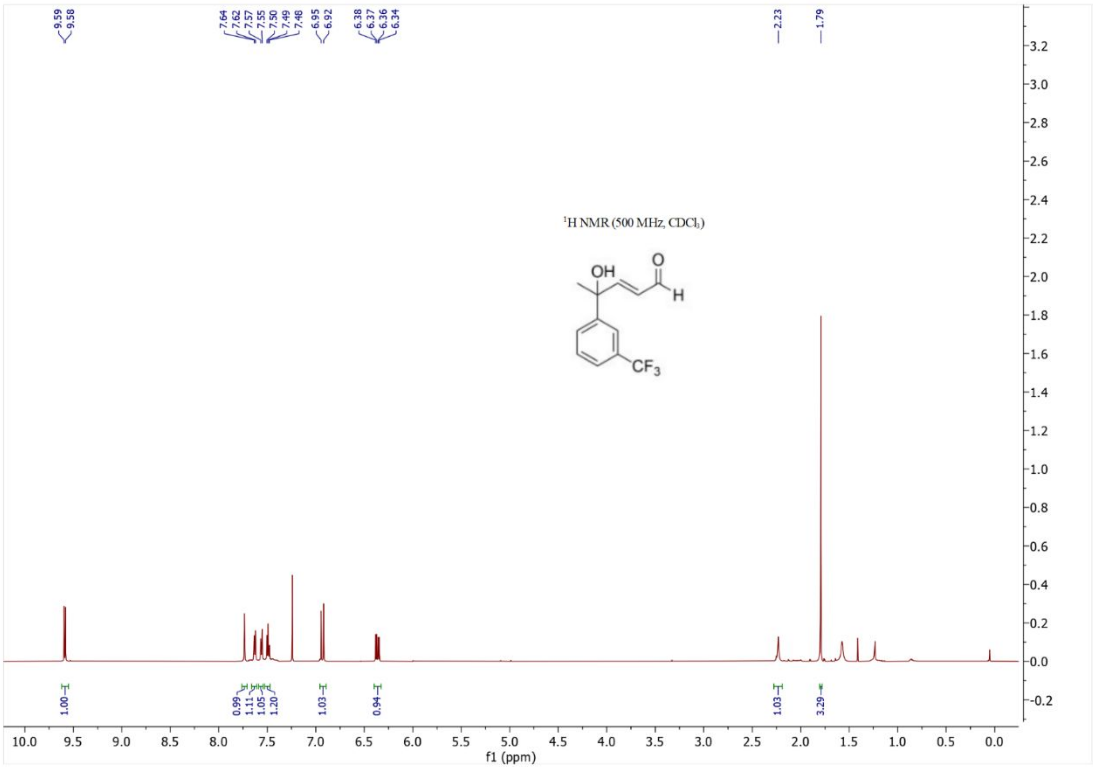

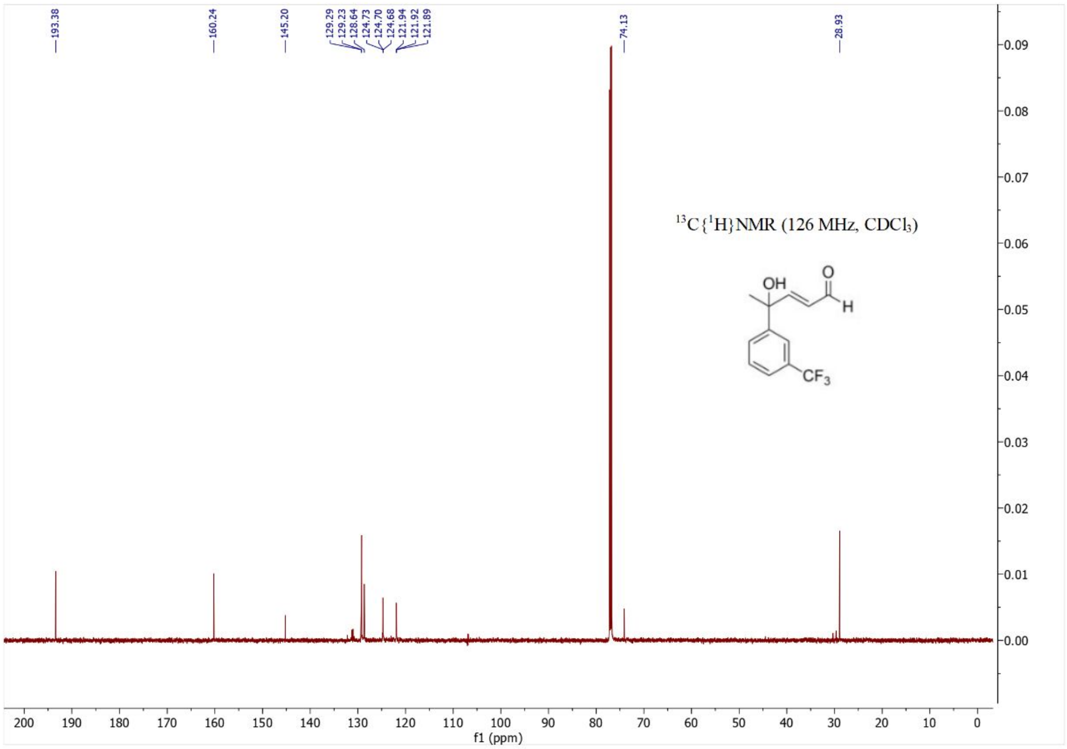
193.38
160.24
145.20
129.29
129.23
128.64
124.73
124.70
124.68
121.94
121.92
121.89
74.13
28.93
$^{13}C\{^1H\}$NMR (126 MHz, $CDCl_3$)
OH
O
H
CF_3
0.09
0.08
0.07
0.06
0.05
0.04
0.03
0.02
0.01
0.00
200 190 180 170 160 150 140 130 120 110 100 90 80 70 60 50 40 30 20 10 0
f1 (ppm)

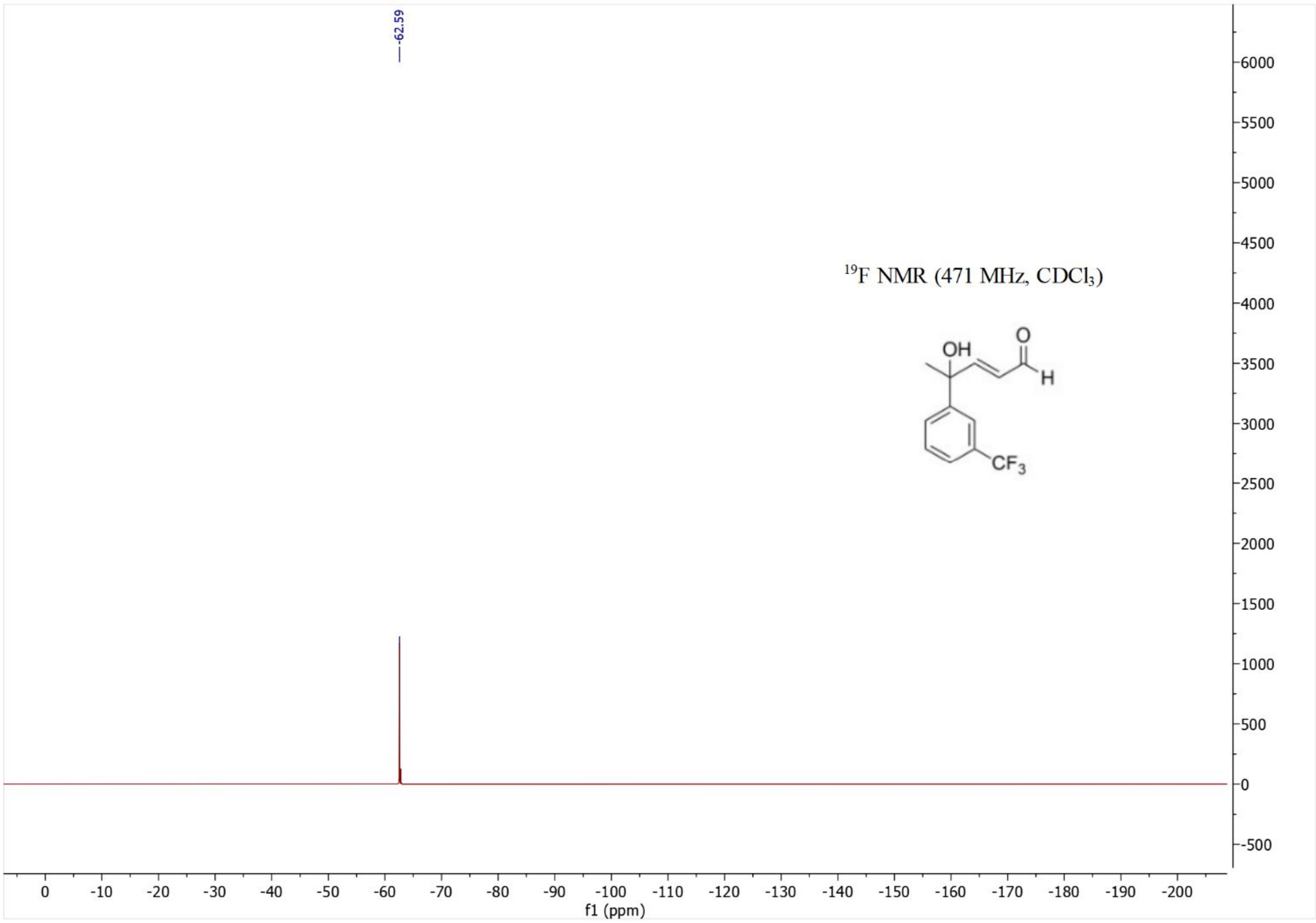
-62.59
¹⁹F NMR (471 MHz, CDCl₃)
OH
O
H
CF₃
f1 (ppm)

(*E*)-4-(3-bromophenyl)-4-hydroxypent-2-enal (7h)

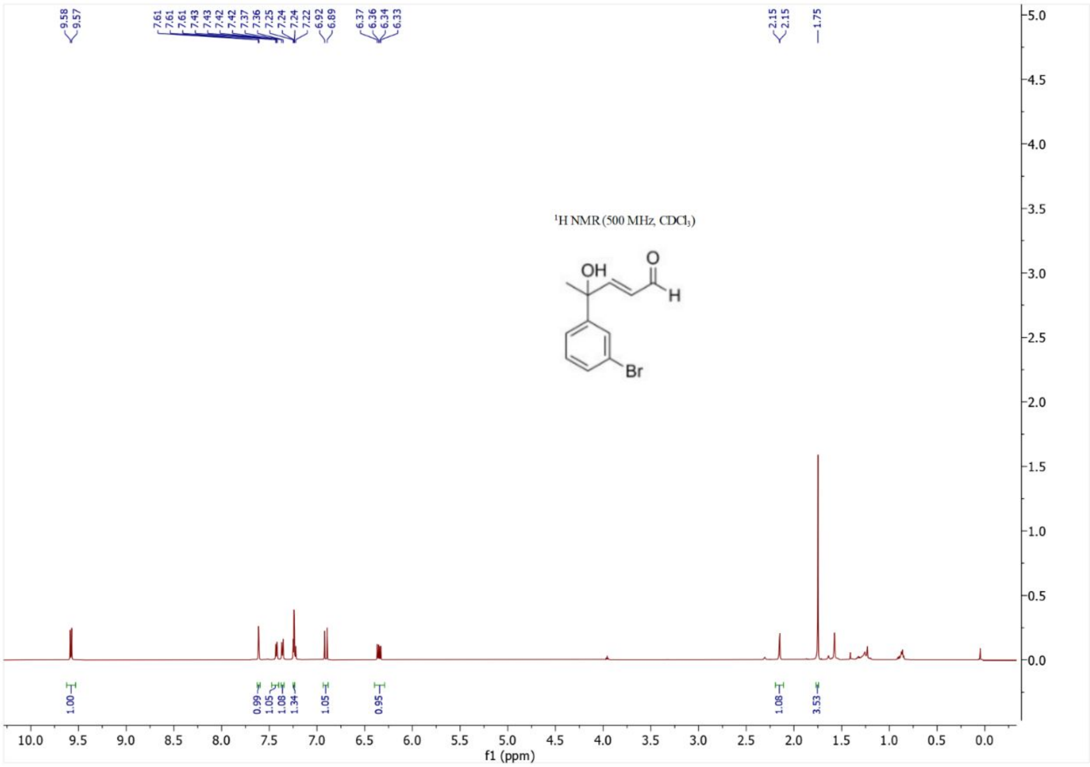

.

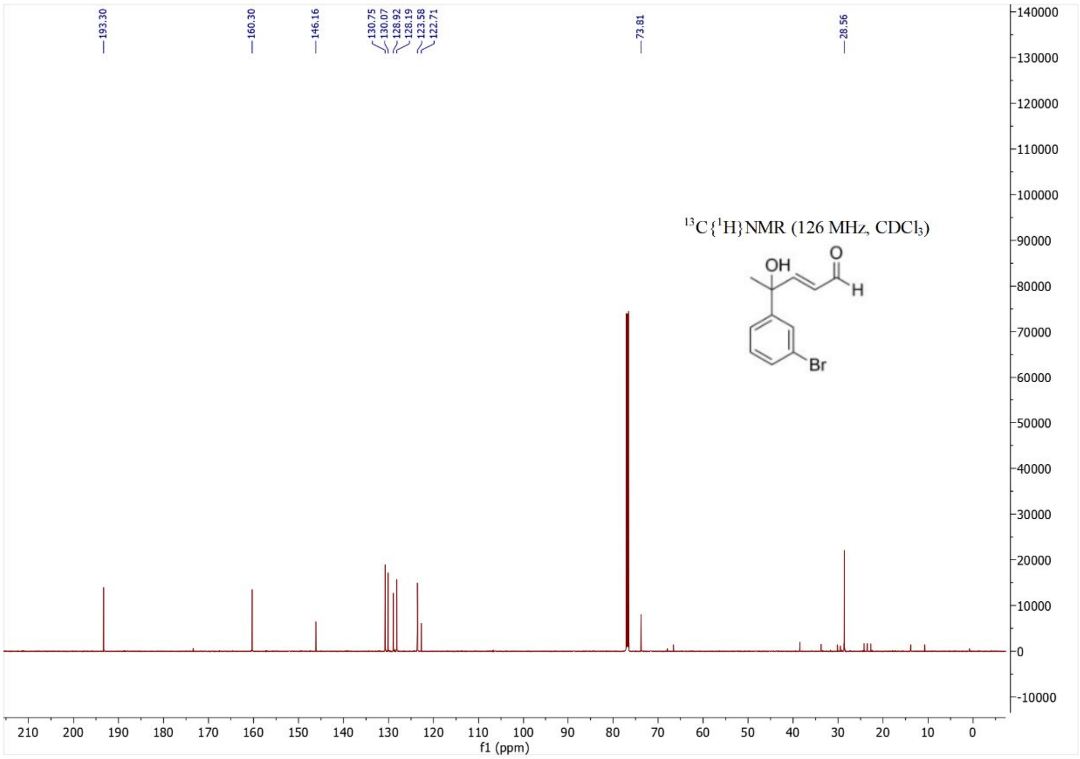

193.30
160.30
146.16
130.75
130.07
128.92
128.19
123.58
122.71
73.81
28.56
$^{13}C\{^1H\}$NMR (126 MHz, $CDCl_3$)
OH
O
H
Br
f1 (ppm)

(*E*)-4-hydroxy-4-(4-isobutylphenyl)pent-2-enal (7i)

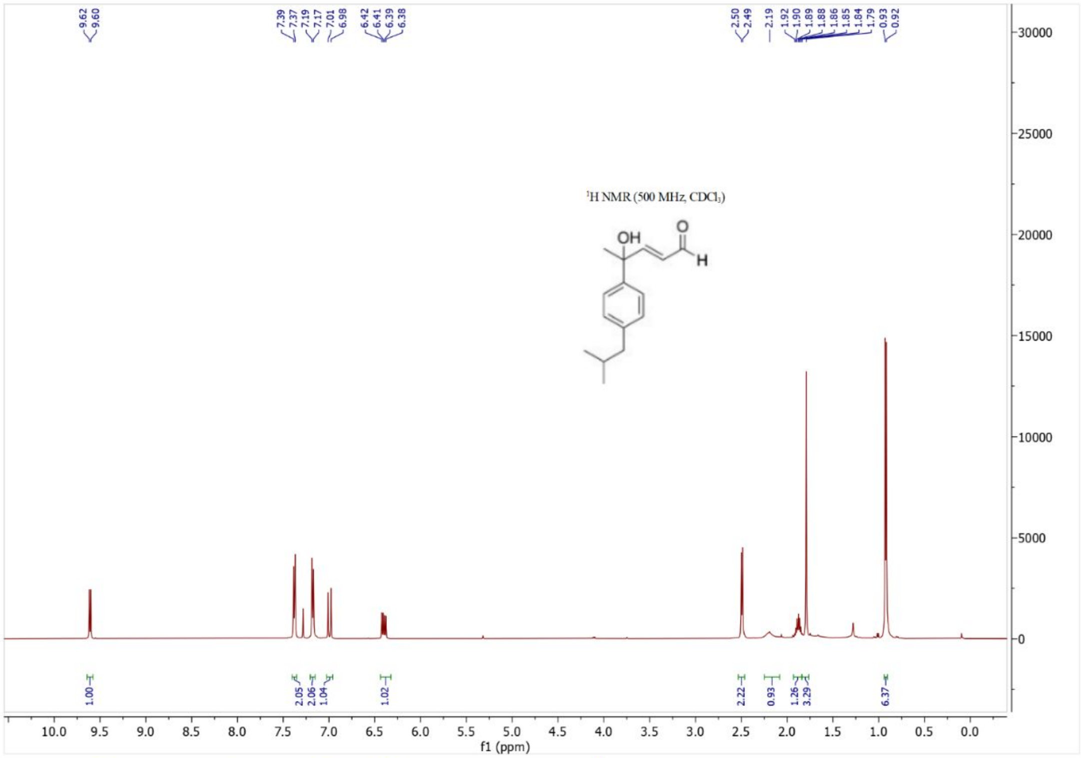

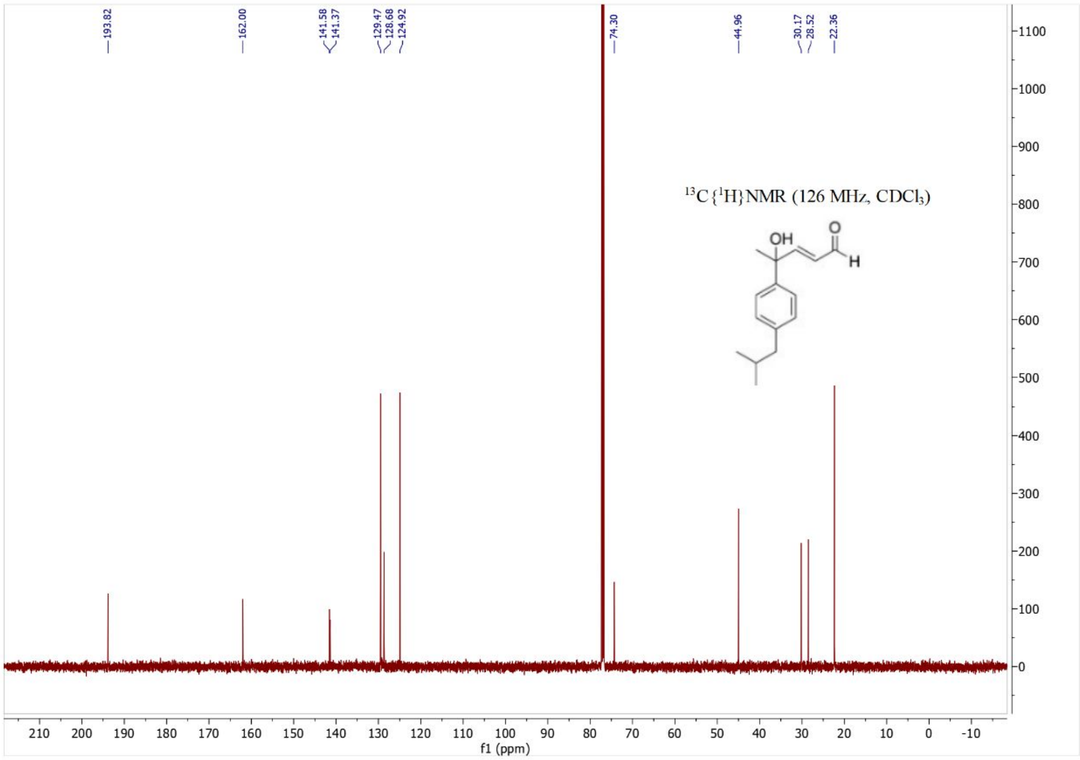

(*E*)-4-hydroxy-4-(naphthalen-2-yl)pent-2-enal (7j)

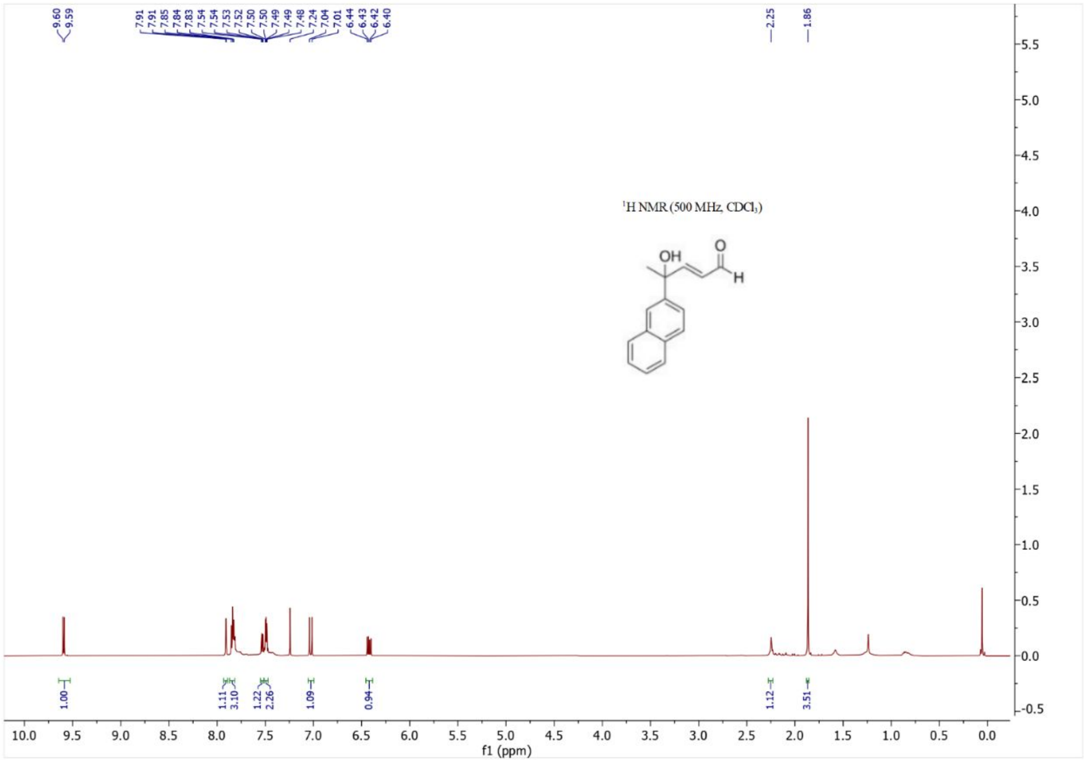

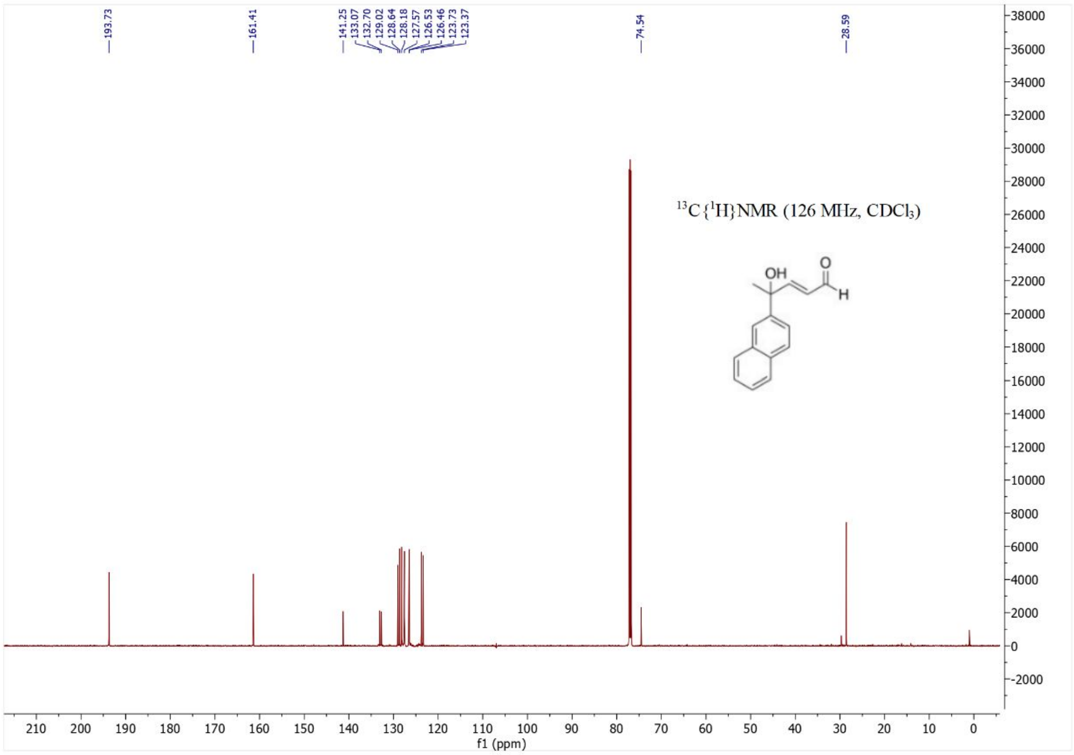

(E)-4-hydroxy-4-(thiophen-3-yl)pent-2-enal (7k)

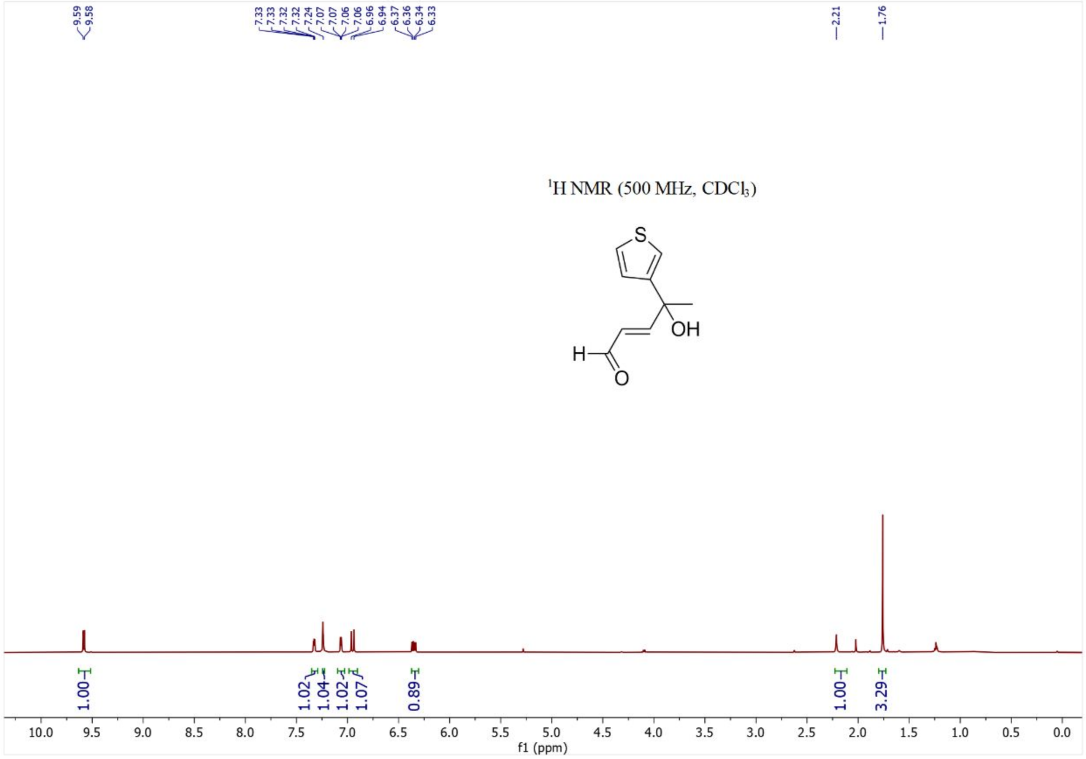

9.59
9.58
7.33
7.33
7.32
7.32
7.24
7.07
7.07
7.06
7.06
6.96
6.94
6.37
6.36
6.34
6.33
2.21
1.76
^{1}H NMR (500 MHz, $CDCl_3$)
S
OH
H
O
1.00
1.02
1.04
1.02
1.07
0.89
1.00
3.29
10.0
9.5
9.0
8.5
8.0
7.5
7.0
6.5
6.0
5.5
5.0
4.5
4.0
3.5
3.0
2.5
2.0
1.5
1.0
0.5
0.0
f1 (ppm)

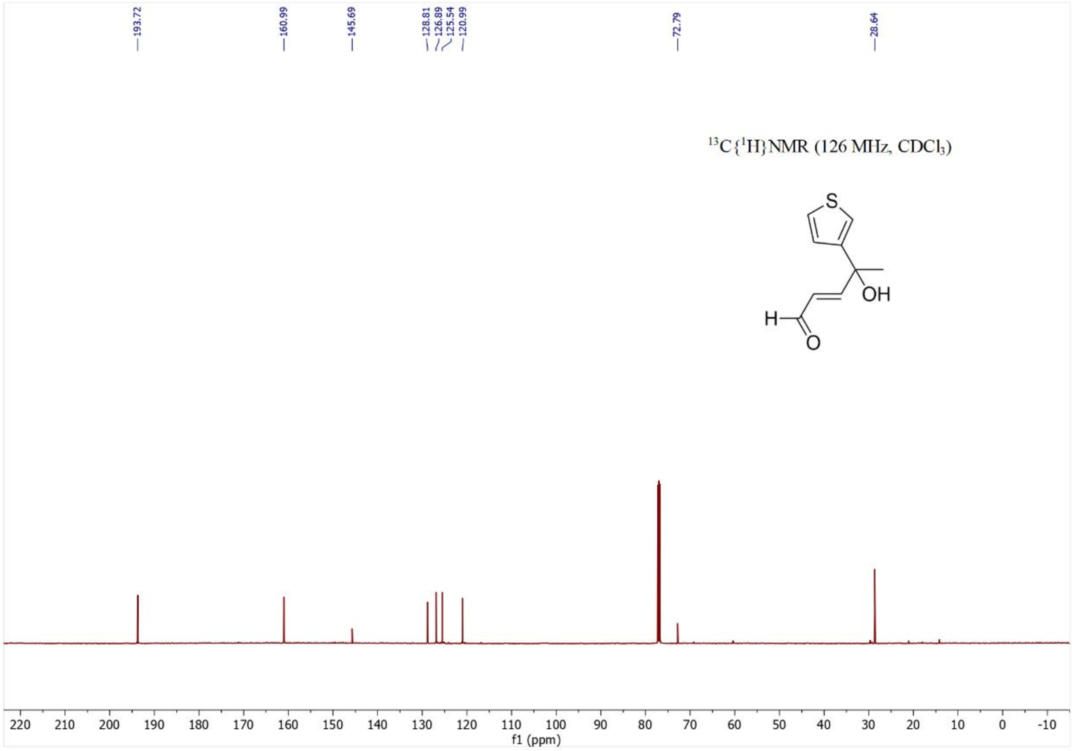

(2*E*,4*Z*)-4-((3*S*,10*R*,13*S*,16*S*)-3-((tert-butyldimethylsilyl)oxy)-16-hydroxy-10,13-dimethyl-1,2,3,4,7,8,9,10,11,12,13,14,15,16-tetradecahydro-17H-cyclopenta[a]phenanthren-17-ylidene)pent-2-enal (7l major)

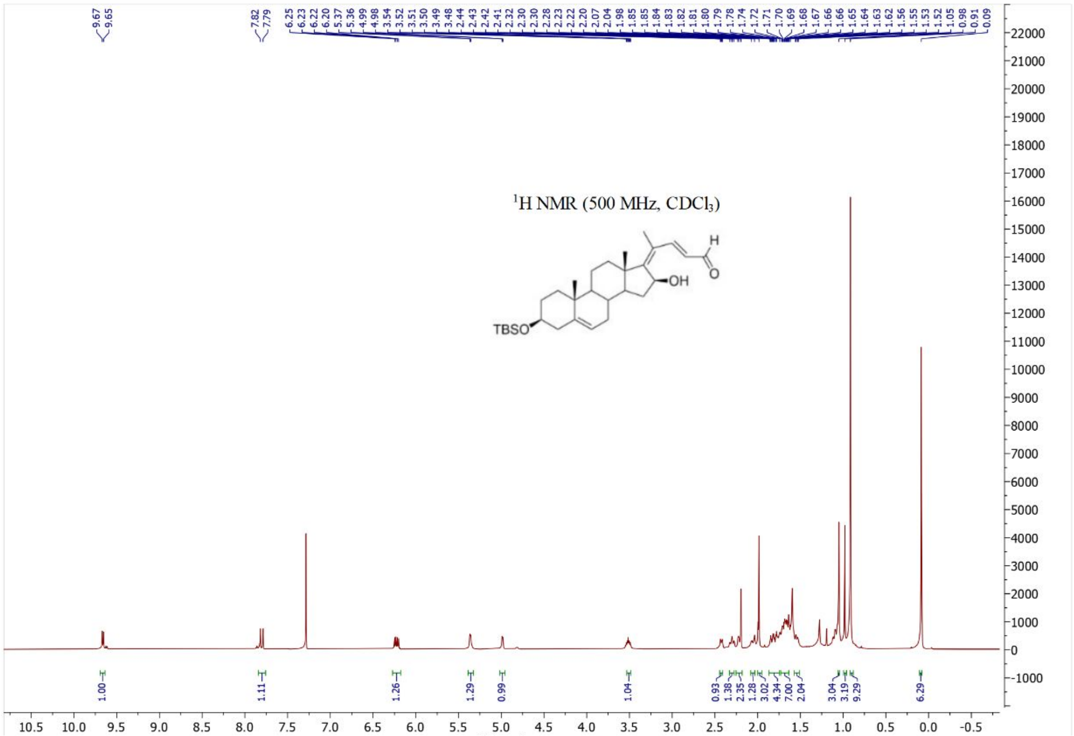

1H NMR (500 MHz, CDCl3)
TBSO
OH
H
O

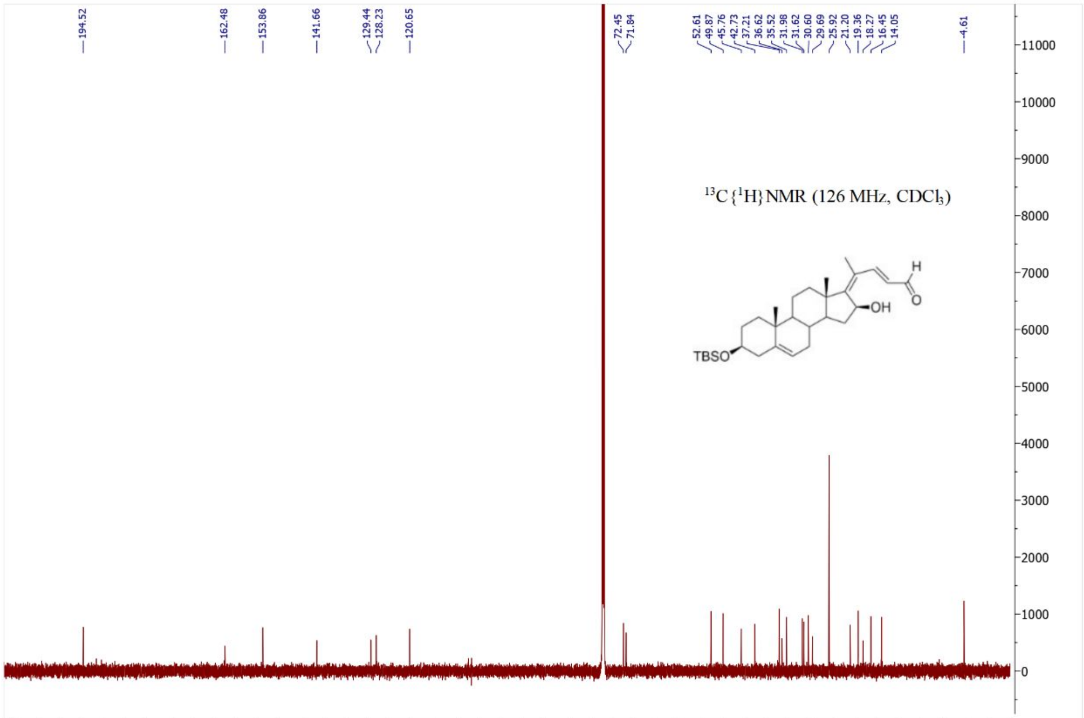

13C{1H} NMR (126 MHz, CDCl3)
TBSO
OH
H
O
f1 (ppm)

NOESY

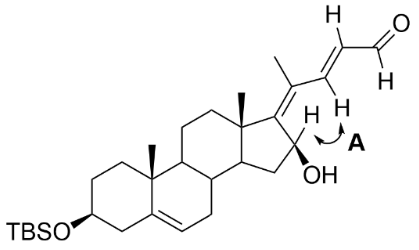

H
O
H
H
H
A
OH
TBSO

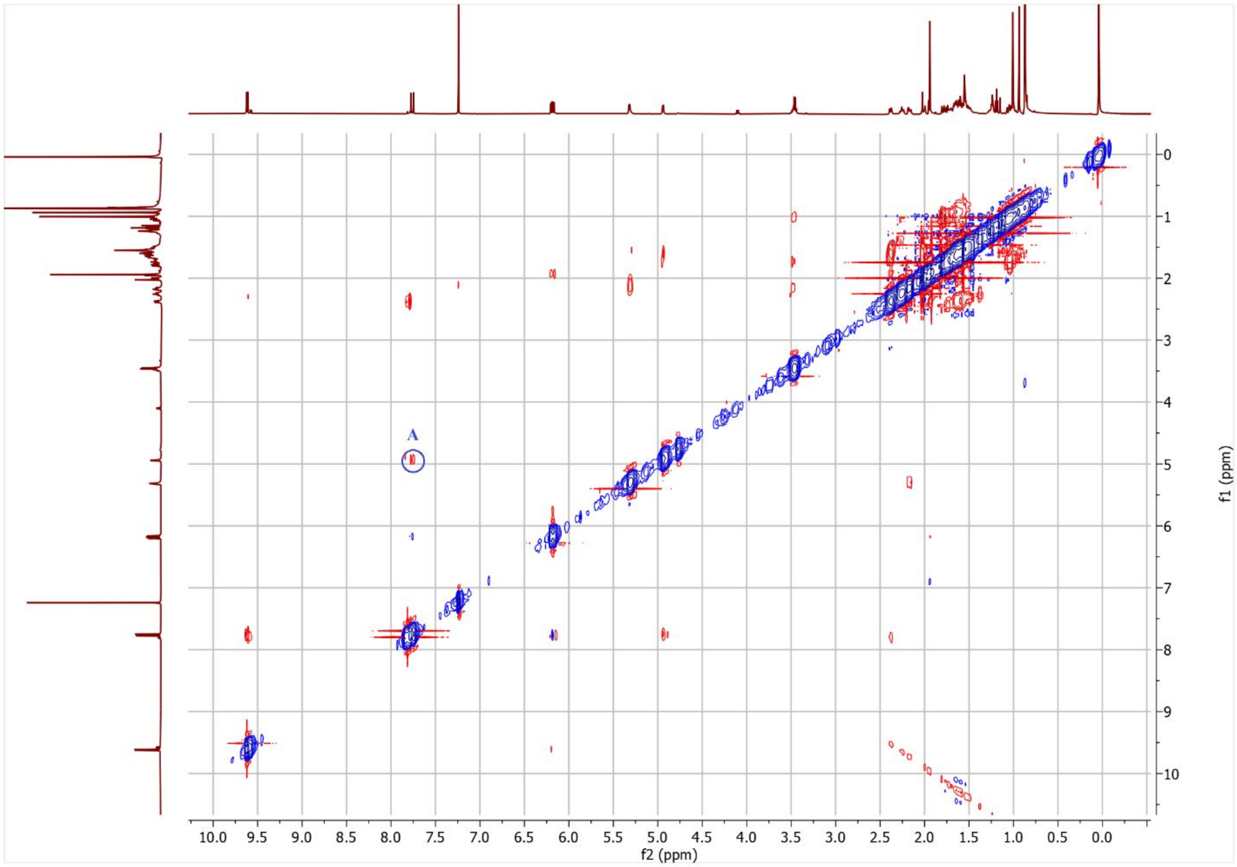

A
f1 (ppm)
f2 (ppm)

(2*E*,4*E*)-4-((3*S*,10*R*,13*S*,16*S*)-3-((tert-butyldimethylsilyl)oxy)-16-hydroxy-10,13-dimethyl-1,2,3,4,7,8,9,10,11,12,13,14,15,16-tetradecahydro-17H-cyclopenta[a]phenanthren-17-ylidene)pent-2-enal (7l minor)

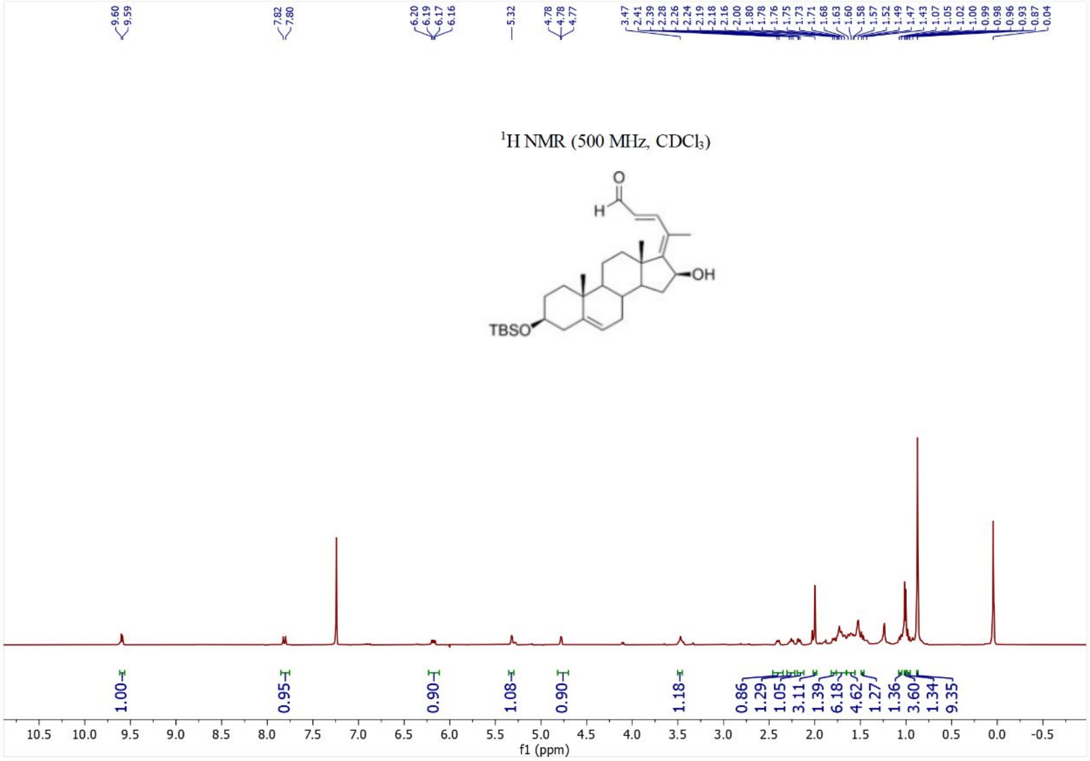

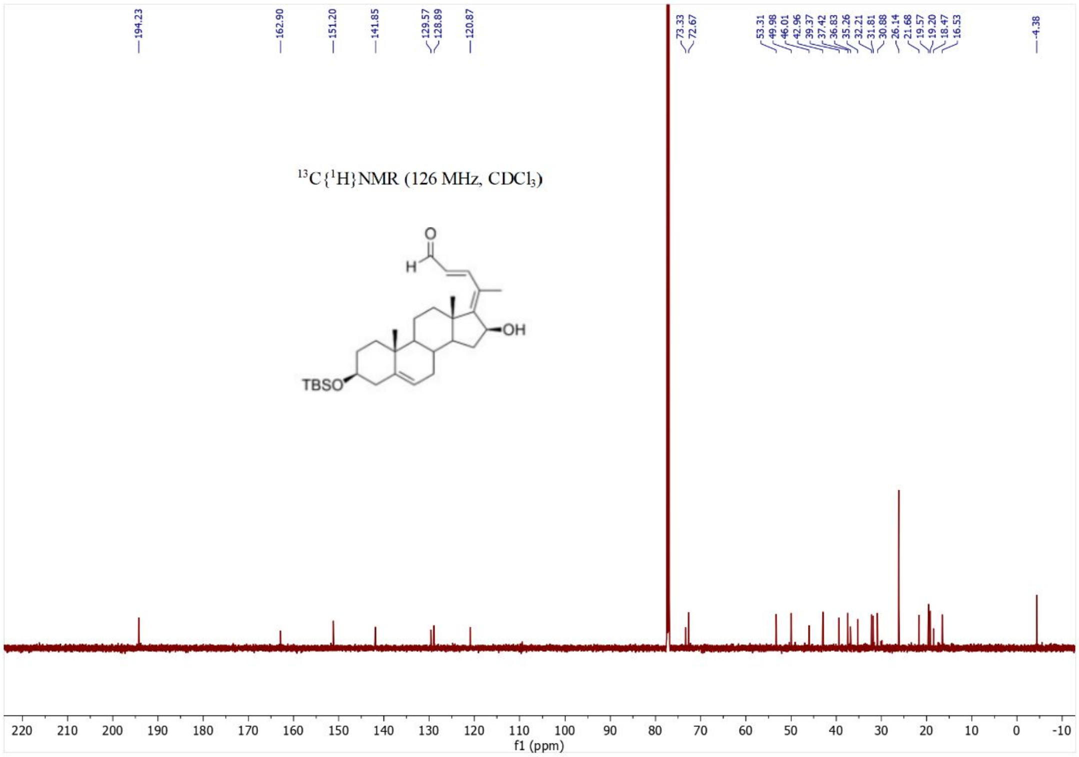
194.23
162.90
151.20
141.85
129.57
128.89
120.87
73.33
72.67
53.31
49.98
46.01
42.96
39.37
37.42
36.83
35.26
32.21
31.81
30.88
26.14
21.68
19.57
19.20
18.47
16.53
-4.38
13C{1H}NMR (126 MHz, CDCl3)
TBSO
OH
f1 (ppm)

NOESY

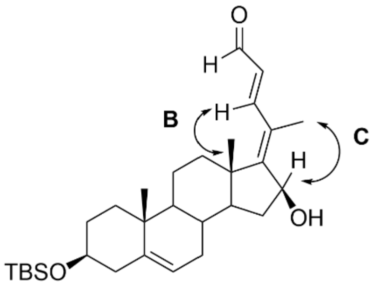
B
C
TBSO
OH

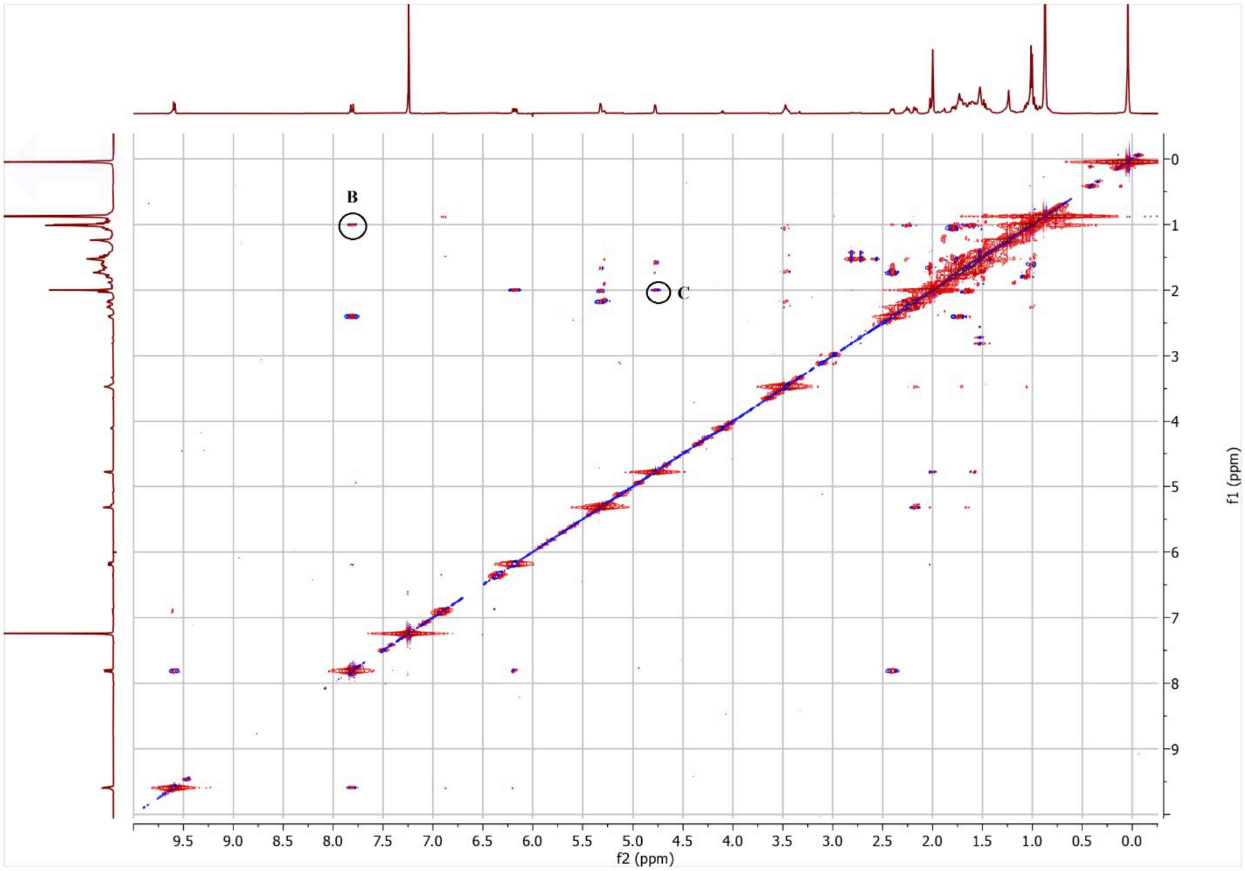

(*E*)-3-(4-hydroxychroman-4-yl)acrylaldehyde (7m)

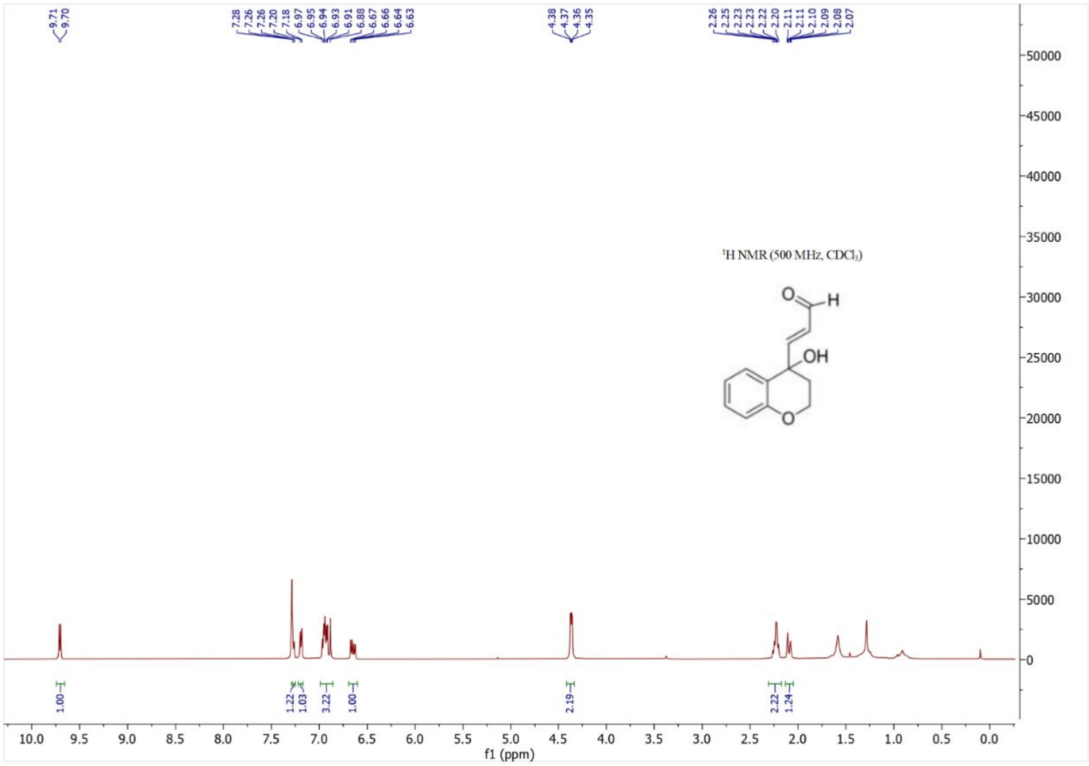

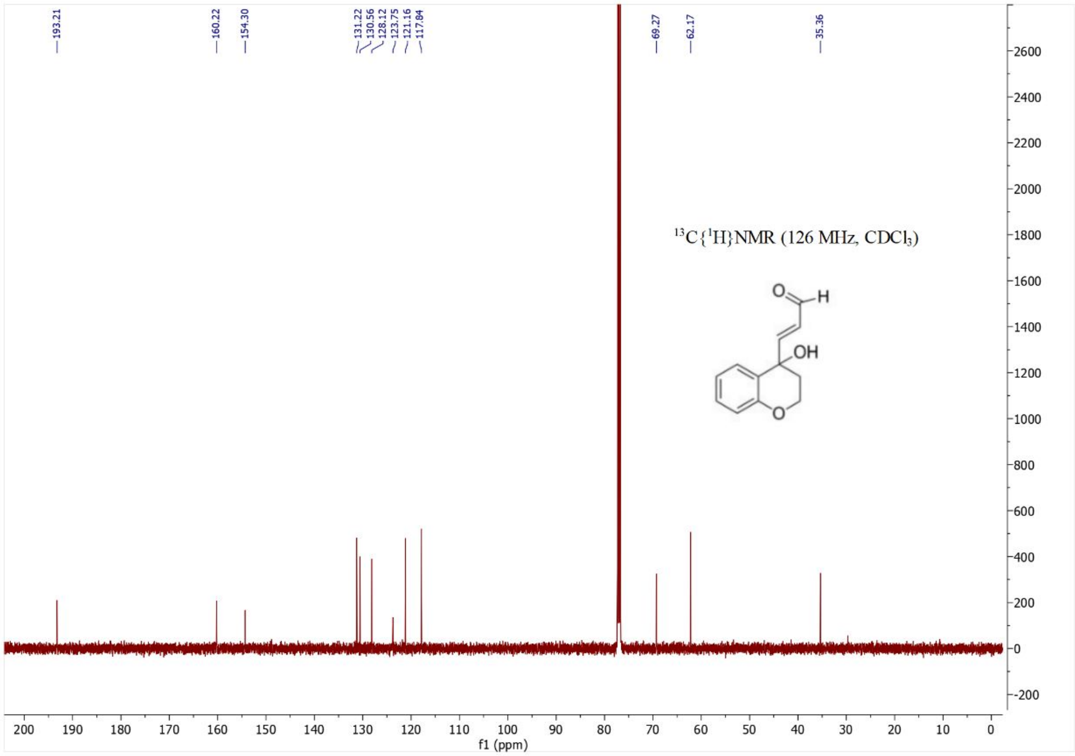

(*E*)-4-hydroxy-4-phenylhex-2-enal (7n)

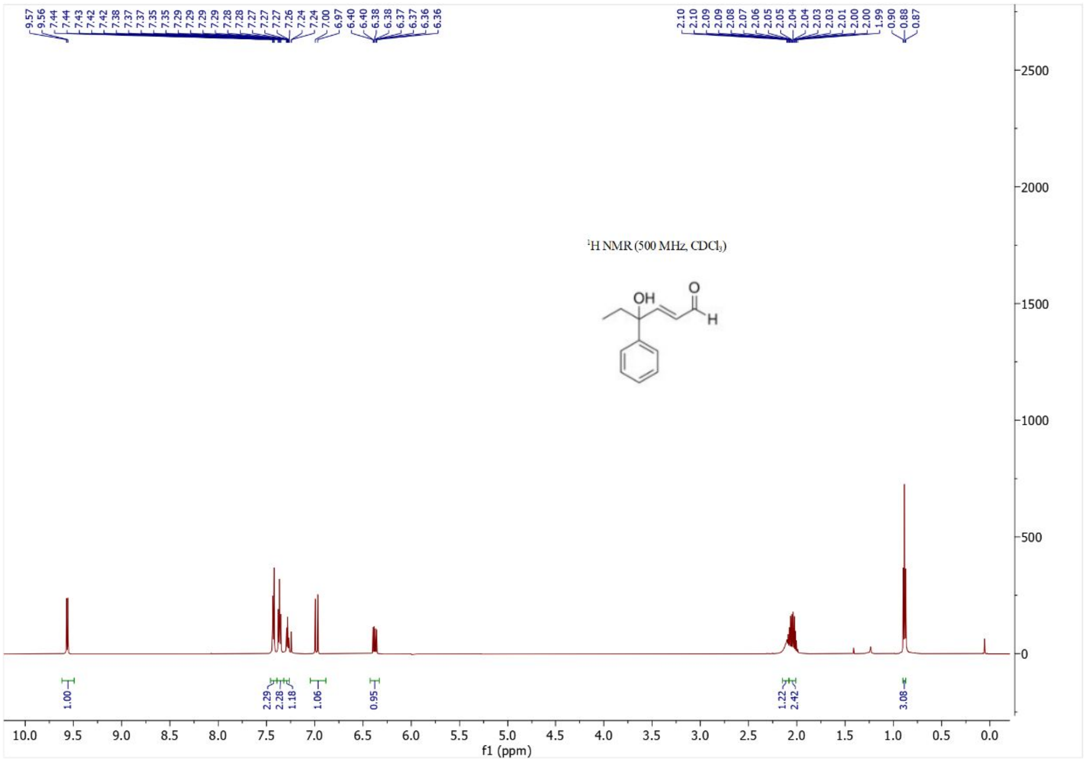

9.57
9.56
7.44
7.43
7.42
7.42
7.38
7.37
7.37
7.35
7.29
7.29
7.29
7.28
7.28
7.27
7.27
7.26
7.24
7.24
7.00
6.97
6.40
6.40
6.38
6.38
6.37
6.37
6.36
6.36
2.10
2.10
2.09
2.09
2.08
2.07
2.06
2.05
2.05
2.04
2.04
2.03
2.03
2.01
2.00
2.00
1.99
0.90
0.88
0.87
^{1}H NMR (500 MHz, CDCl$_3$)
OH
O
H
1.00
2.29
2.28
1.18
1.06
0.95
1.22
2.42
3.08
10.0 9.5 9.0 8.5 8.0 7.5 7.0 6.5 6.0 5.5 5.0 4.5 4.0 3.5 3.0 2.5 2.0 1.5 1.0 0.5 0.0
f1 (ppm)
2500
2000
1500
1000
500
0

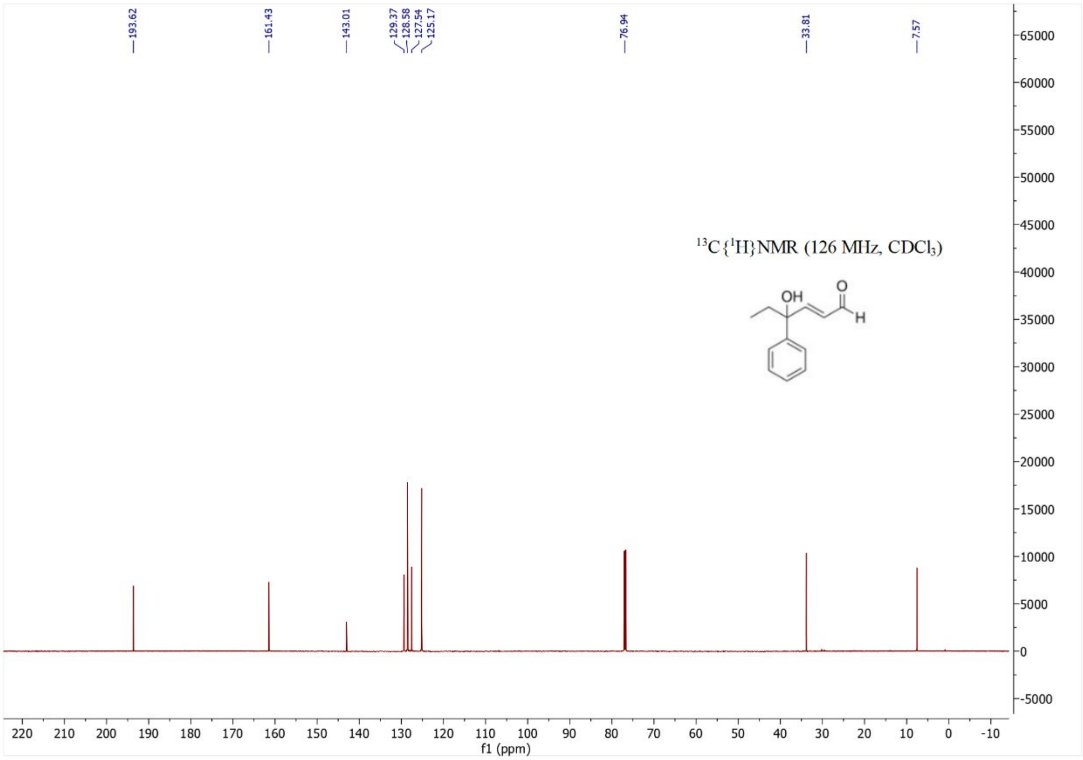

193.62
161.43
143.01
129.37
128.58
127.54
125.17
76.94
33.81
7.57
^{13}C{^{1}H}NMR (126 MHz, CDCl$_3$)
OH
O
H
65000
60000
55000
50000
45000
40000
35000
30000
25000
20000
15000
10000
5000
0
-5000
220 210 200 190 180 170 160 150 140 130 120 110 100 90 80 70 60 50 40 30 20 10 0 -10
f1 (ppm)

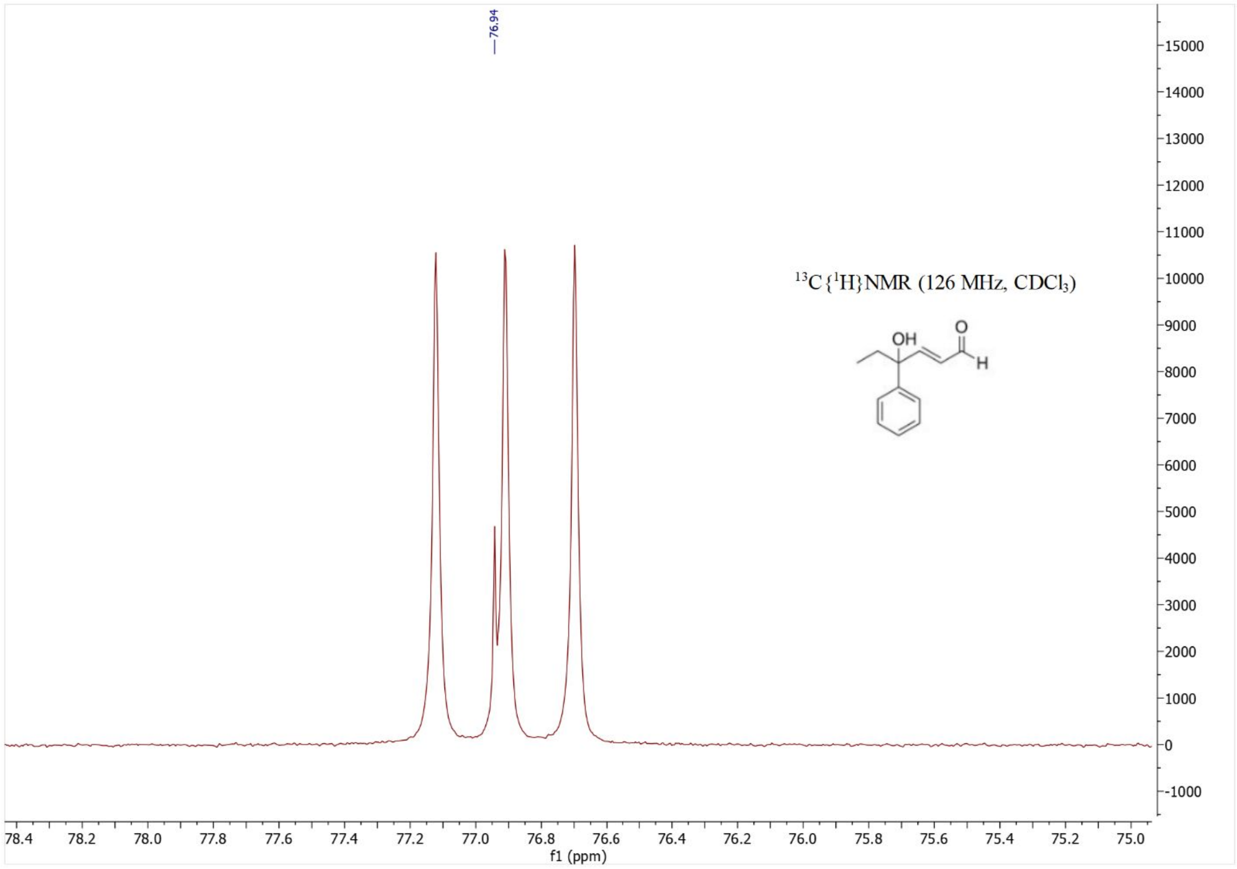
76.94
$^{13}C\{^1H\}$NMR (126 MHz, $CDCl_3$)
OH
O
H
15000
14000
13000
12000
11000
10000
9000
8000
7000
6000
5000
4000
3000
2000
1000
0
-1000
78.4
78.2
78.0
77.8
77.6
77.4
77.2
77.0
76.8
76.6
76.4
76.2
76.0
75.8
75.6
75.4
75.2
75.0
f1 (ppm)

(2*E*,4*Z*)-4-((3*S*,10*R*,13*S*)-3-((tert-butyldimethylsilyl)oxy)-10,13-dimethyl-16-oxo-1,2,3,4,7,8,9,10,11,12,13,14,15,16-tetradecahydro-17H-cyclopenta[a]phenanthren-17-ylidene)pent-2-enal (19)

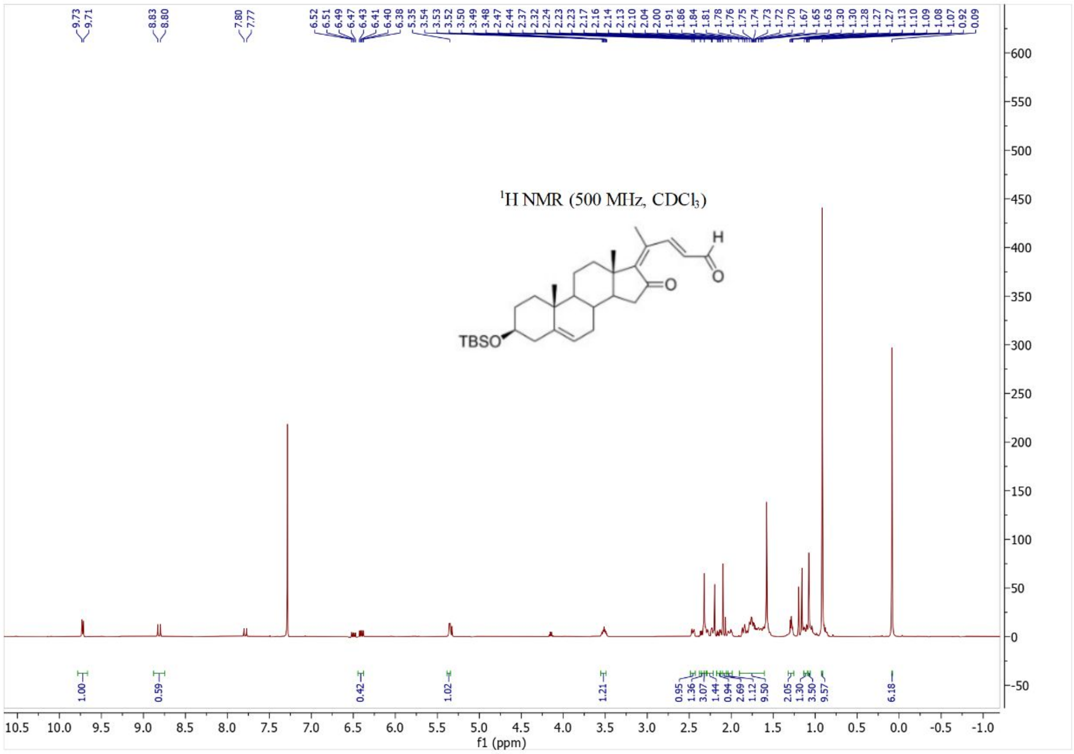

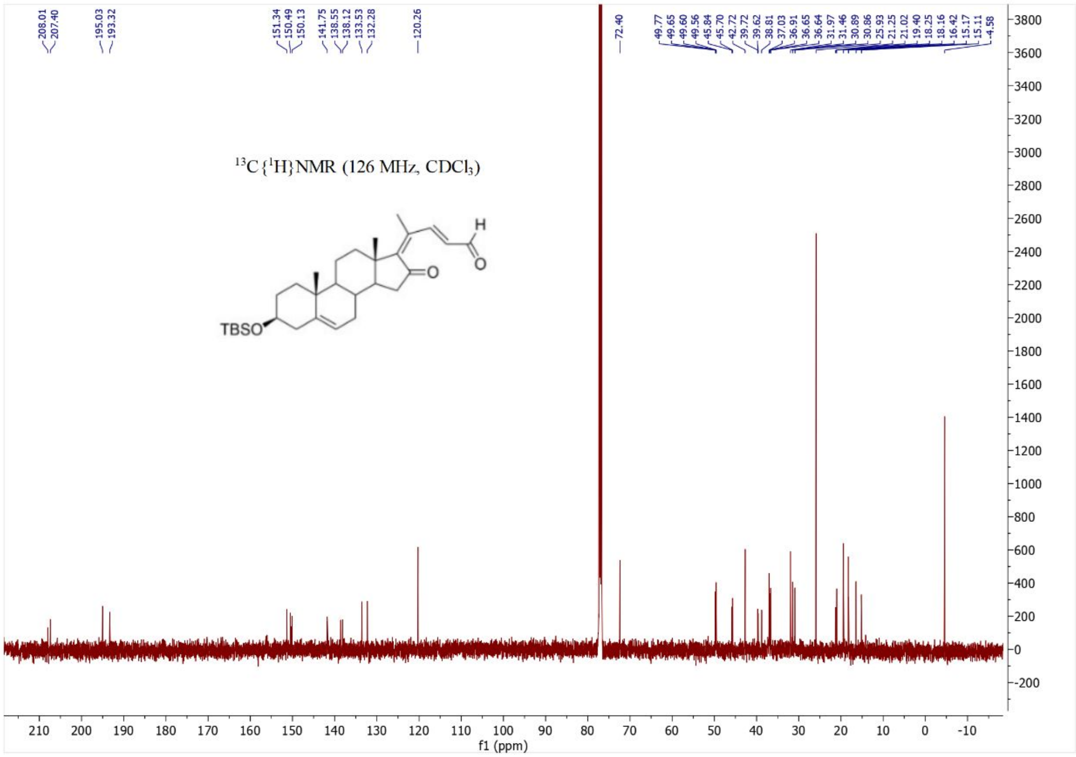

NOESY

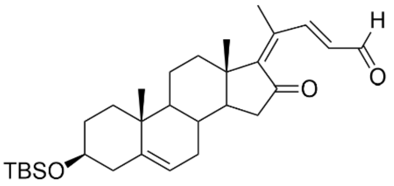

Major

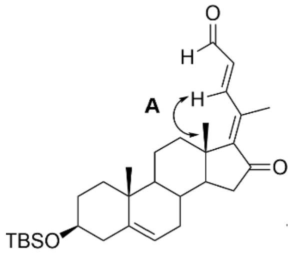

Minor

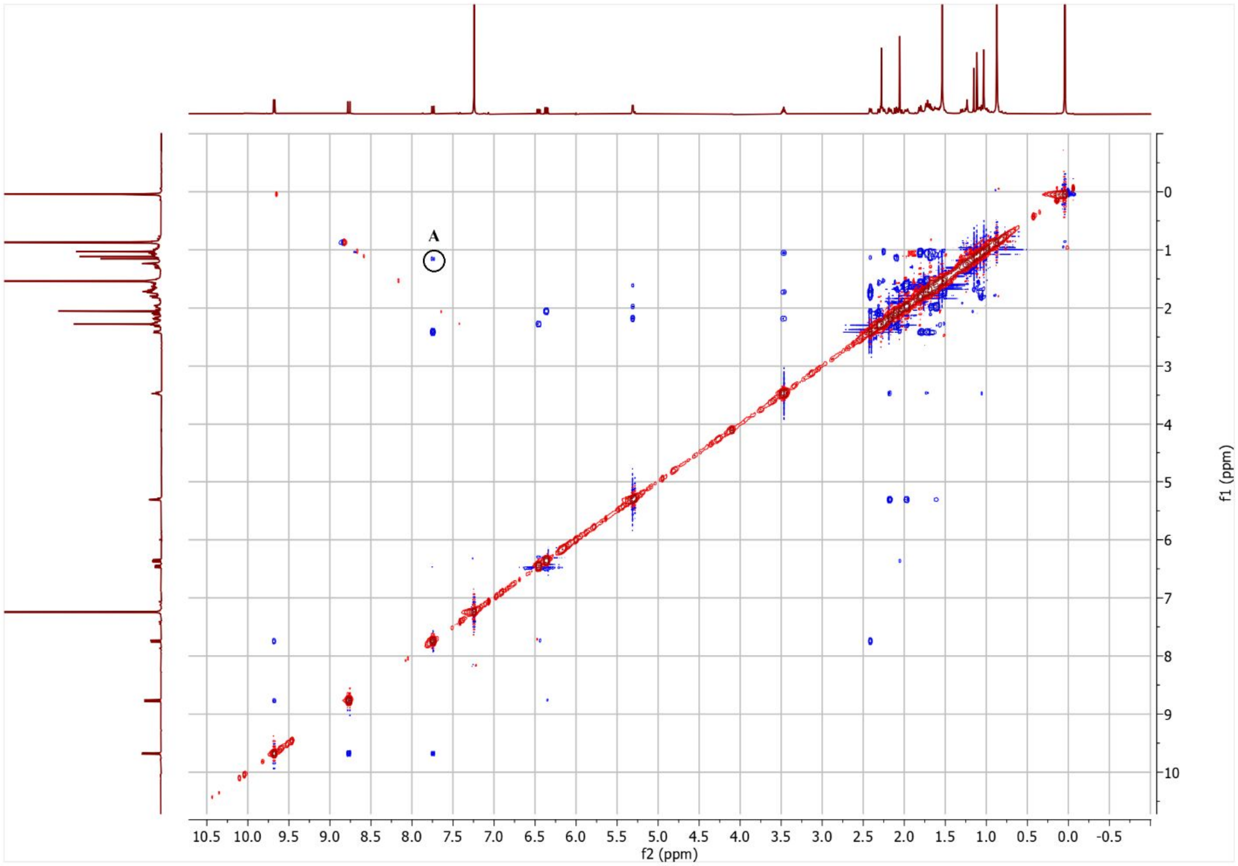

(*E*)-4-benzyl-5,5,5-trifluoro-4-hydroxypent-2-enal

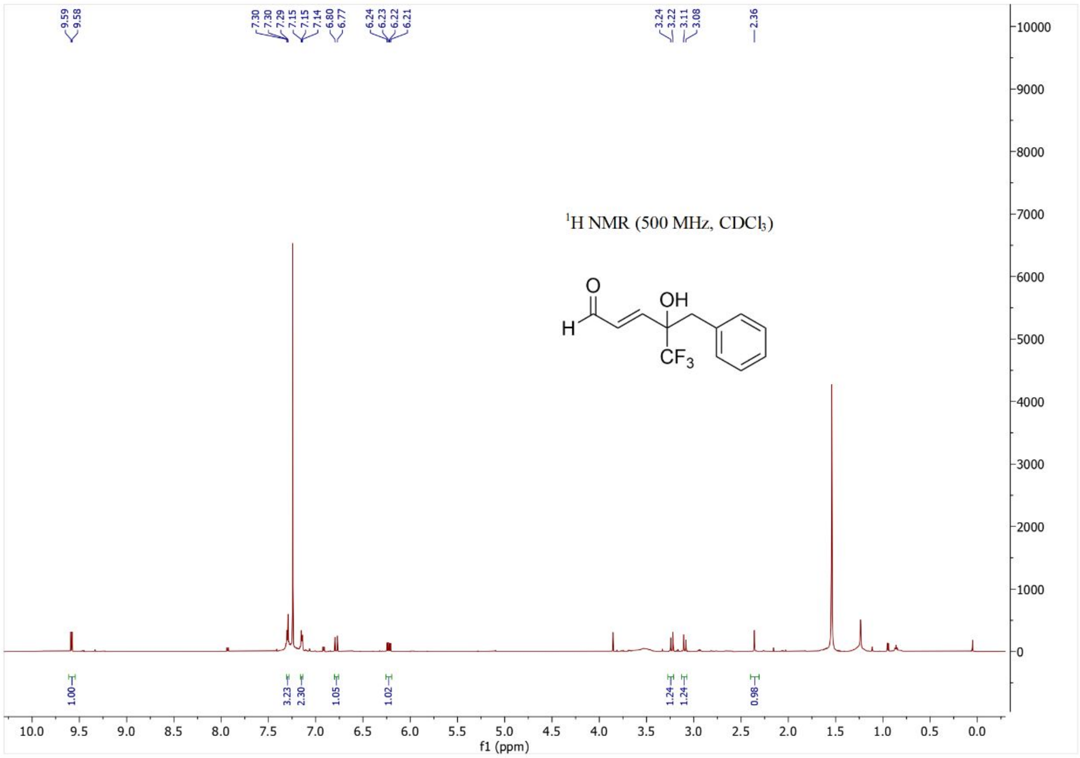

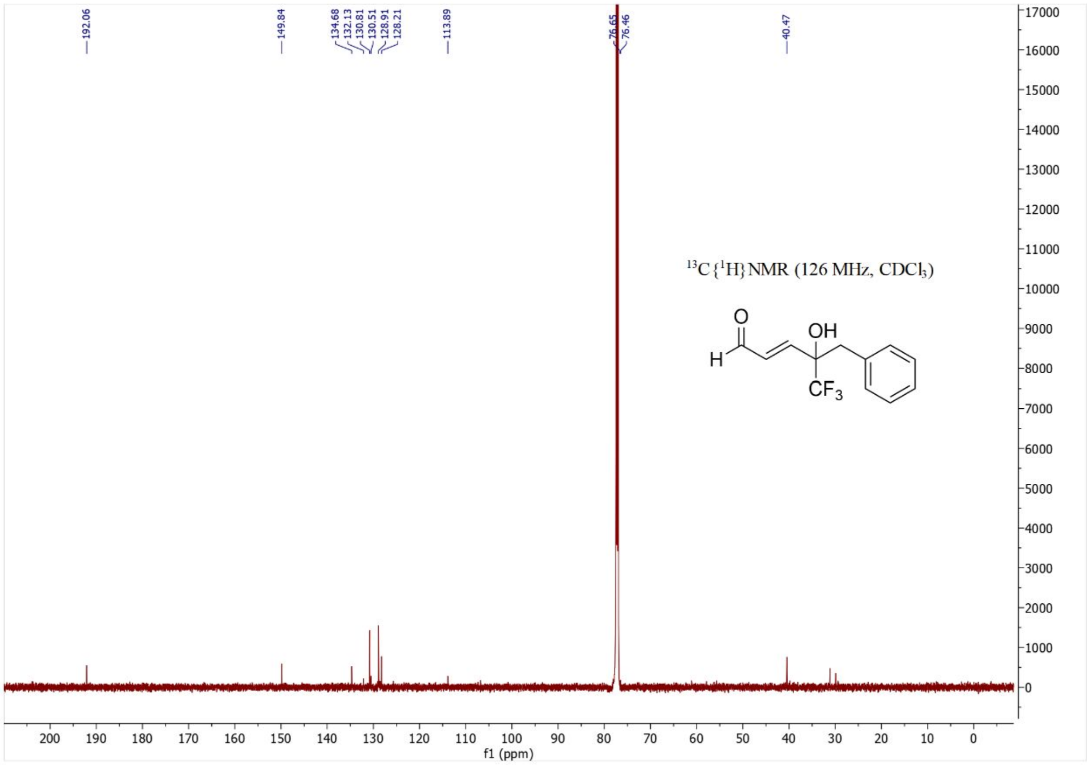

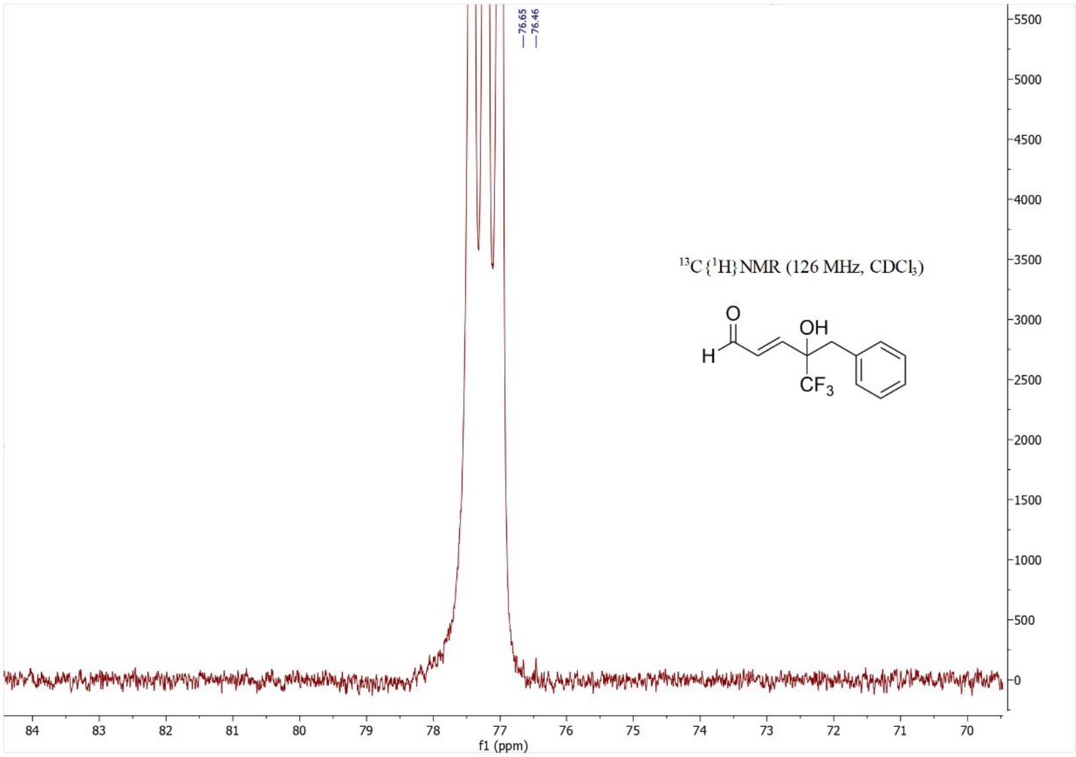
76.65
76.46
$^{13}C\{^1H\}$NMR (126 MHz, $CDCl_3$)
O
H
OH
CF_3
f1 (ppm)

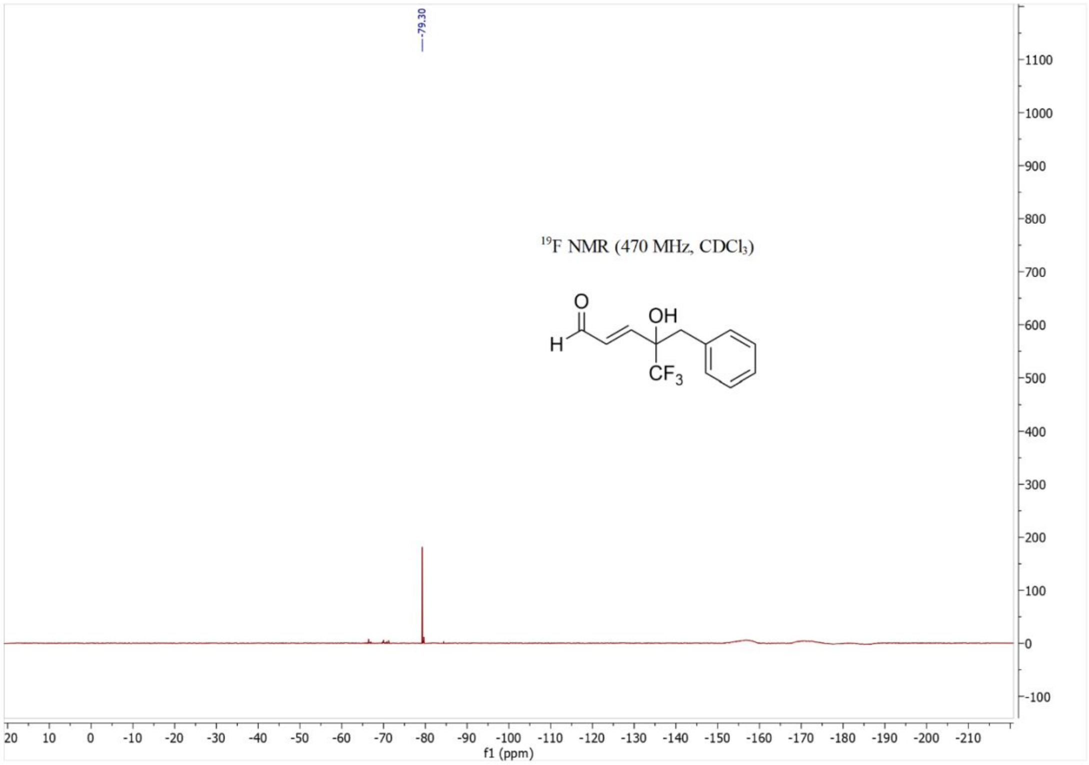

(*Z*)-5-phenylhex-4-en-2-one

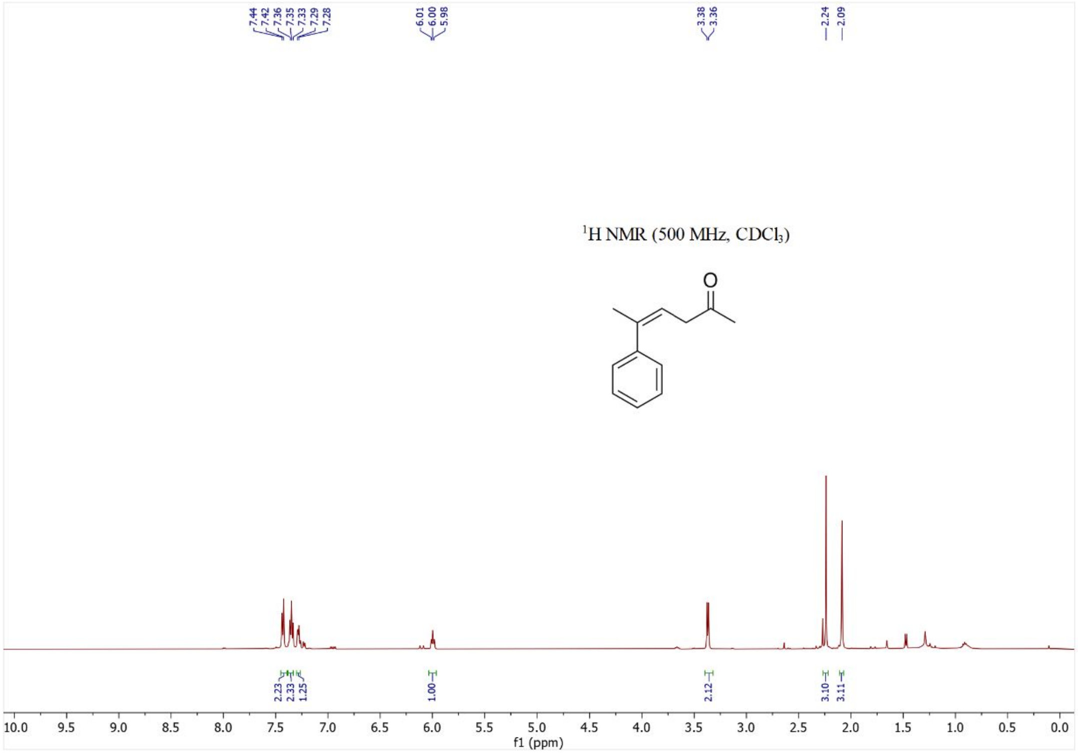

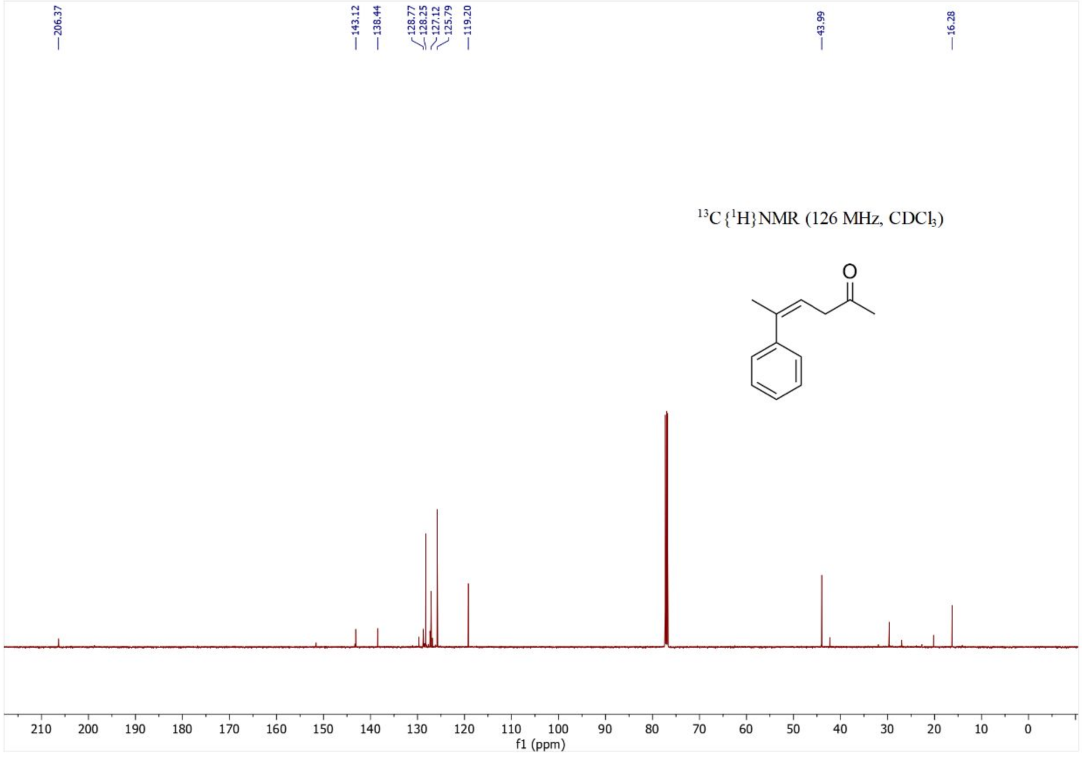

(*E*)-2-(2-hydroxy-3-phenylpropylidene)hexanal (9)

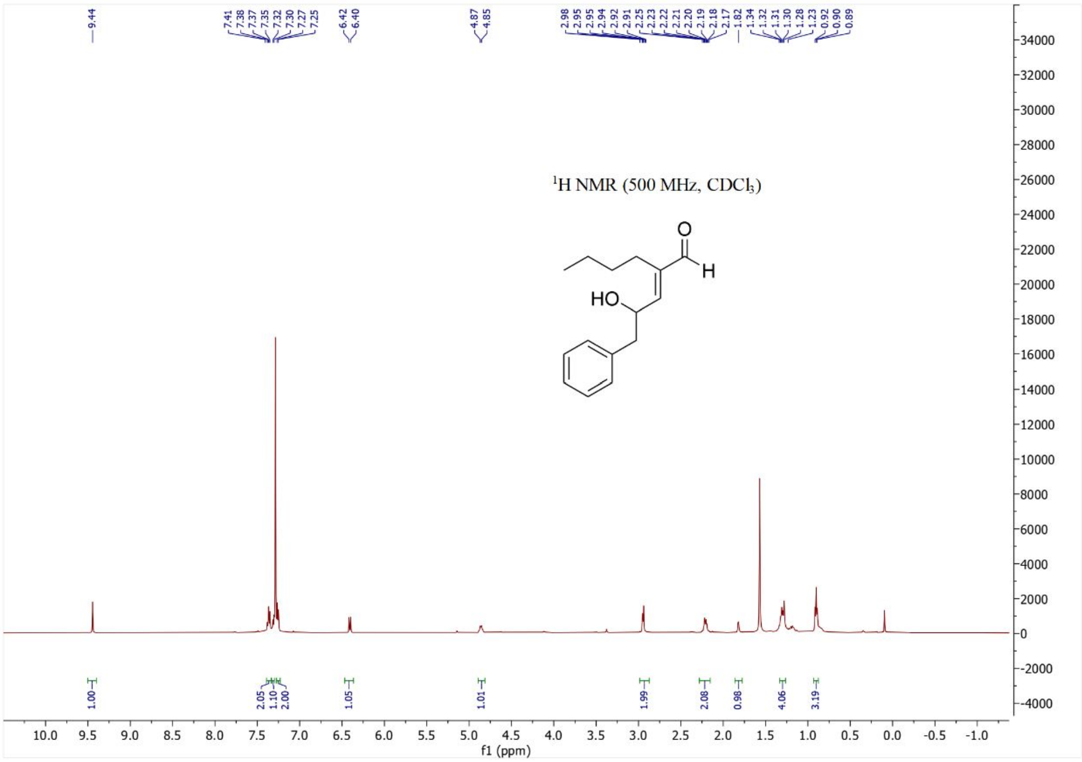

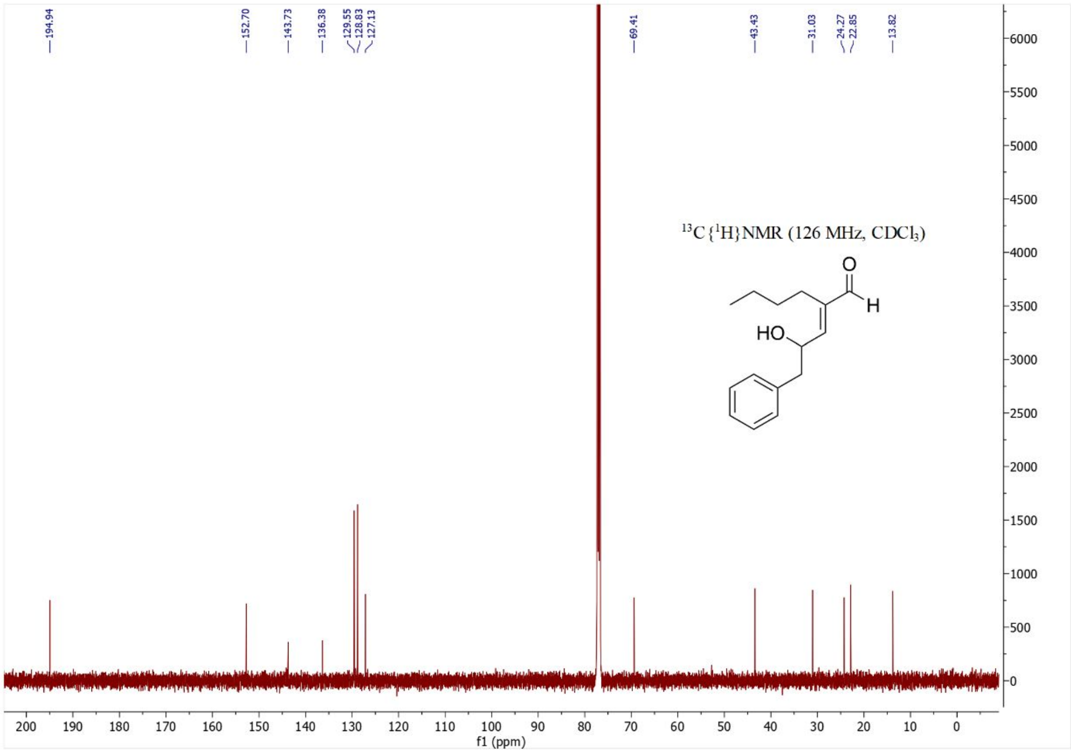

(*E*)-2-(2-oxo-3-phenylpropylidene)hexanal (10)

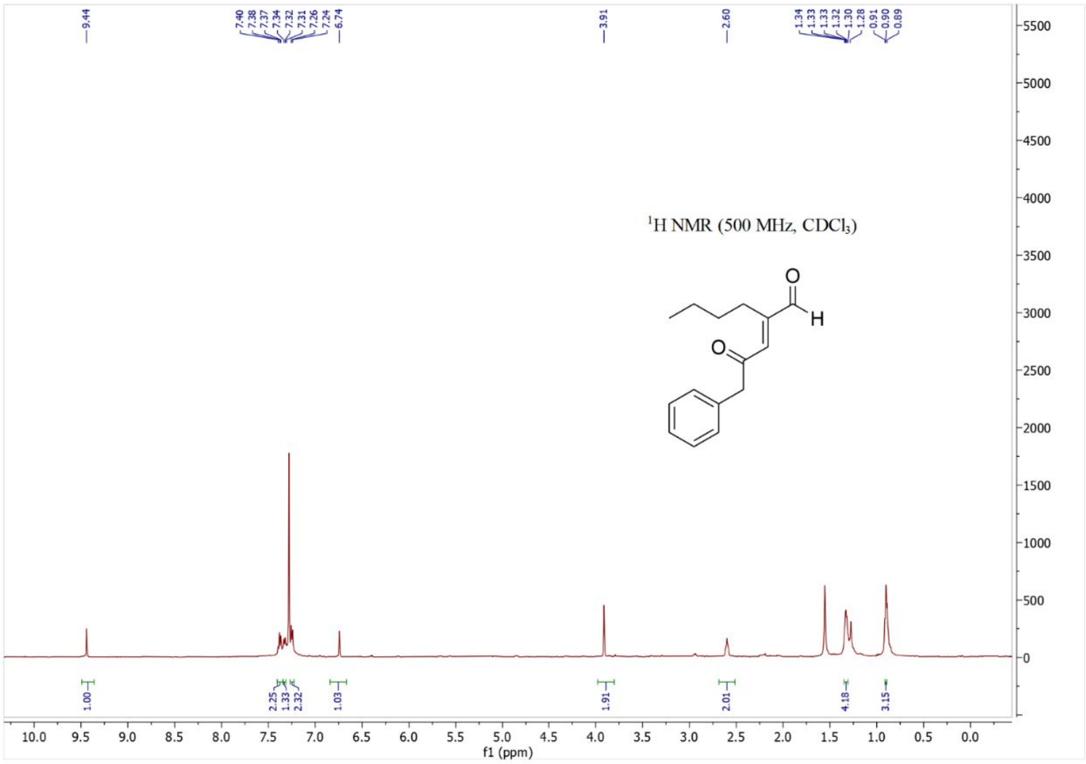

9.44
7.40
7.38
7.37
7.34
7.32
7.31
7.26
7.24
6.74
3.91
2.60
1.34
1.33
1.33
1.32
1.30
1.28
0.91
0.90
0.89
^{1}H NMR (500 MHz, CDCl$_3$)
O
H
O
1.00
2.25
1.33
2.32
1.03
1.91
2.01
4.18
3.15
10.0
9.5
9.0
8.5
8.0
7.5
7.0
6.5
6.0
5.5
5.0
4.5
4.0
3.5
3.0
2.5
2.0
1.5
1.0
0.5
0.0
f1 (ppm)
5500
5000
4500
4000
3500
3000
2500
2000
1500
1000
500
0

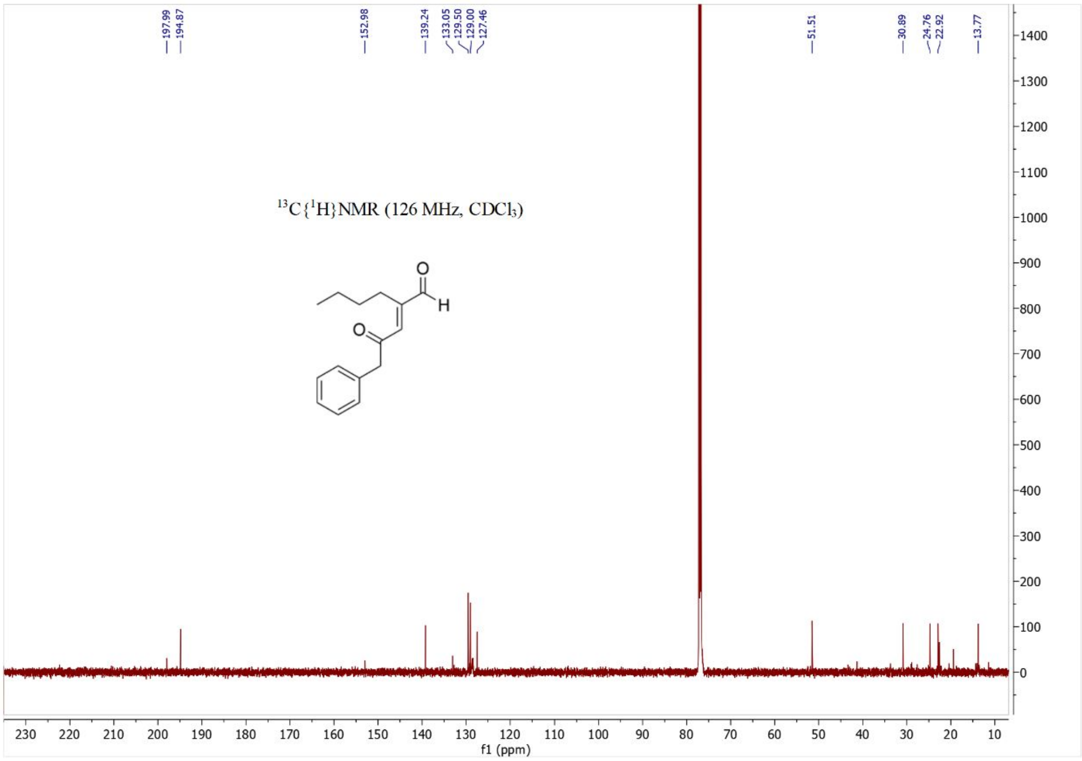

(*E*)-4-hydroperoxy-4-phenylpent-2-enal (6a-H)

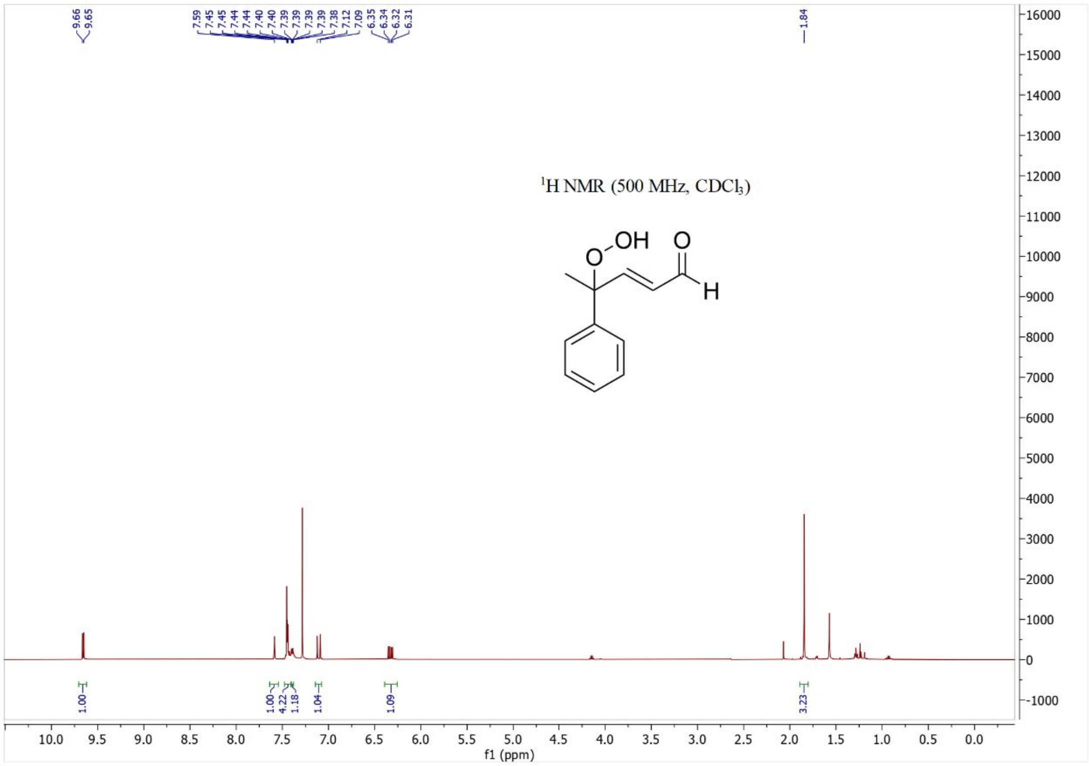

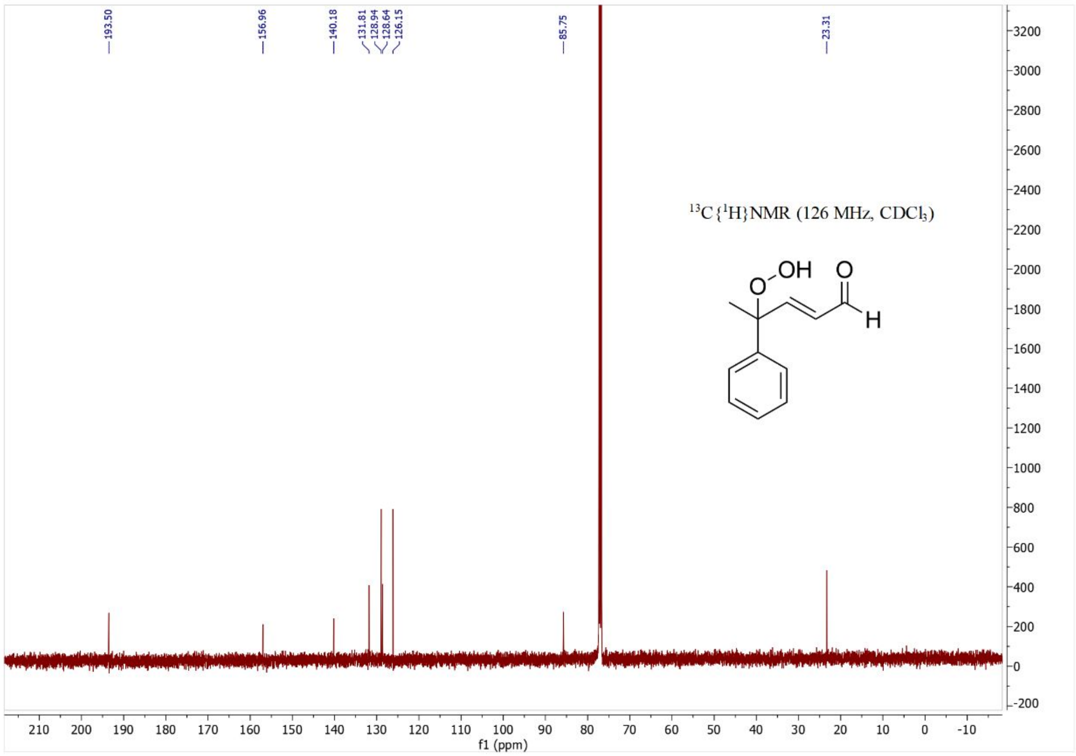

193.50
156.96
140.18
131.81
128.94
128.64
126.15
85.75
23.31
$^{13}C\{^1H\}$NMR (126 MHz, CDCl$_3$)
OH
O
O
H
f1 (ppm)

Acetophenone (17a)

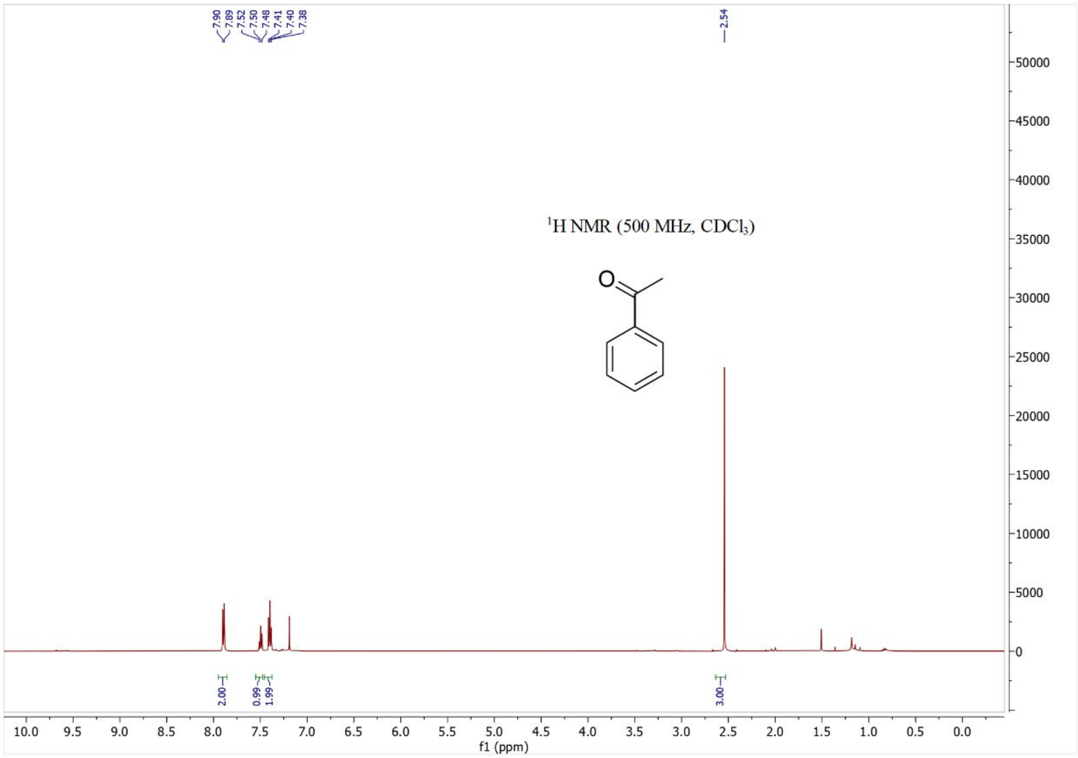

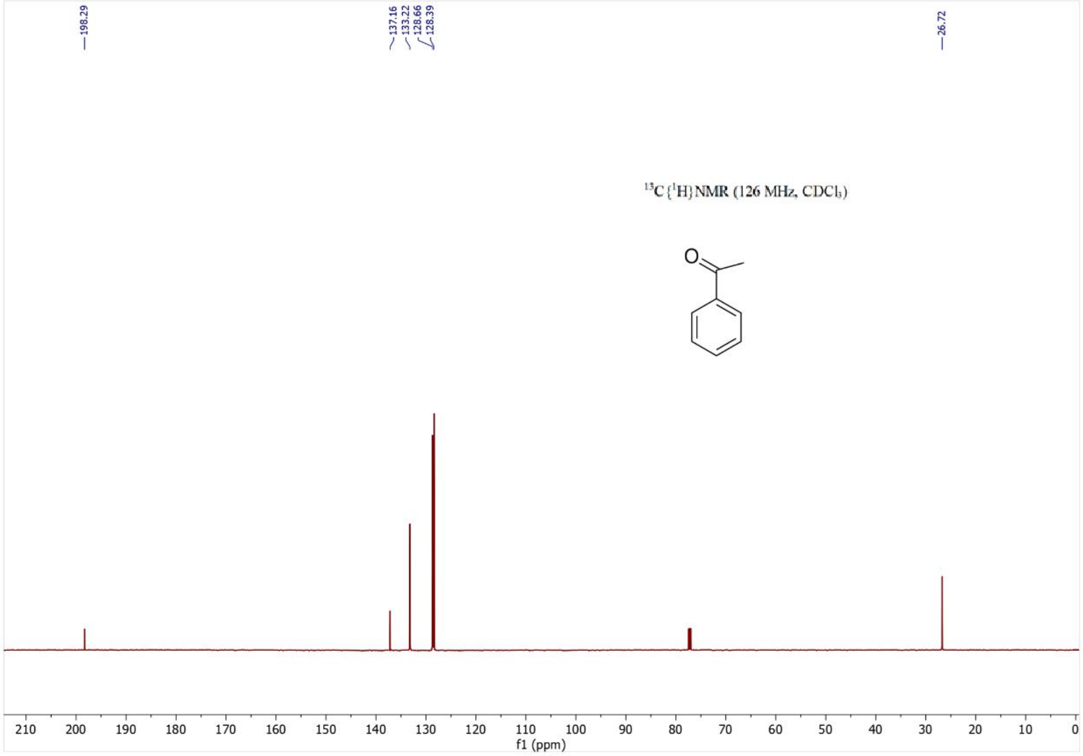

(*E*)-3-(pyrrolidin-1-yl)acrylaldehyde (16)

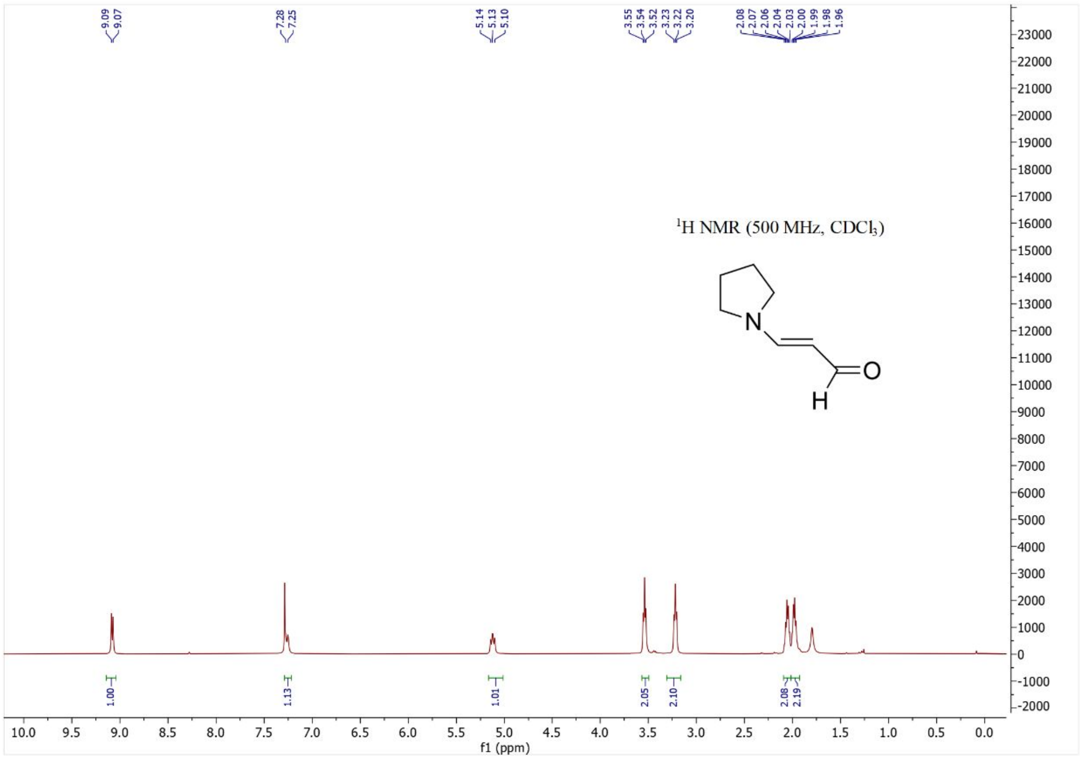

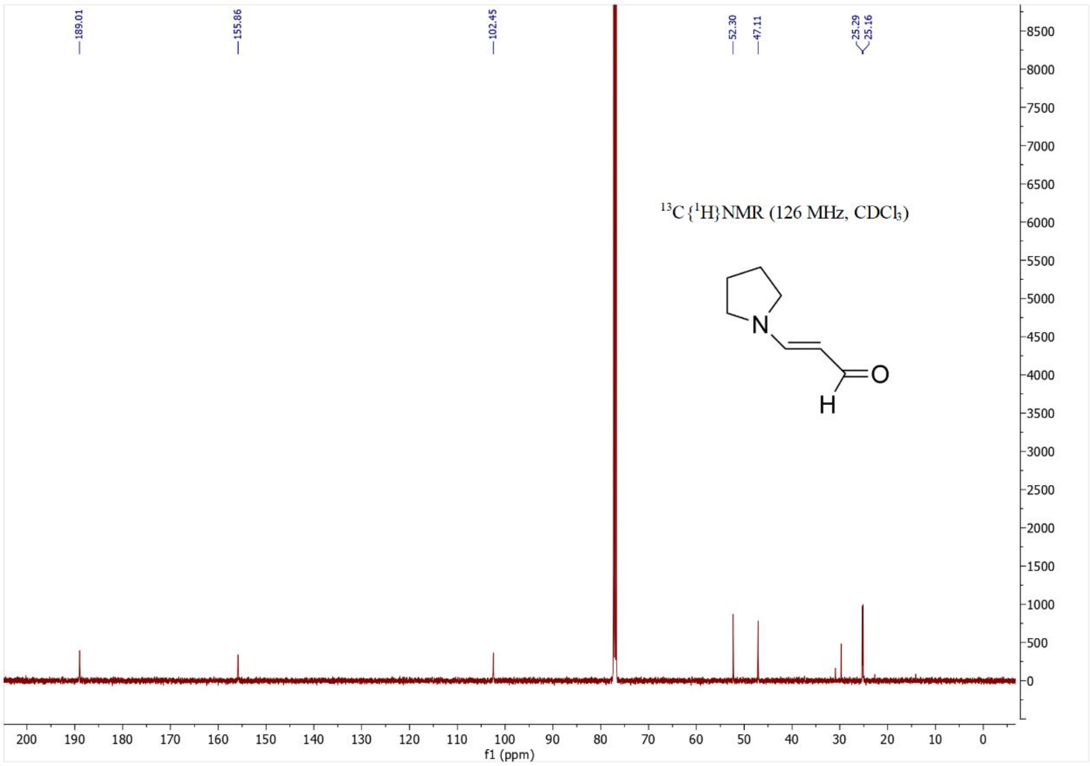

1-4-phenylpenta-1,3-dien-1-yl)pyrrolidine (18a)

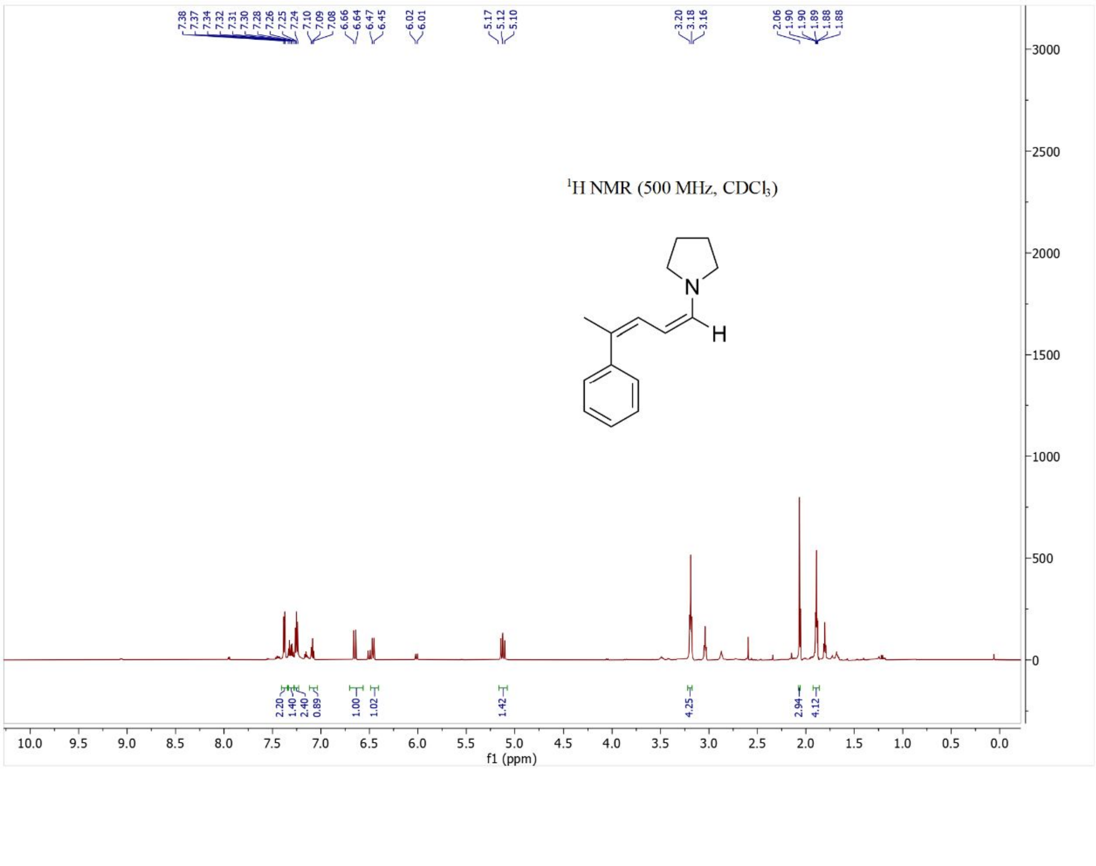

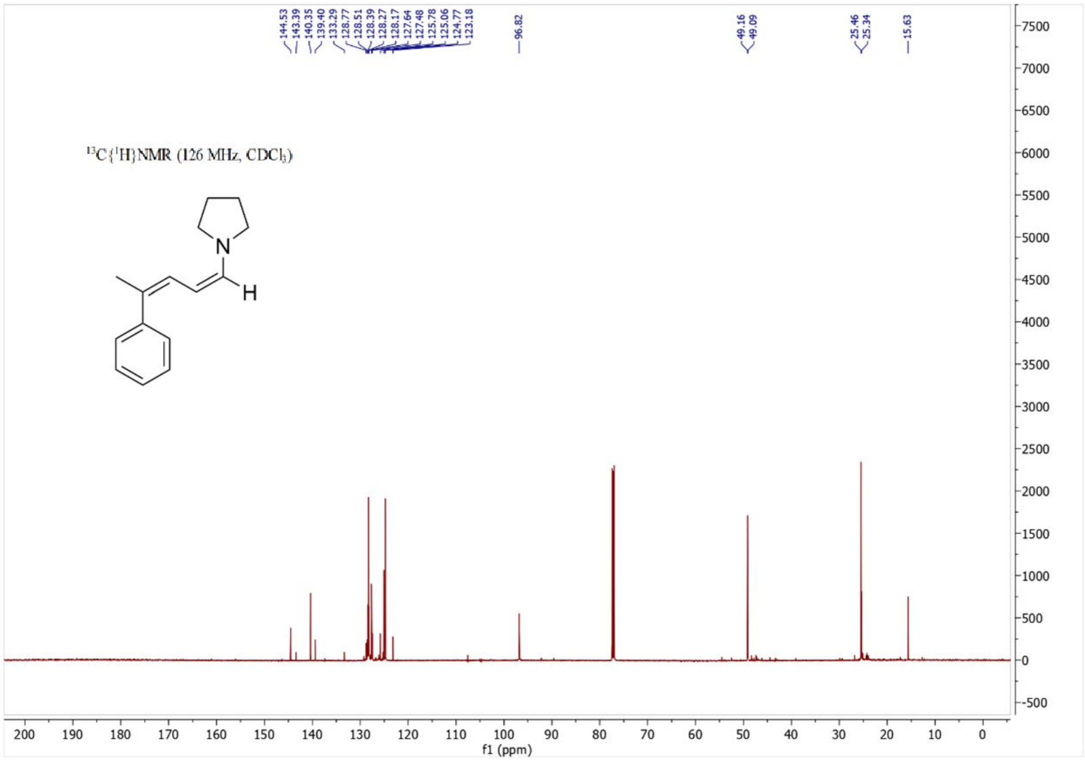
144.53
143.39
140.35
139.40
133.29
128.77
128.51
128.39
128.27
128.17
127.64
127.48
125.78
125.06
124.77
123.18
96.82
49.16
49.09
25.46
25.34
15.63
^{13}C{^{1}H}NMR (126 MHz, CDCl$_3$)
N
H
f1 (ppm)

NOESY

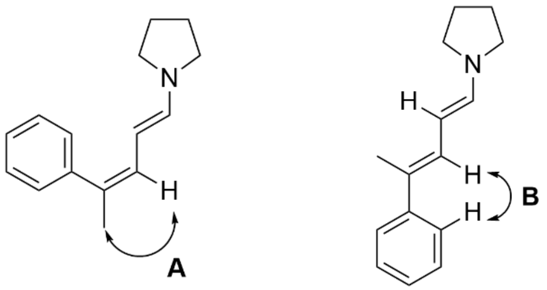

Minor **Major**

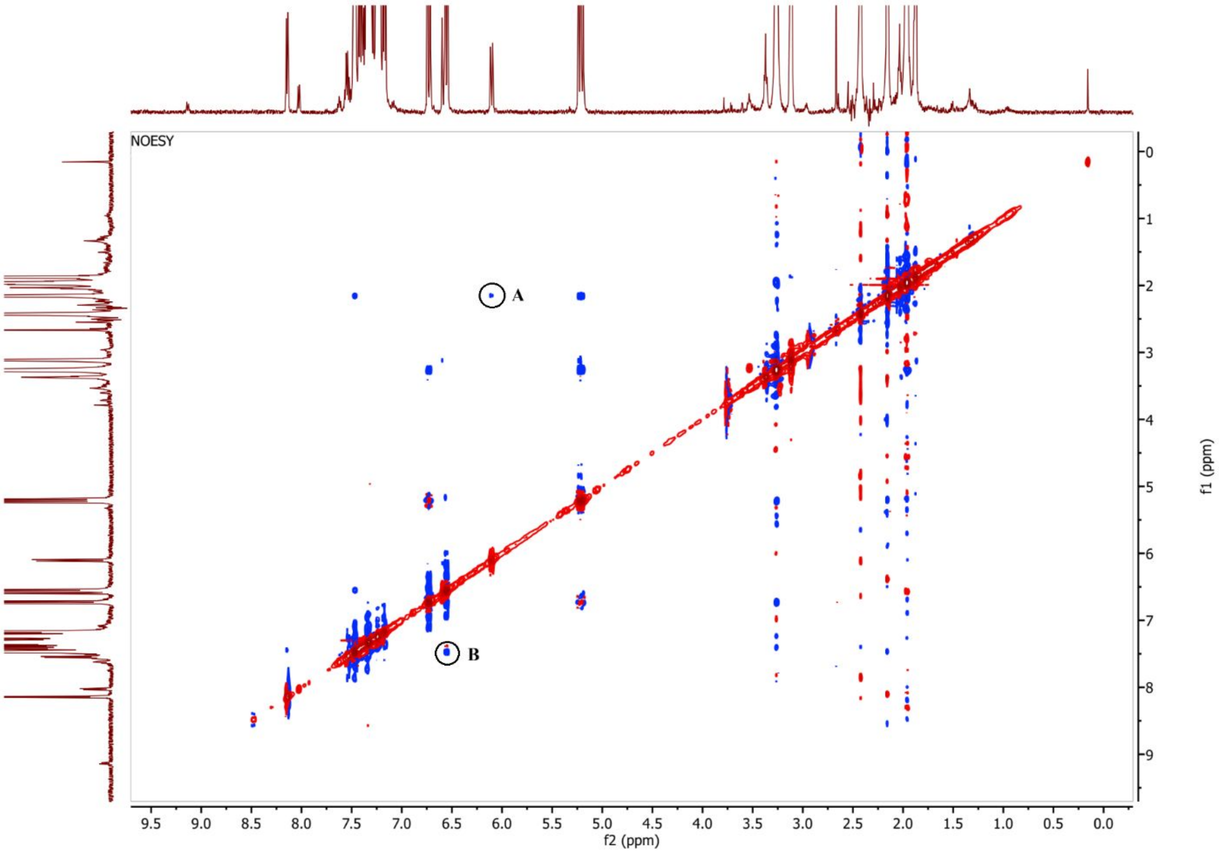

1-(4-(4-fluorophenyl)penta-1,3-dien-1-yl)pyrrolidine (18b)

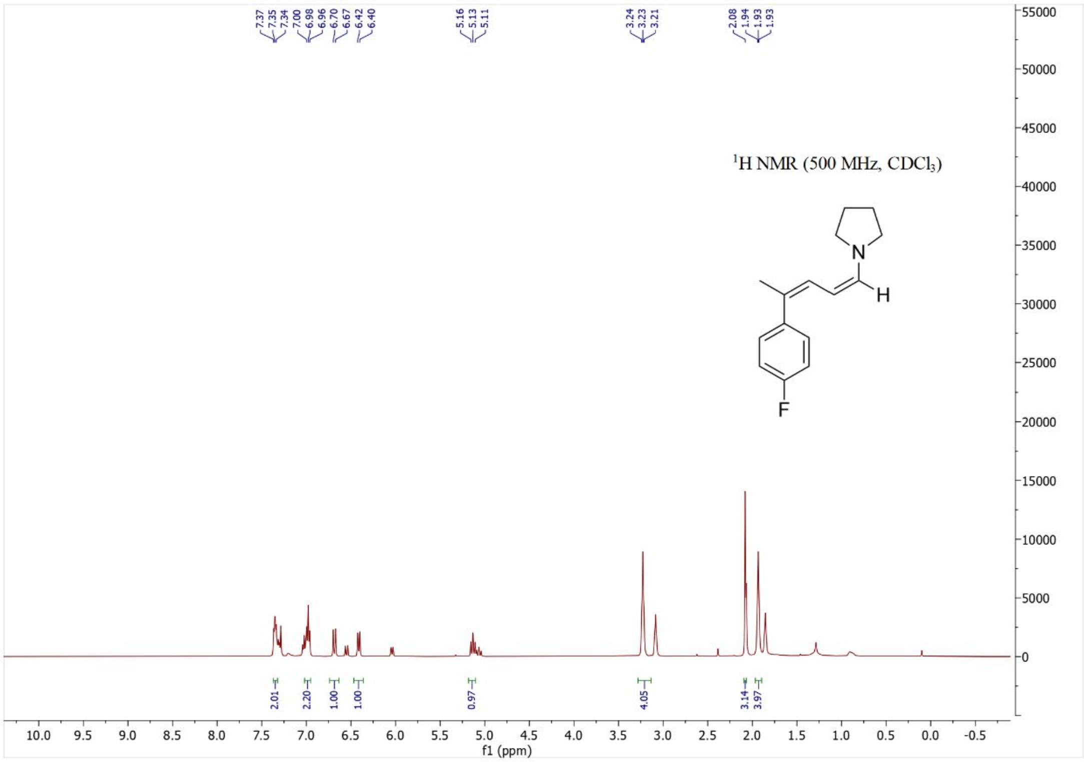

^{1}H NMR (500 MHz, CDCl$_3$)
N
H
F
f1 (ppm)

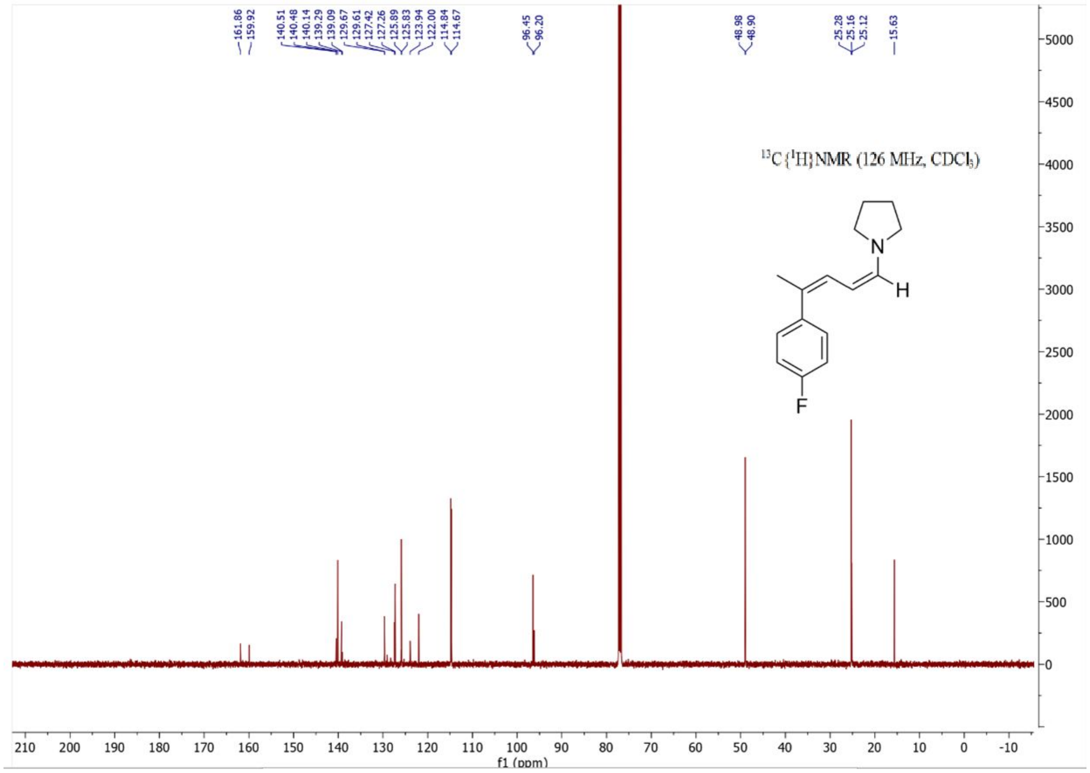

^{13}C{^{1}H}NMR (126 MHz, CDCl$_3$)
N
H
F
f1 (ppm)

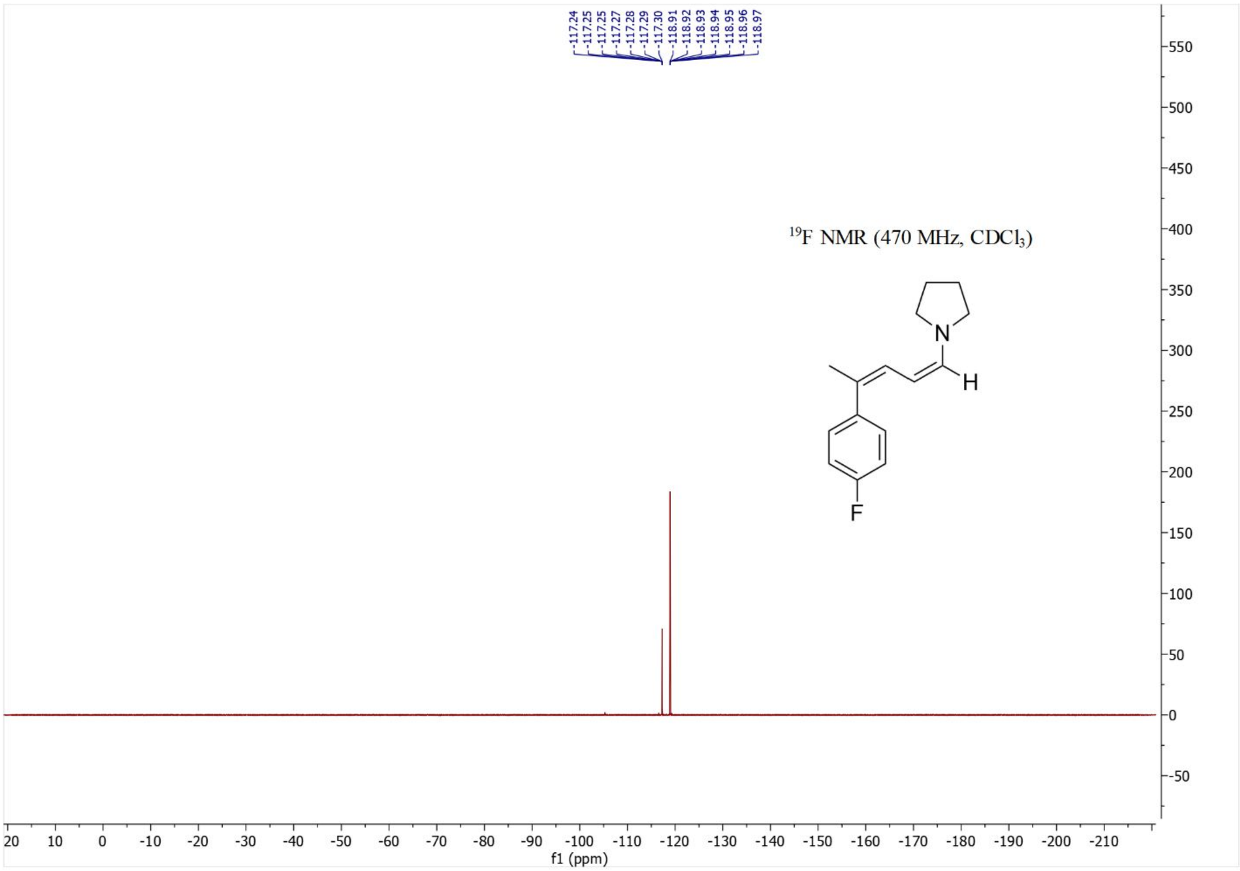
-117.24
-117.25
-117.25
-117.27
-117.28
-117.29
-117.30
-118.91
-118.92
-118.93
-118.94
-118.95
-118.96
-118.97
^{19}F NMR (470 MHz, CDCl$_3$)
N
H
F
f1 (ppm)

Boivinianin A

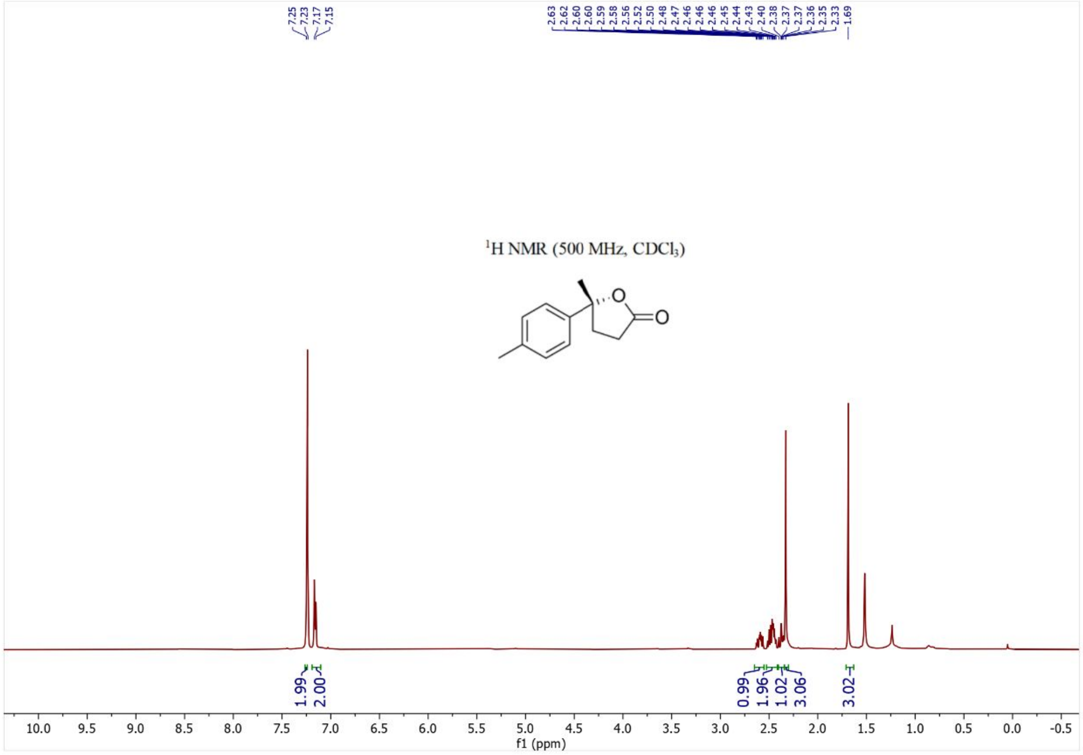

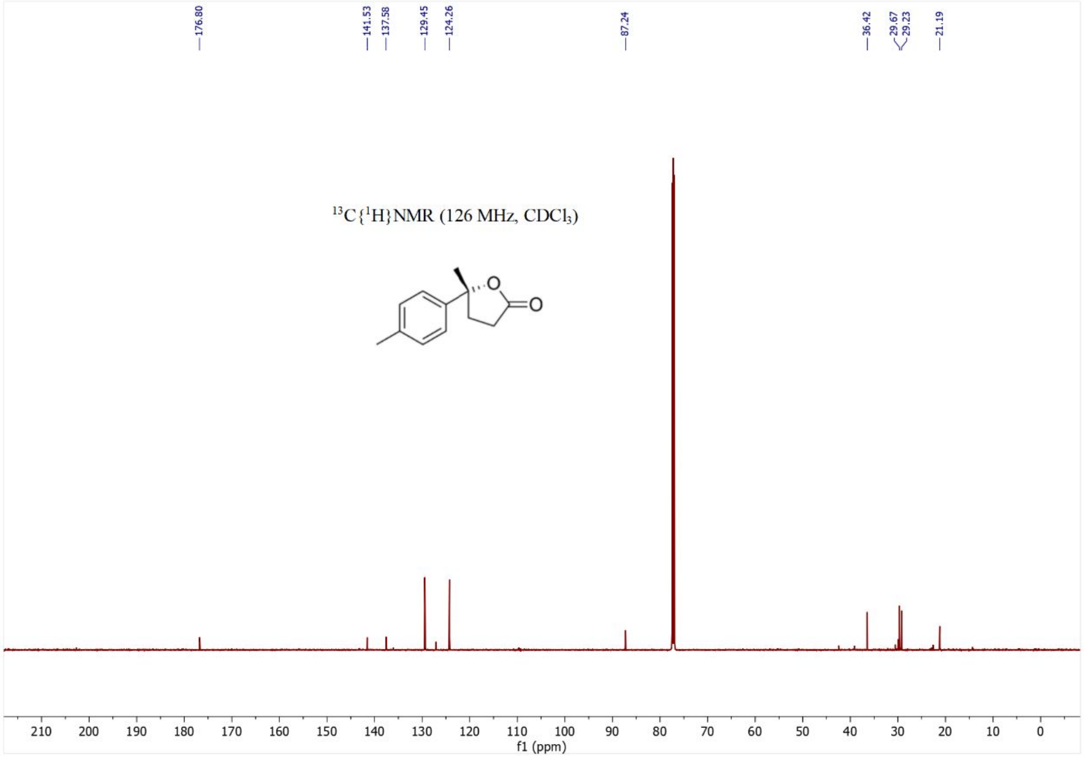

XI. Spectroscopic data (HPLC)

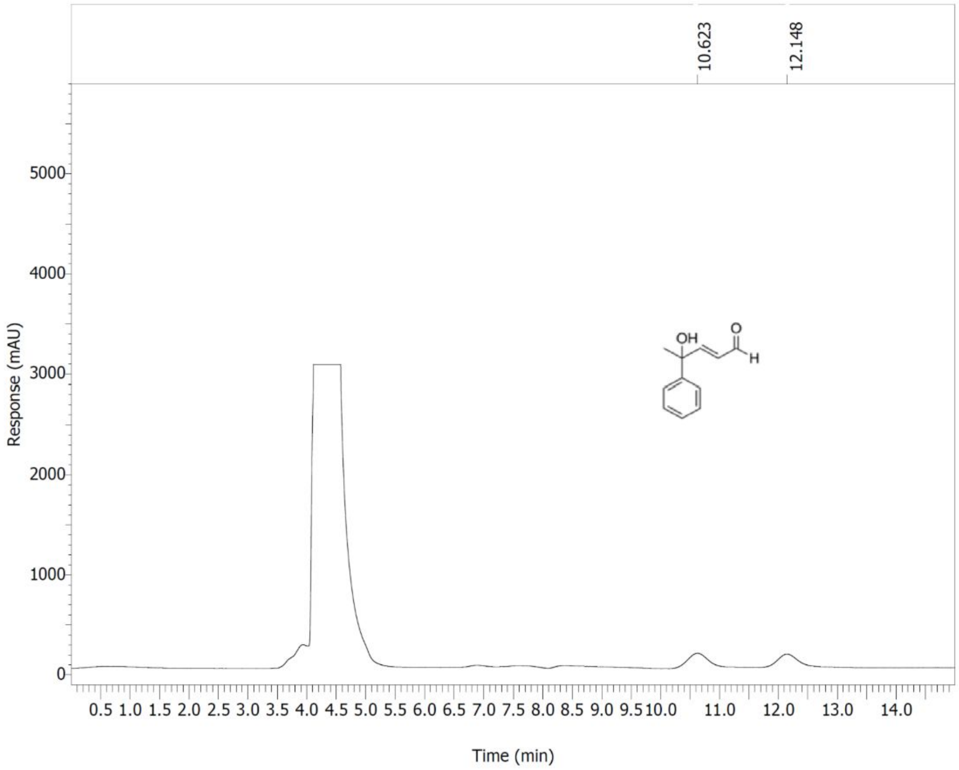

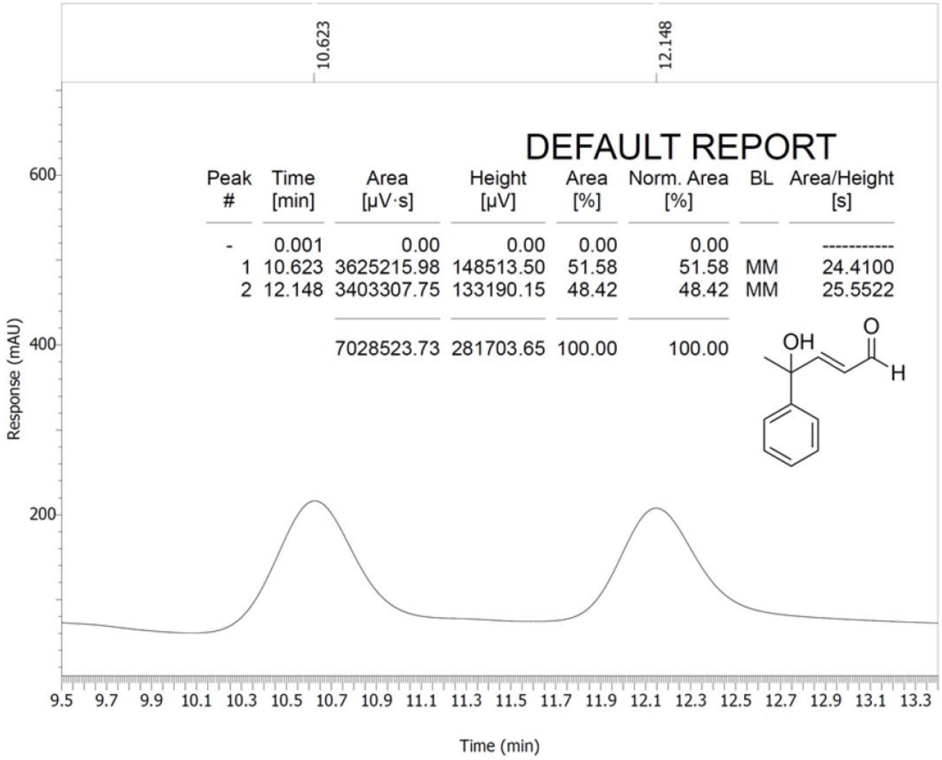

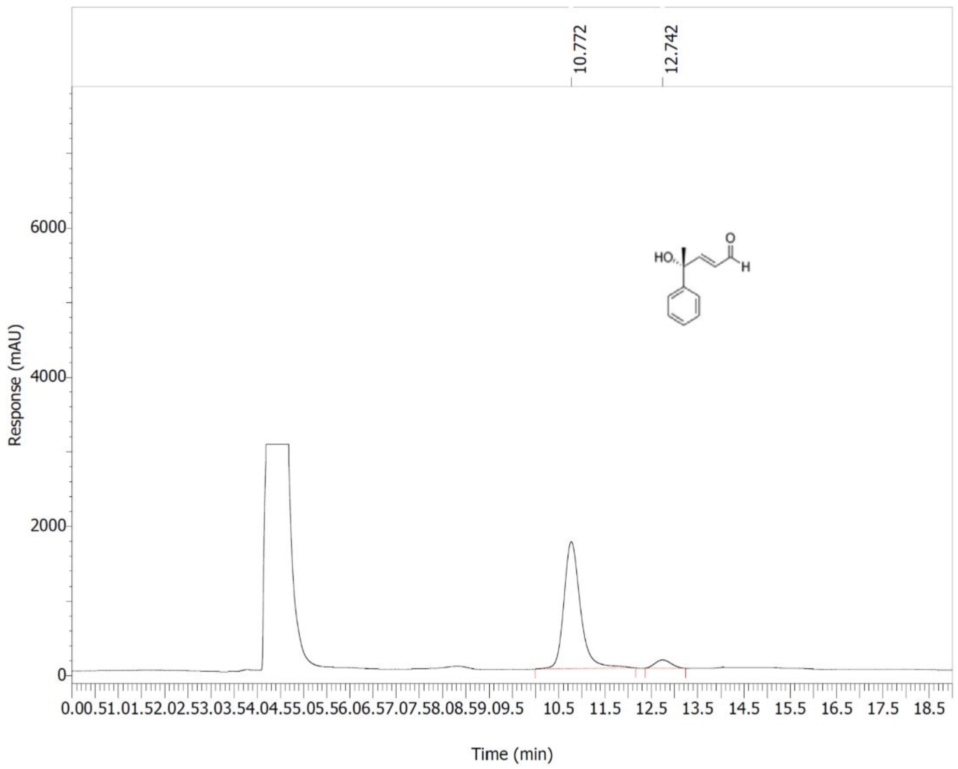

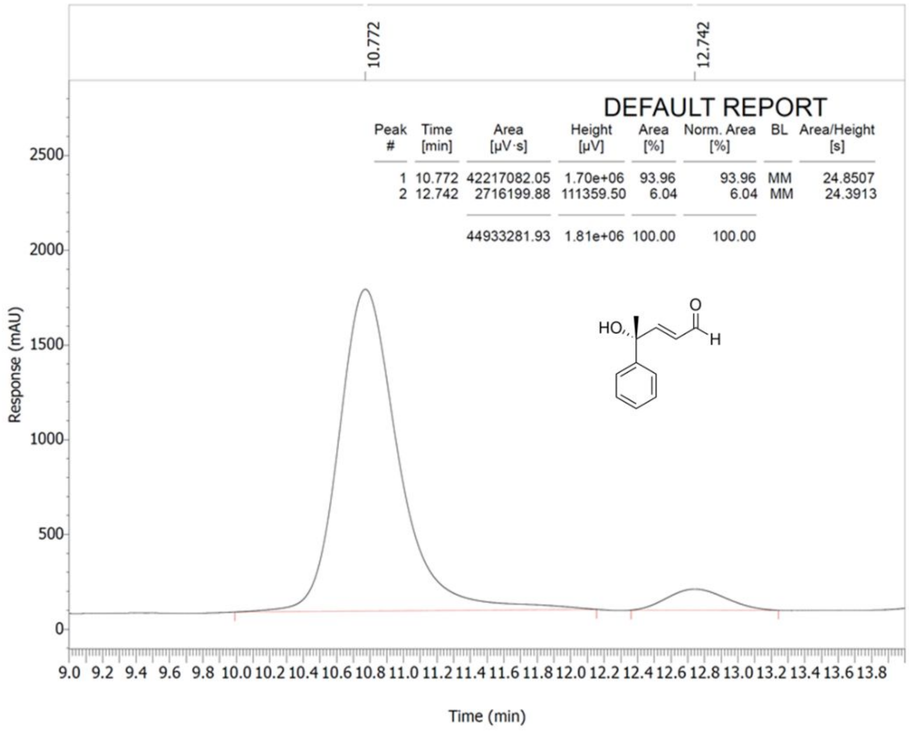

Peak #	Time [min]	Area [µV·s]	Height [µV]	Area [%]	Norm. Area [%]	BL	Area/Height [s]
1	10.772	42217082.05	1.70e+06	93.96	93.96	MM	24.8507
2	12.742	2716199.88	111359.50	6.04	6.04	MM	24.3913
		44933281.93	1.81e+06	100.00	100.00		

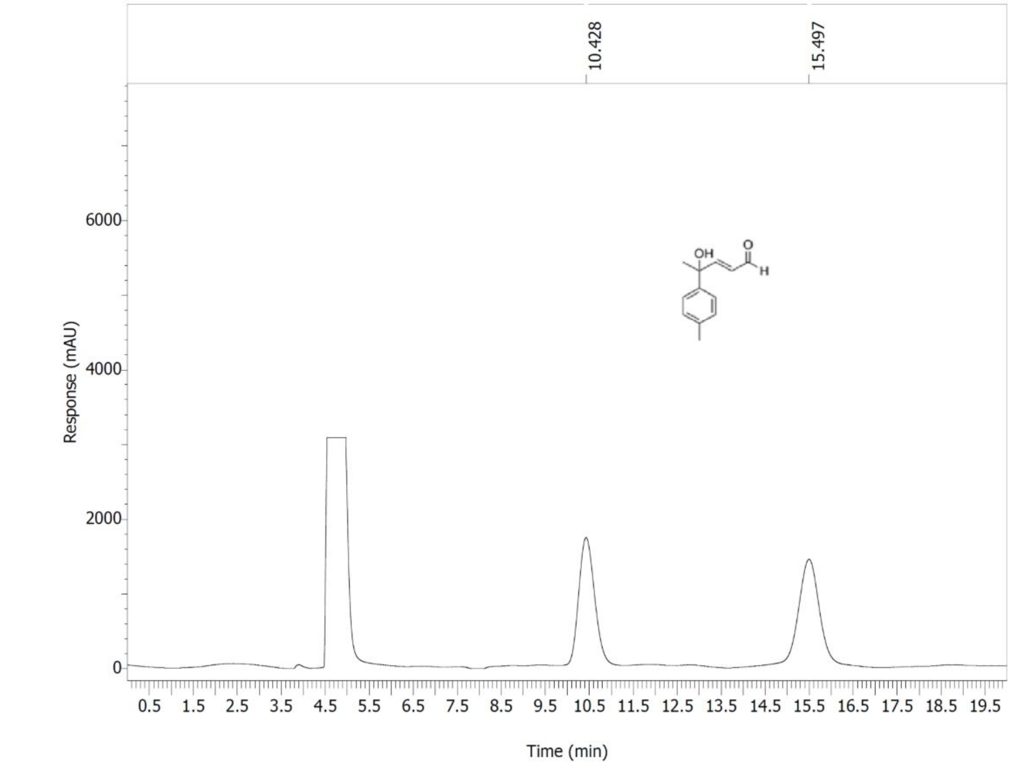

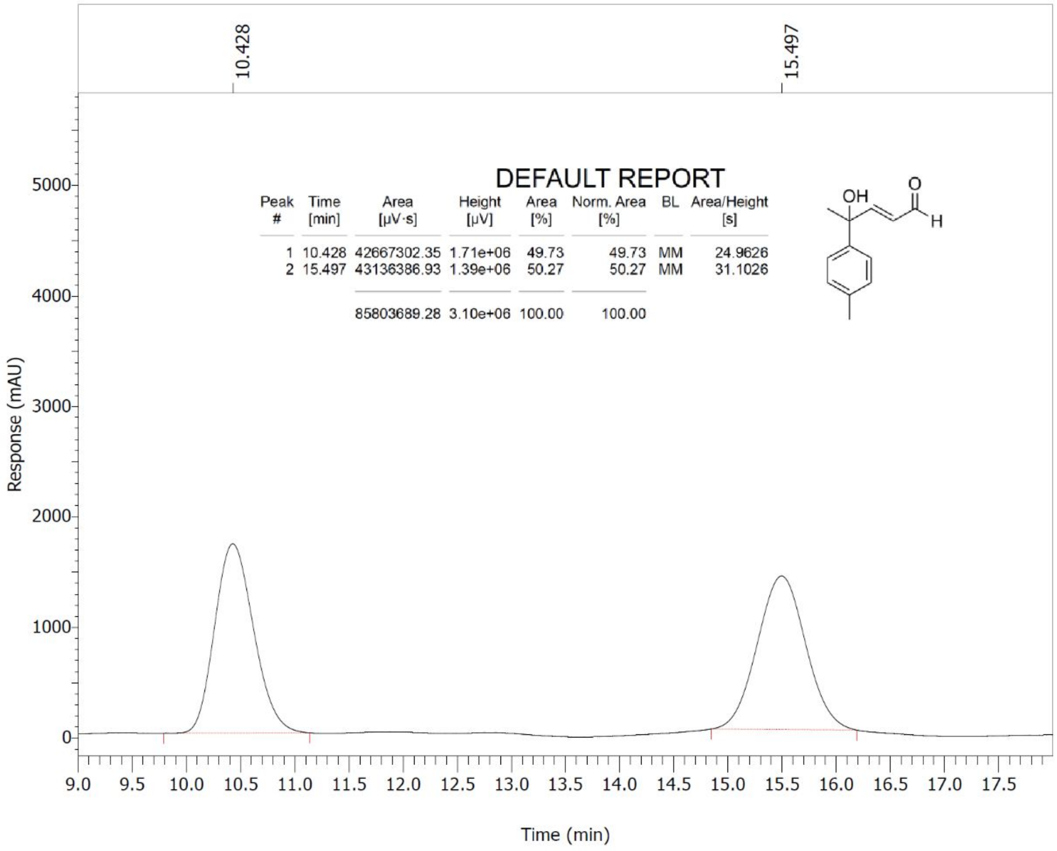

DEFAULT REPORT

Peak #	Time [min]	Area [µV·s]	Height [µV]	Area [%]	Norm. Area [%]	BL	Area/Height [s]
1	10.428	42667302.35	1.71e+06	49.73	49.73	MM	24.9626
2	15.497	43136386.93	1.39e+06	50.27	50.27	MM	31.1026
		85803689.28	3.10e+06	100.00	100.00		

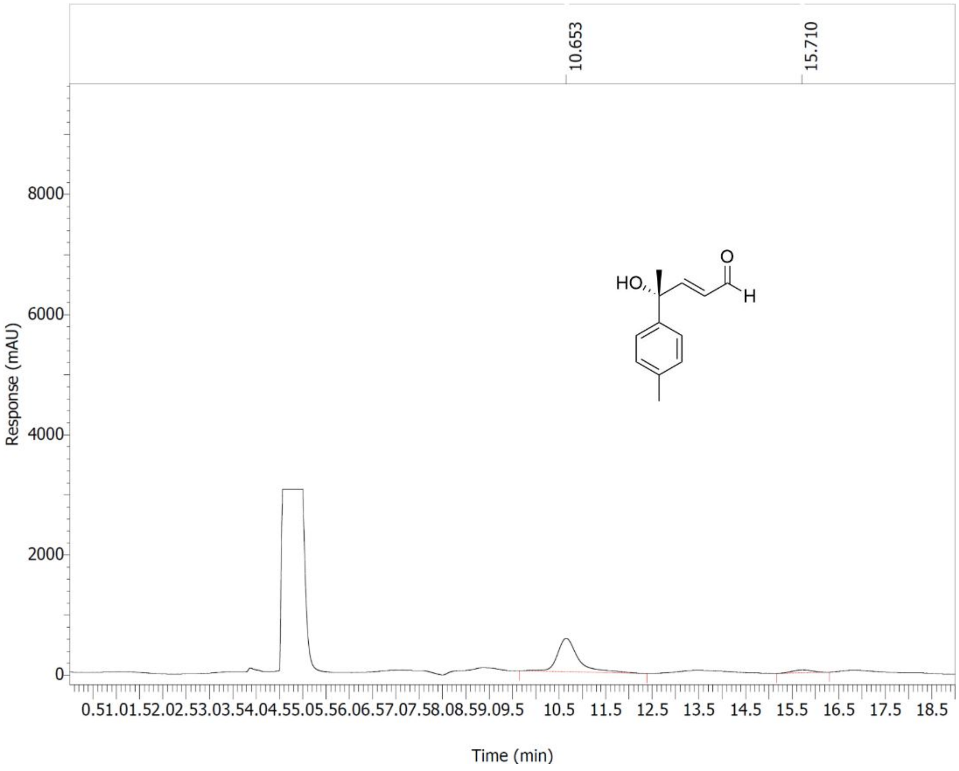

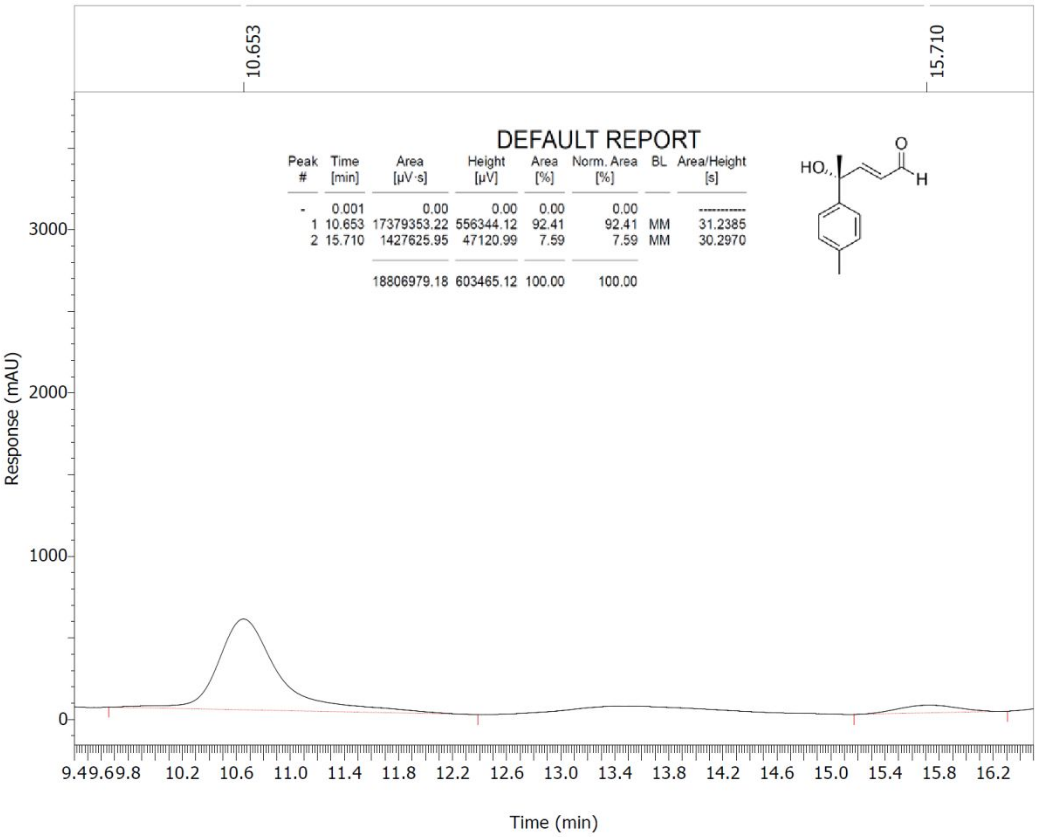

DEFAULT REPORT

Peak #	Time [min]	Area [µV·s]	Height [µV]	Area [%]	Norm. Area [%]	BL	Area/Height [s]
-	0.001	0.00	0.00	0.00	0.00		-----------
1	10.653	17379353.22	556344.12	92.41	92.41	MM	31.2385
2	15.710	1427625.95	47120.99	7.59	7.59	MM	30.2970
		18806979.18	603465.12	100.00	100.00		

XII. References

(1) Ghosh, P.; "Acid-catalyzed air-oxidative fragmentation of the carbon-carbon bond in 2-aryl-1-tetralones." *ACS Omega,* **2019**, *4*, 8065-8070.

(2) Arimitsu, S.; Nakasone, M. Stereoselective Organocatalytic Synthesis of α,α-Difluoro-γ,γ-Disubstituted Butenals. *J. Org. Chem.* **2016**, *81* (15), 6707–6713. https://doi.org/10.1021/acs.joc.6b01334.

(3) Meyers, A.I.; Walkup, R. D. Diastereofacil Selectivity via Aldol Reactions Using Ethyl Dithioacetate and Ethyl Dithiopropionate Enolates. *Tetrahedron,* **1985**, *41* (22), 5089-5106.

(4) Briand, M.; Thai, L. D.; Bourdreux, F.; Vanthuyne, N.; Moreau, X.; Magnier, E.; Anselmi, E.; Dagousset, G. Remote Radical Trifluoromethylation: A Unified Approach to the Selective Synthesis of γ-Trifluoromethyl α,β-Unsaturated Carbonyl Compounds. *Org. Lett.* **2022**, *24* (51), 9375–9380.

(5) Zhu, X.; Li, R.; Yao, H.; Lin, A. Palladium-Catalyzed Allenamide Carbopalladation/Allylation with Active Methine Compounds. *Org. Lett.* **2021**, *23* (12), 4630–4634. https://doi.org/10.1021/acs.orglett.1c01369.

(6) Zhang, H.-J.; Schuppe, A. W.; Pan, S.-T.; Chen, J.-X.; Wang, B.-R.; Newhouse, T. R.; Yin, L. Copper-Catalyzed Vinylogous Aerobic Oxidation of Unsaturated Compounds with Air. *J. Am. Chem. Soc.* **2018**, *140* (15), 5300–5310. https://doi.org/10.1021/jacs.8b01886.

(7) Guan, Q.; Ji, Y.; Zhao, Q.; Zhang, C. Copper-Catalyzed Highly Enantioselective 1,4-Protoboration of Terminal 1,3-Dienes. *CCS Chem* **2022**, *4* (5), 1545–1556. https://doi.org/10.31635/ccschem.021.202100947.

(8) Stevens, J. M.; MacMillan, D.W.C. Enantioselective α-Alkenylation of Aldehydes with Boronic Acids via the Synergistic Combination of Copper(II) and Amine Catalysis. *J. Am. Chem. Soc*. **2013**, *135*, 11756−11759.

(9) Xu, X.X.; Dong, H.Q. Enantioselective Total Syntheses and Stereochemical Studies of All Four Stereoisomers of Yingzhaosu C. *J. Org. Chem*. **1995**, *60*, 3039-3044.
